# Genome-Wide Identification Analysis of the 4-Coumarate: Coa Ligase (4CL) Gene Family in *Brassica* U’s Triangle Species and Its Potential Role in the Accumulation of Flavonoids in *Brassica napus* L.

**DOI:** 10.3390/plants14050714

**Published:** 2025-02-26

**Authors:** Mengzhen Zhang, Mengjiao Tian, Ziwuyun Weng, Yaping Yang, Nian Pan, Shulin Shen, Huiyan Zhao, Hai Du, Cunmin Qu, Nengwen Yin

**Affiliations:** 1 Integrative Science Center of Germplasm Creation in Western China (CHONGQING) Science City, College of Agronomy and Biotechnology, Southwest University, Beibei, Chongqing 400715, China; zmz0110@email.swu.edu.cn (M.Z.); suwtmj@email.swu.edu.cn (M.T.); wzwy18716955096@email.swu.edu.cn (Z.W.); 15662619623@163.com (Y.Y.); p1666835803@email.swu.edu.cn (N.P.); ssl7159@email.swu.edu.cn (S.S.); zhaohuiyan@swu.edu.cn (H.Z.); haidu81@126.com (H.D.); 2 Academy of Agricultural Sciences, Southwest University, Chongqing 400715, China; 3 Engineering Research Center of South Upland Agriculture, Ministry of Education, Chongqing 400715, China

**Keywords:** 4-Coumarate: CoA ligase (4CL), U’s triangle, *Brassica napus*, expression analysis, flavonoids

## Abstract

4-Coumarate: CoA ligase (4CL) is a key branch point enzyme at the end of the phenylpropanoid metabolic pathway. It regulates the synthesis of various metabolites and participates in plant growth and development by catalyzing the formation of CoA ester compounds. However, *4CL* family members have not been identified and analyzed among U’s triangle species in *Brassica*. In this study, 53 *4CL* genes were identified in *Brassica* U’s triangle species and divided into 4 groups (group I, II, III and IV) according to phylogenetic relationship. Based on phylogenetics, gene structure, conserved motifs, chromosome localization and collinearity analysis, *4CLs* were relatively conserved in the evolution of *Brassica* U’s triangle species. The promoter region contains a large number of cis-acting elements, implying the functional diversity of *4CLs*. Further combining transcriptome data and reverse transcription quantitative PCR (RT-qPCR), we found that *Bna4CLs* have tissue specificity and can not only respond to exogenous phytohormone changes but also regulate the flavonoid biosynthetic pathway in the yellow- and black-seeded *B. napus*. Our results complement the lack of research on the *4CL* gene family in *Brassica*, clarify the sequence characteristics and functional diversity of these genes and lay a foundation for further exploration of *4CL* genes in response to abiotic stress and regulation of seed coat flavonoid accumulation.

## 1. Introduction

Flavonoids are important secondary metabolites in plants, which are present in various tissues (flowers, seeds, stems and fruits). They are involved in a variety of physiological activities in plants, such as protecting plants from ultraviolet rays [1], participating in the transport of the phytohormone growth hormone [2], reducing excessive free radicals in plant [3] and affecting the color of organs such as flowers, leaves and fruit [4]. 4-Coumarate: CoA ligase (4CL) is a crucial branch point enzyme in the phenylpropanoid metabolic pathway. It is located at the end of the phenylpropanoid pathway and can catalyze the synthesis of the corresponding coenzyme A, which is then catalyzed by oxygenase, reductase and transferase for the synthesis of various secondary metabolites [5,6].

To date, the *4CL* genes have been intensively studied, and members of its gene family have been identified in numerous plants, including Arabidopsis (*Arabidopsis thaliana* L.) Heynh.) [7], rice (*Oryza sativa* L.) [8], apple (*Malus pumila* Mill.) [9] and parsley (*Petroselinum crispum* (Mill.) Fuss) [10]. The amino acid sequence of 4CLs has been shown to contain two conserved polypeptide motifs, designated Box I (SSGTTGLPKGV) and Box II (GEICIRG) [11]. Box I is conserved at the N-terminal and can directly participate in the catalytic process, facilitating the binding of adenosine monophosphate (AMP). Moreover, it has been identified as a highly conserved domain, not only within the *4CL* gene family but across the entirety of adenylate-forming enzyme gene families [12]. In contrast, Box II exhibits a high degree of conservation exclusively within the *4CL* family, yet it does not directly participate in catalysis, and its function remains unclear [13]. Previously, the *4CL* genes of dicotyledonous plants were divided into two categories: Class I and Class II, which had different functions. Class I primarily contributes to lignin biosynthesis, while Class II is involved in flavonoid synthesis [12]. *A. thaliana* is the most widely studied plant, and among the genes in this species, *At4CL1*, *At4CL2* and *At4CL4* are categorized under Class I, while *At4CL3* is classified under Class II [14]. Of the five genes identified in rice, *Os4CL2* plays a role in regulating the synthesis of flavonoids and exhibits the highest expression levels in anther tissue [15]. *Md4CL10* and *Md4CL23* in the apple gene family had somewhat higher expression in materials with high anthocyanin content [9], and the expression of *Pg4CL2* in the 12 *4CL* genes of pomegranate had a similar trend of changes in flavonoid content, which may be involved in the synthesis of flavonoids and accumulation of pigments [16]. Studies have shown that a large number of reactive oxygen species are produced in plant cells under UV stress, drought, salt stress and ABA and MeJA hormone treatment. [17,18,19]. The *4CL* genes have been identified as a key factor in the response to a range of abiotic stresses and hormone treatments, regulating the content of flavonoids and thereby mitigating the detrimental effects induced by reactive oxygen species in vivo. For example, both *At4CL3* and *Os4CL2* as in Class II were significantly increased under UV irradiation [15,20], thus reducing the excessive production of reactive oxygen species and averting damage to plants.

There are many kinds of *Brassica* species, which are used as animal fodder, vegetable oil and biofuel resource [21,22,23]. “U’s triangle” theory describes the hybridization between three ancestral diploid species in *Brassica*, including three diploid ancestors, *Brassica rapa* (*B. rapa*, *Bra*, AA, 2n  =  20), *Brassica nigra* (*B. nigra*, *Bni*, BB, 2n = 16) and *Brassica oleracea* (*B. oleracea*, *Bol*, CC, 2n = 18), and three allotetraploid species, *Brassica juncea* (*B. juncea*, *Bju*, AABB, 2n = 36), *Brassica napus* (*B. napus*, *Bna*, AACC, 2n = 38) and *Brassica carinata* (*B. carinata*, *Bca*, BBCC, 2n = 34) [24,25,26]. The chromosome-scale genome assembly of these six species has been completed, so this theory offers a practical evolutionary model for studying gene families [22,23,27,28,29,30,31]. In recent years, multiple gene families have been reported among *Brassica* species in this theory, including the Sucrose nonfermenting 2 (Snf2) proteins family [32], the RAV gene family [33] and the HKT gene family [34].

Rapeseed (*Brassica napus* L.) is a significant oilseed crop globally, having become the most widely cultivated oilseed crop due to its high yield, large plant size and disease resistance [27]. Increasing rapeseed yield and improving rapeseed quality and cake quality has always been one of the important goals of rapeseed breeding. The traditional black-seeded *B. napus* has a thick seed coat, low oil and protein content, and contains more pigments, which is not convenient for processing and decolorization. The yellow-seeded *B. napus* improves the quality and weakens the shortcomings of black-seeded *B. napus*. At present, it has become an excellent germplasm resource that people pursue [35,36]. The production of yellow-seed is associated with the accumulation of flavonoids in the seed coat, especially proanthocyanidins and polyphenols [37]. In *B. napus*, many gene families related to flavonoid accumulation have been studied, such as *Dihydroflavonol 4-reductase* (DFR) [38], B-box (BBX) [39] and *Flavonol synthase* (FLS) [40]. However, there is a lack of analysis of the *4CL* gene family in *B. napus*.

In recent years, the identification of the *4CL* gene family has been widely studied, but the study in *Brassica* U’s triangle species has not been reported. This study aims to use bioinformatics methods, with *At4CLs* as the query genes, to discover *4CL* members, analyze phylogenetic relationships, gene structure, regulatory elements and tissue-specific expression, as well as investigate the expression profiles of *Bna4CLs* under phytohormone treatments, so as to establish a strong theoretical basis for the comprehensive analysis of the evolution of *4CLs* in *Brassica* U’s triangle. In addition, the role of *4CLs* in seeds, especially in yellow seed formation, has not been studied. If the expression of *Bna4CLs* in yellow- and black-seeded *B. napus* is different, it will offer fresh insights into the role of *4CLs.*

## 2. Results

### 2.1. Identification of 4CL Family Genes in the Brassica U’s Triangle Species

In order to obtain the 4CL-coding proteins in U ’s triangle species, we performed a preliminary BLASTP search using the At4CLs protein sequence. Except for the gene with incomplete structure (BniB021049-TA) (Figure S1), 53 candidate 4CLs in *Brassica* U’s triangle species were found. In order to simplify the gene name, we named these genes according to their chromosomal locations in different species, including 12 Bna4CLs, 13 Bju4CLs, 10 Bca4CLs, 7 Bra4CLs, 6 Bni4CLs and 5 Bol4CLs (Table 1). Physicochemical property analysis showed that the 4CL proteins length ranged from 483 to 592 aa. The molecular weight is between 52.43 and 64.98 kDa; the pI ranged from 5.16 to 8.79 (Table 1). The instability index ranged from 24.82 to 42.89, and structural stability was good in 75% of the 4CL proteins, with a score below 40 indicating stability [41]; subcellular localization analysis revealed that different 4CLs were localized differently, which might be related to the function of the 4CL proteins, with the most localized being the cytoplasmic, followed by the plasma membrane peroxisomal and chloroplast.

### 2.2. Phylogenetic Analysis of 4CL Proteins in A. thaliana and the Brassica U’s Triangle Species

**Figure 1:**
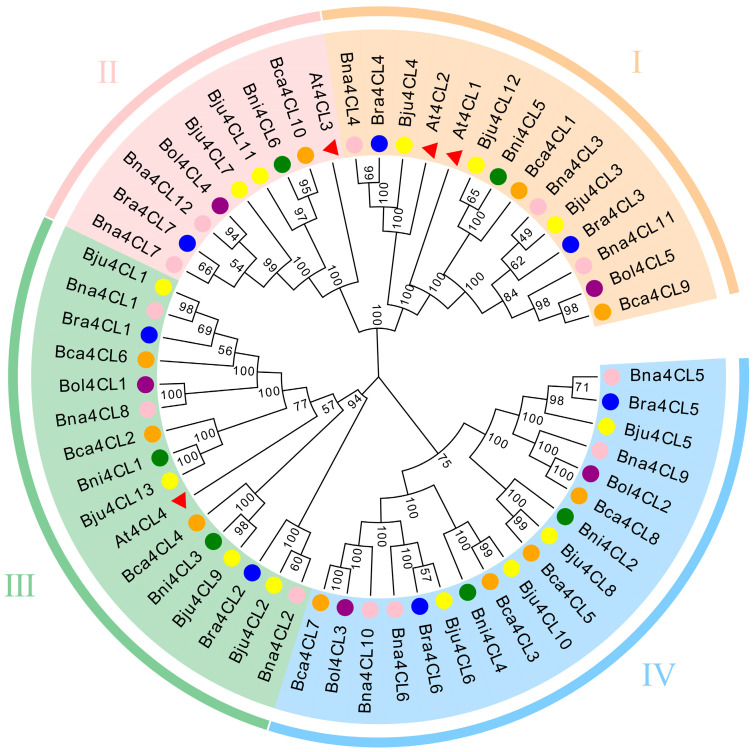
Phylogenetic tree of the 4CL protein family from *A. thaliana* and the *Brassica* U’s triangle species. Four groups (**I**–**IV**) are respectively represented in different colors: orange, pink, green and blue. *A. thaliana*, red triangle; *B. napus*, pink circle; *B. rapa*, blue circle; *B. juncea*, yellow circle; *B. oleracea*, purple circle; *B. carinata*, orange circle; *B. nigra*, green circle.

To explore the phylogenetic relationships of the 4CL proteins even further, we used the known 4CL protein sequences to create an NJ tree. According to the distance of genetic relationship and the clustering relationship with At4CLs we divided the 57 4CL proteins into 4 groups: groups I–IV (Figure 1). The genes in group I were clustered with At4CL1 and At4CL2, including 3 Bna4CLs (25%), 2 Bra4CLs, 3 Bju4CLs, 1 Bol4CLs, 2 Bca4CLs and 1 Bni4CLs; the genes in group III were clustered with At4CL4, including 3 Bna4CLs (25%), 2 Bra4CLs, 4 Bju4CLs, 2 Bca4CLs, 1 Bol4CLs and 2 Bni4CLs, indicating a potential association with the biosynthesis of lignin. The genes in group II were clustered with At4CL3, including 2 Bna4CLs (17%), 1 Bra4CLs, 2 Bju4CLs, 1 Bol4CLs, 1 Bca4CLs and 1 Bni4CLs, which were speculated to be related to flavonoid synthesis. Within the NJ tree, the quantity of Bna4CLs surpassed that of At4CLs by 1.5 times in group I, doubled in group II, and tripled in group III. This shows that the amplification rate is different according to the specific functional requirements in the evolution process. In addition, the remaining genes were not clustered with At4CLs and were assigned to group IV, including 4 Bna4CLs (33%), 2 Bra4CLs, 4 Bju4CLs, 2 Bol4CLs, 2 Bca4CLs and 2 Bni4CLs.

### 2.3. Conserved Protein Motifs and Gene Structures of 4CL Family Members

**Figure 2:**
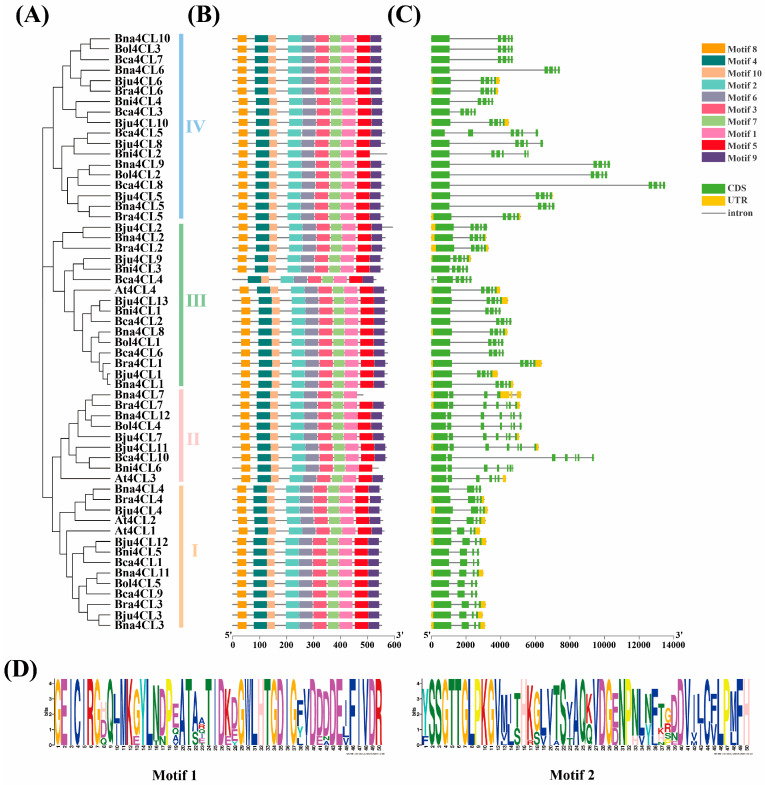
NJ tree, conserved motifs and gene structure analysis of 57 4CLs. (**A**) NJ tree of the 57 4CLs (identical to Figure 1). (**B**) Conserved motifs of 4CL proteins. The MEME website was used to obtain 10 motifs, which were illustrated with blocks of assorted colors. (**C**) Gene structures of *4CL* family genes. On the right side, different functional areas are displayed with different signs. (**D**) WebLogos of Motif 1 and Motif 2 for Figure 3B.

In order to explore the conservation between 57 4CL proteins more clearly, this study identified its conserved motifs through the MEME online website (Figure 2). Conserved motif analysis showed that fifty-three 4CL proteins all contained ten conserved motifs, indicating that they were still highly conserved during evolution. Four 4CL proteins had conserved motif deletions, among which Bni4CL2 and Bni4CL6 lacked Motif 9, Bna4CL7 lacked Motif 5 and Motif 9, and Bca4CL4 lacked Motif 8 (Figure 2B). In addition, Motif 1 contained Box II (GEICIRG) conserved polypeptide motif, Motif 2 contained Box I (SSGTTGLPKGV) conserved polypeptide motif (Figure 2D), and all 4CL proteins identified contain these two motifs, ensuring the catalytic activity of the 4CL enzyme.

Secondly, gene structure analysis found that *4CL* genes had multiple introns and exons (4–7). Members within the same group exhibited highly similar gene structures to their corresponding *At4CLs* homologs, as well as among themselves, with only a few exceptions. Notably, untranslated regions (UTRs) were identified at both the 5′ and 3′ ends of *Bol4CLs*, partially present in their allotetraploid counterparts (*Bna4CLs* and *Bju4CLs*), but entirely absent in the *4CL* genes of the remaining three *Brassica* species (Figure 2C). In general, the gene structure in each group is highly conserved.

### 2.4. Chromosomal Localization of 53 Brassica 4CL Genes

**Figure 3:**
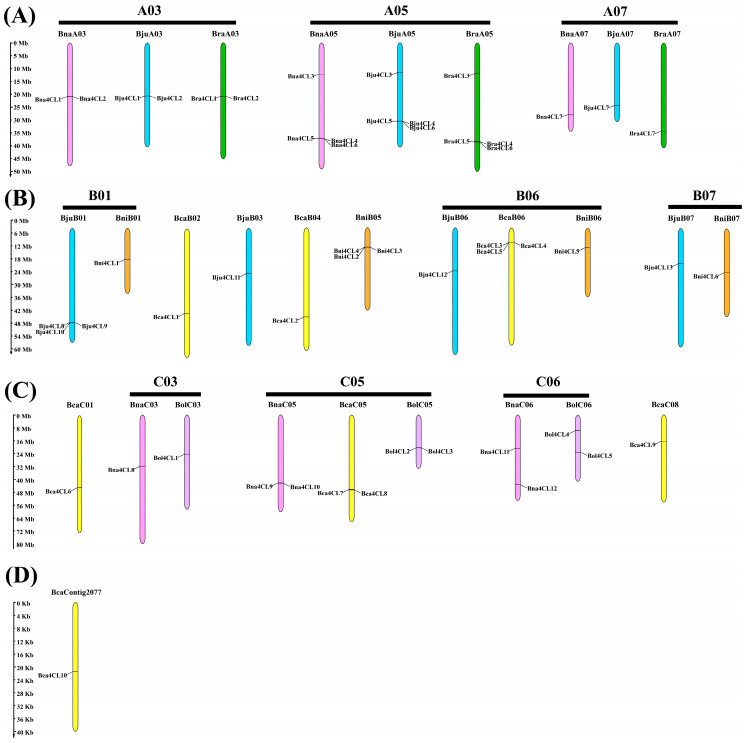
Chromosome distribution of 53 *4CL* genes. (**A**) *4CL* genes distributed on the A subgenome in *Bju*, *Bna* and *Bra*. (**B**) *4CL* genes distributed on the B subgenome in *Bju*, *Bca* and *Bni*. (**C**) *4CL* genes distributed on the C subgenome in *Bna*, *Bca* and *Bol*. (**D**) *Bca4CL10* distributed on the BcaContig2077. Chromosomes of different species use different colors: *Bna*, pink; *Bju*, blue; *Bra*, green; *Bni*, orange; *Bca*, yellow; *Bol*, purple.

In this study, we mapped 53 *4CL* genes on chromosomes; of which, 21 genes were found in the A subgenome, 17 in the B subgenome and 14 in the C subgenome (Figure 3A–C). In addition, *Bca4CL10* is located on contig and does not belong to one of the three subgenomes (Figure 3D). Notably, we observed that Bna, Bju and Bra have the most *4CL* genes on chromosome A05, with four genes each. Bni and Bca had the most *4CL* genes on B05 and Bca chromosomes, with three genes each, while Bol had the most and equal number of *4CL* genes on C05 and C06 chromosomes, with two genes each. These results indicate that the *4CL* gene is unevenly distributed on the chromosomes of the U’s triangle *Brassica* species. Meanwhile, it was discovered that the majority of these genes in the respective subgenome were situated in parallel positions, such as A03, A05, A07, B07, C03, C05 and C06, and even the number of genes was the same.

### 2.5. Collinearity Analysis of 4CL Genes in U’s Triangle Species

**Figure 4:**
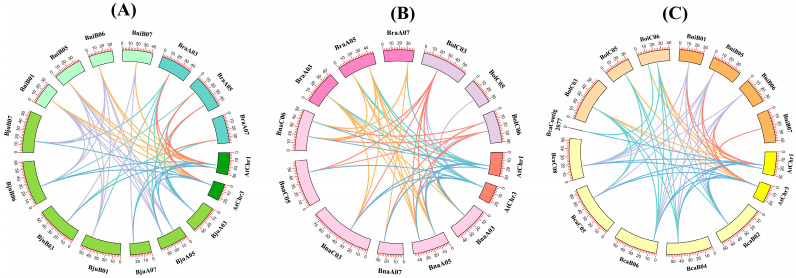
Collinearity analysis of *4CL* family genes between *A. thaliana* and U’s triangle species. (**A**) *4CLs* collinearity analysis among *A. thaliana*, *B. nigra*, *B. rapa* and *B. juncea*. (**B**) *4CL* collinearity analysis among *A. thaliana*, *B. oleracea*, *B. rapa* and *B. napus*. (**C**) *4CL* collinearity analysis among *A. thaliana*, *B. nigra*, *B. oleracea* and *B. carinata*.

According to previous studies, cruciferous species show significant collinearity in their protein-coding genes [42,43]. Therefore, we studied the collinearity between 57 *4CL* genes. Based on the evolutionary relationship, they were divided into three groups: *A. thaliana*, allotetraploid (*B. napus*, *B. juncea* and *B. carinata*) and two diploid ancestors (*B. rapa*, *B. oleracea* and *B. nigra*), and orthologous gene pairs were identified (Figure 4). It was found that there were 14, 7, 8, 19 and 13 pairs of orthologous genes in *A. thaliana* and *B. juncea*, *A. thaliana* and *B. nigra*, *A. thaliana* and *B. rapa*, *B. juncea* and *B. nigra*, and *B. juncea* and *B. rapa*, respectively. In group B, there were 14, 8, 6, 16 and 12 pairs of *A. thaliana* and *B. napus*, *A. thaliana* and *B. rapa*, *A. thaliana* and *B. oleracea*, *B. napus* and *B. rapa*, and *B. napus* and *B. oleracea*, respectively. In group C, there were 11, 7, 6, 13 and 13 pairs of *A. thaliana* and *B. carinata*, *A. thaliana* and *B. nigra*, *A. thaliana* and *B. oleracea*, *B. carinata* and *B. nigra*, and *B. carinata* and *B. oleracea*, respectively. These findings demonstrate that collinear *4CL* homologs exhibit widespread genomic distribution across *A. thaliana* and the U’s triangle species, highlighting their strong evolutionary conservation. In order to reveal the selection pressure between *4CL* homologous gene pairs, we calculated the numbers of nonsynonymous substitutions (Ka), synonymous substitutions (Ks), and the Ka/Ks ratios for the *4CL* gene pairs. The data revealed that all Ka/Ks ratios were below 1 (Table S2), indicating that all gene pairs had undergone purification selection.

### 2.6. Cis-Element Analysis of the Promoter Region of 4CLs in U’s Triangle Brassica Species

**Figure 5:**
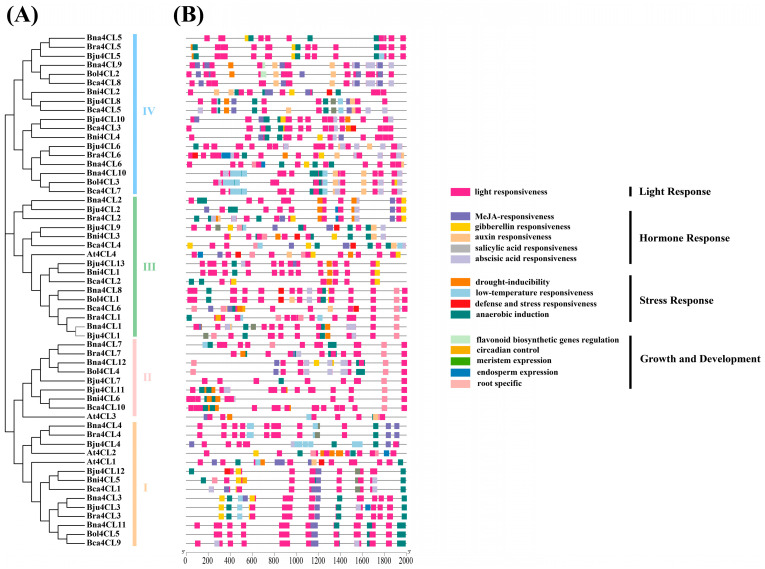
Predicted cis elements in the promoter region of *4CLs* in *A. thaliana* and U’s triangle *Brassica* species. (**A**) NJ tree of the 57 4CL proteins (identical to Figure 1). (**B**) *4CLs* promoter region cis-acting elements.

To clarify the potential role of *4CL*s in U’s triangle species, we extracted the 2000 bp region on the transcription start site of the 57 genes identified and predicted the cis-acting elements. Based on the function, they were categorized into four types, each of which is widely distributed on the promoter (Figure 5B). Among them, the light response element accounts for the largest proportion and exists in each gene promoter. Five cis-acting elements of hormone response were predicted, including MeJA, gibberellin, auxin, salicylic acid and abscisic acid responsiveness. Four stress responsive elements were identified, including defense and stress responsiveness, low-temperature responsiveness, drought and anaerobic inducibility. The growth and development class includes five types: flavonoid biosynthetic gene regulation, circadian control, meristem expression, endosperm expression and root specific. At the same time, combined with the phylogenetic tree (Figure 5A), we found that the closer the genetic relationship, the more similar the type and location of cis-acting elements.

### 2.7. Expression Profiles of Bna4CLs in Various Tissues and Periods

**Figure 6:**
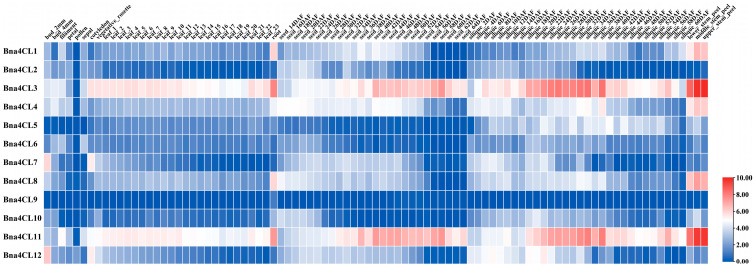
Tissue-specific expressions of *Bna4CLs*. The expression profiles of each *Bna4CLs* were represented as Log2 (FPKM value + 1). Different colors represent different expression levels (red: high; white: medium; blue: low).

According to the transcriptome data of each tissue site on the BnIR website, with the exception of the extremely low expression of *Bna4CL9*, almost all members of the *Bna4CLs* are expressed to varying extents in various tissue parts, and the expression levels are also different at different developmental periods. *Bna4CL7* and *Bna4CL12* demonstrated higher expression in flower buds, whereas the expression of other genes was comparatively lower. *Bna4CL3* and *Bna4CL11* showed significant expression in leaves, roots, seeds, siliques and stems. Moreover, *Bna4CL1*, *Bna4CL4* and *Bna4CL8* displayed significant transcriptional activity in roots and stems (Figure 6). The results showed that *Bna4CLs* except *Bna4CL9* had tissue specificity and played different functions in *B. napus*.

### 2.8. Expression Profiles of Bna4CLs Under Phytohormone Treatment

**Figure 7:**
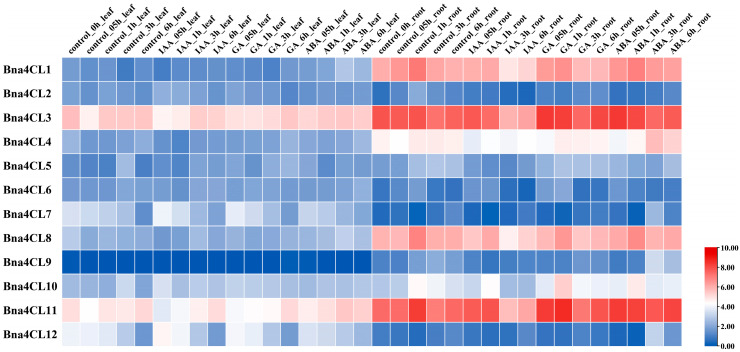
Expression profiles of *Bna4CLs* under phytohormone treatments. The heatmap of the *Bna4CLs* is represented as Log2 (FPKM value + 1). IAA, indole-3-acetic acid or heteroauxin; ABA, abscisic acid; GA, gibberellins. Labels 0.5 h, 1 h, 3 h and 6 h represent the hours after treatment.

Using publicly available RNA-seq data, we detected the expression of *4CL* genes in the leaf and roots of ZS11 seedlings under different phytohormone (IAA, GA and ABA) treatment (Figure 7). The results showed that *Bna4CL9* was not sensitive to phytohormones in the leaf. In addition to this gene, the other 11 genes were sensitive to hormones. However, the trends shown are different. In leaves, *Bna4CL3*, *Bna4CL4* and *Bna4CL11* showed a continuous decreasing trend after IAA treatment. Under IAA and GA treatments, the expressions of *Bna4CL7*, *Bna4CL10* and *Bna4CL12* exhibited an initial upregulation, followed by a downregulation phase at 3 h, and subsequently, resumed upregulation. At the same time, *Bna4CL5* also showed the same trend under GA treatment. *Bna4CL2* and *Bna4CL3* were up-regulated at 0.5 h and down-regulated at 1 h after GA treatment. *Bna4CL11* still showed a continuous downward trend under GA treatment. After ABA treatment, *Bna4CL1*, *Bna4CL4* and *Bna4CL8* showed a continuous increasing trend, and *Bna4CL5*, *Bna4CL6*, and *Bna4CL10* exhibited a sequential increase–decrease–increase pattern in their expression levels.

In roots, after IAA treatment, *Bna4CL1*, *Bna4CL2*, *Bna4CL4*, *Bna4CL5*, *Bna4CL7* and *Bna4CL8* showed a consistent downward trend, *Bna4CL3*, *Bna4CL6*, *Bna4CL9*, *Bna4CL10* and *Bna4CL11* initially increased and then decreased consistently, and all increased only 0.5 h after treatment. After GA treatment, *Bna4CL1*, *Bna4CL2* and *Bna4CL4* showed a consistent downward trend; *Bna4CL3*, *Bna4CL10* and *Bna4CL11* showed a consistent upward trend. After ABA treatment, *Bna4CL2*, *Bna4CL4*, *Bna4CL9* and *Bna4CL12* initially decreased and then increased consistently, but the time period of change was different.

### 2.9. Expression Patterns of Bna4CLs in Yellow- and Black-Seeded B. napus

**Figure 8:**
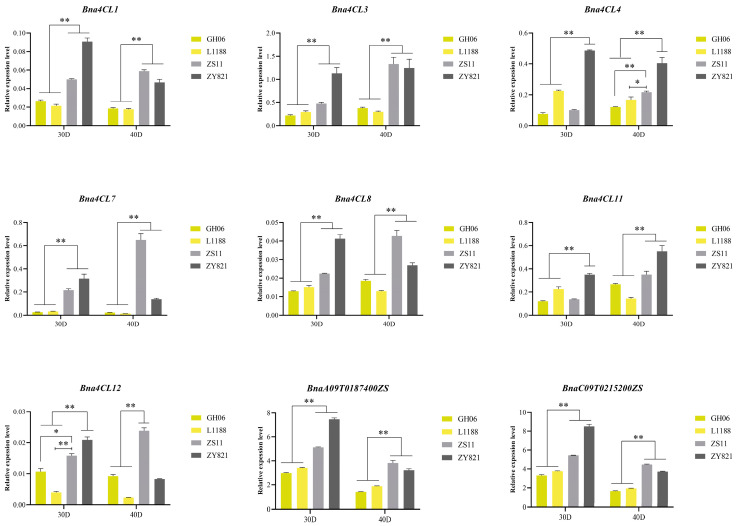
RT-qPCR of *Bna4CLs* and *BnaDFRs* in yellow- and black-seeded *B. napus*. Error bars represent the standard deviation (SD) of three biological replicates. Statistically significant differences were analyzed by Student’s *t*-test. * *p* < 0.05; ** *p* < 0.01.

Flavonoids, especially the biosynthesis of epicatechin and proanthocyanidins, exert a dominant influence on seed coat pigmentation in *B. napus* [44]. In this study, we performed RT-qPCR analysis of the expression levels of 7 *Bna4CLs* in two pairs of yellow-seeded *B. napus* (GH06 and L1188) and black-seeded *B. napus* (ZS11 and ZY821) at 30 and 40 days. As shown in the figure (Figure 8), a significant upregulation of *Bna4CL1*, *Bna4CL3*, *Bna4CL7* and *Bna4CL8* expression was detected in black-seeded plants compared to yellow-seeded plants. These findings suggest that the four genes contribute as positive regulators to the flavonoid pathway and may contribute to seed coat pigmentation. *Bna4CL4* and *Bna4CL11* in black-seeded accession were also significantly increased relative to those in yellow-seeded accession at 40 days of seed development, but the expression level in ZS11 was lower than that in yellow-seeded accession at 30 days of seed development, and ZY821 exhibited a notably higher expression level compared to two yellow-seeded accessions. *Bna4CL12* was expressed at a much higher level in black-seeded accessions than in yellow-seeded ones at 30 days, but at 40 days, only ZS11 had a significantly greater expression compared to yellow-seeded plants. As an important structural gene of the flavonoid metabolic pathway, *DFR* is one of the downstream genes of *4CL*. It is widely reported in the literature that *DFR* can affect the accumulation of flavonoids in *B. napus* [45]. We also performed RT-qPCR on its expression level in yellow- and black-seeded *B. napus* and found that the expression levels of two members (*BnaA09T0187400ZS* and *BnaC09T0215200ZS*) in black-seeded accession were always higher than those in yellow-seeded accession, so we speculated that some genes in *Bna4CLs* may also regulate the synthesis of flavonoids in seed coat.

## 3. Discussion

4CL, an important enzyme in the phenylpropanoid pathway, operates at the terminal stage of this metabolic process [46]. The accumulation of flavonoids, lignin and other metabolic compounds were affected by the activity of the 4CL enzyme. In addition, *4CL* genes can regulate plant growth and contribute to resistance against stresses [47]. Recently, the study of *4CL* gene family is expanding to more species. The count of identified *4CLs* varies from species to species, such as 2 in *P. crispum*, 4 in *A. thaliana*, 5 in *O. sativa* and 69 in *M. pumila* [7,8,9,10]. To date, the *4CL* gene family has yet to be reported in *Brassica* U ’s triangle. In this study, we identified 53 *4CL* members in the U’s triangle model, including 12 *Bna4CLs*, 13 *Bju4CLs*, 10 *Bca4CLs*, 5 *Bol4CLs*, 7 *Bra4CLs* and 6 *Bni4CLs* (Table 1). There were also differences in the number of *4CLs* in each species, and they all exceeded the number of *A. thaliana*. Allotetraploid species are formed by hybridization of diploid species, and the number of genes should also increase due to polyploidization [27,48]. Here, we also observed similar results. The number of *4CL* genes in allotetraploid crops *B. napus*, *B. juncea* and *B. carinata* is roughly equal to the sum of the number of *4CL* genes in their diploid ancestors *B. rapa* and *B. oleracea*, *B. rapa* and *B. nigra*, and *B. nigra* and *B. oleracea*. All *4CLs* homologous gene pairs Ka/Ks were below 1, showing that the *4CLs* in the U’s triangle experienced strong purification selection during evolution. A high degree of chromosomal collinearity was preserved in these genes after their divergence from a common ancestor (Figure 4). In summary, it shows that the evolutionary process might be relatively conservative. In addition, the gene structure and protein motifs of 4CLs remain relatively conserved within each group (Figure 2). The number of exons and introns is highly similar, and conserved motifs also exist in most genes. All the genes included Motif 1 and Motif 2, and these two motifs contained conserved polypeptide sequences Box I (SSGTTGLPKGV) and Box II (GEICIRG), which were consistent with the results of multiple sequence alignment (Figure S1), and also verified the results of previous studies, so that the 4CL enzyme can play its catalytic role.

According to the function, *4CL* genes are divided into two classes. Most of the *4CL* genes in dicotyledonous plants belong to Class I and are involved in lignin synthesis, such as *At4CL1* and *At4CL2* in *A. thaliana*, *Pt4CL1* in poplar and *Gm4CL2* in soybean. [14,47,49,50]. Class II, including the *4CL* genes of monocotyledonous plants, gymnosperms and some dicotyledonous plants, such as Arabidopsis *At4CL3*, Populus *Pt4CL2*, and rice *Os4CL2*, mainly regulate the accumulation of plant flavonoids and antitoxins [15,47,51]. In this study, 57 genes were divided into 4 groups according to their homology (Figure 1). Based on this result, we speculate that it also has similar functional characteristics in *B. napus*. *Bna4CL7* and *Bna4CL12* in *B. napus* may be related to flavonoid synthesis, while *Bna4CL1*, *Bna4CL2*, *Bna4CL3*, *Bna4CL4*, *Bna4CL8* and *Bna4CL11* are related to lignin synthesis. *4CL* genes are crucial for plant growth and development, and there are differences in expression at different plant tissues. *Os4CL2* may be related to flavonoid synthesis and is specifically expressed in rice anthers [15]. In apple, *Md4CL36*, *59* and *61* were highly expressed across all tissues, particularly in leaves and flowers [9]. In pomegranate, *Pg4CL1, 4, 5, 6* and *11* showed high expression levels in roots, leaves, flowers and pericarps [16]. The *4CLs* in *A. thaliana* are also tissue specific, *At4CL1* and *At4CL2* show the highest expression in seedling roots, whereas *At4CL3* is highly expressed in flowers [46]. Similar trends were also observed among *4CL* genes in *B. napus* (Figure 6). *Bna4CL3*, *Bna4CL4* and *Bna4CL11* were closely related to *At4CL1* and *At4CL2*, and the expression levels of the three genes were relatively high in plant roots. *Bna4CL1* and *Bna4CL8*, which are closely related to *At4CL4*, also are expressed at higher levels in roots. In addition to high expression in roots, it is also highly expressed in stems, especially *Bna4CL3* and *Bna4CL11*, mainly because *4CL1* contributes the most to lignin biosynthesis [46]. In summary, these genes show potential functional associations with lignin biosynthesis. However, *Bna4CL9* is an exception, and its function disappears during evolution, resulting in poor tissue specificity. The specific reasons need to be further explored.

Cis-acting elements within the promoter regions can control gene expression and enable genes to perform various functions. [52]. The cis-acting elements in *Bna4CLs* identified in this study were divided into four categories according to their functions, light, hormone, stress response and growth and development, indicating that they may regulate *B. napus* growth by being involved in the hormone regulation of abiotic stress responses (Figure 7). This is consistent with the results of previous studies. Light induction controls *At4CL3*, which in turn influences flavonoid synthesis in *A. thaliana* when exposed to blue light. [14,20]. Under the treatment of white light, UV, PEG, ABA and Me JA, the expression of 70% *St4CL* genes in potato up-regulated significantly or stayed the same [53]. The induction of *Nt4CL1* and *Nt4CL2* expression in tobacco was caused by wounding and MeJA treatment [54]. Under abiotic stress, it is easy to cause excessive accumulation of ROS, damage cell structure and function, and then hinder the growth and development of plants [17,19], while flavonoids have a strong ability to remove ROS in plants [3]. Here, the expression of *Bna4CLs* was also induced by a variety of phytohormones. In addition to the more previously studied treatments of ABA and GA, *Bna4CLs* was also regulated under IAA treatments. Among them, the expression levels of multiple *Bna4CLs* in the roots treated with these plant hormones showed a consistent trend. It shows that there is a certain similarity in the sensitivity of *Bna4CLs* to phytohormone. In addition, *Bna4CL7* and *Bna4CL12*, which are closely related to *At4CL3* and have collinearity, are speculated to have similar functions. The upregulation of these genes leads to increased flavonoid accumulation, improved ROS scavenging efficiency, and may enhanced abiotic stress tolerance in *B. napus*.

As a crucial enzyme in the phenylpropanoid pathway, *4CL* is essential for producing the compounds needed for flavonoid biosynthesis. [7,55]. During the development of mulberry fruit, the content of total flavonoids was first down-regulated and then up-regulated, and the content was higher in the later stage. This was similar to the expression pattern of *Ma4CL3*, which belongs to Class II of *4CLs*, during the whole fruit development process [56]. In apple, the increase in the expression level of most *4CL* genes was consistent with the deepening of peel color, indicating that the expression of *Md4CLs* showed a positive correlation with anthocyanin accumulation in apple peel [9]. The large number of advantages of yellow-seeded rapeseed have made it a hot topic in breeding research. In *B. napus*, yellow-seeded varieties accumulate less flavonoid pigments in their seed coats compared with black-seeded varieties [57]. Here, the results showed the expression levels of most *Bna4CLs* in black-seeded *B. napus* were significantly higher than those in yellow-seeded *B. napus*, indicating that *Bna4CLs* can positively regulate other key genes in the flavonoid metabolic pathway, thereby increasing the anthocyanin/proanthocyanidin content in the seed coat (Figure 8). In summary, this study focused on the structural characteristics of *4CLs* in *Brassica* U’s triangle species, analyzed the expression levels of *Bna4CLs* across various tissues, the expression profiles under exogenous phytohormone treatment, and the expression levels in yellow- and black-seeded *B. napus*, which laid a foundation for subsequent research.

## 4. Materials and Methods

### 4.1. Identification and Analysis of 4CLs

The 4CL protein sequences of *A. thaliana* and six *Brassica* species in the U’s triangle (*B. rapa*, *B. nigra*, *B. oleracea*, *B. juncea*, *B. napus* and *B. carinata*) were downloaded from the TAIR database (https://www.arabidopsis.org/ (accessed on 7 August 2024)) [58], the Brassica Database (BRAD, http://www.brassicadb.cn/ (accessed on 26 August 2024)) [59] and The Brassicaceae genome resource (TBGR, http://www.tbgr.org.cn/ (accessed on 16 January 2025)) [60], respectively. Four *At4CLs* (*AT1G51680.1*, *AT3G21240.1*, *AT1G65060.1* and *AT3G21230.1*) in *A. thaliana* were used as seed sequences to predict gene members in *Brassica* species [53]. The BLASTP program was used to compare the whole genome sequence data of 4CL family proteins, and the TBtools (v2.154)-Quick Find Best Homology function was used to search homologous genes [61]. Combined with the results of the two, the candidate genes were determined. In order to confirm whether the *4CL* candidate members really belong to *4CLs*, NCBI Conserved Domain Database (CDD) (https://www.ncbi.nlm.nih.gov/Structure/cdd (accessed on 27 August 2024)) and TBtools (v2.154)-Batch SMART were used for domain verification [61,62]. The CDD database contains the 4CL domain and the AFD _ class _ I superfamily domain, and the TBtools (v2.154)-Batch SMART database contains the AMP binding domain as the final member. In order to analyze whether the identified 4CLs protein sequence is missing, MEGA 11 was used for Clustalw multiple sequence alignment, and the alignments were visualized by ESPript 3.0 (https://espript.ibcp.fr/ESPript/cgi-bin/ESPript.cgi (accessed on 16 January 2025)) [63,64]. For *Bca4CL1*, *Bca4CL3* and *Bca4CL6* with poor gene structure annotation, we used the softberry website (http://www.softberry.com/berry.phtml (accessed on 9 February 2025)) to correct their gene structure annotation. Tbtools (v2.154)-Protein Paramter Calc was utilized to predict the length, molecular weight (MW, kDa), isoelectric point (pI) and stability index of each 4CL protein sequence [61]. The WoLF PSORT website (https://wolfpsort.hgc.jp/ (accessed on 9 November 2024)) was used to predict protein subcellular localization [65].

### 4.2. Phylogenetic Analysis of 4CL Gene Family Members

The phylogenetic tree was constructed using MEGA 11 software [64]. A total of 57 4CL protein sequences were aligned using ClustalW, and a Neighbor-Joining (NJ) phylogenetic tree was constructed based on the alignment results with 1000 bootstrap replicates, while other parameters were set to default. Then Evolview v3 (http://www.evolgenius.info/evolview/ (accessed on 9 September 2024)) was used for beautification [66]. Groupings were added to the phylogenetic tree and different symbols and colors were used to represent different species.

### 4.3. Gene Structure and Conserved Motifs Analysis of the 4CL Family Members

The online program Multiple Expectation Maximization for Motif Elucidation (MEME, https://meme-suite.org/meme/doc/meme.html (accessed on 23 September 2024)) was utilized to identify and analyze the conserved motifs of *4CL* family members [67], with a maximum number of motifs of 10. TBtools (v2.154)-Gene Structure View was used to visualize the 4CL phylogenetic tree, obtained motifs and gene structure [61].

### 4.4. Chromosomal Localization and Colinearity Analysis of 4CL Genes

The chromosomal location information of *4CL* family genes was extracted using the genome sequence annotation of *Brassica* species. Then we used the online website MG2C_v2.1 (http://mg2c.iask.in/mg2c_v2.1/ (accessed on 26 September 2024)) to visualize the chromosome localization of the *4CL* genes [68]. In addition, the tandem repeats were identified according to the physical location of the *4CL* tandem repeats on a single chromosome. In order to better understand the collinearity relationship, *Brassica* species were divided into three groups, and the homologous relationship between *4CL* genes was analyzed by TBtools (v2.154)-One Step MCScanX and TBtools (v2.154)-Advanced Circos [61]. Then TBtools (v2.154)-Simple Ka/Ks Calculator was used to calculate the non-synonymous substitution (Ka), synonymous substitution (Ks), and Ka/Ks ratio of homologous genes to estimate the selection pressure during evolution [61].

### 4.5. Cis-Element Analysis of the Promoter Region of 4CLs Genes

Tbtools was used to obtain the 2000 bp region at the 5’ end of the translation initiation site of each *4CL* gene as the promoter sequence [61]. The cis-acting elements were analyzed using the Plant CARE website (http://bioinformatics.psb.ugent.be/webtools/plantcare/html/ (accessed on 11 September 2024)) [69]. The results were systematically categorized and subsequently visualized through the TBtools (v2.154)-Simple BioSequence Viewer [61].

### 4.6. Expression Profile Analysis of 4CL Genes

In this study, RNA-seq data were derived from *Brassica napus* multi-omics information resource (BnIR, https://yanglab.hzau.edu.cn/BnIR (accessed on 16 January 2025)) to study the plant hormone response expression pattern of *Bna4CLs*. *B. napus* Zhongshuang 11 (ZS11) was grown in a Petri dish with wet filter paper for 2 days. The seedlings that had germinated were placed in Hoagland nutrient solution for 12 days. After two weeks, the seedlings were moved to Hoagland nutrient solution with or without hormones for further treatment. Samples were collected at 0, 0.5, 1, 3 and 6 h, and leaves and roots were frozen separately. The exogenous phytohormones were IAA (10 μM), ABA (10 μM) and GA3 (10 μM) [70]. Transcriptome data of each tissue were also obtained from BnIR (accessed on 12 October 2024) [71]. The heat map was generated by TBtools (v2.154)-HeatMap [61].

### 4.7. RT-qPCR Analysis of Bna4CLs

The primers of *Bna4CLs* required for RT-qPCR were designed using the website BrassicaEDB (https://qprimerdb.biodb.org/ (accessed on 18 October 2024)). Total RNA was extracted from seeds of *B. napus* (GH06, L1188, ZS11 and ZY821) at 30 and 40 days after flowering using the EZ-10 DNAaway RNA Mini-Preps Kit (Shenggong, Shanghai, China). The *B. napus* plants were cultivated under natural environmental conditions in the experimental field on the campus of Southwest University in Chongqing. The cDNA was obtained by reverse transcription using ExonScript RT Mix (with dsDNase) kit (Baoguang, Chongqing, China) reverse transcription kit. RT-qPCR was performed using Bio-Rad CFX96 Real Time System (Bio-Rad Laboratories, Hercules, CA, USA) (Baoguang, Chongqing, China), and three replicates of RT-qPCR were performed. Final data normalization involved applying the 2^−ΔΔCT^ calculation method, where all experimental values were ratio adjusted relative to the expression levels of BnaActin7 (EV116054) as the internal standard [44,72].

## 5. Conclusions

Here, 53 *4CL* genes were identified in U’s triangle *Brassica* species. They contained different numbers in different species, with different protein length, MW, pI and stability, but they experienced strong purification selection in evolution. According to the phylogenetic relationship, the division resulted in four groups, with genes in each group showing significant structural and functional resemblance. *Bna4CLs* show specificity in different tissues of *B. napus*, thus exerting specific functions. The expression profile under phytohormone treatment showed that *Bna4CLs* were sensitive to phytohormones, and the presence of a large number of hormone-responsive cis-acting elements in the promoter region supported this conclusion. RT-qPCR analysis showed that the expression levels of some genes (*Bna4Cl1*, *Bna4Cl3*, *Bna4Cl7* and *Bna4Cl8*) in black-seeded *B. napus* were higher than those in yellow-seeded *B. napus*. The trend was the same as that of the downstream gene *BnaDFRs,* which could regulate the change in grain color, indicating that *4CL* genes could not only regulate the accumulation of flavonoids in flowers and leaves, but also had a certain regulatory effect on the accumulation of flavonoids in seed coat, which filled the lack of function of *4CL* in seeds and provided a new idea for future research. In summary, our results systematically revealed the characteristics of 4CL family proteins and the diversity of gene functions in U’s triangle species, which laid a basis for the analysis of *4CLs* in *Brassica* in the future, especially for elucidating the participation in response to abiotic stress and regulation of flavonoid synthesis in seed coat, and the study of its mechanism of action will become the focus of future research.

## Figures and Tables

**Table 1:** Physicochemical properties of identified *4CLs*.

Gene Name	Gene ID	Location of Chromosome	Length (aa)	Molecular Weight (kDa)	Theoretical pI	Instability Index	Predicted Subcellular Location
*Bna4CL1*	BnaA03T0366500ZS	BnaA03	572	62.98	5.31	36.03	Chloroplast
*Bna4CL2*	BnaA03T0366600ZS	BnaA03	565	61.81	5.2	27	Cytoplasmic
*Bna4CL3*	BnaA05T0171400ZS	BnaA05	552	60.05	5.38	40.63	Plasma membrane
*Bna4CL4*	BnaA05T0345400ZS	BnaA05	552	59.99	5.81	38.01	Plasma membrane
*Bna4CL5*	BnaA05T0345500ZS	BnaA05	559	61.12	5.59	35.7	Cytoplasmic
*Bna4CL6*	BnaA05T0345800ZS	BnaA05	553	60.90	8.59	31.17	Peroxisomal
*Bna4CL7*	BnaA07T0281800ZS	BnaA07	483	52.43	5.39	38.52	Cytoplasmic
*Bna4CL8*	BnaC03T0447200ZS	BnaC03	572	62.96	5.46	34.17	Chloroplast
*Bna4CL9*	BnaC05T0372300ZS	BnaC05	562	61.36	5.34	33.46	Chloroplast
*Bna4CL10*	BnaC05T0372600ZS	BnaC05	553	60.71	7.63	29.85	Peroxisomal
*Bna4CL11*	BnaC06T0113600ZS	BnaC06	551	59.97	5.46	42.1	Plasma membrane
*Bna4CL12*	BnaC06T0322800ZS	BnaC06	557	60.48	5.8	41.14	Cytoplasmic
*Bju4CL1*	BjuVA03G41320	BjuA03	572	62.94	5.31	35.81	Chloroplast
*Bju4CL2*	BjuVA03G41330	BjuA03	592	64.98	5.62	24.82	Cytoplasmic
*Bju4CL3*	BjuVA05G19140	BjuA05	552	60.10	5.26	41.77	Plasma membrane
*Bju4CL4*	BjuVA05G27610	BjuA05	552	60.08	5.7	39.49	Plasma membrane
*Bju4CL5*	BjuVA05G27640	BjuA05	559	61.09	5.57	34.47	Cytoplasmic
*Bju4CL6*	BjuVA05G27670	BjuA05	553	60.85	8.71	30.26	Peroxisomal
*Bju4CL7*	BjuVA07G32970	BjuA07	564	61.39	5.99	39.49	Cytoplasmic
*Bju4CL8*	BjuVB01G33150	BjuB01	564	62.36	5.81	30.6	Cytoplasmic
*Bju4CL9*	BjuVB01G33160	BjuB01	557	61.27	5.21	30.74	Cytoplasmic
*Bju4CL10*	BjuVB01G33250	BjuB01	557	60.83	6.17	29.81	Peroxisomal
*Bju4CL11*	BjuVB03G38620	BjuB03	569	61.83	5.92	42.89	Cytoplasmic
*Bju4CL12*	BjuVB06G27310	BjuB06	551	59.96	5.39	41.16	Plasma membrane
*Bju4CL13*	BjuVB07G27300	BjuB07	572	62.83	5.58	33.78	Plasma membrane
*Bca4CL1*	BcaB02g11270	BcaB02	542	58.95	5.44	40.67	Plasma membrane
*Bca4CL2*	BcaB04g19154	BcaB04	572	62.64	5.37	33.56	Plasma membrane
*Bca4CL3*	BcaB06g26203	BcaB06	557	60.88	6.17	29.81	Plasma membrane
*Bca4CL4*	BcaB06g26209	BcaB06	531	58.08	5.25	30.49	Cytoplasmic
*Bca4CL5*	BcaB06g26210	BcaB06	564	62.09	6.21	30.53	Cytoplasmic
*Bca4CL6*	BcaC01g03516	BcaC01	563	61.71	5.3	35.73	Chloroplast
*Bca4CL7*	BcaC05g28268	BcaC05	553	60.71	7.63	29.85	Peroxisomal
*Bca4CL8*	BcaC05g28272	BcaC05	562	61.36	5.34	33.46	Chloroplast
*Bca4CL9*	BcaC08g43929	BcaC08	551	59.97	5.46	42.1	Plasma membrane
*Bca4CL10*	BcaNung05537	BcaContig2077	570	61.86	5.85	39.23	Cytoplasmic
*Bra4CL1*	BraA03g040280.4.1C.1	BraA03	574	63.09	5.26	35.51	Chloroplast
*Bra4CL2*	BraA03g040290.4.1C.1	BraA03	565	62.01	5.27	27.11	Cytoplasmic
*Bra4CL3*	BraA05g018900.4.1C.1	BraA05	552	60.09	5.38	39.21	Plasma membrane
*Bra4CL4*	BraA05g027970.4.1C.3	BraA05	557	60.64	5.57	38.77	Plasma membrane
*Bra4CL5*	BraA05g027990.4.1C.1	BraA05	559	61.20	5.65	35.58	Cytoplasmic
*Bra4CL6*	BraA05g028020.4.1C.3	BraA05	553	60.85	8.79	29.72	Peroxisomal
*Bra4CL7*	BraA07g032760.4.1C.1	BraA07	564	61.39	5.81	40.27	Cytoplasmic
*Bni4CL1*	BniB021051-TA	BniB01	572	62.84	5.5	34.19	Cytoplasmic
*Bni4CL2*	BniB003144-TA	BniB05	571	62.73	5.16	31.82	Chloroplast
*Bni4CL3*	BniB003145-TA	BniB05	557	61.21	5.26	32.6	Cytoplasmic
*Bni4CL4*	BniB003147-TA	BniB05	557	60.96	6.05	29.16	Peroxisomal
*Bni4CL5*	BniB046377-TA	BniB06	551	59.93	5.39	40.73	Plasma membrane
*Bni4CL6*	BniB049482-TA	BniB07	539	58.25	5.39	38.22	Plasma membrane
*Bol4CL1*	Bol026623	BolC03	572	62.96	5.46	34.17	Chloroplast
*Bol4CL2*	Bol038387	BolC05	562	61.36	5.34	33.46	Chloroplast
*Bol4CL3*	Bol038389	BolC05	553	60.71	7.63	29.85	Peroxisomal
*Bol4CL4*	Bol012584	BolC06	557	60.48	5.8	41.14	Cytoplasmic
*Bol4CL5*	Bol031583	BolC06	551	59.97	5.46	42.1	Plasma membrane
*At4CL1*	AT1G51680.1	AtChr1	561	61.05	5.25	35.93	Chloroplast
*At4CL2*	AT3G21240.1	AtChr3	556	60.84	5.39	40.42	Plasma membrane
*At4CL3*	AT1G65060.1	AtChr1	561	61.31	5.91	36.9	Chloroplast
*At4CL4*	AT3G21230.1	AtChr3	570	62.56	5.52	33.24	Vacuolar

Bna, *B. napus*; Bju, *B. juncea*; Bca, *B. carinata*; Bra, *B. rapa*; Bni, *B. nigra*; Bol, *B. oleracea*; At, *A. thaliana*; aa, amino acid residues; pI, isoelectric point.

## Data Availability

All additional datasets supporting the findings of this study are included within the article and Supplementary Materials.

## Supplementary Materials

The following supporting information can be downloaded at: https://www.mdpi.com/article/10.3390/plants14050714/s1, Table S1: Primers used for RT-qPCR analysis of seven *Bna4CLs* and *BnaDFRs*; Table S2: The Ka/Ks ratio of *4CLs* homologous gene pairs in the U’s triangle; Figure S1: Multiple sequence alignment of 4CLs in the U’s triangle; Figure S2: Amplification efficiency of RT-qPCR; Figure S3: Structural annotation correction of *Bca4CL1*, *Bca4CL3* and *Bca4CL6* genes.
