# Supplementary material for: Genome-Wide Identification Analysis of the 4-Coumarate: Coa Ligase (4CL) Gene Family in Brassica U’s Triangle Species and Its Potential Role in the Accumulation of Flavonoids in Brassica napus L
Source: Plants (Basel). 2025 Feb 26;14(5):714. doi: 10.3390/plants14050714 (PMC11902127; doi:10.3390/plants14050714)
Supplement: Supplementary file 1 [file plants-14-00714-s001.zip › Supplementary Materials/Figure S1/Figure S1.pdf]

```
AT1G51680.1            1  MAPQEQAVSQVMEKQSNNNN............SDVIFRSKLPDIYIPNHLSLHDYIFQNISE
AT3G21240.1            1  MTTQDVIVNDQNDQKQCSN..............DVIFRSRLPDIYIPNHLPLHDYIFENISE
AT1G65060.1            1  MITAALHEP.QIHKPTDTSVVSDDVLPHSPPTP.RIFRSKLPDIDIPNHLPLHTYCFEKLSS
AT3G21230.1            1  MVLQQQ..THFLTKKIDQE.....DEEEEPSH.DFIFRSKLPDIFIPNHLPLTDYVFQRFSG
BnaA03T0366500ZS       1  MVLQQQQTLFVTKKTDQELP..LKTDPEPSH.EFIFRSKLPDISIPNHLPLTDYVFQKFSG
BnaA03T0366600ZS       1  .MLSRQQETLFLTKKLDQE...........PCQDIIFRSKLPDIFIPNHLSLTDYVFQRFSG
BnaA05T0171400ZS       1  MAPQE....DAMQKQSSNNN............SDVIFRSKLPDIYIPNHLPLHDYIFQNISE
BnaA05T0345400ZS       1  MSTREETVT...DQKHSSA..............DVIFRSKLPDIHISNHLSLHDYIFQNISE
BnaA05T0345500ZS       1  MALLQQQETLSLAK..NQEP...........SQDFIFRSKLPDISIPNHLPLTDYLFQKFSG
BnaA05T0345800ZS       1  .MAFRQQEALSLTKKTDQD...............FIFRSKLPDISIPNHLPLTEYVFQKFSG
BnaA07T0281800ZS       1  MITATLQEP.QIHQPVDTTT....PPTDAPPTPPRIFRSKLPDIDIPNHLPLHTYCFEKLPS
BnaC03T0447200ZS       1  MVLQQQQTLFVTKKTDQELP..LKTDPEPSR.EFIFRSKLPDISIPNHLPLTDYVFQKFSG
BnaC05T0372300ZS       1  MALLRQQETLSLAK..NQEP...........SQDFIFRSKLPDISIPNHLSLTDYLFQKFSG
BnaC05T0372600ZS       1  .MAFRQQETLSLTKKTDQD...............FIFRSKLPDISIPNHLPLAEYVFQKFSG
BnaC06T0113600ZS       1  MAPQE....DAMQKQSSNK.............SDVIFRSKLPDIYIPNHLPLHDYIFQNISE
BnaC06T0322800ZS       1  MITATLQEP.QIHQPVDTTT....PPADAPPTPPRIFRSKLPDIDIPNHLPLHNYCFQKLSS
BjuVA03G41320          1  MVLQQQQTLFVTKKTDQELP..LKTDPEPSH.EFIFRSKLPDISIPNHLPLTDYVFQKFSG
BjuVA03G41330          1  .MLSRQQETLFLTKKLDQE...........PCQDIIFRSKLPDIFIPNHLSLTDYVFQRFSG
BjuVA05G19140          1  MAPQE....DAMQKQSSNNN............SDVIFRSKLPDIYIPNHLPLHDYIFQNISE
BjuVA05G27610          1  MSTREETVT...DQKHSSA..............DVIFRSKLPDIYIPNHLPLHDYIFQNISE
BjuVA05G27640          1  MALLQQQETLSLAK..NQEP...........SQDFIFRSKLPDISIPNHLPLTDYLFQKFSG
BjuVA05G27670          1  .MAFRQQEALSLTKKTDQD...............FIFRSKLPDISIPNHLPLTQYVFQKFSG
BjuVA07G32970          1  MITATLQEP.QIHQPVDTTT....PPTDAPPTPPRIFRSKLPDIDIPNHLPLHTYCFQKLSS
BjuVB01G33150          1  .MALRQQETLSLAKKTDQEP...........SQDIIFRSKLPDISIPNHLPLIEYIFHRFSG
BjuVB01G33160          1  .MALPQQETLSLTKKHSQD...............FIFRSKLPDISIPNHLSLTDYVFQKFSG
BjuVB01G33250          1  .MAFRQQEALSLTKKTDQDP...........SQDCIFRSKLPDISIPNHLPLTDYIFQKFAG
BjuVB03G38620          1  MITATLQEPHEIHKPVDTTS...SPATDAPPTPPRIFRSKLPDIDIPNHLPLHTYCFEKLPS
BjuVB06G27310          1  MAPQE....DAMQKQSSDN.............SDVIFRSKLPDIYIPNHLPLHDYIFQNISE
BjuVB07G27300          1  MVLQQQQTLFLTKKTDQELS..LKTDQEPSHDEFIFRSKLPDISIPNHLSLTDYVFQKFSG
BcaB02g11270           1  MAPQE....DAMQKQSSDN.............SDVIFRSKLPDIYIPNHLPLHDYIFQNISE
BcaB04g19154           1  MVLQQQQTLFLTKKTDQELS..VKTDQEPSHDEFIFRSKLPDISIPNHLSLTDYVFQKFSG
BcaB06g26203           1  .MAFRQQEALSLTKKTDQDP...........SQDLIFRSKLPDISIPNHLPLTDYIFQKFSG
BcaB06g26209           1  ...................................MFCKTLP.......LGVHYKTGLKFSG
BcaB06g26210           1  .MALRQQETLSLAKKTDQEP...........SQDIIFRSKLPDISIPNHLPLIEYIFHRFSG
BcaC01g03516           1  MVLQQQQTLFVTKKTDQELP..LKTDPEPSR.EFIFRSKLPDISIPNHLPLTDYVFQKFSG
BcaC05g28268           1  .MAFRQQETLSLTKKTDQD...............FIFRSKLPDISIPNHLPLAEYVFQKFSG
BcaC05g28272           1  MALLRQQETLSLAK..NQEP...........SQDFIFRSKLPDISIPNHLSLTDYLFQKFSG
BcaC08g43929           1  MAPQE....DAMQKQSSNK.............SDVIFRSKLPDIYIPNHLPLHDYIFQNISE
BcaNung05537           1  MITATLQEPHEIHKPVDTTTT..SPTTDAPPTPPRIFRSKLPDIDIPNHLPLHTYCFEKLPS
BraA03g040280.4.1C.1   1  MVLQQQQTLFVTKKTDQELP..LKTDPEPSH.DFIFRSKLPDISIPNHLPLTDYVFQKFSG
BraA03g040290.4.1C.1   1  .MLSRQQETLFLTKKLDQE...........PRQDFIFRSKLPDIFIPNHLPLTDYVFQRFSG
BraA05g018900.4.1C.1   1  MAPQE....DAMQKQSSNNN............SDVIFRSKLPDIYIPNHLPLHDYIFQNISE
BraA05g027970.4.1C.3   1  MSTREETVT...DQKHSSA..............DVIFRSKLPDIHISNHLSLHDYIFQNISE
BraA05g027990.4.1C.1   1  MALLQQQETLSLAK..NQEP...........SQDFIFRSKLPDISIPNHLPLTDYLFQKFSG
BraA05g028020.4.1C.3   1  .MAFRQQEALSLTKKTDQD...............FIFRSKLPDISIPNHLPLTEYVFQKFSG
BraA07g032760.4.1C.1   1  MITATLQEP.QIHQPVDTTT....PPTDAPPTPPRIFRSKLPDIDIPNHLPLHTYCFQKLSS
BniB021049-PA          1  .........MFLT.........................SSSPITF....LSLT.......TS
BniB021051-TA          1  MVLQQQQTLFLTKKTDQELS..LKTDQEPSHDEFIFRSKLPDISIPNHLSLTDYVFQKFSG
BniB003144-TA          1  .MALRQQETLSLAKKTDQEP...........SQDIIFRSKLPDISIPNHLPLIEYIFHRFSG
BniB003145-TA          1  .MALPQQETLSLTKKHSQD...............FIFRSKLPDISIPNHLSLTDYVFQKFSG
BniB003147-TA          1  .MEFRQQEALSLTKKTDQDP...........SQDFIFRSKLPDISIPNHLPLTDYIFQKFSG
BniB046377-TA          1  MAPQE....DAMQKQSSDN.............SDVIFRSKLPDIYIPNHLPLHDYIFQNISE
BniB049482-TA          1  MITATLQEPHEIHKPVDTTTT..SPTTDAPPTPPRIFRSKLPDIDIPNHLPLHTYCFEKLPS
Bol026623              1  MVLQQQQTLFVTKKTDQELP..LKTDPEPSR.EFIFRSKLPDISIPNHLPLTDYVFQKFSG
Bol038387              1  MALLRQQETLSLAK..NQEP...........SQDFIFRSKLPDISIPNHLSLTDYLFQKFSG
Bol038389              1  .MAFRQQETLSLTKKTDQD...............FIFRSKLPDISIPNHLPLAEYVFQKFSG
Bol012584              1  MITATLQEP.QIHQPVDTTT....PPADAPPTPPRIFRSKLPDIDIPNHLPLHNYCFQKLSS
Bol031583              1  MAPQE....DAMQKQSSNK.............SDVIFRSKLPDIYIPNHLPLHDYIFQNISE
```

```
                                          .         .         .         .         .
AT1G51680.1            51 FATKP......CLINGPTGHVYTYSDVHVISRQIAANFHKLGVNQNDVVMLLPNCPEFVLS
AT3G21240.1            49 FAAKP......CLINGPTGEVYTYADVHVTSRKLAAGLHNLGVKQHDVVMILLPNSPEVVLT
AT1G65060.1            61 VSDKP......CLIVGSTGKSYTYGETHLICRRVASGLYKLGIRKGDVIMILLQNSAEFVFS
AT3G21230.1            55 DGDGD..SSTTCIIDGATGRILTYADVQTNMRRIAAGIHRLGIRHGDVVMLLLPNSPEFALS
BnaA03T0366500ZS       60 DGDGD..STATCLIDGATGRIFTYGDVQISLRRIAAGIHRLGIRQRDTVMLLLPNSPEFALS
BnaA03T0366600ZS       51 NGDGD..STATCLIDSATGRIFTYADVQINSQRVAVSIHRLGIRQRDTVMLLLPNSPEFAFS
BnaA05T0171400ZS       47 FASKP......CLINGPTGHVYTYSDVHVASRRIAAGFQKLGVNQNDVVMILLSNCPEFVLS
BnaA05T0345400ZS       46 YAAKP......CLINGPTGEVYTYADVHATSRKLSAGLRKLGVGQHDVVMILLPNSPEFVFT
BnaA05T0345500ZS       50 DGGGD..STATCLIDGATGRIFTYADVQITLRSIAAGLYRLGIRHGDTVMLLLPNSPEFSLS
BnaA05T0345800ZS       47 DGDGD..STTTCLIDSATGRTFTYADVQITLQRIAAGLHRLGIRHGDTVMLLLPNSPEFALS
BnaA07T0281800ZS       58 VSDKP......CLIVGSTGKSYTYEETHLICRRVAAGLHKMGIRKGDVIMILLQNSAEFVFS
BnaC03T0447200ZS       60 DGDGD..STATCLINGATGRIFTYGDVQISLRRIAAGIHRLGIRQRDTVMLLLPNSPEFALS
BnaC05T0372300ZS       50 DGGGD..STATCLIDGATGRIFTYADVQITLRSIAAGLYRLGIRHGDTVMLLLPNSPEFALS
BnaC05T0372600ZS       47 DGDGD..STTTCLIDSATGRTFTYANVQITLRRIAAGLHRLGIRHGDTVMLLLPNSPEFALS
BnaC06T0113600ZS       46 FASKP......CLINGPTGHVYTYSEVHVASRRIAAGFQKLGVNQNDVVMILLSNCPEFVLS
BnaC06T0322800ZS       58 VSDKP......CLIVGSTGKNYTYGETHLICRRVAAGLHKMGIRKGDVIMILLQNSAEFVFS
BjuVA03G41320          60 DGDGD..STATCLIDGATGRIFTYGDVQISLRRIAAGIHRLGIRQRDTVMLLLPNSPEFALS
BjuVA03G41330          51 NGDGD..STATCLIDSATGRIFTYADVQINSQRVAVSIHRLGIRQRDTVMLLLPNSPEFAFS
BjuVA05G19140          47 FASKP......CLINGPTGHVYTYSDVHVASRRIAAGFQNLGVNQNDVVMILLSNCPEFVLS
BjuVA05G27610          46 YAAKP......CLINGPTGDVHTYADVHATSRKLSAGLRKLGVGQHDVVMILLPNSPEFVFT
BjuVA05G27640          50 DGGGD..STATCLIDGGTGRIFTYADVQITLRSIAAGLYRLGIRHGDTVMLLLPNSPEFAHS
BjuVA05G27670          47 GGDGD..STTTCLIDSATGRTFTYADVQITLQRIAAGLHRLGIRHGDTVMLLLPNSPEFALS
BjuVA07G32970          58 VSDKP......CLIVGSTGKSYTYGETHLICRRVAAGLHKMGIRKGDVIMILLQNSAEFVFS
BjuVB01G33150          51 NGDGG..STTTCFIDGVTGRIFTYADVQITLRRIAAGIYRLGIRHSDTVMLLLPNSPEFALS
BjuVB01G33160          47 DGDGD..STTTCLVDSATGHIFTYADVQITSRRVAAGLHRLGIRHGDTVMLLLPNSPEFALS
BjuVB01G33250          51 HGDGD..STTTCLIDGVTGRTFTYADVQITLRRIAAGIYGLGIRHGDTVMLLLPNSPEFALS
BjuVB03G38620          60 VSDKP......CLIVGSTGKSYTYGETHLTCRRVAAGLHKMGIRKGDVIMILLQNSAEFVFS
BjuVB06G27310          46 FASKP......CLINGPTGHVYTYSDVHVASRRIAAGFQKLGVNRNDVVMILLSNCPEFVLS
BjuVB07G27300          61 DGGGD..STATCLIDGANGRIFTYGDVQLSLRRIAAGIHRLGIRQRDTVMLLLPNSPEFALC
BcaB02g11270           46 FASKP......CLINGPTGHVYTYSDVHVASRRIAAGFQKLGVNRNDVVMILLSNCPEFVLS
BcaB04g19154           61 DGGGD..STATCLIDGANGRIFTYGDVQLSLRRIAAGIHRLGIRQRDTVMLLLPNSPEFALC
BcaB06g26203           51 HGDGD..STTTCLIDGVTGRTFTYADVQITLRRIAAGIYGLGIRHGDTVMLLLPNSPEFALS
BcaB06g26209           21 DGDGD..STTTCLVDSATGHIFTYADVQITSRRVAAGLHRLGIRHGDTVMLLLPNSPEFALS
BcaB06g26210           51 NGDGG..STTTCFIDGVTGRIFTYADVQITLRRIAAGIYRLGIRHSDTVMLLLPNSPEFALS
BcaC01g03516           60 DGDGH..STATCLINGATGRIFTYGDVQISLRRIAAGIHRLGIRQRDTVMLLLPNSPEFALS
BcaC05g28268           47 DGDGD..STTTCLIDSATGRTFTYANVQITLRRIAAGLHRLGIRHGDTVMLLLPNSPEFALS
BcaC05g28272           50 DGGGD..STATCLIDGATGRIFTYADVQITLRSIAAGLYRLGIRHGDTVMLLLPNSPEFALS
BcaC08g43929           46 FASKP......CLINGPTGHVYTYSEVHVASRRIAAGFQKLGVNQNDVVMILLSNCPEFVLS
BcaNung05537           61 VSDKP......CLIVGSTGKSYTYGETHLTCRRVAAGLHRLGIRKGDVIMILLQNSAEFVFS
BraA03g040280.4.1C.1   60 DGDGDGDSTATCLIDGATGRIFTYGDVQISLRRIAAGIHRLGIRQRDTVMLLLPNSPEFALS
BraA03g040290.4.1C.1   51 NGDGD..STATCLIDSATGRIFTYADVQINSQRVAVSIHRLGIRQRDTVMLLLPNSPEFAFS
BraA05g018900.4.1C.1   47 FASKP......CLINGPTGHVYTYSDVHVASRRIAAGFQKLGVNQNDVVMILLSNCPEFVLS
BraA05g027970.4.1C.3   46 YAAKP......CLINGPTGEVYTYADVHATSRKLSAGLRKLGVGQHDVVMILLPNSPEFVFT
BraA05g027990.4.1C.1   50 DGGGD..STATCLIDGATGRIFTYADVQITLRSIAAGLYRLGIRHGDTVMLLLPNSPEFSLS
BraA05g028020.4.1C.3   47 GGDGD..STTTCLIDSATGRTFTYADVQITLQRIAAGLHRLGIRHGDTVMLLLPNSPEFALS
BraA07g032760.4.1C.1   58 VSDKP......CLIVGSTGKSYTYDETHLICRRVAAGLHKMGIRKGDVIMILLQNSAEFVFS
BniB021049-PA          18 SRDSP..ATAT...............AIPPPHVSSTVP...................PAFALS
BniB021051-TA          61 DGGGD..STATCLIDGANGRIFTYGDVQLSLRRIAAGIHRLGIRQRDTVMLLLPNSPEFALC
BniB003144-TA          51 NGDGG..STTTCFIDGVTGRIFTYADVQITLRRIAAGIYRLGIRHSDTVMLLLPNSPEFALS
BniB003145-TA          47 DGDGD..STTTCLVDSATGHIFTYADVQITSRRVAAGLHRLGIRHGDTVMLLLPNSPEFALS
BniB003147-TA          51 HGDGD..STTTCLIDGVTGRTFTYADVQITLRRIAAGIYGLGIRHGDTVMLLLPNSPEFALS
BniB046377-TA          46 FASKP......CLINGPTGHVYTYSDVHVASRRIAAGFQKLGVNRNDVVMILLSNCPEFVLS
BniB049482-TA          61 VSDKP......CLIVGSTGKSYTYGETHLTCRRVAAGLHRLGIRKGDVIMILLQNSAEFVFS
Bol026623              60 DGDGD..STATCLINGATGRIFTYGDVQISLRRIAAGIHRLGIRQRDTVMLLLPNSPEFALS
Bol038387              50 DGGGD..STATCLIDGATGRIFTYADVQITLRSIAAGLYRLGIRHGDTVMLLLPNSPEFALS
Bol038389              47 DGDGD..STTTCLIDSATGRTFTYANVQITLRRIAAGLHRLGIRHGDTVMLLLPNSPEFALS
Bol012584              58 VSDKP......CLIVGSTGKNYTYGETHLICRRVAAGLHKMGIRKGDVIMILLQNSAEFVFS
Bol031583              46 FASKP......CLINGPTGHVYTYSEVHVASRRIAAGFQKLGVNQNDVVMILLSNCPEFVLS
```

```
AT1G51680.1              107  FLAASFRGATATAANPFFTPAEIAKQAKASNTKLIITEARYVDKIKPLQNDDGVVIVCIDDN
AT3G21240.1              105  FLAASFIGAITTSANPFFTPAEISKQAKASAAKLIVTQSRYVDKIKNLQND.GVLIVTTDS.
AT1G65060.1              117  FMGASMIGAVSTTANPFYTSQELYKQLKSSGAKLIITHSQYVDKLKNLG......ENLTLIT
AT3G21230.1              115  FLAVAYLGAVSTTANPFYTQPEIAKQAKASAAKMIITKKCLVDKLTNLKND.GVLIVCLDDD
BnaA03T0366500ZS         120  FLAVAYLGAVSTTANPFYTESEIEKQAKASATKMIITKSCYVDKLTNMKND.SVLIVCVEDE
BnaA03T0366600ZS         111  FLAVAYLGAVTTTANPLYTQAEIARQANASAAKMIITKQCYVDKLINLQND.GVLIVCVDDE
BnaA05T0171400ZS         103  FLAASFRGATATAANPFFTPAEIAKQAKASNSKLIVTESRYVDKIKDLQND.GVIIVCTDE.
BnaA05T0345400ZS         102  FLAASFLGAVTTAANPFFTPAEISKQAKASAAKLIVTQSRYVDKVRDL....GLLIICTDS.
BnaA05T0345500ZS         110  FLAAAYLGAISTPANPLFTQPEIAKQAQASGAKMIITKPCYVDKLKNLEN...VLIVCVP..
BnaA05T0345800ZS         107  FLAVVHLGAVSTSANPLFTQTEIAKQAKASAAKMIITKSCYVHKLTNLRQL.GVVIVCVDDK
BnaA07T0281800ZS         114  FMGASMIGAVSTTANPFYTSQEIHKQVKSSGAKLIITHSHYVDKLRNLDGETRIGEDLTVIT
BnaC03T0447200ZS         120  FLAVAYLGAVSTTANPFYTKSEIEKQAKASATKMIITKPCYVDKLTNLKND.GVLIVCVEDE
BnaC05T0372300ZS         110  LLGAAYLGAISTPANPLFTQPEIAKQAQASGAKMIITKPCYVDKLTNLEN...VLIVCVHDG
BnaC05T0372600ZS         107  FLAAVYLGAVSTSANPLFTQTEIAKQAKASAAKMIITKPCYVHKLTNLQQL.GAVIVCVDDR
BnaC06T0113600ZS         102  FLAASFRGATATAANPFFTPAEIAKQAKASNSKLIVTESRYVDKIKDLQND.GVIIVCTDE.
BnaC06T0322800ZS         114  FMGASMIGAVSTTANPFYTSQEIHKQVKSSGAKLIITHSHYVDKLRNLDGETRIGEDLTVIT
BjuVA03G41320            120  FLAVAYLGAVSTTANPFYTESEIEKQAKASATKMIITKSCYVDKLTNMKND.SVLIVCVEDE
BjuVA03G41330            111  FLAVAYLGAVTTTANPLYTQAEIARQANASNAKMIITKQCYVDKLINFQND.GVLIVCVDDE
BjuVA05G19140            103  FLAASFRGATATAANPFFTPEEIAKQAKASNSKLIVTESRYVDKIKDLQND.GVIIVCTDE.
BjuVA05G27610            102  FLAASFLGAVTTAANPFFTPAEISKQAKASAAKLIVTQSRYVDKVRDL....GLLIICTDS.
BjuVA05G27640            110  FLAAAYLGAISTPANPLFTQPEIAKQAQASGAKMIITKPCYVDKLKNLEN...VLIVCVP..
BjuVA05G27670            107  FLAVVHLGAVSTSANPLFTQTEIAKQAKASAAKMIITKSCYVHKLTNLRQL.GVVIVCVDDR
BjuVA07G32970            114  FMGASMIGAVSTTANPFYTSQEIHKQVKSSGAKLIITHSHYVDKLRNLDGETRIGEDLTVIT
BjuVB01G33150            111  FLAAAYLGAITTPANPLFTPPEIAKQAKASGTKMIITKPCYVDKLTDFQN...VLIVCID..
BjuVB01G33160            107  FLAVAYLGAISTPANPLLTESEIAKQAKASATKIVITKRSYVDKLTNLQN...VLIVCMDDG
BjuVB01G33250            111  FLAVVDLGAVSTSANPLFTQTEIAKQAKGSAAKMIITKPCYVHKLTNLQRL.GVVIVCVDDG
BjuVB03G38620            116  FMGASMIGAVSTTANPFYTSQEIHKQVRSSGAKLIVTHSHYVDKLRNLDGETKIGEDLTVIT
BjuVB06G27310            102  FLAASFRGATATAANPFFTPAEIAKQAKASNSKLIVTESRYVDKIKDLQND.GVVIVCTDE.
BjuVB07G27300            121  FLAVAYLGAVSTTANPFYTQSEIAKQAKASATKMIITKPCYVDKLTNLKND.GISIVCVEDE
BcaB02g11270             102  FLAASFCGATATAANPFFTPAEIAKQAKASNSKLIVTESRYVDKIKDLQND.GVVIVCTDE.
BcaB04g19154             121  FLAVAYLGAVSTTANPFYTQSEIAKQAKASATKMIITKPCYVDKLTNLKND.GVLIVCVEDE
BcaB06g26203             111  FLAVVDLGAVSTSANPLFTQTEIAKQAKGSAAKMIITKPCYVHKLTNLQRL.GVVIVCVDDG
BcaB06g26209              81  FLAVAYLGAISTPANPLLTESEIAKQAKASATKIVITKRSYVDKLTNLQN...VLIVCMDDG
BcaB06g26210             111  FLAAAYLGAITTPANPLFTPTEIAKQAKASGTKMIITKPCYVDKLTDLQN...VLIVCID..
BcaC01g03516             120  FLAVAYLGAVSTTANPFYTESEIEKQAKASATKMIITKSCYVDKLTNMKND.SVLIVCVEDE
BcaC05g28268             107  FLAAVYLGAVSTSANPLFTQTEIAKQAKASAAKMIITKPCYVHKLTNLQQL.GAVIVCVDDR
BcaC05g28272             110  LLGAAYLGAISTPANPLFTQPEIAKQAQASGAKMIITKPCYVDKLTNLEN...VLIVCVHDG
BcaC08g43929             102  FLAASFRGATATAANPFFTPAEIAKQAKASNSKLIVTESRYVDKIKDLQND.GVIIVCTDE.
BcaNung05537             117  FMGASMIGAVSTTANPFYTSQEIHKQVRSSGAKLIVTHSHYVDKLRNLDGETKIGEDLAVIT
BraA03g040280.4.1C.1     122  FLAVAYLGAVSTTANPFYTESEIEKQAKASATKMIITKSCYVDKLTNMKND.GVLIVCVEDE
BraA03g040290.4.1C.1     111  FLAVAYLGAVTTTANPLYTQAEIARQANASNAKMIITKQCYVDKLINFQND.GVLIVCVDDE
BraA05g018900.4.1C.1     103  FLAASFRGATATAANPFFTPAEIAKQAKASNSKLIVTESRYVDKIKDLQND.GVIIVCTDE.
BraA05g027970.4.1C.3     102  FLAASFLGAVTTAANPFFTPAEISKQAKASAAKLIVTQSRYVDKVRDL....GLLIICTDS.
BraA05g027990.4.1C.1     110  FLAAAYLGAISTPANPLFTQPEIAKQAQASGAKMIITKPCYVDKLKNLEN...VLIVCVP..
BraA05g028020.4.1C.3     107  FLAVVYLGAVSTSANPLFTQTEIAKQAKASAAKMIITKSCYVHKLTNLRQL.GVVIVCVDDR
BraA07g032760.4.1C.1     114  FMGASMIGAVSTTANPFYTSQEIHKQVKSSGAKLIITHSHYVDKLRNLDGETRIGEDLTVIT
BniB021049-PA             45  FLALAYLGAVTTTANPLSTQAEIARQAKASAAKMIITRQCHADRLKNLRND.RVLIVCVDDE
BniB021051-TA            121  FLAVAYLGAVSTTANPFYTQSEIAKQAKASATKMIITKPCYVDKLTNLKND.GILIVCVEDE
BniB003144-TA            111  FLAAAYLGAITTPANPLFTPPEIAKQAKASGTKMIITKPCYVDKLTDFQN...VLIVCID..
BniB003145-TA            107  FLAVAYLGAISTPANPLLTESEIAKQAKASATKILITKRSYVDKLTNLQN...VLIVCMDDG
BniB003147-TA            111  FLAVVDLGAVSTSANPLFTQTEIAKQAKGSAAKMIITKPCYVHKLTNLQRL.GVVIVCVDDG
BniB046377-TA            102  FLAASFRGATATAANPFFTPAEIAKQAKASNSKLIVTESRYVDKIKDLQND.GVVIVCTDE.
BniB049482-TA            117  FMGASMIGAVSTTANPFYTSQEIHKQVRSSGAKLIVTHSHYVDKLRNLDGETKIGEDLAVIT
Bol026623                120  FLAVAYLGAVSTTANPFYTKSEIEKQAKASATKMIITKPCYVDKLTNLKND.GVLIVCVEDE
Bol038387                110  LLGAAYLGAISTPANPLFTQPEIAKQAQASGAKMIITKPCYVDKLTNLEN...VLIVCVHDG
Bol038389                107  FLAAVYLGAVSTSANPLFTQTEIAKQAKASAAKMIITKPCYVHKLTNLQQL.GAVIVCVDDR
Bol012584                114  FMGASMIGAVSTTANPFYTSQEIHKQVKSSGAKLIITHSHYVDKLRNLDGETRIGEDLTVIT
Bol031583                102  FLAASFRGATATAANPFFTPAEIAKQAKASNSKLIVTESRYVDKIKDLQND.GVIIVCTDE.
```

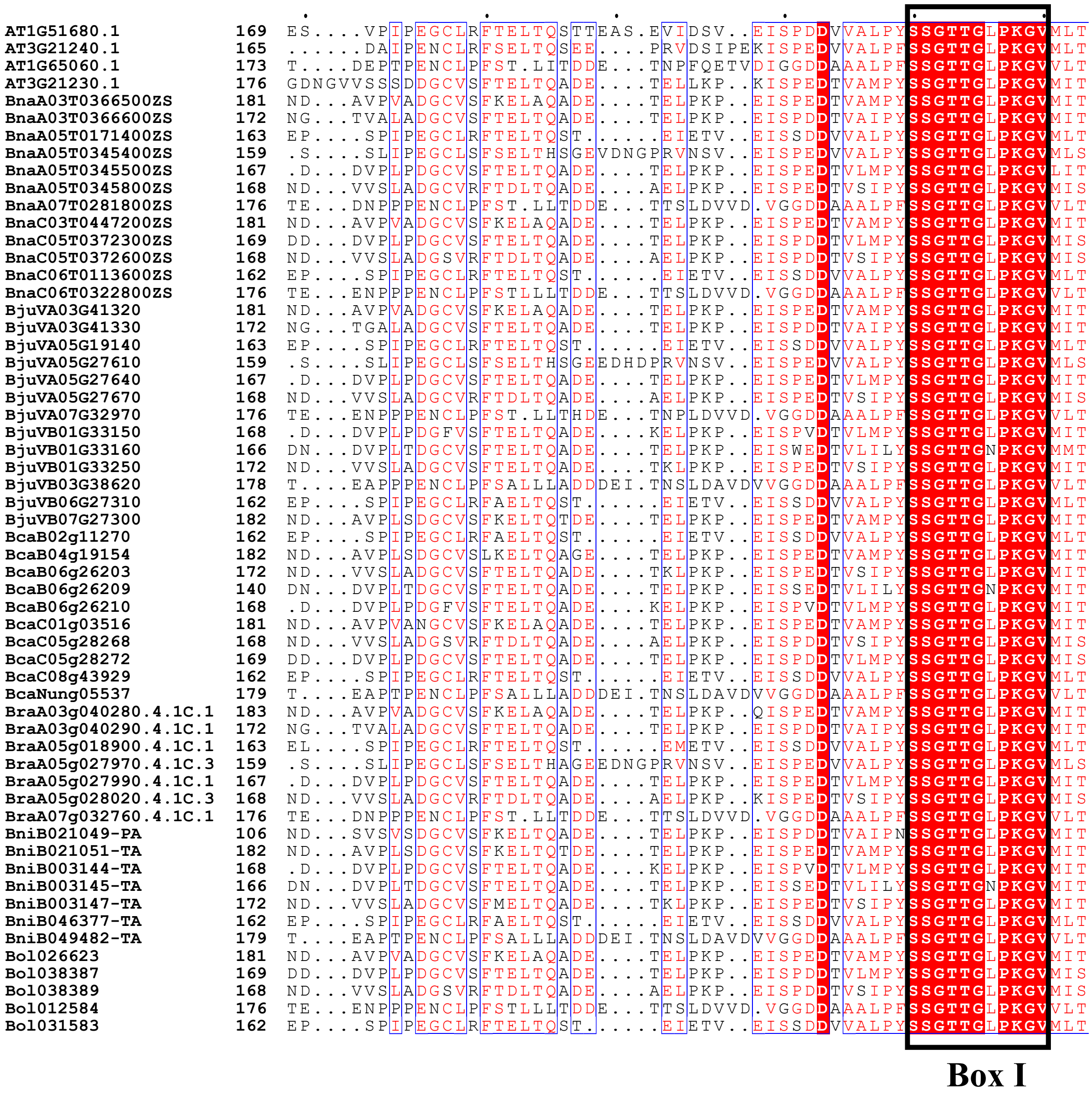
AT1G51680.1 169 ES....VPIPEGCLRFTELTQSTTEAS.EVIDSV..EISPDDVVALPYSSGTTGLPKGVMLT
AT3G21240.1 165 ......DAIPENCLRFSELTQSEE....PRVDSIPEKISPEDVVALPFSSGTTGLPKGVMLT
AT1G65060.1 173 T....DEPTPENCLPFST.LITDDE...TNPFQETVDIGGDDAAALPFSSGTTGLPKGVVLT
AT3G21230.1 176 GDNGVVSSSDDGCVSFTELTQADE....TELLKP..KISPEDTVAMPYSSGTTGLPKGVMIT
BnaA03T0366500ZS 181 ND...AVPVADGCVSFKELAQADE....TELPKP..EISPEDTVAMPYSSGTTGLPKGVMIT
BnaA03T0366600ZS 172 NG...TVALADGCVSFTELTQADE....TELPKP..EISPDDTVAIPYSSGTTGLPKGVMIT
BnaA05T0171400ZS 163 EP....SPIPEGCLRFTELTQST......EIETV..EISSDDVVALPYSSGTTGLPKGVMLT
BnaA05T0345400ZS 159 .S....SLIPEGCLSFSELTHSGEVDNGPRVNSV..EISPEDVVALPYSSGTTGLPKGVMLS
BnaA05T0345500ZS 167 .D...DVPLPDGCVSFTELTQADE....TELPKP..EISPEDTVLMPYSSGTTGLPKGVLIT
BnaA05T0345800ZS 168 ND...VVSLADGCVRFTDLTQADE....AELPKP..EISPEDTVSIPYSSGTTGLPKGVMIS
BnaA07T0281800ZS 176 TE...DNPPPENCLPFST.LLTDDE...TTSLDVVD.VGGDDAAALPFSSGTTGLPKGVVLT
BnaC03T0447200ZS 181 ND...AVPVADGCVSFKELAQADE....TELPKP..EISPEDTVAMPYSSGTTGLPKGVMIT
BnaC05T0372300ZS 169 DD...DVPLPDGCVSFTELTQADE....TELPKP..EISPDDTVLMPYSSGTTGLPKGVMIS
BnaC05T0372600ZS 168 ND...VVSLADGSVRFTDLTQADE....AELPKP..EISPDDTVSIPYSSGTTGLPKGVMIS
BnaC06T0113600ZS 162 EP....SPIPEGCLRFTELTQST......EIETV..EISSDDVVALPYSSGTTGLPKGVMLT
BnaC06T0322800ZS 176 TE...ENPPPENCLPFSTLLLTDDE...TTSLDVVD.VGGDDAAALPFSSGTTGLPKGVVLT
BjuVA03G41320 181 ND...AVPVADGCVSFKELAQADE....TELPKP..EISPEDTVAMPYSSGTTGLPKGVMIT
BjuVA03G41330 172 NG...TGALADGCVSFTELTQADE....TELPKP..EISPDDTVAIPYSSGTTGLPKGVMIT
BjuVA05G19140 163 EP....SPIPEGCLRFTELTQST......EIETV..EISSDDVVALPYSSGTTGLPKGVMLT
BjuVA05G27610 159 .S....SLIPEGCLSFSELTHSGEEDHDPRVNSV..EISPEDVVALPYSSGTTGLPKGVMLS
BjuVA05G27640 167 .D...DVPLPDGCVSFTELTQADE....TELPKP..EISPEDTVLMPYSSGTTGLPKGVMIT
BjuVA05G27670 168 ND...VVSLADGCVRFTDLTQADE....AELPKP..EISPEDTVSIPYSSGTTGLPKGVMIS
BjuVA07G32970 176 TE...ENPPPENCLPFST.LLTHDE...TNPLDVVD.VGGDDAAALPFSSGTTGLPKGVVLT
BjuVB01G33150 168 .D...DVPLPDGFVSFTELTQADE....KELPKP..EISPVDTVLMPYSSGTTGLPKGVMIT
BjuVB01G33160 166 DN...DVPLTDGCVSFTELTQADE....TELPKP..EISWEDTVLILYSSGTTGNPKGVMMT
BjuVB01G33250 172 ND...VVSLADGCVSFTELTQADE....TKLPKP..EISPEDTVSIPYSSGTTGLPKGVMIT
BjuVB03G38620 178 T....EAPPPENCLPFSALLLADDDEI.TNSLDAVDVVGGDDAAALPFSSGTTGLPKGVVLT
BjuVB06G27310 162 EP....SPIPEGCLRFAELTQST......EIETV..EISSDDVVALPYSSGTTGLPKGVMLT
BjuVB07G27300 182 ND...AVPLSDGCVSFKELTQTDE....TELPKP..EISPEDTVAMPYSSGTTGLPKGVMIT
BcaB02g11270 162 EP....SPIPEGCLRFAELTQST......EIETV..EISSDDVVALPYSSGTTGLPKGVMLT
BcaB04g19154 182 ND...AVPLSDGCVSLKELTQAGE....TELPKP..EISPEDTVAMPYSSGTTGLPKGVMIT
BcaB06g26203 172 ND...VVSLADGCVSFTELTQADE....TKLPKP..EISPEDTVSIPYSSGTTGLPKGVMIT
BcaB06g26209 140 DN...DVPLTDGCVSFTELTQADE....TELPKP..EISSEDTVLILYSSGTTGNPKGVMIT
BcaB06g26210 168 .D...DVPLPDGFVSFTELTQADE....KELPKP..EISPVDTVLMPYSSGTTGLPKGVMIT
BcaC01g03516 181 ND...AVPVANGCVSFKELAQADE....TELPKP..EISPEDTVAMPYSSGTTGLPKGVMIT
BcaC05g28268 168 ND...VVSLADGSVRFTDLTQADE....AELPKP..EISPDDTVSIPYSSGTTGLPKGVMIS
BcaC05g28272 169 DD...DVPLPDGCVSFTELTQADE....TELPKP..EISPDDTVLMPYSSGTTGLPKGVMIS
BcaC08g43929 162 EP....SPIPEGCLRFTELTQST......EIETV..EISSDDVVALPYSSGTTGLPKGVMLT
BcaNung05537 179 T....EAPTPENCLPFSALLLADDDEI.TNSLDAVDVVGGDDAAALPFSSGTTGLPKGVVLT
BraA03g040280.4.1C.1 183 ND...AVPVADGCVSFKELAQADE....TELPKP..QISPEDTVAMPYSSGTTGLPKGVMIT
BraA03g040290.4.1C.1 172 NG...TVALADGCVSFTELTQADE....TELPKP..EISPDDTVAIPYSSGTTGLPKGVMIT
BraA05g018900.4.1C.1 163 EL....SPIPEGCLRFTELTQST......EMETV..EISSDDVVALPYSSGTTGLPKGVMLT
BraA05g027970.4.1C.3 159 .S....SLIPEGCLSFSELTHAGEEDNGPRVNSV..EISPEDVVALPYSSGTTGLPKGVMLS
BraA05g027990.4.1C.1 167 .D...DVPLPDGCVSFTELTQADE....TELPKP..EISPEDTVLMPYSSGTTGLPKGVMIT
BraA05g028020.4.1C.3 168 ND...VVSLADGCVRFTDLTQADE....AELPKP..KISPEDTVSIPYSSGTTGLPKGVMIS
BraA07g032760.4.1C.1 176 TE...DNPPPENCLPFST.LLTDDE...TTSLDVVD.VGGDDAAALPFSSGTTGLPKGVVLT
BniB021049-PA 106 ND...SVSVSDGCVSFKELTQADE....TELPKP..EISPDDTVAIPNSSGTTGLPKGVMIT
BniB021051-TA 182 ND...AVPLSDGCVSFKELTQTDE....TELPKP..EISPEDTVAMPYSSGTTGLPKGVMIT
BniB003144-TA 168 .D...DVPLPDGCVSFTELTQADE....KELPKP..EISPVDTVLMPYSSGTTGLPKGVMIT
BniB003145-TA 166 DN...DVPLTDGCVSFTELTQADE....TELPKP..EISSEDTVLILYSSGTTGNPKGVMIT
BniB003147-TA 172 ND...VVSLADGCVSFMELTQADE....TKLPKP..EISPEDTVSIPYSSGTTGLPKGVMIT
BniB046377-TA 162 EP....SPIPEGCLRFAELTQST......EIETV..EISSDDVVALPYSSGTTGLPKGVMLT
BniB049482-TA 179 T....EAPTPENCLPFSALLLADDDEI.TNSLDAVDVVGGDDAAALPFSSGTTGLPKGVVLT
Bol026623 181 ND...AVPVADGCVSFKELAQADE....TELPKP..EISPEDTVAMPYSSGTTGLPKGVMIT
Bol038387 169 DD...DVPLPDGCVSFTELTQADE....TELPKP..EISPDDTVLMPYSSGTTGLPKGVMIS
Bol038389 168 ND...VVSLADGSVRFTDLTQADE....AELPKP..EISPDDTVSIPYSSGTTGLPKGVMIS
Bol012584 176 TE...ENPPPENCLPFSTLLLTDDE...TTSLDVVD.VGGDDAAALPFSSGTTGLPKGVVLT
Bol031583 162 EP....SPIPEGCLRFTELTQST......EIETV..EISSDDVVALPYSSGTTGLPKGVMLT
Box I

```
AT1G51680.1              224 HKGLVTSVAQQVDGENPNLYFHSDDVILCVLPMFHIY....ALNSIMLCGLRVGAAILIMPK
AT3G21240.1              217 HKGLVTSVAQQVDGENPNLYFNRDDVILCVLPMFHIY....ALNSIMLCSLRVGATILIMPK
AT1G65060.1              227 HKSLITSVAQQVDGDNPNLYLKSNDVILCVLPLFHIY....SLNSVLLNSLRSGATVLLMHK
AT3G21230.1              232 HKGLVTSIAQKVDGENPNLNFTANDVILCFLPMFHIY....ALDALMLSAMRTGAALLIVPR
BnaA03T0366500ZS         234 HKGLVTSIAQKVDGENPNVNFTGDDVILCFLPMFHIY....ALDALMLSAMRTGAAILIVPR
BnaA03T0366600ZS         225 HKSLVTSIAQKVDGENPNLNFTGDDVIICFLPMFHTY....AFNALILSAMRTGAAVLIVPR
BnaA05T0171400ZS         213 HRGLVTSVAQQVDGDNPNLYFHSDDVILCVLPLFHIY....ALNSIMLCGLRVGASILIMPK
BnaA05T0345400ZS         214 HKGLVTSVAQQVDGENPNLYFNREDVILCVLPMFHIY....ALNSIMLCSLRVGATILIMPK
BnaA05T0345500ZS         219 HKGVVTSTAQKVDGENPHLNFTGDDVIICFLPMFHTY....AHNSLMLSAMRAGAAFLILPR
BnaA05T0345800ZS         221 HKGLVTSIAQKVDGENPNLNFTRDDVIICFLPMFHTF....THSSLMLSAMRTGAAFLILPR
BnaA07T0281800ZS         230 HKSLITSVAQQVDGDNPNLYLKPNDVVLCVLPLFHIY....SLNSVLLNSIRSGATVLLMHK
BnaC03T0447200ZS         234 HKGLVTSIAQKVDGENPNVNFTGDDVILCFLPMFHIY....ALDALMLSAMRTGAAILIVPR
BnaC05T0372300ZS         222 HKGVVASTAQKVDGENPHLNFTGDDVMICFLPMFHTY....AHNLLMLSAMRAGAAFLILPR
BnaC05T0372600ZS         221 HKGLVTSIAQKVDGENPNLNFTRDDVIICFLPMFHTF....THSSLMLSAMRTGAALLILPR
BnaC06T0113600ZS         212 HKGLVTSVAQQVDGDNPNLYFHSDDVILCVLPMFHIY....ALNSIMLCGLRVGASILIMPK
BnaC06T0322800ZS         231 HKSLITSVAQQVDGDNPNLYLKPNDVVLCVLPLFHIY....SLNSVLLNSIRSGATVLLMHK
BjuVA03G41320            234 HKGLVTSIAQKVDGENPNVNFTGDDVILCFLPMFHIY....ALDALMLSAMRTGAAILIVPR
BjuVA03G41330            225 HKSLVTSIAQKVDGENPNLNFTGDDVIICFLPMFHTY....AFNALILSAMRTGAAVLIVPR
BjuVA05G19140            213 HKGLVTSVAQQVDGDNPNLYFHSDDVILCVLPLFHIY....ALNSIMLCGLRVGASILIMPK
BjuVA05G27610            214 HKGLVTSVAQQVDGENPNLYFNKEDVILCVLPMFHIY....ALNSIMLCSLRVGATILIMPK
BjuVA05G27640            219 HKGVVTSTAQKVDGENPHLNFTGDDVIICFLPMFHTY....AHNSLMLSAMRAGAAFLILPR
BjuVA05G27670            221 HKGLVTSIAQKVDGENPNLNFTRDDVIICFLPMFHTF....THSSLMLSAMRTGAAFLILPR
BjuVA07G32970            230 HKSLITSVAQQVDGDNPNLYLKPNDVVLCVLPLFHIY....SLNSVLLNSIRSGATVLLMHK
BjuVB01G33150            220 HKGVVTSTAQKVDGENPHLNFTGDDVIICFLPMFHTY....THNSLMLSAMRVGAAFLILPM
BjuVB01G33160            219 HKGVVTSIAQKVDGENPNVNFTRDDVIIFFLPMFHLY....PLDALMLSAMRTGAAILIVPR
BjuVB01G33250            225 HKGLVTSVAQKVDGENPNLNFTGDDVIICFLPMFHTF....THSSLMLSAMRTGAAFLILPR
BjuVB03G38620            235 HRSLITSVAQQVDGDNPNLYLKSNDVVLCVLPLFHIY....SLNSVLLNSIRSGATVLLMHK
BjuVB06G27310            212 HKGLVTSVAQQVDGDNPNLYFHSDDVILCVLPMFHIY....ALNSIMLCGLRVGASILIMPK
BjuVB07G27300            235 HKGLVTSIAQKVDGENPNVNFTGDDVIICFLPMFHIY....ALDALMLSAMRTGAAILIVPR
BcaB02g11270             212 HKGLVTSVAQQVDGDNPNLYFHSDDVILCVLPMFHIY....ALNSIMLCGLRVGASILIMPK
BcaB04g19154             235 HKGLVTSIAQKVDGENPNLNFTGDDVIICFLPMFHIY....ALDALMLSAMRTGAAILIVPR
BcaB06g26203             225 HKGLVTSVAQKVDGENPNLNFTGDDVIICFLPMFHTF....THSSLMLSAMRTGAAFLILPR
BcaB06g26209             193 HKGVVTSIAQKVDGENPNVNFTRDDVIIFFLPMFHLY....PLDALMLSAMRTGAAILIVPR
BcaB06g26210             220 HKGVVTSTAQKVDGENPHLNFTGDDVIICFLPMFHTYTATYTHNSLMLSAMRVGAAFLILPM
BcaC01g03516             234 HKGLVTSIAQKVDGENPNVNFTGDDVILCFLPMFHIY....ALDALMLSAMRSGAAILIVPR
BcaC05g28268             221 HKGLVTSIAQKVDGENPNLNFTRDDVIICFLPMFHTF....THSSLMLSAMRTGAALLILPR
BcaC05g28272             222 HKGVVASTAQKVDGENPHLNFTGDDVMICFLPMFHTY....AHNLLMLSAMRAGAAFLILPR
BcaC08g43929             212 HKGLVTSVAQQVDGDNPNLYFHSDDVILCVLPMFHIY....ALNSIMLCGLRVGASILIMPK
BcaNung05537             236 HKSLITSVAQQVDGDNPNLYLKSNDVVLCVLPLFHIY....SLNSVLLNSIRSGATVLLMHK
BraA03g040280.4.1C.1     236 HKGLVTSIAQKVDGENPNVNFTGDDVILCFLPMFHIY....ALDALMLSAMRTGAAILIVPR
BraA03g040290.4.1C.1     225 HKSLVTSIAQKVEGENPNLNFTGDDVIICFLPMFHTY....AFNALILSAMRTGAAVLIVPR
BraA05g018900.4.1C.1     213 HKGLVTSVAQQVDGDNPNLYFHSDDVILCVLPLFHIY....ALNSIMLCGLRVGASILIMPK
BraA05g027970.4.1C.3     214 HKGLVTSVAQQVDGENPNLYFNREDVILCVLPMFHIY....ALNSIMLCSLRVGATILIMPK
BraA05g027990.4.1C.1     219 HKGVVTSTAQKVDGENPHLNFTGDDVIICFLPMFHTY....AHNSLMLSAMRAGAAFLILPR
BraA05g028020.4.1C.3     221 HKGLVTSIAQKVDGENPNLNFTRDDVIICFLPMFHTF....THSSLMLSAMRTGAAFLILPR
BraA07g032760.4.1C.1     230 HKSLITSVAQQVDGDNPNLYLKPNDVVLCVLPLFHIY....SLNSVLLNSIRSGATVLLMHK
BniB021049-PA            159 YKGLVTSIAQKVDGENPNLNFTVDDVIICFLPMFHTY....AFNALILSAMRTGAAILIVPR
BniB021051-TA            235 HKGLVTSIAQKVDGENPNVNFTGDDVIICFLPMFHIY....ALDALMLSAMRTGAAILIVPR
BniB003144-TA            220 HKGIVTSTAQKVDGENPHLNFTGDDVIICFLPMFHTY....THNSLMLSAMRVGAAFLILPM
BniB003145-TA            219 HKGVVTSIAQKVDGENPNVNFTRDDVIIFFLPMFHLY....PLDALMLSAMRTGAAILIVPR
BniB003147-TA            225 HKGLVTSVAQKVDGENPNLNFTGDDVIICFLPMFHTF....THSSLMLSAMRTGAAFLILPR
BniB046377-TA            212 HKGLVTSVAQQVDGDNPNLYFHSDDVILCVLPMFHIY....ALNSIMLCGLRVGASILIMPK
BniB049482-TA            236 HKSLITSVAQQVDGDNPNLYLKSNDVVLCVLPLFHIY....SLNSVLLNSIRSGATVLLMHK
Bol026623                234 HKGLVTSIAQKVDGENPNVNFTGDDVILCFLPMFHIY....ALDALMLSAMRTGAAILIVPR
Bol038387                222 HKGVVASTAQKVDGENPHLNFTGDDVMICFLPMFHTY....AHNLLMLSAMRAGAAFLILPR
Bol038389                221 HKGLVTSIAQKVDGENPNLNFTRDDVIICFLPMFHTF....THSSLMLSAMRTGAALLILPR
Bol012584                231 HKSLITSVAQQVDGDNPNLYLKPNDVVLCVLPLFHIY....SLNSVLLNSIRSGATVLLMHK
Bol031583                212 HKGLVTSVAQQVDGDNPNLYFHSDDVILCVLPMFHIY....ALNSIMLCGLRVGASILIMPK
```

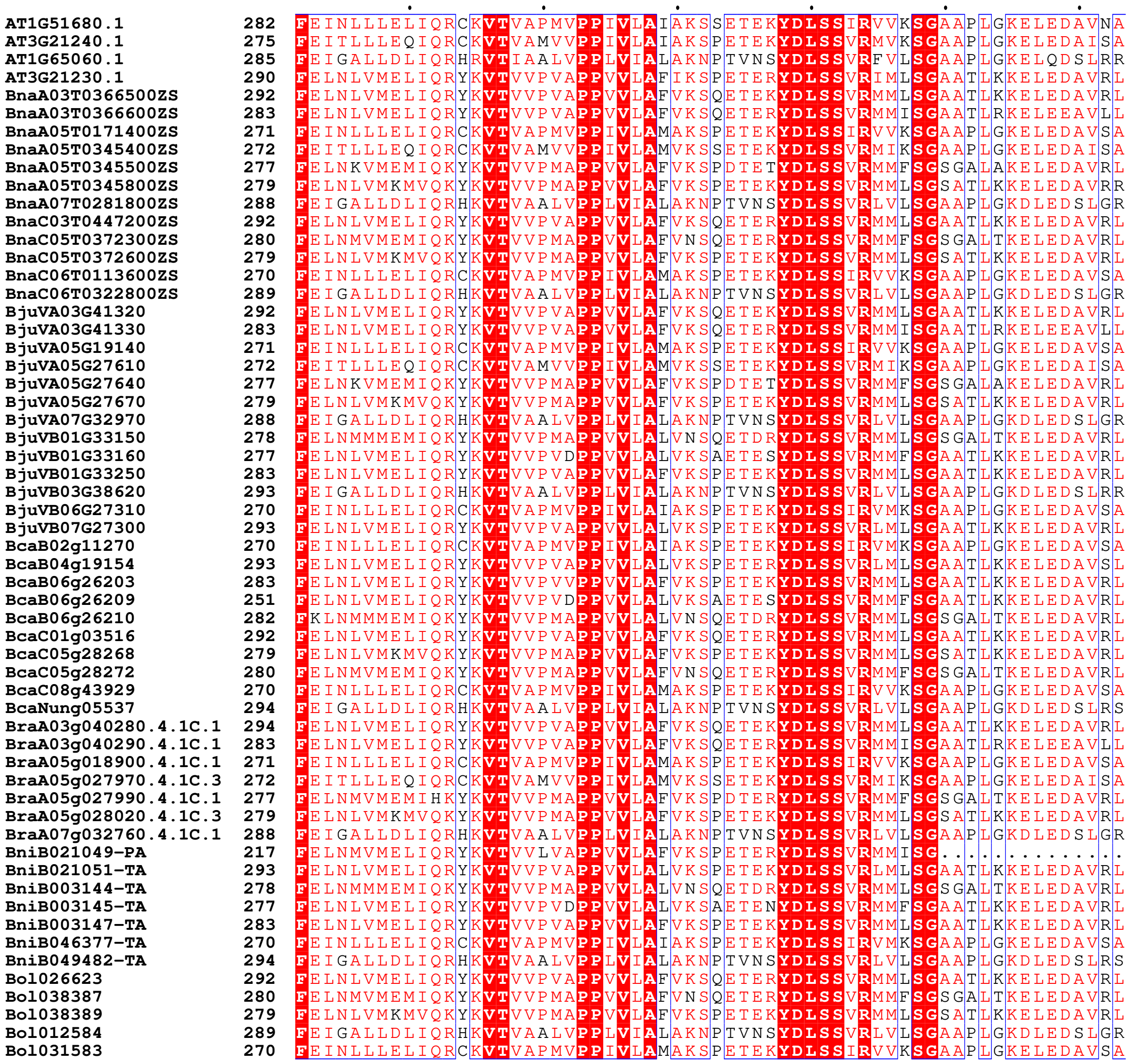

AT1G51680.1 282 FEINLLLELIQRCKVTVAPMVPPIVLAIAKSSETEKYDLSSIRVVKSGAAPLGKELEDAVNA
AT3G21240.1 275 FEITLLLEQIQRCKVTVAMVVPPIVLAIAKSPETEKYDLSSVRMVKSGAAPLGKELEDAISA
AT1G65060.1 285 FEIGALLDLIQRHRVTIAALVPPLVIALAKNPTVNSYDLSSVRFVLSGAAPLGKELQDSLRR
AT3G21230.1 290 FELNLVMELIQRYKVTVVPVAPPVVLAFIKSPETERYDLSSVRIMLSGAATLKKELEDAVRL
BnaA03T0366500ZS 292 FELNLVMELIQRYKVTVVPVAPPVVLAFVKSQETEKYDLSSVRMMLSGAATLKKELEDAVRL
BnaA03T0366600ZS 283 FELNLVMELIQRYKVTVVPVAPPVVLAFVKSQETERYDLSSVRMMISGAATLRKELEEAVLL
BnaA05T0171400ZS 271 FEINLLLELIQRCKVTVAPMVPPIVLAMAKSPETEKYDLSSIRVVKSGAAPLGKELEDAVSA
BnaA05T0345400ZS 272 FEITLLLEQIQRCKVTVAMVVPPIVLAMVKSSETEKYDLSSVRMIKSGAAPLGKELEDAISA
BnaA05T0345500ZS 277 FELNKVMEMIQKYKVTVVPMAPPVVLAFVKSPDTETYDLSSVRMMFSGSGALAKELEDAVRL
BnaA05T0345800ZS 279 FELNLVMKMVQKYKVTVVPMAPPVVLAFVKSPETEKYDLSSVRMMLSGSATLKKELEDAVRR
BnaA07T0281800ZS 288 FEIGALLDLIQRHKVTVAALVPPLVIALAKNPTVNSYDLSSVRLVLSGAAPLGKDLEDSLGR
BnaC03T0447200ZS 292 FELNLVMELIQRYKVTVVPVAPPVVLAFVKSQETERYDLSSVRMMLSGAATLKKELEDAVRL
BnaC05T0372300ZS 280 FELNMVMEMIQKYKVTVVPMAPPVVLAFVNSQETERYDLSSVRMMFSGSGALTKELEDAVRL
BnaC05T0372600ZS 279 FELNLVMKMVQKYKVTVVPMAPPVVLAFVKSPETEKYDLSSVRMMLSGSATLKKELEDAVRL
BnaC06T0113600ZS 270 FEINLLLELIQRCKVTVAPMVPPIVLAMAKSPETEKYDLSSIRVVKSGAAPLGKELEDAVSA
BnaC06T0322800ZS 289 FEIGALLDLIQRHKVTVAALVPPLVIALAKNPTVNSYDLSSVRLVLSGAAPLGKDLEDSLGR
BjuVA03G41320 292 FELNLVMELIQRYKVTVVPVAPPVVLAFVKSQETEKYDLSSVRMMLSGAATLKKELEDAVRL
BjuVA03G41330 283 FELNLVMELIQRYKVTVVPVAPPVVLAFVKSQETERYDLSSVRMMISGAATLRKELEEAVLL
BjuVA05G19140 271 FEINLLLELIQRCKVTVAPMVPPIVLAMAKSPETEKYDLSSIRVVKSGAAPLGKELEDAVSA
BjuVA05G27610 272 FEITLLLEQIQRCKVTVAMVVPPIVLAMVKSSETEKYDLSSVRMIKSGAAPLGKELEDAISA
BjuVA05G27640 277 FELNKVMEMIQKYKVTVVPMAPPVVLAFVKSPDTETYDLSSVRMMFSGSGALAKELEDAVRL
BjuVA05G27670 279 FELNLVMKMVQKYKVTVVPMAPPVVLAFVKSPETEKYDLSSVRMMLSGSATLKKELEDAVRL
BjuVA07G32970 288 FEIGALLDLIQRHKVTVAALVPPLVIALAKNPTVNSYDLSSVRLVLSGAAPLGKDLEDSLGR
BjuVB01G33150 278 FELNMMMEMIQKYKVTVVPMAPPVVLALVNSQETDRYDLSSVRMMLSGSGALTKELEDAVRL
BjuVB01G33160 277 FELNLVMELIQRYKVTVVPVDPPVVLALVKSAETESYDLSSVRMMFSGAATLKKELEDAVRL
BjuVB01G33250 283 FELNLVMELIQRYKVTVVPVAPPVVLAFVKSPETEKYDLSSVRMMLSGAATLKKELEDAVRL
BjuVB03G38620 293 FEIGALLDLIQRHKVTVAALVPPLVIALAKNPTVNSYDLSSVRLVLSGAAPLGKDLEDSLRR
BjuVB06G27310 270 FEINLLLELIQRCKVTVAPMVPPIVLAIAKSPETEKYDLSSIRVMKSGAAPLGKELEDAVSA
BjuVB07G27300 293 FELNLVMELIQRYKVTVVPVAPPVVLALVKSPETEKYDLSSVRLMLSGAATLKKELEDAVRL
BcaB02g11270 270 FEINLLLELIQRCKVTVAPMVPPIVLAIAKSPETEKYDLSSIRVMKSGAAPLGKELEDAVSA
BcaB04g19154 293 FELNLVMELIQRYKVTVVPVAPPVVLALVKSPETERYDLSSVRLMLSGAATLKKELEDAVSL
BcaB06g26203 283 FELNLVMELIQRYKVTVVPVVPPVVLAFVKSPETEKYDLSSVRMMLSGAATLKKELEDAVRL
BcaB06g26209 251 FELNLVMELIQRYKVTVVPVDPPVVLALVKSAETESYDLSSVRMMFSGAATLKKELEDAVRL
BcaB06g26210 282 FKLNMMMEMIQKYKVTVVPMAPPVVLALVNSQETDRYDLSSVRMMLSGSGALTKELEDAVRL
BcaC01g03516 292 FELNLVMELIQRYKVTVVPVAPPVVLAFVKSQETERYDLSSVRMMLSGAATLKKELEDAVRL
BcaC05g28268 279 FELNLVMKMVQKYKVTVVPMAPPVVLAFVKSPETEKYDLSSVRMMLSGSATLKKELEDAVRL
BcaC05g28272 280 FELNMVMEMIQKYKVTVVPMAPPVVLAFVNSQETERYDLSSVRMMFSGSGALTKELEDAVRL
BcaC08g43929 270 FEINLLLELIQRCKVTVAPMVPPIVLAMAKSPETEKYDLSSIRVVKSGAAPLGKELEDAVSA
BcaNung05537 294 FEIGALLDLIQRHKVTVAALVPPLVIALAKNPTVNSYDLSSVRLVLSGAAPLGKDLEDSLRS
BraA03g040280.4.1C.1 294 FELNLVMELIQRYKVTVVPVAPPVVLAFVKSQETEKYDLSSVRMMLSGAATLKKELEDAVRL
BraA03g040290.4.1C.1 283 FELNLVMELIQRYKVTVVPVAPPVVLAFVKSQETERYDLSSVRMMISGAATLRKELEEAVLL
BraA05g018900.4.1C.1 271 FEINLLLELIQRCKVTVAPMVPPIVLAMAKSPETEKYDLSSIRVVKSGAAPLGKELEDAVSA
BraA05g027970.4.1C.3 272 FEITLLLEQIQRCKVTVAMVVPPIVLAMVKSSETEKYDLSSVRMIKSGAAPLGKELEDAISA
BraA05g027990.4.1C.1 277 FELNMVMEMIHKYKVTVVPMAPPVVLAFVKSPDTERYDLSSVRMMFSGSGALTKELEDAVRL
BraA05g028020.4.1C.3 279 FELNLVMKMVQKYKVTVVPMAPPVVLAFVKSPETEKYDLSSVRMMLSGSATLKKELEDAVRL
BraA07g032760.4.1C.1 288 FEIGALLDLIQRHKVTVAALVPPLVIALAKNPTVNSYDLSSVRLVLSGAAPLGKDLEDSLGR
BniB021049-PA 217 FELNMVMELIQRYKVTVVLVAPPVVLAFVKSPETERYDLSSVRMMISG..............
BniB021051-TA 293 FELNLVMELIQRYKVTVVPVAPPVVLALVKSPETEKYDLSSVRLMLSGAATLKKELEDAVRL
BniB003144-TA 278 FELNMMMEMIQKYKVTVVPMAPPVVLALVNSQETDRYDLSSVRMMLSGSGALTKELEDAVRL
BniB003145-TA 277 FELNLVMELIQRYKVTVVPVDPPVVLALVKSAETENYDLSSVRMMFSGAATLKKELEDAVRL
BniB003147-TA 283 FELNLVMELIQRYKVTVVPVAPPVVLAFVKSPETEKYDLSSVRMMLSGAATLKKELEDAVRL
BniB046377-TA 270 FEINLLLELIQRCKVTVAPMVPPIVLAIAKSPETEKYDLSSIRVMKSGAAPLGKELEDAVSA
BniB049482-TA 294 FEIGALLDLIQRHKVTVAALVPPLVIALAKNPTVNSYDLSSVRLVLSGAAPLGKDLEDSLRS
Bol026623 292 FELNLVMELIQRYKVTVVPVAPPVVLAFVKSQETERYDLSSVRMMLSGAATLKKELEDAVRL
Bol038387 280 FELNMVMEMIQKYKVTVVPMAPPVVLAFVNSQETERYDLSSVRMMFSGSGALTKELEDAVRL
Bol038389 279 FELNLVMKMVQKYKVTVVPMAPPVVLAFVKSPETEKYDLSSVRMMLSGSATLKKELEDAVRL
Bol012584 289 FEIGALLDLIQRHKVTVAALVPPLVIALAKNPTVNSYDLSSVRLVLSGAAPLGKDLEDSLGR
Bol031583 270 FEINLLLELIQRCKVTVAPMVPPIVLAMAKSPETEKYDLSSIRVVKSGAAPLGKELEDAVSA

```
AT1G51680.1             344  KFPNAKLGQGYGMTEAGPVLAMSLGFAKEPFPVKSGACGTVVRNAEMKIVDPDTGDSLSRNQ
AT3G21240.1             337  KFPNAKLGQGYGMTEAGPVLAMSLGFAKEPFPVKSGACGTVVRNAEMKILDPDTGDSLPRNK
AT1G65060.1             347  RLPQAILGQGYGMTEAGPVLSMSLGFAKEPIPTKSGSCGTVVRNAELKVVHLETRLSLGYNQ
AT3G21230.1             352  KFPNAIFGQGYGMTESG.TVAKSLAFAKNPFKTKSGACGTVIRNAEMKVVDTETGISLPRNK
BnaA03T0366500ZS        354  KFPNAIFGQGYGMTESG.TVAKSLAFAKNPFKTKSGACGTVIRNAEMKVVDTITGVSLPRNK
BnaA03T0366600ZS        345  KFPNAIFGQGYGMTESA.TVAKSLSFAKNPFKTKSGACGTVIRNADMKVVDTITGVSLPRNK
BnaA05T0171400ZS        333  KFPNAKLGQGYGMTEAGPVLAMSLGFAKEPFPVKSGACGTVVRNAEMKIIDPDTGDSLSKNK
BnaA05T0345400ZS        334  KFPNARLGQGYGMTEAGPVLAMSLGFAKEPFPVKSGACGTVVRNAELKIADPDTGSSLPRNS
BnaA05T0345500ZS        339  KLPNAIFGQSYGMTEMG.TVAKSLAFAKNPFKIKSGSCGTVIRNAEMKVVDTITGVSLPHNK
BnaA05T0345800ZS        341  KLPNAIFGQSYGMTEAG.TVANSLAFAKHPFKTKSGSCGTVIRNAEMKVVDTITGASLPRNK
BnaA07T0281800ZS        350  RLPQAVLGQGYGMTEAGPVLSMSLGFAKEPTPSKSGSCGTVVRNAELKVVHLETRLSLGYNQ
BnaC03T0447200ZS        354  KFPNAIFGQGYGMTESG.TVAKSLAFAKNPFKTKSGACGTVIRNADMKVVDTITGVSLPRNK
BnaC05T0372300ZS        342  KLPNAIFGQSYGMTEMG.TVAKSLAFAKNPFKIKSGSCGTVIRNAEMKVVDTITGVSLPHNK
BnaC05T0372600ZS        341  KLPNAIFGQSYGMTEAG.TVANSLAFAKHPFKTKSGSCGTVIRNAEMKVVDTITGASLPRNK
BnaC06T0113600ZS        332  KFPNAKLGQGYGMTEAGPVLAMSLGFAKEPFPVKSGACGTVVRNAEMKIIDPDTGDSLSKNK
BnaC06T0322800ZS        351  RLPQAVLGQGYGMTEAGPVLSMSLGFAKEPTPSKSGSCGTVVRNAELKVVHLETRLSLGYNQ
BjuVA03G41320           354  KFPNAIFGQGYGMTESG.TVAKSLAFAKNPLKTKSGACGTVIRNAEMKVVDTITGVSLPRNK
BjuVA03G41330           345  KFPNAIFGQGYGMTGSA.TVAKSLSFAKNPFKTKSGACGTVIRNADMKVVDTITGVSLPRNK
BjuVA05G19140           333  KFPNAKLGQGYGMTEAGPVLAMSLGFAKEPFPVKSGACGTVVRNAEMKIIDPDTGDSLSKNK
BjuVA05G27610           334  KFPNARLGQGYGMTEAGPVLAMSLGFAKEPFPVKSGACGTVVRNAELKIADPDTGSSLPRNS
BjuVA05G27640           339  KLPNAIFGQSYGMTEMG.TVAKSLAFAKNPFKIKSGSCGTVIRNAEMKVVDTITGLSLPHNK
BjuVA05G27670           341  KLPNAIFGQSYGMTEAG.TVANSLAFAKHPFKTKSGSCGTVIRNAEMKVVDTITGASLPRNK
BjuVA07G32970           350  RLPQAVLGQGYGMTEAGPVLSMSLGFAKEPTPSKSGSCGTVVRNAELKVVHLETRLSLGYNQ
BjuVB01G33150           340  KLPSAIFGQSYGMTEMG.TVAKSLAFAKNPFKIKSGSCGTVIRNAEMKVVDTITGVSLPHNK
BjuVB01G33160           339  KFPNAILGQGYGMTESG.TVAKSLAFAKYPFQTKSGACGTVIRNAEMKVVEPITGVSLPRNK
BjuVB01G33250           345  KLPNAIFGQSYGMTEAG.TVANSLAFAKNPFKTKSGSCGTVIRNAEMKVVDTISGVSLPRNK
BjuVB03G38620           355  RLPQAVLGQGYGMTEAGPVLSMSLGFAKEPTPSKSGSCGTVVRNAELKVVHLETRLSLGYNQ
BjuVB06G27310           332  KFPNAKLGQGYGMTEAGPVLAMSLGFAKEPFPVKSGACGTVVRNAEMKIIDPDTGDSLSKNK
BjuVB07G27300           355  KFPNAIFGQGYGMTESG.TVAKSLAFAKKPFKTKSGACGTVIRNAEMKVVHTITGVSLPRNK
BcaB02g11270            332  KFPNAKLGQGYGMTEAGPVLAMSLGFAKEPFPVKSGACGTVVRNAEMKIIDPDTGDSLSKNK
BcaB04g19154            355  KFPNAIFGQGYGMTESG.TVAKSLAFAKNPFKTKSGACGTVIRNAEMKVVDTITGVSLPRNK
BcaB06g26203            345  KLPNAIFGQSYGMTEAG.TVANSLAFAKNPFKTKSGSCGTVIRNAEMKVVDTISGVSLPRNK
BcaB06g26209            313  KFPNAILGQGYGMTESG.TVAKSLAFAKYPFQTKSGACGTVIRNAEMKVVDTITGVSLPRKK
BcaB06g26210            344  KLPSAIFGQSYGMTEMG.TVAKSLAFAKNPFKIKSGSCGTVIRNAEMKVVDTITGVSLPHNK
BcaC01g03516            354  KFPNAIFGQGYGMTESG.TVAKSLAFAKNPFKTKSGACGTVIRNAEMKVVDTITGVSLPRNK
BcaC05g28268            341  KLPNAIFGQSYGMTEAG.TVANSLAFAKHPFKTKSGSCGTVIRNAEMKVVDTITGASLPRNK
BcaC05g28272            342  KLPNAIFGQSYGMTEMG.TVAKSLAFAKNPFKIKSGSCGTVIRNAEMKVVDTITGVSLPHNK
BcaC08g43929            332  KFPNAKLGQGYGMTEAGPVLAMSLGFAKEPFPVKSGACGTVVRNAEMKIIDPDTGDSLSKNK
BcaNung05537            356  RLPQAVLGQGYGMTEAGPVLSMSLGFAKEPTPSKSGSCGTVVRNAELKVVHLETRLSLGYNQ
BraA03g040280.4.1C.1    356  KFPNAIFGQGYGMTESG.TVAKSLAFAKNPFKTKSGACGTVIRNAEMKVVDTITGVSLPRNK
BraA03g040290.4.1C.1    345  KFPNAIFGQGYGMTESA.TVAKSLSFAKNPFKTKSGACGTVIRNADMKVVDTITGVSLPRNK
BraA05g018900.4.1C.1    333  KFPNAKLGQGYGMTEAGPVLAMSLGFAKEPFPVKSGACGTVVRNAEMKIIDPDTGDSLSKNK
BraA05g027970.4.1C.3    334  KFPNARLGQGYGMTEAGPVLAMSLGFAKEPFPVKSGACGTVVRNAELKIADPDTGSSLPRNS
BraA05g027990.4.1C.1    339  KLPNAIFGQSYGMTEMG.TVAKSLAFAKNPFKIKSGSCGTVIRNAEMKVVDTITGVSLPHNK
BraA05g028020.4.1C.3    341  KLPNAIFGQSYGMTEAG.TVANSLAFAKHPFKTKSGSCGTVIRNAEMKVVDTITGASLPRNK
BraA07g032760.4.1C.1    350  RLPQAVLGQGYGMTEAGPVLSMSLGFAKEPTPSKSGSCGTVVRNAELKVVHLETRLSLGYNQ
BniB021049-PA           265  ...................VPRSGRSSKKPCSSS............................
BniB021051-TA           355  KFPNAIFGQGYGMTESG.TVAKSLAFAKNPFKTKSGACGTVIRNAEMKVVHTITGVSLPRNK
BniB003144-TA           340  KLPSAIFGQSYGMTEMG.TVAKSLAFAKNPFKIKSGSCGTVIRNAEMKVVDTITGVSFSRNK
BniB003145-TA           339  KFPNAILGQGYGMTESA.TVAKSLAFAKYPFQTKSGACGTVIRNAEMKVVEPITGVSLPRNK
BniB003147-TA           345  KLPNAIFGQSYGMTEAG.TVANSLAFAKNPFKTKSGSCGTVIRNAEMKVVDTISGVSLPRNK
BniB046377-TA           332  KFPNAKLGQGYGMTEAGPVLAMSLGFAKEPFPVKSGACGTVVRNAEMKIIDPDTGDSLSKSK
BniB049482-TA           356  RLPQAVLGQGYGMTEAGPVLSMSLGFAKEPTPSKSGSCGTVVRNAELKVVHLETRLSLGYNQ
Bol026623               354  KFPNAIFGQGYGMTESG.TVAKSLAFAKNPFKTKSGACGTVIRNADMKVVDTITGVSLPRNK
Bol038387               342  KLPNAIFGQSYGMTEMG.TVAKSLAFAKNPFKIKSGSCGTVIRNAEMKVVDTITGVSLPHNK
Bol038389               341  KLPNAIFGQSYGMTEAG.TVANSLAFAKHPFKTKSGSCGTVIRNAEMKVVDTITGASLPRNK
Bol012584               351  RLPQAVLGQGYGMTEAGPVLSMSLGFAKEPTPSKSGSCGTVVRNAELKVVHLETRLSLGYNQ
Bol031583               332  KFPNAKLGQGYGMTEAGPVLAMSLGFAKEPFPVKSGACGTVVRNAEMKIIDPDTGDSLSKNK
```

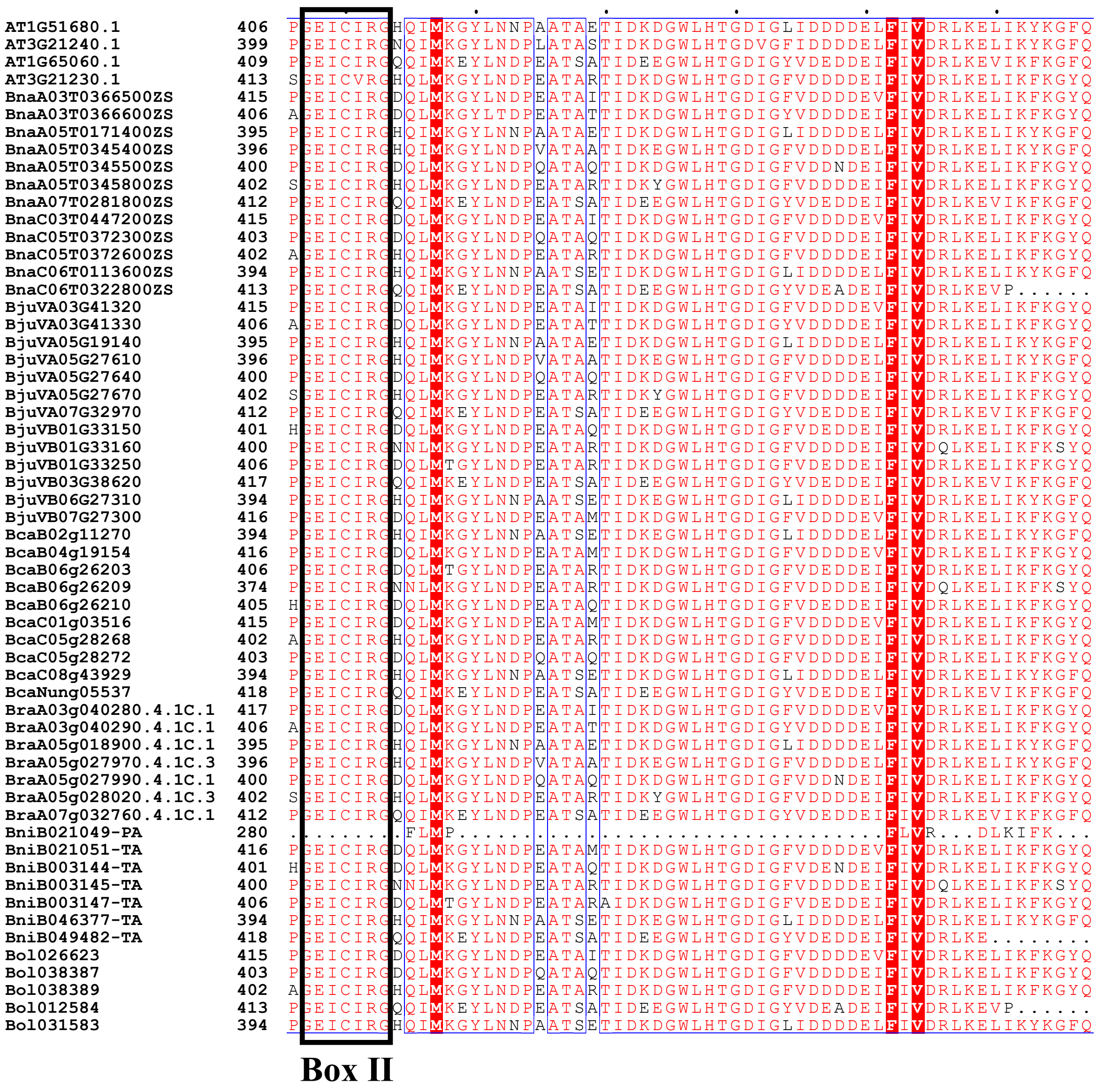
AT1G51680.1 406 PGEICIRGHQIMKGYLNNPAATAETIDKDGWLHTGDIGLIDDDDELFIVDRLKELIKYKGFQ
AT3G21240.1 399 PGEICIRGNQIMKGYLNDPLATASTIDKDGWLHTGDVGFIDDDDELFIVDRLKELIKYKGFQ
AT1G65060.1 409 PGEICIRGQQIMKEYLNDPEATSATIDEEGWLHTGDIGYVDEDDEIFIVDRLKEVIKFKGFQ
AT3G21230.1 413 SGEICVRGHQLMKGYLNDPEATARTIDKDGWLHTGDIGFVDDDDEIFIVDRLKELIKFKGYQ
BnaA03T0366500ZS 415 PGEICIRGDQLMKGYLNDPEATAITIDKDGWLHTGDIGFVDDDDEVFIVDRLKELIKFKGYQ
BnaA03T0366600ZS 406 AGEICIRGDQLMKGYLTDPEATATTIDKDGWLHTGDIGYVDDDDEIFIVDRLKELIKFKGYQ
BnaA05T0171400ZS 395 PGEICIRGHQIMKGYLNNPAATAETIDKDGWLHTGDIGLIDDDDELFIVDRLKELIKYKGFQ
BnaA05T0345400ZS 396 PGEICIRGHQIMKGYLNDPVATAATIDKEGWLHTGDIGFVDDDDELFIVDRLKELIKYKGFQ
BnaA05T0345500ZS 400 PGEICIRGDQLMKGYLNDPQATAQTIDKDGWLHTGDIGFVDDNDEIFIVDRLKELIKFKGYQ
BnaA05T0345800ZS 402 SGEICIRGHQLMKGYLNDPEATARTIDKYGWLHTGDIGFVDDDDEIFIVDRLKELIKFKGYQ
BnaA07T0281800ZS 412 PGEICIRGQQIMKEYLNDPEATSATIDEEGWLHTGDIGYVDEDDEIFIVDRLKEVIKFKGFQ
BnaC03T0447200ZS 415 PGEICIRGDQLMKGYLNDPEATAITIDKDGWLHTGDIGFVDDDDEVFIVDRLKELIKFKGYQ
BnaC05T0372300ZS 403 PGEICIRGDQLMKGYLNDPQATAQTIDKDGWLHTGDIGFVDDDDEIFIVDRLKELIKFKGYQ
BnaC05T0372600ZS 402 AGEICIRGHQLMKGYLNDPEATARTIDKDGWLHTGDIGFVDDDDEIFIVDRLKELIKFKGYQ
BnaC06T0113600ZS 394 PGEICIRGHQIMKGYLNNPAATSETIDKDGWLHTGDIGLIDDDDELFIVDRLKELIKYKGFQ
BnaC06T0322800ZS 413 PGEICIRGQQIMKEYLNDPEATSATIDEEGWLHTGDIGYVDEADEIFIVDRLKEVP......
BjuVA03G41320 415 PGEICIRGDQLMKGYLNDPEATAITIDKDGWLHTGDIGFVDDDDEVFIVDRLKELIKFKGYQ
BjuVA03G41330 406 AGEICIRGDQLMKGYLNDPEATATTIDKDGWLHTGDIGYVDDDDEIFIVDRLKELIKFKGYQ
BjuVA05G19140 395 PGEICIRGHQIMKGYLNNPAATAETIDKDGWLHTGDIGLIDDDDELFIVDRLKELIKYKGFQ
BjuVA05G27610 396 PGEICIRGHQIMKGYLNDPVATAATIDKEGWLHTGDIGFVDDDDELFIVDRLKELIKYKGFQ
BjuVA05G27640 400 PGEICIRGDQLMKGYLNDPQATAQTIDKDGWLHTGDIGFVDDDDEIFIVDRLKELIKFKGYQ
BjuVA05G27670 402 SGEICIRGHQLMKGYLNDPEATARTIDKYGWLHTGDIGFVDDDDEIFIVDRLKELIKFKGYQ
BjuVA07G32970 412 PGEICIRGQQIMKEYLNDPEATSATIDEEGWLHTGDIGYVDEDDEIFIVDRLKEVIKFKGFQ
BjuVB01G33150 401 HGEICIRGDQLMKGYLNDPEATAQTIDKDGWLHTGDIGFVDEDDEIFIVDRLKELIKFKGYQ
BjuVB01G33160 400 PGEICIRGNNLMKGYLNDPEATARTIDKDGWLHTGDIGFVDDDDEIFIVDQLKELIKFKSYQ
BjuVB01G33250 406 PGEICIRGDQLMTGYLNDPEATARTIDKDGWLHTGDIGFVDEDDEIFIVDRLKELIKFKGYQ
BjuVB03G38620 417 PGEICIRGQQIMKEYLNDPEATSATIDEEGWLHTGDIGYVDEDDEIFIVDRLKEVIKFKGFQ
BjuVB06G27310 394 PGEICIRGHQIMKGYLNNPAATSETIDKEGWLHTGDIGLIDDDDELFIVDRLKELIKYKGFQ
BjuVB07G27300 416 PGEICIRGDQLMKGYLNDPEATAMTIDKDGWLHTGDIGFVDDDDEVFIVDRLKELIKFKGYQ
BcaB02g11270 394 PGEICIRGHQIMKGYLNNPAATSETIDKEGWLHTGDIGLIDDDDELFIVDRLKELIKYKGFQ
BcaB04g19154 416 PGEICIRGDQLMKGYLNDPEATAMTIDKDGWLHTGDIGFVDDDDEVFIVDRLKELIKFKGYQ
BcaB06g26203 406 PGEICIRGDQLMTGYLNDPEATARTIDKDGWLHTGDIGFVDEDDEIFIVDRLKELIKFKGYQ
BcaB06g26209 374 PGEICIRGNNLMKGYLNDPEATARTIDKDGWLHTGDIGFVDDDDEIFIVDQLKELIKFKSYQ
BcaB06g26210 405 HGEICIRGDQLMKGYLNDPEATAQTIDKDGWLHTGDIGFVDEDDEIFIVDRLKELIKFKGYQ
BcaC01g03516 415 PGEICIRGDQLMKGYLNDPEATAMTIDKDGWLHTGDIGFVDDDDEVFIVDRLKELIKFKGYQ
BcaC05g28268 402 AGEICIRGHQLMKGYLNDPEATARTIDKDGWLHTGDIGFVDDDDEIFIVDRLKELIKFKGYQ
BcaC05g28272 403 PGEICIRGDQLMKGYLNDPQATAQTIDKDGWLHTGDIGFVDDDDEIFIVDRLKELIKFKGYQ
BcaC08g43929 394 PGEICIRGHQIMKGYLNNPAATSETIDKDGWLHTGDIGLIDDDDELFIVDRLKELIKYKGFQ
BcaNung05537 418 PGEICIRGQQIMKEYLNDPEATSATIDEEGWLHTGDIGYVDEDDEIFIVDRLKEVIKFKGFQ
BraA03g040280.4.1C.1 417 PGEICIRGDQLMKGYLNDPEATAITIDKDGWLHTGDIGFVDDDDEVFIVDRLKELIKFKGYQ
BraA03g040290.4.1C.1 406 AGEICIRGDQLMKGYLNDPEATATTIDKDGWLHTGDIGYVDDDDEIFIVDRLKELIKFKGYQ
BraA05g018900.4.1C.1 395 PGEICIRGHQIMKGYLNNPAATAETIDKDGWLHTGDIGLIDDDDELFIVDRLKELIKYKGFQ
BraA05g027970.4.1C.3 396 PGEICIRGHQIMKGYLNDPVATAATIDKEGWLHTGDIGFVDDDDELFIVDRLKELIKYKGFQ
BraA05g027990.4.1C.1 400 PGEICIRGDQLMKGYLNDPQATAQTIDKDGWLHTGDIGFVDDNDEIFIVDRLKELIKFKGYQ
BraA05g028020.4.1C.3 402 SGEICIRGHQLMKGYLNDPEATARTIDKYGWLHTGDIGFVDDDDEIFIVDRLKELIKFKGYQ
BraA07g032760.4.1C.1 412 PGEICIRGQQIMKEYLNDPEATSATIDEEGWLHTGDIGYVDEDDEIFIVDRLKEVIKFKGFQ
BniB021049-PA 280 .........FLMP.........................FLVR...DLKIFK...
BniB021051-TA 416 PGEICIRGDQLMKGYLNDPEATAMTIDKDGWLHTGDIGFVDDDDEVFIVDRLKELIKFKGYQ
BniB003144-TA 401 HGEICIRGDQLMKGYLNDPEATAQTIDKDGWLHTGDIGFVDENDEIFIVDRLKELIKFKGYQ
BniB003145-TA 400 PGEICIRGNNLMKGYLNDPEATARTIDKDGWLHTGDIGFVDDDDEIFIVDQLKELIKFKSYQ
BniB003147-TA 406 PGEICIRGDQLMTGYLNDPEATARAIDKDGWLHTGDIGFVDEDDEIFIVDRLKELIKFKGYQ
BniB046377-TA 394 PGEICIRGHQIMKGYLNNPAATSETIDKEGWLHTGDIGLIDDDDELFIVDRLKELIKYKGFQ
BniB049482-TA 418 PGEICIRGQQIMKEYLNDPEATSATIDEEGWLHTGDIGYVDEDDEIFIVDRLKE........
Bol026623 415 PGEICIRGDQLMKGYLNDPEATAITIDKDGWLHTGDIGFVDDDDEVFIVDRLKELIKFKGYQ
Bol038387 403 PGEICIRGDQLMKGYLNDPQATAQTIDKDGWLHTGDIGFVDDDDEIFIVDRLKELIKFKGYQ
Bol038389 402 AGEICIRGHQLMKGYLNDPEATARTIDKDGWLHTGDIGFVDDDDEIFIVDRLKELIKFKGYQ
Bol012584 413 PGEICIRGQQIMKEYLNDPEATSATIDEEGWLHTGDIGYVDEADEIFIVDRLKEVP......
Bol031583 394 PGEICIRGHQIMKGYLNNPAATSETIDKDGWLHTGDIGLIDDDDELFIVDRLKELIKYKGFQ
Box II

```
AT1G51680.1           468  VAPAELEALLIGHPDITDVAVVA.....MKEEAAGEVPVAFVVKSKDSELSEDDVKQFVSKQ
AT3G21240.1           461  VAPAELESLLIGHPEINDVAVVA.....MKEEDAGEVPVAFVVRSKDSNISEDEIKQFVSKQ
AT1G65060.1           471  VPPAELESLLINHHSIADAAVVP.....QNDEVAGEVPVAFVVRSNGNDITEEDVKEYVAKQ
AT3G21230.1           475  VAPAELEALLISHPSIDDAAVVA.....MKDEVADEVPVAFVARSQGSQLTEDDVKSYVNKQ
BnaA03T0366500ZS      477  VAPAELEALLISHPYIEDAAVVA.....MKDEVADEVPVAYVVRSEGSHLTEDDVKSYVNKQ
BnaA03T0366600ZS      468  VAPAELEALLISHPCIEDAAVVA.....MKDEVAGEVPVAFVVSSEGSQLTEDDVTSYICKQ
BnaA05T0171400ZS      457  VAPAELEALLIGHQDITDVAVVA.....MKEEAAGEVPVAFVVKSKDSELSEDDVKQFVAKQ
BnaA05T0345400ZS      458  VAPAELESLLISHSDINDVAVVA.....MKEDDAGEVPVAFVVRSKESNLSEDEIKQFVSKQ
BnaA05T0345500ZS      462  MAPAELEALLISHPFIEDVAVVA.....MKDEIAGEVPVVFVVRSEGSQLTEDDVMSYINKQ
BnaA05T0345800ZS      464  VAPAELEALLIFHPYIEDAAVVA.....MKDEVADEVPVAFVVRSKGSQLTEDDIKNYVNKQ
BnaA07T0281800ZS      474  ASP..LTIFVFN..................................................
BnaC03T0447200ZS      477  VAPAELEALLISHPYIEDAAVVA.....MKDEVADEVPVAYVVRSEGSQLTEDDVKSYVNKQ
BnaC05T0372300ZS      465  VAPAELEALLISHPFIEDVAVVA.....MKDEIAGEVPVVFVVRSEGSQLTEDDVTSCINKQ
BnaC05T0372600ZS      464  VAPAELEALLIFHPYIEDAAVVA.....MTDEVADEVPVAFVVRSEGSQLTEDDIKNYVNKQ
BnaC06T0113600ZS      456  VAPAELEALLIGHQDITDVAVVA.....MKEEAAGEVPVAFVVKSKDSELSEDDVKQFVAKQ
BnaC06T0322800ZS      469  ..PAELEALLINHHSIADAAVVP.....QRDEVAGEVPVAFVVRSNGNVITEEDIKEYIAKQ
BjuVA03G41320         477  VAPAELEALLISHPYIEDAAVVA.....MKDEVADEVPVAYVVRSEGSHLTEDDVKSYVNKQ
BjuVA03G41330         468  VAPAELEALLISHPCIEDAAVVA.....MKDEVAGEVPVAFVVSSEGSQLTEDDVTSYICKQ
BjuVA05G19140         457  VAPAELEALLIGHQDITDVAVVA.....MKEEAAGEVPVAFVVKSKDSELSEDDVKQFVAKQ
BjuVA05G27610         458  VAPAELESLLISHSDINDVAVVA.....MKEDDAGEVPVAFVVRSKESNLSEDEIKQFVSKQ
BjuVA05G27640         462  VAPAELEALLISHPFIEDVAVVA.....MKDEIAGEVPVVFVVRSEGSQLTEDDVTSYINKQ
BjuVA05G27670         464  VAPAELEALLIFHPYIEDAAVVP.....MKDEVADEVPVAFVVRSKGSQLTEDDIKNYVNKQ
BjuVA07G32970         474  VPPAELEALLINHHSIADAAVVP.....QRDEVAGEVPVAFVVRSNGNVITEEDIKEYIAKQ
BjuVB01G33150         463  VAPAELEALLISHPFIKDVAVVG.....MKDEVAGEIPVAFVVRSEGSQLTEDDVTRYINKQ
BjuVB01G33160         462  VGPAELEALLISHPCIEDAAVVA.....MKDEITDEVAVAFVVRSEGSHLTEDDVKSYLNTQ
BjuVB01G33250         468  VAPAELEALLISHPYIEDAAVVA.....MTDEVANEVPVAFVVKSEGYHITEEDVKNFVNKQ
BjuVB03G38620         479  VPPAELEALLINHHSIADAAVVP.....QRDEVAGEVPVAFVVRSNGNVITEEDIKEYIAKQ
BjuVB06G27310         456  VAPAELEALLIGHQDITDVAVVA.....MKEEAAGEVPVAFVVKSKDSELSEDDVKQFVAKQ
BjuVB07G27300         478  VAPAELEALLISHPCIEDAAVVA.....MKDEVADEVPVAFVVRSEGSQLTEDVVKSYVNKQ
BcaB02g11270          456  VAPAELEALLIGHQDITDVAVVA.....S.........VAFVVKSKDSELSEDDVKQFVAKQ
BcaB04g19154          478  VAPAELEALLISHPCIEDAAVVA.....MKDEVADEVPVAFVVRSEGSQLTEDVVKSYVNKQ
BcaB06g26203          468  VAPAELEALLISHPYIEDAAVVA.....MTDEVANEVPVAFVVKSEGYHITEEDVKNFVNKQ
BcaB06g26209          436  VGPAELEALLISHPCIEDAAVVA.....IEDEITDDVAVAFVVRSEGSHLTEDDVKSYLNTQ
BcaB06g26210          467  VAPAELEALLISHPFIKDVAVVG.....MKDEVAGEIPVAFVVRSEGSQLTEDDVTRYINKQ
BcaC01g03516          477  VAPAELEALLISHPCIEDAAVVA.....MKDEVADEVPVAFVVRSEGSQLTEDVVKSYVNKQ
BcaC05g28268          464  VAPAELEALLIFHPYIEDAAVVA.....MTDEVADEVPVAFVVRSEGSQLTEDDIKNYVNKQ
BcaC05g28272          465  VAPAELEALLISHPFIEDVAVVA.....MKDEIAGEVPVVFVVRSEGSQLTEDDVTSCINKQ
BcaC08g43929          456  VAPAELEALLIGHQDITDVAVVA.....MKEEAAGEVPVAFVVKSKDSELSEDDVKQFVAKQ
BcaNung05537          480  VPPAELEALLINHHSIADAAVVP.....QRDEVAGEVPVAFVVRSNGNVITEEDIKEYIAKQ
BraA03g040280.4.1C.1  479  VAPAELEALLISHPYIEDAAVVA.....MKDEVADEVPVAYVVRSEGSQLTEDDVKSYVNKQ
BraA03g040290.4.1C.1  468  VAPAELEALLISHPCIEDAAVVA.....MKDEVAGEVPVAFVVSSEGSQLTEDDVTSYICKQ
BraA05g018900.4.1C.1  457  VAPAELEALLIGHQDITDVAVVA.....MKEEAAGEVPVAFVVKSKDSELSEDDVKQFVAKQ
BraA05g027970.4.1C.3  458  VAPAELESLLISHSDINDVAVVAYVDFDMKEDDAGEVPVAFVVRSKESNLSEDEIKQFVSKQ
BraA05g027990.4.1C.1  462  VAPAELEALLISHPFIEDVAVVA.....MKDEIAGEVPVVFVVRSEGSQLTEDDVMSYINKQ
BraA05g028020.4.1C.3  464  VAPAELEALLIFHPYIEDAAVVA.....MKDEVADEVPVAFVVRSKGSQLTEDDIKNYVNKQ
BraA07g032760.4.1C.1  474  VPPAELEALLINHHSIADAAVVP.....QRDEVAGEVPVAFVVRSNGNVITEEDIKEYIAKQ
BniB021049-PA         294  ....................................MPVLFTAADAAS..............
BniB021051-TA         478  VAPAELEALLISHPCIEDAAVVA.....MKDEVADEVPVAFVVRSEGSQLTEDVVKSYVNKQ
BniB003144-TA         463  VAPAELEALLISHPFIKDVAVVG.....MKDEVAGEIPVAFVVRSEGSQLTEDDVRRCINKQ
BniB003145-TA         462  VGPAELEALLISHPCIEDAAVVA.....MKDEITDEVAVAFVVRSEGSHLTEDDVKSYLNTQ
BniB003147-TA         468  VAPAELEALLISHPYIEDAAVVA.....MIDEVANEVPVAFVVKSEGYHITEEDVKNFVNKQ
BniB046377-TA         456  VAPAELEALLIGHQDITDVAVVA.....MKEEAAGEVPVAFVVKSKDSELSEDDVKQFVAKQ
BniB049482-TA         472  VPPAELEALLINHHSIADAAVVP.....QRDEVAGEVPVAFVVRSNGNVITEEDIKEYIAKQ
Bol026623             477  VAPAELEALLISHPYIEDAAVVA.....MKDEVADEVPVAYVVRSEGSQLTEDDVKSYVNKQ
Bol038387             465  VAPAELEALLISHPFIEDVAVVA.....MKDEIAGEVPVVFVVRSEGSQLTEDDVTSCINKQ
Bol038389             464  VAPAELEALLIFHPYIEDAAVVA.....MTDEVADEVPVAFVVRSEGSQLTEDDIKNYVNKQ
Bol012584             469  ..PAELEALLINHHSIADAAVVP.....QRDEVAGEVPVAFVVRSNGNVITEEDIKEYIAKQ
Bol031583             456  VAPAELEALLIGHQDITDVAVVA.....MKEEAAGEVPVAFVVKSKDSELSEDDVKQFVAKQ
```

```
AT1G51680.1            525  .VVFYK.......RINKVFFTESIPKAPSGKILRKDLRAKLANGL.................
AT3G21240.1            518  .VVFYK.......RINKVFFTDSIPKAPSGKILRKDLRARLANGLMN...............
AT1G65060.1            528  VVFYKR........LHKVFFVASIPKSPSGKILRKDLKAKLC....................
AT3G21230.1            532  .VVHYK.......RIKMVFFIEVIPKAVSGKILRKDLRAKLETMCSK...............
BnaA03T0366500ZS       534  .VVHYK.......RIKMVFFIEAIPKAVSGKILRKELRAKLESEYPK...............
BnaA03T0366600ZS       525  .VVHYK.......RVKMVFFTDSIPKAPSGKILRKDLRARLANGLMNLS.............
BnaA05T0171400ZS       514  .VVFYK.......RINKVFFVESIPKAPSGKILRKDLRAKLANGLVN...............
BnaA05T0345400ZS       515  .VVFYK.......RINKVFFTDSIPKAPSGKILRKDLRARLANGLT................
BnaA05T0345500ZS       519  .VVHYK.......RIKMVFFTDCIPKAPSGKILRKDLRARLANGLMNKS.............
BnaA05T0345800ZS       521  .VVHYK.......RIKMVFFVKAIPKSASGKLLRKVLRAKL.....................
BnaA07T0281800ZS            ..............................................................
BnaC03T0447200ZS       534  .VVHYK.......RIKMVFFIEAIPKAVSGKILRKELRAKLESEYPK...............
BnaC05T0372300ZS       522  .VVHYK.......RIKMVFFTDSIPKAPSGKILRKDLRARLANGLMNKS.............
BnaC05T0372600ZS       521  .VVHYK.......RIKMVFFVKAIPKSASGKLLRKVLRAKL.....................
BnaC06T0113600ZS       513  .VVFYK.......RINKVFFVESIPKAPSGKILRKDLRAKLANGLVN...............
BnaC06T0322800ZS       524  VVFYKR........LHKVFFVPSIPKSPSGKILRKDLKAKLC....................
BjuVA03G41320          534  .VVHYK.......RIKMVFFIEAIPKAVSGKILRKELRAKLESEYPK...............
BjuVA03G41330          525  .VVHYK.......RVKMVFFTDSIPKAPSGKILRKDLRARLANGLMNLSLNCQMCYLIIHAN
BjuVA05G19140          514  .VVFYK.......RINKVFFVESIPKAPSGKILRKDLRAKLVNGLVN...............
BjuVA05G27610          515  .VVFYK.......RINKVFFTDSIPKAPSGKILRKDLRARLANGLT................
BjuVA05G27640          519  .VVHYK.......RIKMVFFTDCIPKAPSGKILRKDLRARLANGLMNKS.............
BjuVA05G27670          521  .VVHYK.......RIKMVFFVKAIPKSASGKLLRKVLRAKL.....................
BjuVA07G32970          531  VVFYKR........LHKVFFVPSIPKSPSGKILRKDLKAKLC....................
BjuVB01G33150          520  .VVHYK.......RIKMVFFTDFIPKAPSGKILRKDLRARLSWIDEQKLNNLI.........
BjuVB01G33160          519  .VVHYK.......QIKLVLFIEAIPKADYGKILRKELRAKLETEYPK...............
BjuVB01G33250          525  .VVHYK.......RIKMVFFVKAIPKSASGKLLRKVLRAKL.....................
BjuVB03G38620          536  VVFYKR........LHKVFFVPSIPKSPSGKILRKDLKAKLC....................
BjuVB06G27310          513  .VVFYK.......RINKVFFVESIPKAPSGKILRKDLRAKLANGLVN...............
BjuVB07G27300          535  .VVHYK.......RIKMVFFIEAIPKAVSGKILRKELRAKLESEYL................
BcaB02g11270           504  .VVFYK.......RINKVFFVESIPKAPSGKILRKDLRAKLANGLVN...............
BcaB04g19154           535  .VVHYK.......RIKMVFFIEAIPKAVSGKILRKELRAKLESEYL................
BcaB06g26203           525  .VVHYK.......RIKMVFFVKAIPKSASGKLLRKVLRAKL.....................
BcaB06g26209           493  .VVHYK.......QIKMVLFIEAIPKAVYGKILRKELRTKLETEYPK...............
BcaB06g26210           524  .VVHYK.......RIKMVFFTDVIPKAPSGKILRKDLRARLS...NGLMNKS..........
BcaC01g03516           534  .VVHYK.......RIKMVFFIEAIPKAVS.....KDIAEGTPS...................
BcaC05g28268           521  .VVHYK.......RIKMVFFVKAIPKSASGKLLRKVLRAKL.....................
BcaC05g28272           522  .VVHYK.......RIKMVFFTDSIPKAPSGKILRKDLRARLANGLMNKS.............
BcaC08g43929           513  .VVFYK.......RINKVFFVESIPKAPSGKILRKDLRAKLANGLVN...............
BcaNung05537           537  VVFYKR........LHKVFFVPSIPKSPSGKILRKDLKAKLC....................
BraA03g040280.4.1C.1   536  .VVHYK.......RIKMVFFIEAIPKAVSGKILRKELRAKLESEYPK...............
BraA03g040290.4.1C.1   525  .VVHYK.......RVKMVFFTDSIPKAPSGKILRKDLRARLANGLMNLS.............
BraA05g018900.4.1C.1   514  .VVFYK.......RINKVFFVESIPKAPSGKILRKDLRAKLVNGLVN...............
BraA05g027970.4.1C.3   520  .VVFYK.......RINKVFFTDSIPKAPSGKILRKDLRARLANGLT................
BraA05g027990.4.1C.1   519  .VVHYK.......RIKMVFFTDCIPKAPSGKILRKDLRARLANGLMNKS.............
BraA05g028020.4.1C.3   521  .VVHYK.......RIKMVFFVKAIPKSASGKLLRKVLRAKL.....................
BraA07g032760.4.1C.1   531  VVFYKR........LHKVFFVPSIPKSPSGKILRKDLKAKLC....................
BniB021049-PA          306  ....................ASQINTSKSRK...............................
BniB021051-TA          535  .VVHYK.......RIKMVFFIEAIPKAVSGKILRKELRAKLESEYL................
BniB003144-TA          520  ELIKFKSYQVAPTELEAFLISHSYIEDAAIFAMKDEIADEVPVACVVRSEGS..........
BniB003145-TA          519  .VVHYK.......QIKMVLFIEAIPKAVYGKILRKELRAKLETEYPK...............
BniB003147-TA          525  .VVHYK.......RIKMVFFVKAIPKSASGKLLRKVLRAKL.....................
BniB046377-TA          513  .VVFYK.......RINKVFFVESIPKAPSGKILRKDLRAKLANGLVN...............
BniB049482-TA          529  ............................NKKVNTNAQKH.......................
Bol026623              534  .VVHYK.......RIKMVFFIEAIPKAVSGKILRKELRAKLESEYPK...............
Bol038387              522  .VVHYK.......RIKMVFFTDSIPKAPSGKILRKDLRARLANGLMNKS.............
Bol038389              521  .VVHYK.......RIKMVFFVKAIPKSASGKLLRKVLRAKL.....................
Bol012584              524  VVFYKR........LHKVFFVPSIPKSPSGKILRKDLKAKLC....................
Bol031583              513  .VVFYK.......RINKVFFVESIPKAPSGKILRKDLRAKLANGLVN...............
```

AT1G51680.1		....
AT3G21240.1		....
AT1G65060.1		....
AT3G21230.1		....
BnaA03T0366500ZS		....
BnaA03T0366600ZS		....
BnaA05T0171400ZS		....
BnaA05T0345400ZS		....
BnaA05T0345500ZS		....
BnaA05T0345800ZS		....
BnaA07T0281800ZS		....
BnaC03T0447200ZS		....
BnaC05T0372300ZS		....
BnaC05T0372600ZS		....
BnaC06T0113600ZS		....
BnaC06T0322800ZS		....
BjuVA03G41320		....
BjuVA03G41330	579	KKIVDKQRILNHSI
BjuVA05G19140		....
BjuVA05G27610		....
BjuVA05G27640		....
BjuVA05G27670		....
BjuVA07G32970		....
BjuVB01G33150		....
BjuVB01G33160		....
BjuVB01G33250		....
BjuVB03G38620		....
BjuVB06G27310		....
BjuVB07G27300		....
BcaB02g11270		....
BcaB04g19154		....
BcaB06g26203		....
BcaB06g26209		....
BcaB06g26210		....
BcaC01g03516		....
BcaC05g28268		....
BcaC05g28272		....
BcaC08g43929		....
BcaNung05537		....
BraA03g040280.4.1C.1		....
BraA03g040290.4.1C.1		....
BraA05g018900.4.1C.1		....
BraA05g027970.4.1C.3		....
BraA05g027990.4.1C.1		....
BraA05g028020.4.1C.3		....
BraA07g032760.4.1C.1		....
BniB021049-PA		....
BniB021051-TA		....
BniB003144-TA		....
BniB003145-TA		....
BniB003147-TA		....
BniB046377-TA		....
BniB049482-TA		....
Bol026623		....
Bol038387		....
Bol038389		....
Bol012584		....
Bol031583		....
