# Supplementary material for: Genome-Wide Identification Analysis of the 4-Coumarate: Coa Ligase (4CL) Gene Family in Brassica U’s Triangle Species and Its Potential Role in the Accumulation of Flavonoids in Brassica napus L
Source: Plants (Basel). 2025 Feb 26;14(5):714. doi: 10.3390/plants14050714 (PMC11902127; doi:10.3390/plants14050714)
Supplement: Supplementary file 1 [file plants-14-00714-s001.zip › Supplementary Materials/Figure S3/Bca4CL1.pdf]

FGENESH 2.6 Prediction of potential genes in Arabidopsis_thaliana genomic DNA
Seq name: ChrB02 40145167 40202633
Length of sequence: 57467
Number of predicted genes 19: in +chain 7, in -chain 12.
Number of predicted exons 61: in +chain 13, in -chain 48.
Positions of predicted genes and exons: Variant 1 from 1, Score:1857.855664

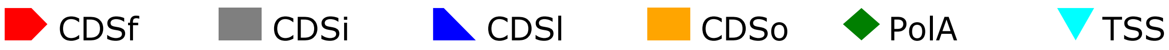

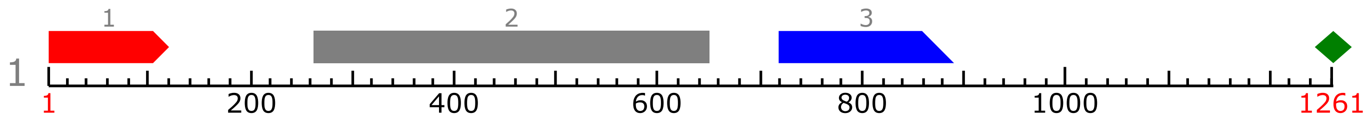

1	+	1	CDSf	1	–	119	8.11	1	–	117	117
1	+	2	CDSi	261	–	649	18.77	262	–	648	387
1	+	3	CDSl	717	–	889	13.51	719	–	889	171
1	+		PolA	1261			1.88				

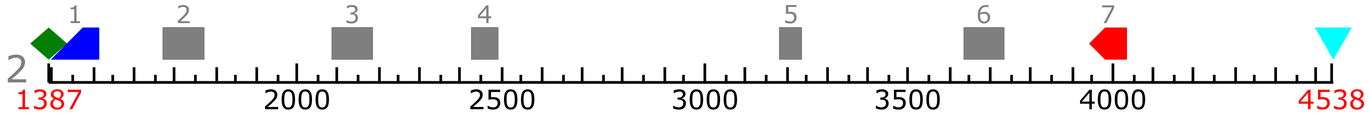

2	–		PolA	1387			-4.92				
2	–	1	CDSl	1392	–	1511	7.19	1392	–	1511	120
2	–	2	CDSi	1667	–	1769	25.55	1667	–	1768	102
2	–	3	CDSi	2081	–	2182	17.56	2083	–	2181	99
2	–	4	CDSi	2423	–	2490	9.71	2425	–	2490	66
2	–	5	CDSi	3179	–	3235	2.42	3179	–	3235	57
2	–	6	CDSi	3631	–	3731	11.24	3631	–	3729	99
2	–	7	CDSf	3939	–	4032	-6.41	3940	–	4032	93
2	–		TSS	4538			-2.12				

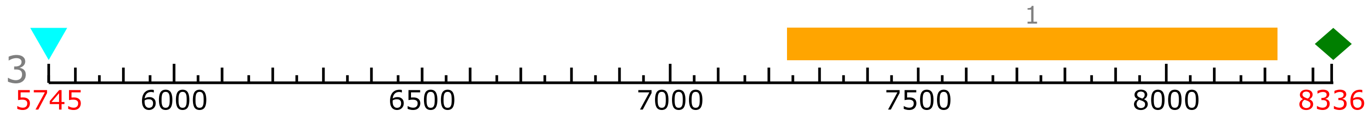

3	+		TSS	5745			0.29				
3	+	1	CDSo	7234	–	8223	115.26	7234	–	8223	990
3	+		PolA	8336			-0.32				

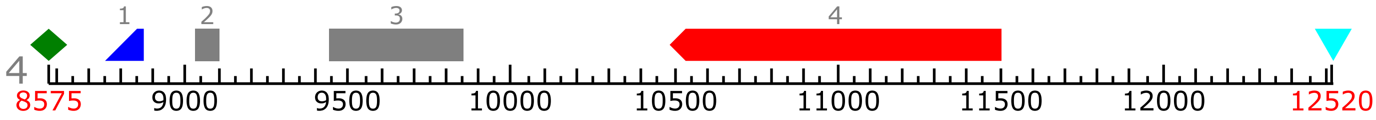

4	–		PolA	8575			1.88				
4	–	1	CDSl	8748	–	8867	6.69	8748	–	8867	120
4	–	2	CDSi	9024	–	9099	1.92	9024	–	9098	75

4	-	3	CDSi	9437	-	9849	77.87	9439	-	9849	411
4	-	4	CDSf	10482	-	11501	197.60	10482	-	11501	1020
4	-		TSS	12520			0.48				

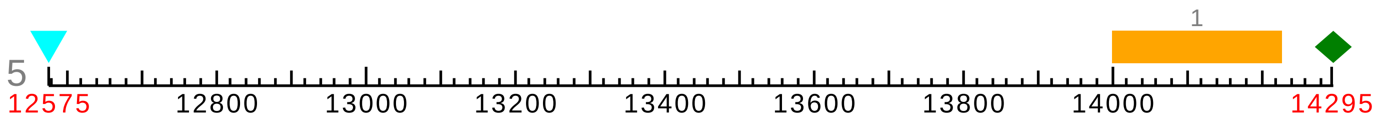

5	+		TSS	12575			1.18				
5	+	1	CDSo	13999	-	14226	4.35	13999	-	14226	228
5	+		PolA	14295			1.88				

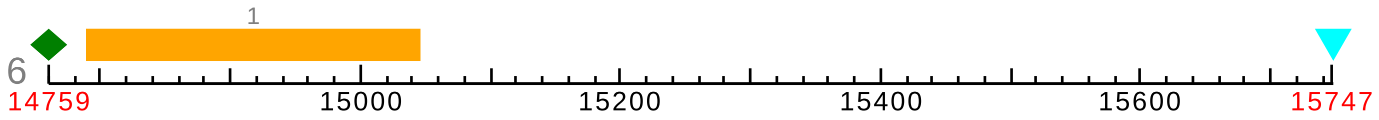

6	-		PolA	14759			-4.92				
6	-	1	CDSo	14788	-	15045	18.89	14788	-	15045	258
6	-		TSS	15747			-1.91				

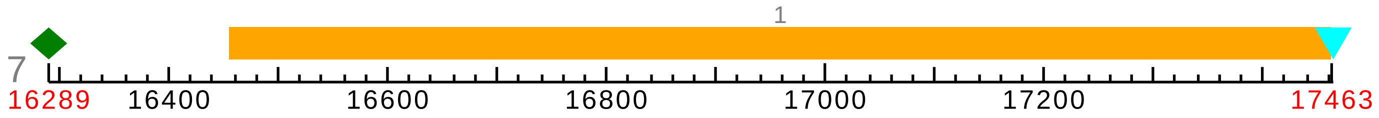

7	-		PolA	16289			1.88				
7	-	1	CDSo	16454	-	17461	61.33	16454	-	17461	1008
7	-		TSS	17463			-9.32				

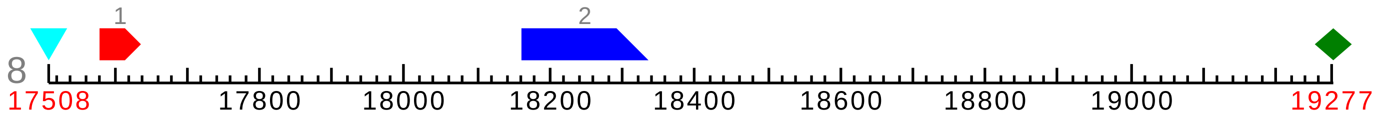

8	+		TSS	17508			-4.01				
8	+	1	CDSf	17578	-	17635	-0.19	17578	-	17634	57
8	+	2	CDSl	18159	-	18334	19.99	18161	-	18334	174
8	+		PolA	19277			1.88				

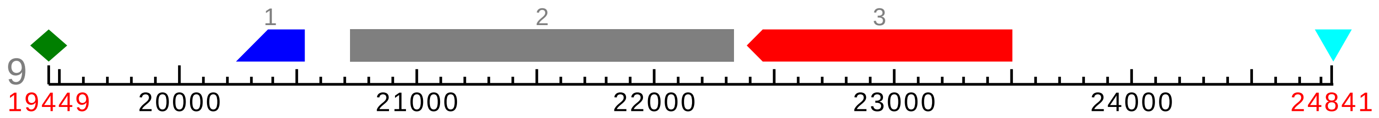

9	-		PolA	19449			1.88				
9	-	1	CDSl	20235	-	20524	37.04	20235	-	20522	288
9	-	2	CDSi	20715	-	22326	134.92	20716	-	22326	1611
9	-	3	CDSf	22379	-	23494	117.58	22379	-	23494	1116
9	-		TSS	24841			3.80				

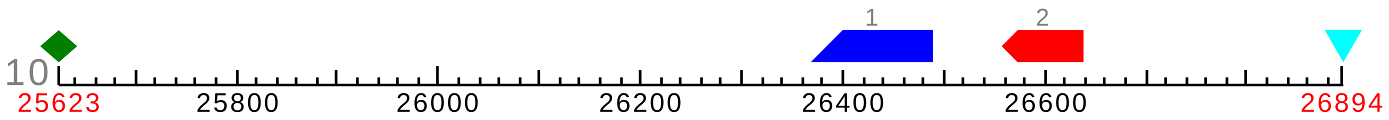

10	-		PolA	25623			1.88				
10	-	1	CDSl	26367	-	26488	1.26	26367	-	26486	120
10	-	2	CDSf	26556	-	26637	3.69	26557	-	26637	81
10	-		TSS	26894			0.18				

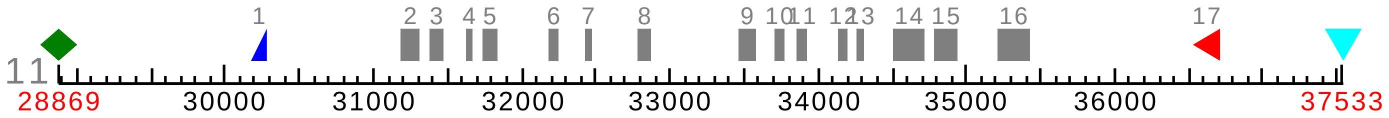

11	-		PolA	28869			1.88				
11	-	1	CDSl	30166	-	30273	6.76	30166	-	30273	108
11	-	2	CDSi	31177	-	31302	8.57	31177	-	31302	126
11	-	3	CDSi	31371	-	31463	6.35	31371	-	31463	93
11	-	4	CDSi	31616	-	31657	0.33	31616	-	31657	42
11	-	5	CDSi	31726	-	31827	10.42	31726	-	31827	102
11	-	6	CDSi	32174	-	32239	-0.09	32174	-	32239	66
11	-	7	CDSi	32419	-	32463	0.86	32419	-	32463	45
11	-	8	CDSi	32774	-	32864	3.39	32774	-	32863	90
11	-	9	CDSi	33454	-	33570	1.70	33456	-	33569	114
11	-	10	CDSi	33696	-	33763	1.90	33698	-	33763	66
11	-	11	CDSi	33846	-	33915	2.71	33846	-	33914	69
11	-	12	CDSi	34128	-	34189	-2.56	34130	-	34189	60
11	-	13	CDSi	34252	-	34300	7.80	34252	-	34299	48
11	-	14	CDSi	34497	-	34709	3.68	34499	-	34708	210
11	-	15	CDSi	34773	-	34931	4.66	34775	-	34930	156
11	-	16	CDSi	35201	-	35417	7.88	35203	-	35415	213
11	-	17	CDSf	36519	-	36702	11.29	36520	-	36702	183
11	-		TSS	37533			1.78				

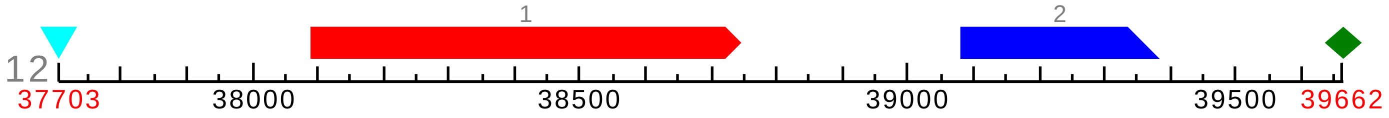

12	+		TSS	37703			-3.51				
12	+	1	CDSf	38087	-	38744	131.86	38087	-	38743	657
12	+	2	CDSl	39078	-	39382	40.00	39080	-	39382	303
12	+		PolA	39662			-4.02				

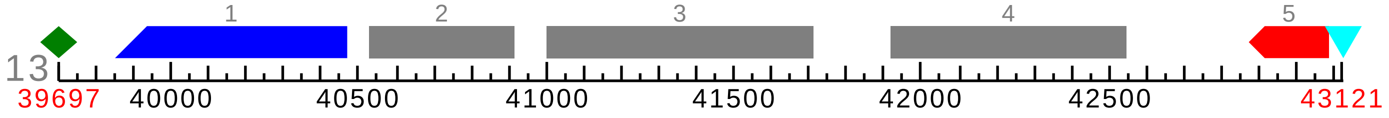

13	-		PolA	39697			-4.22				
13	-	1	CDSl	39847	-	40466	72.99	39847	-	40464	618
13	-	2	CDSi	40524	-	40911	30.44	40525	-	40911	387
13	-	3	CDSi	40997	-	41708	37.66	40997	-	41707	711
13	-	4	CDSi	41914	-	42543	39.82	41916	-	42542	627

13	-	5	CDSf	42869	-	43083	18.26	42871	-	43083	213
13	-		TSS	43121			-8.10				

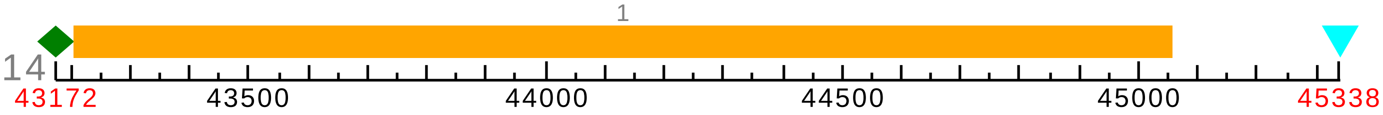

14	-		PolA	43172			-4.92				
14	-	1	CDSo	43202	-	45055	191.95	43202	-	45055	1854
14	-		TSS	45338			-1.30				

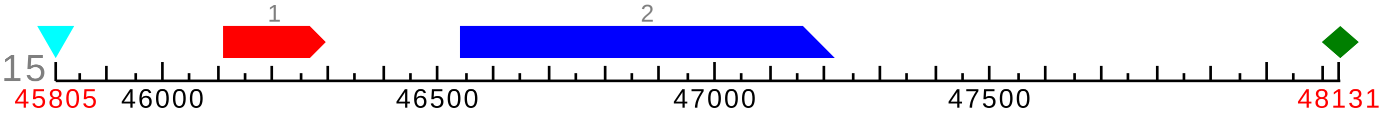

15	+		TSS	45805			-2.01				
15	+	1	CDSf	46108	-	46294	7.33	46108	-	46293	186
15	+	2	CDSl	46537	-	47216	83.32	46539	-	47216	678
15	+		PolA	48131			-2.92				

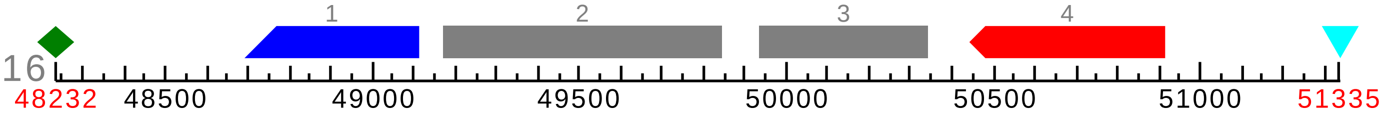

16	-		PolA	48232			-0.32				
16	-	1	CDSl	48688	-	49110	43.24	48688	-	49110	423
16	-	2	CDSi	49168	-	49841	73.65	49168	-	49839	672
16	-	3	CDSi	49931	-	50339	42.58	49932	-	50339	408
16	-	4	CDSf	50439	-	50912	68.01	50439	-	50912	474
16	-		TSS	51335			2.28				

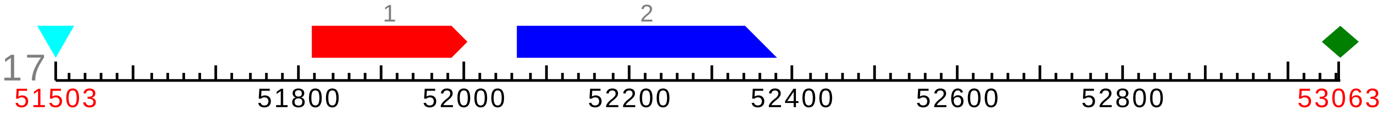

17	+		TSS	51503			-3.91				
17	+	1	CDSf	51814	-	52003	23.70	51814	-	52002	189
17	+	2	CDSl	52063	-	52379	43.63	52065	-	52379	315
17	+		PolA	53063			-3.72				

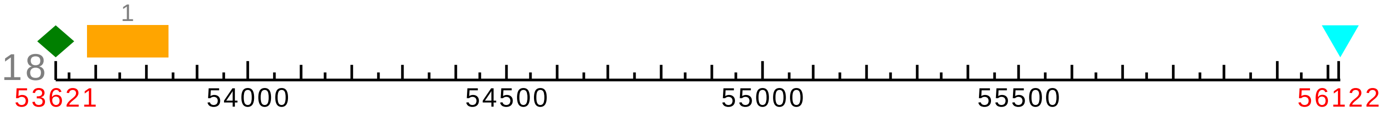

18	-		PolA	53621			1.88				
18	-	1	CDSo	53682	-	53840	-1.18	53682	-	53840	159
18	-		TSS	56122			1.68				

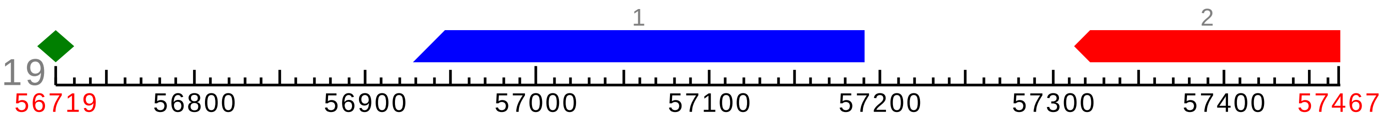

19 -		PolA	56719		1.88				
19 -	1	CDSl	56927 -	57190	22.16	56927 -	57190	264
19 -	2	CDSf	57312 -	57467	13.72	57312 -	57467	156

Predicted protein(s):

>FGENESH:[mRNA] 1 3 exon (s) 1 - 889 681 bp, chain +
ATGTACCTCTCCCTCCAACCAACTTCCGGCGGTTCCACCACCACCTCAGAGCCTAAAAGC
TACTCAGACTTAGTACGTGGCAACCGAATCGGAAGCGGAGCAGGTGGAACCTTTACAAAA
TGTACGATCAAAACGGAGAGATCCAGGTCTTGCTCGAGTTCATGGATCAAGGATCTTTGG
AAAGTGCTCATGTCTGGAACGAGCAGCAGTTGTCCGAGCTCTCGTCAGATACTAAACGGT
TTGGCTTATCTTCACGGCCGTCATATAGTCCATCGAGACATAAAGCCTTCGAATCTGCTT
ATAAACTCTGACAATAACGTCAAGATTGCTGATTTTGGAGTAAGTAGGGTCTTGGCTCAG
ACCCTGTCTCCTTGTAAGTCCTCTGTTGGGACTATCGCTTACATGAGTCCTGAGAGGATC
AACACGGATTTGAATCAGGGGATGTATGATGGGTGTGCTGGTGATATTTGGAGCTTTGGT
GTTAGTGTTCTTGAGTTTTTCTTGGGAGCCCCTGAAGCTCCGGCCACCGCCTCTCCCGAG
TTTAGGCACTTTGTTTCGTGTTGTTTGCAGAGAGAACCGGCGAAGAGGCAAACTGCTGTT
CAGCTTTTGCAACATCCTTTTGTACGTAGAGGAGCGAGTCAGAGTCAGAATCTACGTCAA
CTCTTGCCTCCTCCACACTAA
>FGENESH: 1 3 exon (s) 1 - 889 226 aa, chain +
MYLSLQPTSGGSTTTSEPKSYSDLVRGNRIGSGAGGTFTKCTIKTERSRSCSSSWIKDLW
KVLMSGTSSSCPSSRQILNGLAYLHGRHIVHRDIKPSNLLINSDNNVKIADFGVSRVLAQ
TLSPCKSSVGTIAYMSPERINTDLNQGMYDGCAGDIWSFGVSVLEFFLGAPEAPATASPE
FRHFVSCCLQREPAKRQTAVQLLQHPFVRRGASQSQNLRQLLPPPH
>FGENESH:[mRNA] 2 7 exon (s) 1392 - 4032 645 bp, chain -
ATGCAGGATATGTGGATCCAAGCAAAGGGCAAAATAAGAATGAAATTTGAGAGGAAAGAA
ATGAGGATAGATTGCAAATTAGAGGCCCATTTAAAAATAGTGGGCTCGGTACCCATTCGG
GTTCGGTTCGGATCTATTTGGGTTTCGGGTTTCGGGTTTCGGGTTTTCCGGGTCAAAGAT
TTCAACCCCATTCAGGTATATAAATCTATTTCGGATACATTCGGGTACCCGAAATACTTC
GGTTCGGATCGGGGATACGGAATGACGGAAGCAGGTCCGGTGCTAGCAATGTCGTTAGGG
TTCGCAAAGAGCCGTTTCCGATTGAAAGAGCTTATCAAGTACAAAGGTTTTCAAGTGGCT
CCGGCTGAGCTAGAGGCTCTCCTCATCGGTCATCAGGATATCACTGACGTCGCCGTCGTC
GCAATGAAAGAAGAGGCTGCTGGTGAAGTTCCCGTTGCATTCGTTGTGAAATCCAAGGAT
TCAGAGTTATCAGAAGATGATGTGAAACAATTCGTGGCGAAACAGGTTGTGTTTTACAAG
AGAATCAACAAAGTGTTCTTCGTTGAGTCCATTCCTAAAGCTCCATCAGGGAAGATATTG
AGGAAAGATCTGAGGGCAAAACTAGCAAATGGGTTGGTGAACTAA
>FGENESH: 2 7 exon (s) 1392 - 4032 214 aa, chain -
MQDMWIQAKGKIRMKFERKEMRIDCKLEAHLKIVGSVPIRVRFGSIWVSGFGFRVFRVKD
FNPIQVYKSISDTFGYPKYFGSDRGYGMTEAGPVLAMSLGFAKSRFRLKELIKYKGFQVA
PAELEALLIGHQDITDVAVVAMKEEAAGEVPVAFVVKSKDSELSEDDVKQFVAKQVVFYK
RINKVFFVESIPKAPSGKILRKDLRAKLANGLVN
>FGENESH:[mRNA] 3 1 exon (s) 7234 - 8223 990 bp, chain +
ATGAAACCAATCCAACCGCCACTGGGACTAACCGCTCCGGTTAAGAACCGTCCTCGCCGC
CGTCCAGACCTCTCCTTACCGCTTCCTCACAGGGACGTTTCCCTAGATGTACCTCTCTCC
CTCCAACCAACTTCCGGCGGTTCCACCACCACCTCAGAGCCTAAAAGCTACTCAGACTTA
GTACGTGGCAACCGAATCGGAAGCGGAGCAGGTGGAACCTTTTACAAAGTAGTCCACCGT

CCAACCTCTCGCGTATACGCGCTCAAGATAATCAACGGTAACCACGATGACACTGTTCGG
CGCCAAATCTGCAGAGAGATCGAGATTCTCCGAGACGTGAACCACCCCAACGTGGTGAAA
TGTCACGAGATGTTCGATCACAACGGAGAGATCCAGGTCTTGCTTGAGTTCATGGATAAA
GGATCTTTAGAAGGTGCTCATGTCTGGAACGAGGAGCACTTATCTGACCTCTCACGTCAG
ATATTAAAAGGTTTGGCTTATCTTCATGGCCGTCATGTAGTCCATCGAGCCATAACCCCT
TCGAATCTACTTATAAACTCGAACAATAACGTCAAGATTGCTGATTTTGGAGTGAGTAGG
GTCTTGGCTCAGACCCTGTCCTCTTTTGGGACTCTTGCTTACATGAGTCCTGAGACGTTC
AACACGGATTTGAATCAGGGGATGTATGATGGCTGTGCTGGTGATATTTGGAGCTTTGGT
GTTAGTGTTCTTGAGTTTTTCTTGGGGAGGTTTCCTTTTGATGTGAGTAGGCAAGGTGAT
TGGGCGAGTCTTATATGTGCTATTTGTATGTCTCAGCCGCCTAAAGCTCCGGCCACGGCC
TCTCCGGAGTTTAGACACTTTGTTTCATGTTGCTTGCAGAGAGAACCGGCGAGGAGGCTA
ACTGCTCTTGAGCTTTTGCAACATCCTTTTGTGCGCAGAGGAGCGAGTCAGAGTCAGAAT
CTACGTCAACTCTTGCCTCCTCCACACTAA
>FGENESH: 3 1 exon (s) 7234 - 8223 329 aa, chain +
MKPIQPPLGLTAPVKNRPRRRPDLSLPLPHRDVSLDVPLSLQPTSGGSTTTSEPKSYSDL
VRGNRIGSGAGGTFYKVVHRPTSRVYALKIINGNHDDTVRRQICREIEILRDVNHPNVVK
CHEMFDHNGEIQVLLEFMDKGSLEGAHVWNEEHLSDLSRQILKGLAYLHGRHVVHRAITP
SNLLINSNNNVKIADFGVSRVLAQTLSSFGTLAYMSPETFNTDLNQGMYDGCAGDIWSFG
VSVLEFFLGRFPFDVSRQGDWASLICAICMSQPPKAPATASPEFRHFVSCCLQREPARRL
TALELLQHPFVRRGASQSQNLRQLLPPPH
>FGENESH:[mRNA] 4 4 exon (s) 8748 - 11501 1629 bp, chain -
ATGGCTCCACAAGAAGACGCCATGCAGAAACAGAGCAGCGACAACAGTGACGTCATTTTC
CGATCAAAGCTCCCGGATATTTACATTCCGAACCACCTCCCTCTCCACGACTACATCTTC
CAAAACATCTCCGAGTTCGCCTCGAAGCCTTGCCTAATCAACGGACCCACCGGCCACGTG
TACACTTACTCCGACGTCCACGTCGCTTCCCGTCGAATCGCCGCCGGTTTTCAGAAACTC
GGCGTCAACCGAAACGACGTCGTGATGATCCTCCTCTCGAACTGCCCCGAGTTCGTCCTC
TCTTTCCTCGCCGCCTCCTTCTGCGGCGCAACCGCCACCGCCGCCAACCCGTTTTTCACT
CCGGCGGAGATCGCGAAGCAGGCGAAAGCCTCGAACTCGAAGCTCATCGTCACCGAGTCT
CGCTACGTCGACAAAATCAAAGACCTCCAAAACGACGGCGTCGTAATCGTCTGTACCGAC
GAGGAACCCTCCCCGATCCCGGAAGGCTGCCTCCGATTCGCCGAGTTGACTCAGTCAACG
GAGATCGAAACGGTTGAGATTTCTTCCGACGACGTGGTGGCGCTTCCTTACTCCTCCGGC
ACGACGGGTCTTCCCAAAGGAGTGATGCTGACTCACAAGGGGCTGGTCACGAGCGTCGCT
CAGCAGGTCGACGGCGATAACCCGAACCTCTACTTCCACAGCGATGACGTCATACTCTGC
GTTTTGCCCATGTTCCATATCTACGCTCTGAACTCGATCATGCTGTGTGGGCTTAGAGTC
GGTGCCTCCATCTTGATCATGCCCAAGTTCGAGATTAATCTGCTTTTGGAGCTGATACAG
AGGTGTAAAGTGACGGTGGCTCCGATGGTTCCGCCGATCGTCTTGGCGATTGCGAAGTCG
CCGGAGACGGAGAAGTATGACTTGAGTTCGATTAGAGTCATGAAATCTGGTGCCGCCCCG
CTTGGTAAGGAGCTTGAAGATGCCGTCAGTGCCAAGTTTCCTAACGCCAAACTCGGTCAG
GGATACGGAATGACGGAAGCAGGTCCGGTGCTAGCAATGTCGTTAGGGTTCGCAAAAGAG
CCGTTTCCGGTGAAGTCAGGAGCTTGTGGTACTGTGGTGAGAAACGCCGAGATGAAAATC
ATCGATCCAGACACCGGAGACTCGCTTTCCAAAAACAAACCTGGAGAGATTTGCATCCGT
GGTCACCAGATCATGAAAGGTTACCTCAACAACCCGGCTGCTACATCAGAGACAATAGAC
AAAGAAGGTTGGCTTCACACGGGAGATATCGGGTTGATTGATGACGATGACGAGCTTTTT
ATCGTCGATAGATTGAAAGAGCTTATCAAGTACAAAGGTTTTCAAGTGGCTCCGGCTGAG
CTAGAGGCTCTCCTCATCGGTCATCAGGATATCACTGACGTCGCCGTCGTCGCTTCCGTT
GCATTCGTTGTGAAATCCAAGGATTCAGAGTTATCAGAAGATGATGTGAAACAATTCGTG
GCGAAACAGGTTGTGTTTTACAAGAGAATCAACAAAGTGTTCTTCGTTGAGTCCATTCCT
AAAGCTCCATCAGGGAAGATATTGAGGAAAGATCTGAGGGCAAAACTAGCAAATGGGTTG

GTGAACTAA
>FGENESH: 4 4 exon (s) 8748 - 11501 542 aa, chain -
MAPQEDAMQKQSSDNSDVIFRSKLPDIYIPNHLPLHDYIFQNISEFASKPCLINGPTGHV
YTYSDVHVASRRIAAGFQKLGVNRNDVVMILLSNCPEFVLSFLAASFCGATATAANPFFT
PAEIAKQAKASNSKLIVTESRYVDKIKDLQNDGVVIVCTDEEPSPIPEGCLRFAELTQST
EIETVEISSDDVVALPYSSGTTGLPKGVMLTHKGLVTSVAQQVDGDNPNLYFHSDDVILC
VLPMFHIYALNSIMLCGLRVGASILIMPKFEINLLLELIQRCKVTVAPMVPPIVLAIAKS
PETEKYDLSSIRVMKSGAAPLGKELEDAVSAKFPNAKLGQGYGMTEAGPVLAMSLGFAKE
PFPVKSGACGTVVRNAEMKIIDPDTGDSLSKNKPGEICIRGHQIMKGYLNNPAATSETID
KEGWLHTGDIGLIDDDDELFIVDRLKELIKYKGFQVAPAELEALLIGHQDITDVAVVASV
AFVVKSKDSELSEDDVKQFVAKQVVFYKRINKVFFVESIPKAPSGKILRKDLRAKLANGL
VN
>FGENESH:[mRNA] 5 1 exon (s) 13999 - 14226 228 bp, chain +
ATGGCTTGTCTATTACATCAAGGCGCCAATGACACTTGGGTAAGGGACTTTTCCGGACCA
AGGACATATTCCTTGTCCTTCTTGGGGCCAAAGGATCTGTGTCCAAGTCAAAGGTCCTCA
CGACCATGTCCTTCTTGGGACTTACAATGGGTTTGTGTCTGGAACAAGGGACGTTATGGG
ACAACTCAACTCAATGGGCGAGTTTTGGTCCATGTTAAAACCGTTTGA
>FGENESH: 5 1 exon (s) 13999 - 14226 75 aa, chain +
MACLLHQGANDTWVRDFSGPRTYSLSFLGPKDLCPSQRSSRPCPSWDLQWVCVWNKGRYG
TTQLNGRVLVHVKTV
>FGENESH:[mRNA] 6 1 exon (s) 14788 - 15045 258 bp, chain -
ATGGACATAAGGCCGGTCGGCTCTAAGGTACCAAACACTGAGGCTTGGGACGCCAAGCTT
CAGGGTACTAATGGTCCTGATAATAATGAGATTGTAATCGATTATGTCATGTCTGGAACA
CATTGGAACAGAAAAGATGTCGACATAGATGATTTATTTGCATACAAGGTAGCACTTGAA
TTTATGGATATAAACGAGGATCATGAACCCACGTCAATAAAAGAGTGCGCACTCGTAGAT
CAGATTGGATTAAAATGA
>FGENESH: 6 1 exon (s) 14788 - 15045 85 aa, chain -
MDIRPVGSKVPNTEAWDAKLQGTNGPDNNEIVIDYVMSGTHWNRKDVDIDDLFAYKVALE
FMDINEDHEPTSIKECALVDQIGLK
>FGENESH:[mRNA] 7 1 exon (s) 16454 - 17461 1008 bp, chain -
ATGTCGAAAATCAACAACTTGGAATATGTTGCACTAAATCTCTCTGGAGATAATTACTTA
CAATGGACACTTGATACTAAGATCATCCTGAAATCCAAGGGGCTCGGTGAAAGTATTACC
GAGAACAATAATGTCACTGAGAAAGATAGATACGGAGCTATCCTAATTATTCGCCATCAT
CTGGCTGAGAGTCTCAAAGATCAGTATTTGACAAATGAGAATCCCTTAGACCTTTGGACA
GAGCTGCAAAGCATATATGATCACCAAAGAACGGTGATTTTACCAAAGGCTATGTATGAT
TGGAGGAATCTCAGAATCCAGGACTTCAAGTCCGTGGATGAGTATAACTCAGCCCTATTC
AAAATCGTTTCAAAGTTGAAATTGTGTGGTGAGGAGATAACGGATAAGGTTATGCTAGAA
AAGATATTTTCCACCTTCCACACAAGCAATGTGTTGTTACAACAACAATACCGTGAGAAG
GGCTTCAAGACATATGCAAATCTGATCTTATGTCTATTGCTAGCTGAGCAAAACAATGAG
TTATTAATGAGAAACAGTGAATTGAGACCTCTTGGATCAACCCCATTACCTGAAGCCAAT
AAGGCCGTAGAGGAAAAGAAAGAATCCAAAGAAAGTAACCACGTCTATCATGATAAACCA
CACGGCGTGGCGTGGTGGATGGAAGGGCGTGGCCGTGTAATACAACTCATATGGCCGAGA
ATAGCTATGGCGAGGCCTTGGATATCAATCCCATTCAGGCCGTGGTCGAGAAGCGATGGT
ATTTCTAAACCACAACACTCGACCAAATCACTGTGCCATAGGTGTGGAATGGATAACCAT
TGGGCTAAGATATGTAGGACTCCCAGACATCTTTGTGACCTCTATCAAGAGAGTCAGAAA
GGGAAGAATCCAGAAGCCCATATGGTCCATCATGATGGGGAAGATGATTTTGATCATGAA
CAAGATGACCTTATGGAATATGAAACATCTGATTGTCTAAAAGAATAA
>FGENESH: 7 1 exon (s) 16454 - 17461 335 aa, chain -

MSKINNLEYVALNLSGDNYLQWTLDTKIILKSKGLGESITENNNVTEKDRYGAILIIRHH
LAESLKDQYLTNENPLDLWTELQSIYDHQRTVILPKAMYDWRNLRIQDFKSVDEYNSALF
KIVSKLKLCGEEITDKVMLEKIFSTFHTSNVLLQQQYREKGFKTYANLILCLLLAEQNNE
LLMRNSELRPLGSTPLPEANKAVEEKKESKESNHVYHDKPHGVAWWMEGRGRVIQLIWPR
IAMARPWISIPFRPWSRSDGISKPQHSTKSLCHRCGMDNHWAKICRTPRHLCDLYQESQK
GKNPEAHMVHHDGEDDFDHEQDDLMEYETSDCLKE
>FGENESH:[mRNA] 8 2 exon (s) 17578 - 18334 234 bp, chain +
ATGATGCATTCAGTTCATACGGGTATGATGCAAGAACAAGCCGCACGGCCAAGACATGGG
TTCATAGAGAATTTACCTTATCCTTCGATTGGGTTGAATGGACAAGATCAACAAGAGGAG
ATCTTCGAGCTGATCGTCGCTGAGATCGTCGGGTCGCGAATGGGTTTCTATCGGATCGCG
AACGGACTGAAGCTGAGATGGGAACAAGCTGCTATTGAGTCGCGAACGAACTGA
>FGENESH: 8 2 exon (s) 17578 - 18334 77 aa, chain +
MMHSVHTGMMQEQAARPRHGFIENLPYPSIGLNGQDQQEEIFELIVAEIVGSRMGFYRIA
NGLKLRWEQAAIESRTN
>FGENESH:[mRNA] 9 3 exon (s) 20235 - 23494 3018 bp, chain -
ATGGGCTTTGGGATCTCAAACACTATGGGAACCACCTCTCTTGGCGAGGTTCTCGCTACT
CCCACTTTATCCGGTCTCGGCTTGATCGCTCTATGGCTAACTGCTCATGGGCTGAGAAGT
TCCCAATGGGAAGGTGCTGCTATTTGCGGTTCGAGGGATCCGACCACAGACCCCTCATCA
CTTACTTTAATTCTCCCTCAGCCAGGAAAAGTGGTTCCTTCAGATTCAATCGCTCTCTTA
CGGAAGTTCCTGAAGTTTCAGAGCTGGTTGATGCTGCATGGAACCATTATCCACTGGCCT
CAGTTATCGATAAGCTCAATGAGTGCCAAAGAAGGATCATTCTCTGGACAAAGGAGCGCT
TCAAAAGCAAACCTGCTGATCGCGGCCACTCAAAAGGCTCTCGACGAGCCACTTTCTGCG
CCAGTACCTGATCAGAACCGGATTGACTCTCTAACAAAAACTCTCAGTGCTGCGTATAAG
GAAGAAGAACAGTACTGGAGCCAACGGAGCCGGATTCAATGGCTCAAGAACGGGGATAGG
AATACGGGCTTTTTCCACGCGGCGACAAGGACCCGACGTATGCAAAATGCTTTGGCCGTC
ATAGAAGATGCTCAGGGATCGGAATTCTACGAAGACTCAGAGATAGCAGCCGTCATCTCC
AACTATTTCCAGGAGATTTTCTCTTCGACAAGTGGGGGAAATCTGGACCTGGTGTCTCAA
TCATTACCCAGAAAGGTTACTGCAGAGATGAACGAGCTGCTAACAAGGATGCCAAGTCCG
CAAGAGATAAAGGAAGCGACATTCTCTATTAACAGCGGCAAAGCACCTGGCCCCGATGGC
TTCTCCTCCAAGTTCTACCAATCATACTGGCATATCATAGGAGAGGATGTGGTCAAGGAC
ATTCTCCGGTTCTTTGAGACGAGCATCCTACACCCGGCGAACGAAACCCACATCAGGCTC
ATTCCAAAGGATACATGCCCGCGAAAAGTGTCAGATTACCGCCCTATTGCGCTATGCAAC
ACCCACTACAAGATCATCGCCAAAATCCTTACGAGGAGACTCAAGCCACTACTCCCTTGT
CTTATATCTAACCGCAATCTGCCTTCGTTTCGGGACAACTTCGAGGCAAATAAGTATTGC
TCCATGGCTGTGAAAACGGACATGAGCAAAGCGTATGACCGGATCGAATGGGACTTCTTG
AGAGCGGTGTTGCTGCAACTCGGTTTCGACCCTATTTGGATTGGCTGGATCATGGCCTGT
GTGGAGACGGTGTCATACTCTTTTCTCATCAATGGTTCCCCTCAAGGATCAGTTACTCCC
TCTCGTGGTATACGACAAGGCGACCCTCTGTCACCATATCTTTTCATCCTATGCACGGAA
GTACTGTCCGCTCTCTGTAATAAAGCGCAGGACAATGGATCATTACCGGGAATTCGTGTT
TCTCGGGGTAGTCCTCCGGTTAATCATCTTCTCTTTGCGGATGATACTATGTTCTTCTGT
AGATCGAGTGTGGCGAGTGTAGCAGCCTTGACGAAAATCATTAAGAGCTACGAAGTGGTT
TCGGGACAAAACATCAACCTCTTGAAATCAGCGATCACCTTTTCAGCAAAGACGCCCGCT
GAGGTGAAAGAACGGGTCAAGAATGTGCTTGGTATCACTGCAGAAGGTGGCATCGGCAAG
TACCTTGGCCTTCCCGAGCTATTCGGTAGAAAGAAACGAGACATTTTTGCCTCCATCCTT
GATCGGATACGACAGAGAATAAGGAGCTGGTCCACTCGGTTCTTATCGGGGGCAGGAAAA
CAGGTTCTCCTTAGAGCAGTGCTGGCTGCTATGCCGAACTATGCTATGTCATGCTTTAAA
CTCCCCGCCTCCTTGTGTAAGCAGATCCAATCACTACTAACTCGGTTCTGGTGGGATTCT
AACCCGGAAAAGAGAAAGATGTGCTGGGTAGCTTGGTCTACACTAACCAAACCAAAGCAT

GCCGGGGGATTAGGATTCCGAGATGTGGAAAGCTTCAATGATGCGCTTTTGGCTAAGATT
GGATGGCGATTGATAAAGGAACCAAACTCCTTACTAGCCCAGGTACTGCTGGGGAAATAC
GCTCGAAACTCCACTTTCCTGACATGTTCTAGTCCATCTAACGCTTCACACGGATGGAGG
AGCATCTTAGAGGGACGAAGACTATTGCTAAAGGGGTCTGGCTGGGCGGTGGGCGATGGT
GAAAAGATAAGGGTCTGGAGCGACCCTTGGCTTTCATTATCCGACCAGAAAGTACCCATC
GGTCCTCCAACGGAGACAAATCACTCCCTCCGTGTCCGGGATCTCCTCTGCCCCCTCACC
AACAAATGGGAGATAGATAGCATCCGCCTCCACCTCCCTCAGTATGAGGAGATAATTCTG
AAACTCATAACGAGCTCATCTCAGGTGGACGACTTGGTGGTGTGGTTACATGATAAGACA
GGCTCTTACTCAACCAAATCGGGGTATGGCCTTGCGATTTCTCTAAAACGGAATGAGGCA
ACCCACCTACCAGGCTTTGATTGGATGAAGCAGATATGGAATTGTAAAACCTCGCCGAAG
TTAAAAGATTTTCTGTGGAAAGTGGCAAATAGGGCGATCCCGGTGAGTTCAAACCTTGAA
CGCCGCGGGTTCCCAGCTTTTGAGTGCAAACGATGTGGCGGAGTAGAGGATGCCCTCCAT
TTACTCCTGCACTGTCCCTTCGCCTCAAGACCAGAAGTTATACTCAAGTCTATCAAGGAT
GCAAGAGAGTGGCAAGATGCTCACACTGATGGTGATATACCATGTGCTACTCGCCCGGCT
CGTCTACACCAGCAGCGTGGTCCCCCTGGGCCTGTCTCTTTACAGGGCATATCCTGTCAC
GTTGACGCAGCATGGAACCCGACTTCGGAATCTGTGGCATCGGAGGTGTTTTCACGATCA
CAGTCCCACTCAACGCTCCCACTAACCGCCGACTATCAGCTTCTCACGCCGATTCGTCTC
GTCCGCCCTAATGGCTGA
>FGENESH: 9 3 exon (s) 20235 - 23494 1005 aa, chain -
MGFGISNTMGTTSLGEVLATPTLSGLGLIALWLTAHGLRSSQWEGAAICGSRDPTTDPSS
LTLILPQPGKVVPSDSIALLRKFLKFQSWLMLHGTIIHWPQLSISSMSAKEGSFSGQRSA
SKANLLIAATQKALDEPLSAPVPDQNRIDSLTKTLSAAYKEEEQYWSQRSRIQWLKNGDR
NTGFFHAATRTRRMQNALAVIEDAQGSEFYEDSEIAAVISNYFQEIFSSTSGGNLDLVSQ
SLPRKVTAEMNELLTRMPSPQEIKEATFSINSGKAPGPDGFSSKFYQSYWHIIGEDVVKD
ILRFFETSILHPANETHIRLIPKDTCPRKVSDYRPIALCNTHYKIIAKILTRRLKPLLPC
LISNRNLPSFRDNFEANKYCSMAVKTDMSKAYDRIEWDFLRAVLLQLGFDPIWIGWIMAC
VETVSYSFLINGSPQGSVTPSRGIRQGDPLSPYLFILCTEVLSALCNKAQDNGSLPGIRV
SRGSPPVNHLLFADDTMFFCRSSVASVAALTKIIKSYEVVSGQNINLLKSAITFSAKTPA
EVKERVKNVLGITAEGGIGKYLGLPELFGRKKRDIFASILDRIRQRIRSWSTRFLSGAGK
QVLLRAVLAAMPNYAMSCFKLPASLCKQIQSLLTRFWWDSNPEKRKMCWVAWSTLTKPKH
AGGLGFRDVESFNDALLAKIGWRLIKEPNSLLAQVLLGKYARNSTFLTCSSPSNASHGWR
SILEGRRLLLKGSGWAVGDGEKIRVWSDPWLSLSDQKVPIGPPTETNHSLRVRDLLCPLT
NKWEIDSIRLHLPQYEEIILKLITSSSQVDDLVVWLHDKTGSYSTKSGYGLAISLKRNEA
THLPGFDWMKQIWNCKTSPKLKDFLWKVANRAIPVSSNLERRGFPAFECKRCGGVEDALH
LLLHCPFASRPEVILKSIKDAREWQDAHTDGDIPCATRPARLHQQRGPPGPVSLQGISCH
VDAAWNPTSESVASEVFSRSQSHSTLPLTADYQLLTPIRLVRPNG
>FGENESH:[mRNA] 10 2 exon (s) 26367 - 26637 204 bp, chain -
ATGTCAAATGAGCACCAACAGCGAAACCAAAGGAAAGGTGGTCTCGCAAGGCCAGTCCTC
AAAGCAAACACCGCAGCAGTAGGATATCCGGGATATCTGGATCTGAATCCGGATAGTAAA
ATCAAGGATCCGTCAAAACCAAATCCGGATCCTGATATCTTAATTTTTAGGTCTGGATAT
CCGGATCCGGATCTGTACTTTTAA
>FGENESH: 10 2 exon (s) 26367 - 26637 67 aa, chain -
MSNEHQQRNQRKGGLARPVLKANTAAVGYPGYLDLNPDSKIKDPSKPNPDPDILIFRSGY
PDPDLYF
>FGENESH:[mRNA] 11 17 exon (s) 30166 - 36702 1812 bp, chain -
ATGAAGGACGACGAGATGACGGCGGAGCCTTTCAAGCGGAACAGTGATGGCGGATCCGAT
GCCAGAAACGACGTCGGAGACGACGGCGGAGAAAATTTGAGGATGCTCTTTACGATTCTT
GAATTACATAAAAAAAATAAAACAGATTCTCGTCGTCGACGAGAATCTGTTTTATTTTTT

TTATTTGTGATTGACTCCACCAGCGGTATCAGAAGGCTTAGGGATACGCAACAAGTGGTA
TCAGAGCTTAAGTATTCTGCAACAAGTGATATCAGAGCCAGGTTCATTGATTCATGGATT
CCACGTTTCGAGGAGGAGAGACATATGAAGCTGGTGGTCACATGGTTCTACACACGTGGA
AATCTGATTAGGTTAAAAATCAGAGGATACGCCGAGATCAGTCACAAGGCGTGTGTGACA
CTAAGCATTGGAGTCACAGATTTCTTCATATTCTGCAAGTTATTGGAACTGAAAGCACCG
TGGAGCTCAAGGGTTTTTGCACTGGTTTTGTATGGGATTGTCAGAAGATTCAGTGAGGGT
TTTCGGATTCAATCTACAAGGGGACACCTTAGAACCTTGAATGAGATGAGTGTCTCATGG
CTGGTCGAGTATCAGATGGTATTCTTTAGCCAGCATGATGGGTGTTGCAGAAGGGTTGAA
GGAAACCGACTCATGCGGGTTCGTCGATGTGTGATTATGAGAGTCTACAATTGGGTAGAT
GTTCAGAGAACAAGATACTACAAGACTGCTATTTTAAGAGGGAATGCAGTGTGGTCATAT
GAGGGGAAACAAATGGGTCTACAGGGTATCACAGTTGCAGAGGTTGAGCTAAGCGGCGGG
TTCTTATGGCAACAACAGCGGTTCAGAGAGATGTTCAAGACAAGTTTCAAGGGTGATGAA
CCAGTGATTGTGCTGAAAGATAGGCATCGTCAAGGAAAGAACCAGATGGCGTTTATTTGT
GAAATCATGATGAGAGACTTGCAAGGGATGCAGCGGTTGTTTGCAAGAAAGGGAAAACCC
GTGCTTAAGAAGATGGAATCAAAGGTCATCAATCTCACAGAGACTGAAGGTATTCATGGT
TCACAAGGAATGCAGGTTATATTTGCAAAAGATGGTCAACGGATCTTTCAGCTGCACAGT
GGTCAAGGTGGAGCTCTCAGACAAGAATTGAAGCGCAAAGGCAAAAGGAGATATTTGCAT
ACTTGGCCAGGTAGGATCATGCTTGAACAGCTGAAGGGTAAAAGTTTCGGTAAGCAACTT
GATCAGCTCAAAATAGAATACTCTGCAGTGAGGTTGATACAGAGGAACAGAGAGCCGAGT
ATGATGCAGGGTCGTGGTGATGTACTCATCAGGCTAATGGCGGATTCAATGATTAACAGG
CGTGTTCAAGTCGTTGACGGTGAAGCACAAGTTTCGATATTGGAGACACCTCAATACATG
AAGGCAGCTCGTGAAGGAGAGCCAAGGCGTACCAAGAAGTATATGCGGTACAAAGTGATG
CTCAAGTACAGTCTGTTCAAGAACATCAAGGGTAAGTCAAAGACTATTTCAGAGATATGG
AGCATGAGTGACTTCGAGGTTTGTGTCGAAAGTCTGTTCAGCAAGTGTTTCTGGATGGCG
TGTTCTATTCGAAATAATGGCGGTAAAAAAGGTGTTCAAGGCTGGAGTAGTGGAGAATCA
GGGATTGTTTCAGAACTGAGGCATGACAGAGAGAGTAATGATCTTGTAAACCACATCGGA
GGTGTTTTTCGATACTTCATAGTAGATGTTGTGGATCCTGGATCCACCCCACATGCAGCG
GTGCGGCCGTGA
>FGENESH: 11 17 exon (s) 30166 - 36702 603 aa, chain -
MKDDEMTAEPFKRNSDGGSDARNDVGDDGGENLRMLFTILELHKKNKTDSRRRRESVLFF
LFVIDSTSGIRRLRDTQQVVSELKYSATSDIRARFIDSWIPRFEEERHMKLVVTWFYTRG
NLIRLKIRGYAEISHKACVTLSIGVTDFFIFCKLLELKAPWSSRVFALVLYGIVRRFSEG
FRIQSTRGHLRTLNEMSVSWLVEYQMVFFSQHDGCCRRVEGNRLMRVRRCVIMRVYNWVD
VQRTRYYKTAILRGNAVWSYEGKQMGLQGITVAEVELSGGFLWQQQRFREMFKTSFKGDE
PVIVLKDRHRQGKNQMAFICEIMMRDLQGMQRLFARKGKPVLKKMESKVINLTETEGIHG
SQGMQVIFAKDGQRIFQLHSGQGGALRQELKRKGKRRYLHTWPGRIMLEQLKGKSFGKQL
DQLKIEYSAVRLIQRNREPSMMQGRGDVLIRLMADSMINRRVQVVDGEAQVSILETPQYM
KAAREGEPRRTKKYMRYKVMLKYSLFKNIKGKSKTISEIWSMSDFEVCVESLFSKCFWMA
CSIRNNGGKKGVQGWSSGESGIVSELRHDRESNDLVNHIGGVFRYFIVDVVDPGSTPHAA
VRP
>FGENESH:[mRNA] 12 2 exon (s) 38087 - 39382 963 bp, chain +
ATGGTCCTTGACCTAGGACGGGACAACCTCGGCACGGGTCGTCCTCGCCCATGGGCGAGG
CGGCCACGGTGCGGGTCATCCTCGCCCGTGGGCAAGGCGACCTCGGCAGGGGCCATCCTC
ACCAGTGGGCGAGAACACCTCGGCACGGGTCGTCCTCCCCCGTGGGCGAGGCGGCCTCGG
TGCTGGTCGTCCTCGCCCGTGGGCGAGGTGGCCTCGGTGCGGGCCGTCCTCGCCCGTGGG
CGGCGGCCTCGGTGCTGGTCGCCCTCGCCCGTGGGCGAGGTGGCCTCGGTGCGGGCCGTC
CTCGCCCGTGGGCGAGGCGACCTTGGTGCAAGTCGTCCTCGTCCGTGGGCGAGGCGGCCT
TGGCACGGGCCGTCCTCGCCCATGGGCAAGGTGGCCTCGGTGCGGGTCGTCCTCGCCCGT

GGGCGAGGTGACCTTGGCACGGGCCGTCCTCGCCCGTGGGCGAGAACACCTCGGCACGGG
TCGCCCTCGCCTGCGGGCGCAGGCGGCTTGGTGCGGGTCGCCCCGCCCATGGGCAAGGTG
GCCTCGGTGCTGGTCGCCCTCGCCTGCGGGCGCAGGTGGCTCGTGTCCGGTCGGTCCAAT
CCATACTTGCTTATGTTCGAGACCTCATCCGGACTGAATGAATTCTCGATGCTCTCTATG
AACAACATCCTCCCCGCGACCCCTAGGGACCTGTACGCTAAGCGCGATCTTCTCCGCAAC
AGTCCGTTTTTTTGGGACTCCTTCACCCTAGACAGGATCCGGAACGCAGTGGCGCTTCAT
CGATCGCTAGAAATCCCCCGACCCTTTGGTGCGCCGGATAATGACGAGCCAAATCCTGAC
GTCCTCCCGTTCGGAGGGAGAGAGTTAGGCCTCGGAAGGATAAGGGAGTTGCTTTTGACG
ATGGAAACTTCATCTCGGAGGACCTTCCGCTACCGGATAGACTCCTGGGTTCGCCCCGGG
TGA
>FGENESH: 12 2 exon (s) 38087 - 39382 320 aa, chain +
MVLDLGRDNLGTGRPRPWARRPRCGSSSPVGKATSAGAILTSGREHLGTGRPPPWARRPR
CWSSSPVGEVASVRAVLARGRRPRCWSPSPVGEVASVRAVLARGRGDLGASRPRPWARRP
WHGPSSPMGKVASVRVVLARGRGDLGTGRPRPWARTPRHGSPSPAGAGGLVRVAPPMGKV
ASVLVALACGRRWLVSGRSNPYLLMFETSSGLNEFSMLSMNNILPATPRDLYAKRDLLRN
SPFFWDSFTLDRIRNAVALHRSLEIPRPFGAPDNDEPNPDVLPFGGRELGLGRIRELLLT
METSSRRTFRYRIDSWVRPG
>FGENESH:[mRNA] 13 5 exon (s) 39847 - 43083 2565 bp, chain -
ATGGCTACTTCTTCTGACCTTGCACCGGAGATTCCGAGTGAGTTGCTAGATGTCTTGCAG
GACTTTTCTGATGTCTTTCCAGATGAGAATCCACAGGGATTGCCACCAATACGAGGGATT
GAGCATCAGGTAGACTTTGTTCCGGTGCATCTCGCCGAACCGACCAGCTTATCGAACCAA
TCCGGTCGAGACCAAGGAACTTCAGAAACAAATCGTACTTTCATGCGCTTAATGAATCAT
ATTTTGAGAGCATTCATTGGTCATTTCGTGGTTGTTTACTTTGATGACATTCTCATTTAC
GTAAAGAACCTAGATGATCATAAAAAGCATTTGAAATCTGTTCTTGAAGTTCTTAGGAAA
GAAAAGCTGTTTGCAAATCTTAGTAAATGCTCTTTTGGAACAGATCACGTGGTGTTCTTA
GGTTTGGTTGTAGGTGCTGATGGACTCAGGGTGGACGAGGAGAAGATCAAGGCCATCCGA
GATTGGCCAAGTCCCACCACAGTGGGAAGTAAGGAGTTTCCACGGCCTTGCGGTTTCTAC
CGTCGGTTCATCCAGGATTTCAGTACCATTGCAGCCCCACTTACTGAAGTGATCAAGAAG
AATGTTGGTTTCAAGTGGGAACATGATCAGGAAGAAGCATTCCAAGTCTTAAAAGGGAAG
TTAACTCATGCTCCTTTACTTGTTCTTCCTGATTTCTCCAAGACTTTTGAGATCGAGTGT
GATGCTTCTGGTGTTGGTATTGGTGCTGTTTTGATGCAGGAAAAGAAGCCCATTGCTTAT
TTCAGTGAGAAGCTTGGTGGAGCCACGCCAACTACCCCACTTACGATAAAGAGCTGTATG
CTTTGGTATGCACTTCTCTCAGCTCTTGAAACCAAGTTGCTCGGTTTTGAATTTATCAAA
GATTTATATGCAACTGATCCGGATTTCAAAGAGATTTTCAAAAAGTGTTCCAAAGCGGCT
TATGGGAAGTATTATCAAGATTCTGGATTTTTATTCTTTGATAACCGCTTGTGTGTACCC
CAATGTTCTTTGAGGGAGTTGTTTCTCAGGGAAGCACATGGAGGAGGCCTTATGGGACAT
TTCGGAGTCAAGAAGACACACAAGGTCGTTCATGAGCATTTCGTTTGGCCGAGTTTGATG
AAAGACGTGGAGCGGATCTGTGGCCGATGTGTGGTGTGCAAGAAAGCCAAGTCTAAGGCA
CTGAACCAAGGTTTGTACTCGGCCCTACCCATTCCATCTCATCCTTGGGTTGATATTTCG
ATGGACTTTGTGCTTGGATTGCCTAGGTCTAGATCTGGCCGAGATTCTATCTTTGTGGTT
GTTGATAGGTTCTCTAAGATGGCTCATTTCATACCTTGTCATAAGACTGATGATGCTGTG
CATATTGCTGATTTATTCTTTAGAGAAATTGTTAGGTTGCACGGCATGCCTAAGACCGTT
GTTTCTGATCGTGATGCTAAGTTCCTTAGTTATTTCTGGAAAACTTTGTGGTCTAAGTTA
GGAACGAGGTTGATGTTTTCCACCACTTGTCACCCACAGACCGATGGTCAAACTGAGAAT
TTGCTTATAATCATTCTTCGTGATTCTTCTACTAAGTTTTCTCCATTTGAGGTTGTTTAT
GGTTTCAATCCCTTATCTCCACTTGATCTTTTGCCTTTGCCTTTGAGTGAAAGAGTTTGT
ACAGATGGCAAGAGGAAGGCTGACACCATTCAGAAGCTACATGAGACAGTTCGTGCCAAC
ATCTCAGCCAGAACCGAGGGCTTCACAAGAAGGGCAAACAAGAAAAGAACCAAAGTTGTC

TTTGAGGAGGGCGATCTTGTTTGGGTTCATCTAAGGAAGGAGCGGTTCCCTGACGAAAGA
AAGTCCAAACTCATGCCAAGGGTCGATGGTCCCTTTCAAATCCTTAAGAAGATCCATGAC
AATGCCTATCAGCTTGATCTTCAAGATGATCCGGATTTGTGGACAAATCCTTTTGAAGAG
GGAGGGAATGATGCACCTCAGGACGTGGACCAGGACAAGACACAGCCCCAGCATGGTGAT
CAGGACGACCACATCAGTTCAGCCGAGGTTCATCCATCCGGCCGTACCAGACAGACAGAC
CGAGCCGTGTACCGAATCGACCCGCGTACACCCGGGAAGGACCTTAAGCTGGATCCACGA
CCAGATGACCAAACTGATCAACCCACACGCCTTCTTTCCCGTCCTACTCGCCAATCTAAG
ACCAACAGTCAAGCCAGAACCCACTTTGATCGAGAGGAACCCGATTCAGATCACAGCCTA
TCTCTTTTGGCCCGTTTGGTTCGTACCGCGCACCCGGACGGACATGCTCTTGATCTGATC
GACCAGTTTGATTCATTCATGGGATTTGATGACTCCAATCCATCTAAGGCAAGGATTCTT
AAGCTCTCTGAAGATTTGGATCAAGTGAAATCTCCATCCGTTCATGAAGGTTCGACGATC
ACCCCCGCGGACAGCCCATCGTGTGTCCTTCTCCTTACTGCGATGGAACCGATCATCACG
GACGCACCTAGACACTCCGATGTATCTGGACCAGTCAACATCTGA
>FGENESH: 13 5 exon (s) 39847 - 43083 854 aa, chain -
MATSSDLAPEIPSELLDVLQDFSDVFPDENPQGLPPIRGIEHQVDFVPVHLAEPTSLSNQ
SGRDQGTSETNRTFMRLMNHILRAFIGHFVVVYFDDILIYVKNLDDHKKHLKSVLEVLRK
EKLFANLSKCSFGTDHVVFLGLVVGADGLRVDEEKIKAIRDWPSPTTVGSKEFPRPCGFY
RRFIQDFSTIAAPLTEVIKKNVGFKWEHDQEEAFQVLKGKLTHAPLLVLPDFSKTFEIEC
DASGVGIGAVLMQEKKPIAYFSEKLGGATPTTPLTIKSCMLWYALLSALETKLLGFEFIK
DLYATDPDFKEIFKKCSKAAYGKYYQDSGFLFFDNRLCVPQCSLRELFLREAHGGGLMGH
FGVKKTHKVVHEHFVWPSLMKDVERICGRCVVCKKAKSKALNQGLYSALPIPSHPWVDIS
MDFVLGLPRSRSGRDSIFVVVDRFSKMAHFIPCHKTDDAVHIADLFFREIVRLHGMPKTV
VSDRDAKFLSYFWKTLWSKLGTRLMFSTTCHPQTDGQTENLLIIILRDSSTKFSPFEVVY
GFNPLSPLDLLPLPLSERVCTDGKRKADTIQKLHETVRANISARTEGFTRRANKKRTKVV
FEEGDLVWVHLRKERFPDERKSKLMPRVDGPFQILKKIHDNAYQLDLQDDPDLWTNPFEE
GGNDAPQDVDQDKTQPQHGDQDDHISSAEVHPSGRTRQTDRAVYRIDPRTPGKDLKLDPR
PDDQTDQPTRLLSRPTRQSKTNSQARTHFDREEPDSDHSLSLLARLVRTAHPDGHALDLI
DQFDSFMGFDDSNPSKARILKLSEDLDQVKSPSVHEGSTITPADSPSCVLLLTAMEPIIT
DAPRHSDVSGPVNI
>FGENESH:[mRNA] 14 1 exon (s) 43202 - 45055 1854 bp, chain -
ATGAGCAGTGAGGATGAAAGGAGCCGGCCAGAAAACTCTTTTGCTGGTTTATCTAACTTG
CAGATGCGAGCTTTGAATGACAATATTACTAATCTTATGAATTCAGGTTGGGAACAGATC
CATCAACGGCTTGATGCAATGGAAGCTAGCCAGCCGACCCAAACCAGAACCGGAGCACGG
AGAGATCGTCCAAGGAGGAACAACCGTTCAGATGATGATATCCAAGAGGAGGACTTCCAA
GAGGACGATGATCGATCCATCAACCGACCTAGGAGAAGTCACAGAAACCGAACTCAAGGT
GATATCAATCCTTTTGCTAGGGAAGATCGTGCTGATAATGGTTTAGGAGGATTGAAACTG
AAAATTCCACCTTTTGAGGGTAAAAATGATCCTGATGCTTTTCTTGAATGGGAAAGAAAA
ATTGAACTTGTCTTTGATTGTCAAAACTTCTCTGAACTTAAGAAAGTCAGATTGGTTGCA
ACTGAATTTTCTGGCTATGCTATTAATTGGTATGATCAAGTTGTGACTAGCAGGAGAAGG
AACGGTGGCCGGCCAATAGCCTCATGGGACGAGCTCACAACCTTGATGCGGAGAAGGTTC
GTTCCGGAACACTACCATCGTGACCTTCACCAAAGGTTGAGACGTTTGCTTCAAGGAACT
AAGTCCGTGGAAGATTATTTCCAAGAAATGGAGACTTTGATGATCAAAGCCGATGTAGAC
GAGACCTTGGAAGCAACCATGGCCCGGTTCCTAGCTGGACTCAACCGAGACATCCAAGAC
CCTATGGAGCTTCAAGACTACGAAAGCCTGAACCAGATGCTTCACAAAGCAATTCTGATC
GAACAGCAAACCAAGAGGAAGAGCTACTCCAAGCCGGCCTTTGCTCCCAAACCGGCCTTT
GCGCCCAAACCAAGCTATCAAGACAAGGGTAAGTCTTCTTCCACAACAAATAATGCTTTT
AAGACTGATGTCCCTGCTCGTTTTGACAAAGGAAAAGCAGTTGAGACTCCTAGCCGGGCA
AGAGACATCAGGTGCTTCAAGTGTCAAGGTCTAGGACACTATTCCAACAAATGTCCTAAC

CAAAGGGTGATGATTCTCATGGAGAATGGTGAGGTCGAGTCCGAGGATGAAAAGGAGGAT
GAAGAGGATTCTGGTCCAGTCTTTGATGATGATGAAGAACCATTCGATTACCCACATCAT
GGACCACTTCTCGTTACTAGGAAGCCTTTGGACGATCCACTCGGTCCCATCTTCGATAAG
GAGGACGACCACTCGGTCGATGATTCTGGTCCGAACTTTGATGCTGATTCCGGTCCTATC
TTTGATGAGGAGAACATCTACGACTATCCAGCTCACGGACCACTACTTGTCACTAGGAGA
TCCTTGAGTGTTCAACCCAAAACCAATGAAAAAGAACAAAGGGAGAATCTCTTTCATTCT
CGTTGTTTAATTTCTGAAAAAGTATGTTCTTTAATCATTGATGGTGGGAGTTGTACTAAC
GTTGCTAGTGACAATCTTGTCAGGAAACTTGGACTTGTCACTAGGCCCCTCTCTCGTCCT
TTCAGGTTGGAATGGCTAAATGAGGCTGGTGAGCAGTATGTGAAGGAACAGGTCACGGTC
CCCCTTACCATTGGGCGATATGAAGATGAGGTTGTCTGTAATGTTCTTCCCATGGACGCA
TGCCACATTCTCTTGGGTCGGCCATGGCAGTTTGATAAGAAGGCCGTGCATGATGGCTTC
ACAAACCGACACTCCTTCGACCATAATGGCAAGAAGATCACGCTGGTCCCTTTGACACCT
TTGAGGTTCATCAAGATCAACTTCAGCTCAAGAAGAACCGTGAAAAGAGCCTAA
>FGENESH: 14 1 exon (s) 43202 - 45055 617 aa, chain -
MSSEDERSRPENSFAGLSNLQMRALNDNITNLMNSGWEQIHQRLDAMEASQPTQTRTGAR
RDRPRRNNRSDDDIQEEDFQEDDDRSINRPRRSHRNRTQGDINPFAREDRADNGLGGLKL
KIPPFEGKNDPDAFLEWERKIELVFDCQNFSELKKVRLVATEFSGYAINWYDQVVTSRRR
NGGRPIASWDELTTLMRRRFVPEHYHRDLHQRLRRLLQGTKSVEDYFQEMETLMIKADVD
ETLEATMARFLAGLNRDIQDPMELQDYESLNQMLHKAILIEQQTKRKSYSKPAFAPKPAF
APKPSYQDKGKSSSTTNNAFKTDVPARFDKGKAVETPSRARDIRCFKCQGLGHYSNKCPN
QRVMILMENGEVESEDEKEDEEDSGPVFDDDEEPFDYPHHGPLLVTRKPLDDPLGPIFDK
EDDHSVDDSGPNFDADSGPIFDEENIYDYPAHGPLLVTRRSLSVQPKTNEKEQRENLFHS
RCLISEKVCSLIIDGGSCTNVASDNLVRKLGLVTRPLSRPFRLEWLNEAGEQYVKEQVTV
PLTIGRYEDEVVCNVLPMDACHILLGRPWQFDKKAVHDGFTNRHSFDHNGKKITLVPLTP
LRFIKINFSSRRTVKRA
>FGENESH:[mRNA] 15 2 exon (s) 46108 - 47216 867 bp, chain +
ATGGAGATTTCACTTGACCCAAATCTTGAGAGCTTAAGAATCCTTGCCTTAGATGGATTG
GAGTCATCAAATCCCATGAATGAATCAAACTGGTCGATCAGATCAAGAGCATGTCCGTCG
GGTCTGGTACGAACCAAACGGGCCAAAGAGATAGGCTGTGATCTGAATCGGGTTCCTCTC
GATCAAAGTGCATCAAGTGGGACTAGCGAGGCTCCTCTTCTGGACGACTTCTTTGCTAAC
CTTCCTCCTAGTTTTACTACTCCAGCGTCGCTGGACGAAGTATCGAGGATAGCAGTGGTC
GCGGAAGGTTCTCGATTTATCAACGAGGTTTGGTTTGTAGTTTCCGTATTTGATCCTTGG
TTTTTGCAGGGGACGCATGTTTTCAGCGCGGCACTCGATGGAGGTTTCAGGGAGGCGCGT
CTTTCGCATTTCAAAGCCGAAGAGATTGAGAGGAAGTTCGTCCGTTTCCAGAATGAAGTC
GCGGAAAAAGATCGTAAGCGGGACGAATCTCATTCCCGAGCTCTCCTTCGTGCGGAGAGG
AAAGGGAGGAGGGCGGTTGCTGCTGAGCTAGCGAGGAGAGCCTCGTTGTTCGATGCTGAG
TTCAGGAGTTTCAAGGATGCCCAGGACTTCGTGGGTGCCGCGGCTCGGTTGGTACCGTTT
GGAAGTCATAGGACGCTAACTTTTCCTTTCTTTACGAGGTCACTGAGATGTCGAGCCTTA
TGGAAGGGTGTGCCCATGCTGAGTCGGCGGTTCCTCCGATCGAGAGGAGGATCCAGGAAC
TCTGGGATCCCATCGTGGTCTCGGAGGACACGGCAGAAGCGGAAGAGGGTGCCACGGACG
AGGAAGGAGAAGTCGACCAGCCCGTGA
>FGENESH: 15 2 exon (s) 46108 - 47216 288 aa, chain +
MEISLDPNLESLRILALDGLESSNPMNESNWSIRSRACPSGLVRTKRAKEIGCDLNRVPL
DQSASSGTSEAPLLDDFFANLPPSFTTPASLDEVSRIAVVAEGSRFINEVWFVVSVFDPW
FLQGTHVFSAALDGGFREARLSHFKAEEIERKFVRFQNEVAEKDRKRDESHSRALLRAER
KGRRAVAAELARRASLFDAEFRSFKDAQDFVGAAARLVPFGSHRTLTFPFFTRSLRCRAL
WKGVPMLSRRFLRSRGGSRNSGIPSWSRRTRQKRKRVPRTRKEKSTSP
>FGENESH:[mRNA] 16 4 exon (s) 48688 - 50912 1980 bp, chain -

ATGACAACCGACGCTGACAAAGACCAGCAAACGCACGACGGCGCTCCCATTGATGCCAAC
GCTGATAGAACTCCAGCTGGAAACGTATCGACGGTCACCACTGACACCGTGATACTAGAC
CAGATGAAGGAATTGTTCGCCTCCGCTCAGAAAAAATTGGACGAACAAGGAAAACTCGTG
GCCTCTCTTGCAAAACAGTTGGAAACCTTAACGGCGAAGGCCAAGAGCAAAGCCCCGCGC
GGGGCCACAAGAGCCCGCAGCGGTAGAAGACCCGATTTCGAGACTCCGGGCGATCGAGCT
GCACGTGCGGACAAAACTTCTTCAGGCCAAAACCCTGACGAAACGCTCCCGCTATGTGCA
CAGCCGACTAAGGAGAACCTTCCACCTCCCGCCGGGAGAAACAAAGAGGACAATATTGAG
CAGATCGATCTAGACATAAGCGACCAGTCCGACCGCTCGGACGAAGGCGCTGACGAAGAA
CTGGCCGAGAATCAGGCTCGGATCCACCGCAACCAGCGCCGGCAGGCTCGGAAGGCCACC
GCAAACCTTGACGAGATCCATGATCTCTGCGAGTATATCGCAAAAACCGCGGCAGAGGTC
AAAGCGGTAAAATTGCAAATCCACCACGCGACGAGCGCTGCGCCCGAGATCGACAGACTT
CTCGAGGAAGCACGCAAAACTCTGTTCACTGCCCGGATTACGGAAACTAACATCTCAGAT
CTAGGGAAGATCAAAATTTCCGTCTACAATGGCACCACCGACCCGAAGGCGCATCTGCAG
TCTTTCCAGATTGCGATGAGAAGGGGCAAACTCAAGGAACGTGAACGAGACGCTGGCTAC
TGCCTCCTCTTTGTCGAGAACCTCAAAAAGCCACGCTCGAGTGAAACCTCAGACATTGAC
CTACGGAGTCTATCTCAAAGAGAAGACGAACCACTCCGCGAGTTCATGAATAGGTTCAAG
CTAGTAATGGCAAGAGTAACCGGGATCAGCGACAAATTGGCAATCGATGCTCTGCGGAAA
ACTCTCTGGTATAGGTCGAAACTCCGACAATGGATATCTCTCGAAAAACCGAGAACAATC
CAGGATGCCCTTCACAAGGCAACGAAATTCATCAACATGGAAGAGGAAATGAAAGTCCTC
TCCCAGAAGTACAACCCGCAAAAAGCATCCACCAGAGGGAAAAGCTCTCGTAACAACAAG
TATGTCCACCACAAGGGGGAAGATGTCCAGGGCGAACACAACTACGCCATCAACTCCGAA
CAAGGGAAAACCTCGGGAAACACCTATACAAGGAACCTGTTCAAGGATAACTCCTACTGC
GAGTTCCACCAGACCAGAGGGCATTCCACCATGAACTGCAAGGTTCTCGGAGCAAGGCTC
GCTGCGAAGCTCCTTGCTGGCGAACTGTCAAAGGTCACGGGAATAAAAGACCTCCTTCTG
GAGTCCGACCGCCCTCCTAAACCTGACAAAGAATCTCCCAAGAGCAACGCAAACCAGTAC
GGCGGGAAACGCGGAAGAAGACAAGATGACCGTGGAAACGACAGCAACCGTCGCAGGGCA
TACGGAAGAAAGGCAGAAACGAGTTATAACTGGATGGCTCGGTCCCCGGCCGACGACACT
CCTAACGACACGATCGTTTTCGAAGAGCGGGAAACCATCAGAATCGATAAACCTCACTGT
GACCCGTTGGTCATCGATTTAGTGATCCGAGACCTCGAAGTAGGAAGGGTCCTCATTGAC
ACAGGCAGCACAGTCAACGTCATCTTCCGTGACACTCTCCGAAGAATAAACATCGAACTC
GGGGAAGTCGTCCTGGAACCAAAACCTTTGACTGGTTTCTCGGGCACAATGTTGATGACT
CTCGGATCGATCAAACTTCCGGTCATGGCTAAGGAAGTCACGAAAATCGTTGACTTCGCC
GTAGTCGATAACCCGGCCATCTATAATGCATCATGGGAACCCCCTGGATCAACGCCATGA
>FGENESH: 16 4 exon (s) 48688 - 50912 659 aa, chain -
MTTDADKDQQTHDGAPIDANADRTPAGNVSTVTTDTVILDQMKELFASAQKKLDEQGKLV
ASLAKQLETLTAKAKSKAPRGATRARSGRRPDFETPGDRAARADKTSSGQNPDETLPLCA
QPTKENLPPPAGRNKEDNIEQIDLDISDQSDRSDEGADEELAENQARIHRNQRRQARKAT
ANLDEIHDLCEYIAKTAAEVKAVKLQIHHATSAAPEIDRLLEEARKTLFTARITETNISD
LGKIKISVYNGTTDPKAHLQSFQIAMRRGKLKERERDAGYCLLFVENLKKPRSSETSDID
LRSLSQREDEPLREFMNRFKLVMARVTGISDKLAIDALRKTLWYRSKLRQWISLEKPRTI
QDALHKATKFINMEEEMKVLSQKYNPQKASTRGKSSRNNKYVHHKGEDVQGEHNYAINSE
QGKTSGNTYTRNLFKDNSYCEFHQTRGHSTMNCKVLGARLAAKLLAGELSKVTGIKDLLL
ESDRPPKPDKESPKSNANQYGGKRGRRQDDRGNDSNRRRAYGRKAETSYNWMARSPADDT
PNDTIVFEERETIRIDKPHCDPLVIDLVIRDLEVGRVLIDTGSTVNVIFRDTLRRINIEL
GEVVLEPKPLTGFSGTMLMTLGSIKLPVMAKEVTKIVDFAVVDNPAIYNASWEPPGSTP
>FGENESH:[mRNA] 17 2 exon (s) 51814 - 52379 507 bp, chain +
ATGGTACATGAGTGTTGTGTCCGTAGACCTGATGGTCGAGCTATGGGAACTTGGATGAGG
AGGTACGGTTTCGGGACAGACGGTCCTTGCCCTAGGACCGGAAGACCTCGGCACGGGTTG

TCCTCACCCGTGGGCGAGGCGACTTCGGCACGGGCCGTCCTCGTACGTGGGCAAGATGAC
CTCGGCACGGGCGACCTCGGCACGGACCGTCCTCGCCCGTGGGCGAGGTGGCCTCGGTGC
GGGTCGTCCTCTCCCGTGGGCAAGGGAACCTCGGCACGGGCCGTCCTCGCCCGTGGGCGA
GGCGGCCTCGGTGCGGTCTTCCTCGCTCGTGGGCGAGGTGACCTCGGCACGGGCCGTCCT
CGCCGGTGGGCGAGGTGGCTCGTGTCCCGTCGGTCCGATCCATACTTGCTTATGTCTGAG
ACCTCATCCGGACTGAATGAATTCTCGATGCTCTCTAGTTCGTGTCCGCGGAAAACCGTC
TTTTATTCGGTTAACCATTTCCCCTGA
>FGENESH: 17 2 exon (s) 51814 - 52379 168 aa, chain +
MVHECCVRRPDGRAMGTWMRRYGFGTDGPCPRTGRPRHGLSSPVGEATSARAVLVRGQDD
LGTGDLGTDRPRPWARWPRCGSSSPVGKGTSARAVLARGRGGLGAVFLARGRGDLGTGRP
RRWARWLVSRRSDPYLLMSETSSGLNEFSMLSSSCPRKTVFYSVNHFP
>FGENESH:[mRNA] 18 1 exon (s) 53682 - 53840 159 bp, chain -
ATGGCTATCTTAAATTGGAACTTAATCTTGAAGATGATGCTGATGAAGCTTGATGAATCT
AGAACTACTGATGATTATGTAGTCATGATCAAAGCTTTTGATCTTAATGCTGATGGAGTT
TTGTGTTTTGATGAGTTTGCACGTATGATGATGCGTTAG
>FGENESH: 18 1 exon (s) 53682 - 53840 52 aa, chain -
MAILNWNLILKMMLMKLDESRTTDDYVVMIKAFDLNADGVLCFDEFARMMMR
>FGENESH:[mRNA] 19 2 exon (s) 56927 - 57467 420 bp, chain -
ATGGCGAGAAGCAGAGGATTGAAGAGGAAGAGCAATACGGGGTGTTGCTGTATTACAAGG
TGTACCTCTGTTCCTGATCTAGATGAGCTCATTGCCTTCTATGAATCTAGCTGCAACTCA
CTCGGTGTACTCGGCCGAGCTTTTTACGGTGTGAAGAGCGTTGTGAAAGCTGAGATCAGA
GATATGTTGTCTTCAGGTAGAAGTATGATTCTCAAAGAGCCGGAGGCCGTTGATGCTAGT
GTGGAGAAGCTTTACCCGATGATACAAGATGGCGTTGATCATGTGGAAGTTGAATCTTTT
AAGGACTATGTAATGGAGTTGGGAACACAAGCTGATAAATTGTCTCAGGGGTTAGATCAG
TTACTAGAGGAAGTTGACAGTTTTTTCAAAATGACTTGGAAGAGATGTGTTGCTATGTAA
>FGENESH: 19 2 exon (s) 56927 - 57467 139 aa, chain -
MARSRGLKRKSNTGCCCITRCTSVPDLDELIAFYESSCNSLGVLGRAFYGVKSVVKAEIR
DMLSSGRSMILKEPEAVDASVEKLYPMIQDGVDHVEVESFKDYVMELGTQADKLSQGLDQ
LLEEVDSFFKMTWKRCVAM
