# Supplementary material for: Genome-Wide Identification Analysis of the 4-Coumarate: Coa Ligase (4CL) Gene Family in Brassica U’s Triangle Species and Its Potential Role in the Accumulation of Flavonoids in Brassica napus L
Source: Plants (Basel). 2025 Feb 26;14(5):714. doi: 10.3390/plants14050714 (PMC11902127; doi:10.3390/plants14050714)
Supplement: Supplementary file 1 [file plants-14-00714-s001.zip › Supplementary Materials/Figure S3/Bca4CL3.pdf]

FGENESH 2.6 Prediction of potential genes in Arabidopsis_thaliana genomic DNA
Seq name: ChrB06 6519804 6600708
Length of sequence: 80905
Number of predicted genes 28: in +chain 17, in -chain 11.
Number of predicted exons 88: in +chain 49, in -chain 39.
Positions of predicted genes and exons: Variant 1 from 1, Score:3253.056641

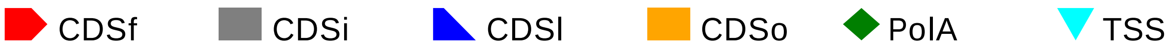

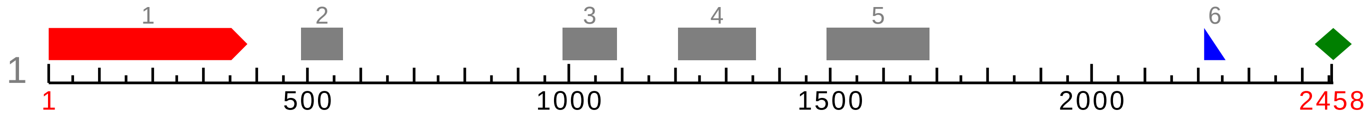

1	+	1	CDSf	1	-	381	62.09	1	-	381	381
1	+	2	CDSi	484	-	564	12.60	484	-	564	81
1	+	3	CDSi	984	-	1088	14.46	984	-	1088	105
1	+	4	CDSi	1205	-	1354	16.41	1205	-	1354	150
1	+	5	CDSi	1489	-	1686	21.67	1489	-	1686	198
1	+	6	CDSl	2211	-	2252	2.72	2211	-	2252	42
1	+		PolA	2458			-4.02				

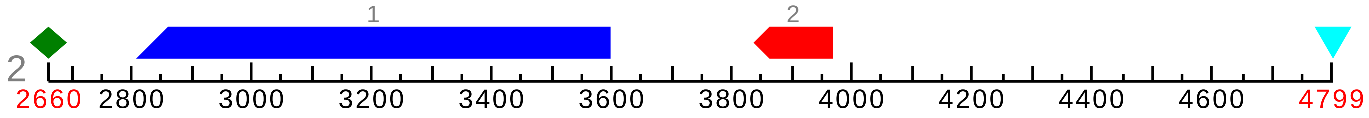

2	-		PolA	2660			-0.32				
2	-	1	CDSl	2806	-	3596	27.04	2806	-	3594	789
2	-	2	CDSf	3834	-	3966	-0.74	3835	-	3966	132
2	-		TSS	4799			-0.60				

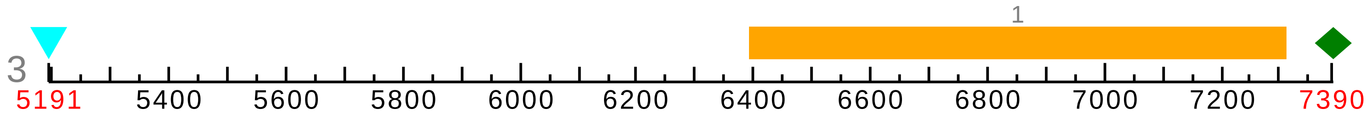

3	+		TSS	5191			0.68				
3	+	1	CDSo	6390	-	7310	24.59	6390	-	7310	921
3	+		PolA	7390			-0.32				

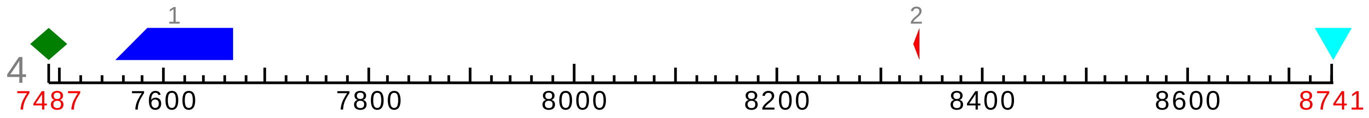

4	-		PolA	7487			1.88				
4	-	1	CDSl	7552	-	7667	18.38	7552	-	7665	114
4	-	2	CDSf	8331	-	8337	-5.26	8332	-	8337	6
4	-		TSS	8741			-1.32				

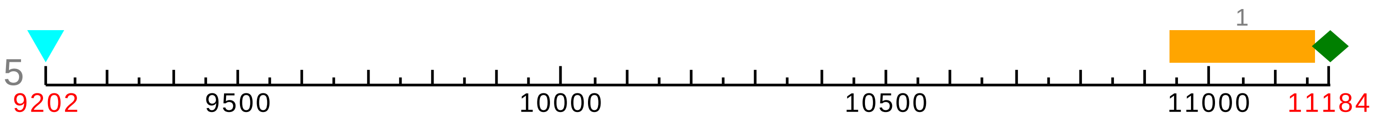

5	+		TSS	9202			0.28				
5	+	1	CDSo	10936	-	11160	5.84	10936	-	11160	225
5	+		PolA	11184			-3.32				

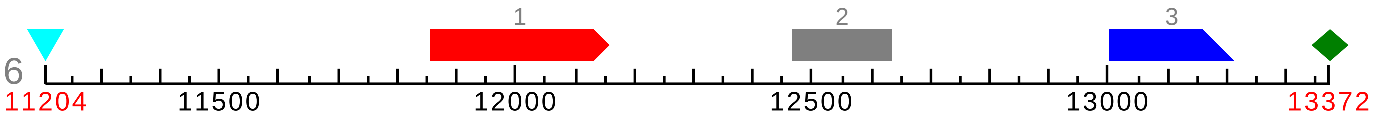

6	+		TSS	11204			-1.11				
6	+	1	CDSf	11853	-	12156	8.20	11853	-	12155	303
6	+	2	CDSi	12464	-	12633	7.96	12466	-	12633	168
6	+	3	CDSl	12999	-	13211	6.20	12999	-	13211	213
6	+		PolA	13372			1.88				

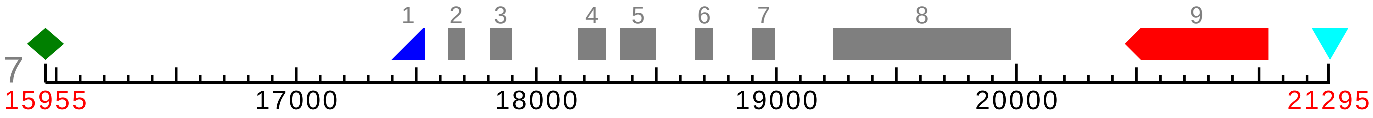

7	-		PolA	15955			1.88				
7	-	1	CDSl	17393	-	17533	1.03	17393	-	17533	141
7	-	2	CDSi	17628	-	17697	5.64	17628	-	17696	69
7	-	3	CDSi	17802	-	17893	11.50	17804	-	17893	90
7	-	4	CDSi	18170	-	18284	23.04	18170	-	18283	114
7	-	5	CDSi	18344	-	18494	7.74	18346	-	18492	147
7	-	6	CDSi	18656	-	18731	6.46	18657	-	18731	75
7	-	7	CDSi	18894	-	18989	18.39	18894	-	18989	96
7	-	8	CDSi	19231	-	19967	23.76	19231	-	19965	735
7	-	9	CDSf	20442	-	21039	35.33	20443	-	21039	597
7	-		TSS	21295			-4.61				

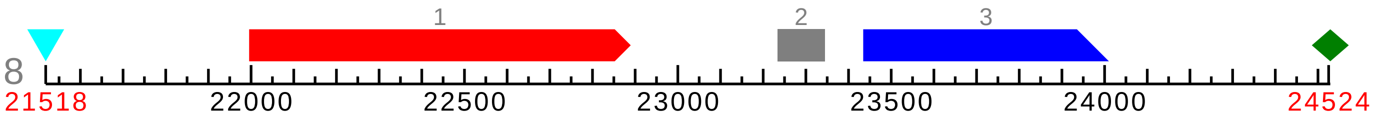

8	+		TSS	21518			-2.52				
8	+	1	CDSf	21994	-	22887	39.41	21994	-	22887	894
8	+	2	CDSi	23231	-	23341	4.30	23231	-	23341	111
8	+	3	CDSl	23431	-	24006	12.96	23431	-	24006	576
8	+		PolA	24524			-0.32				

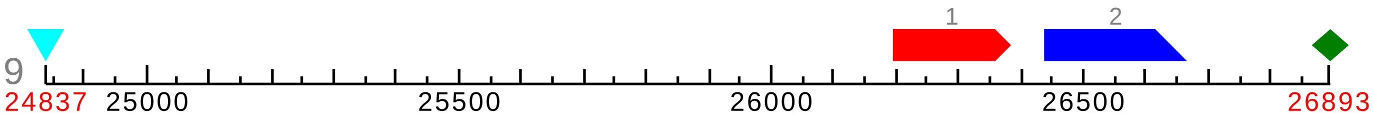

9	+		TSS	24837			-2.03				
9	+	1	CDSf	26193	-	26382	14.09	26193	-	26381	189

9	+	2	CDSl	26435	-	26664	14.05	26437	-	26664	228
9	+		PolA	26893			-0.32				

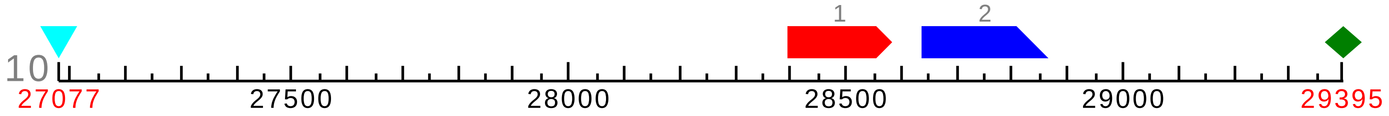

10	+		TSS	27077			-2.21				
10	+	1	CDSf	28392	-	28581	14.09	28392	-	28580	189
10	+	2	CDSl	28634	-	28863	14.05	28636	-	28863	228
10	+		PolA	29395			1.88				

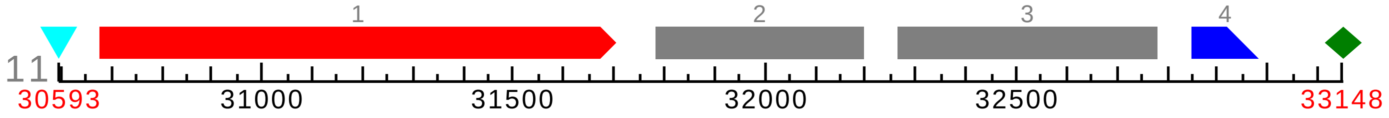

11	+		TSS	30593			1.07				
11	+	1	CDSf	30674	-	31702	115.30	30674	-	31702	1029
11	+	2	CDSi	31780	-	32194	40.10	31780	-	32193	414
11	+	3	CDSi	32261	-	32778	43.89	32263	-	32778	516
11	+	4	CDSl	32846	-	32980	19.53	32846	-	32980	135
11	+		PolA	33148			-5.12				

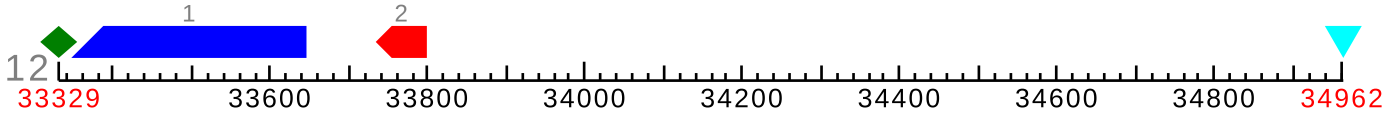

12	-		PolA	33329			-4.92				
12	-	1	CDSl	33345	-	33644	28.57	33345	-	33644	300
12	-	2	CDSf	33732	-	33797	3.93	33732	-	33797	66
12	-		TSS	34962			2.60				

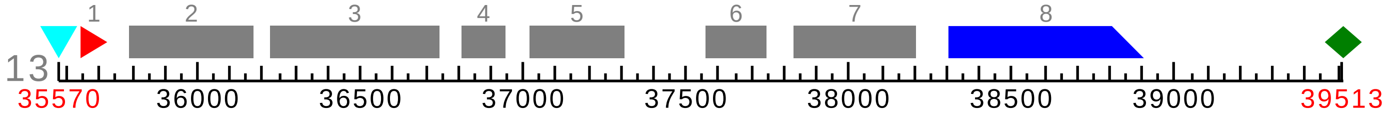

13	+		TSS	35570			-4.13				
13	+	1	CDSf	35637	-	35719	9.07	35637	-	35717	81
13	+	2	CDSi	35787	-	36168	59.36	35788	-	36168	381
13	+	3	CDSi	36219	-	36738	54.83	36219	-	36737	519
13	+	4	CDSi	36807	-	36941	21.62	36809	-	36940	132
13	+	5	CDSi	37015	-	37306	45.93	37017	-	37304	288
13	+	6	CDSi	37556	-	37742	20.96	37557	-	37742	186
13	+	7	CDSi	37826	-	38202	60.56	37826	-	38200	375
13	+	8	CDSl	38301	-	38901	109.54	38302	-	38901	600
13	+		PolA	39513			-2.92				

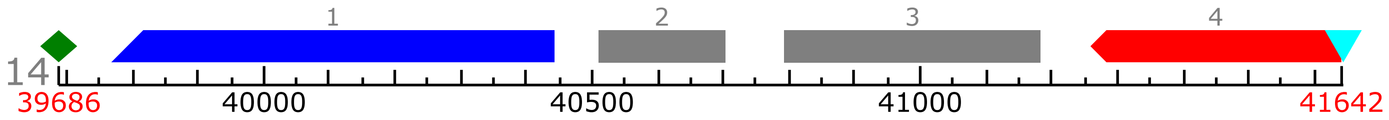

14	-		PolA	39686			-2.92			
14	-	1	CDSl	39766 -	40441	59.80	39766 -	40440	675
14	-	2	CDSi	40508 -	40701	18.64	40510 -	40701	192
14	-	3	CDSi	40791 -	41181	34.28	40791 -	41180	390
14	-	4	CDSf	41257 -	41639	39.52	41259 -	41639	381
14	-		TSS	41642		-7.81			

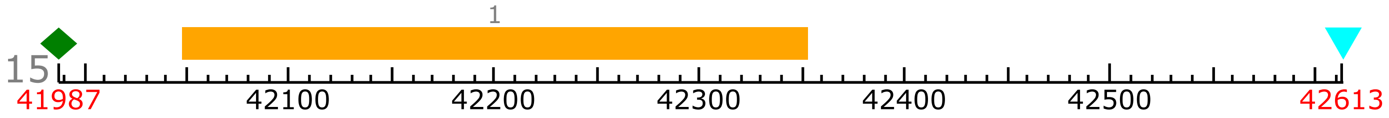

15	-		PolA	41987		-5.12			
15	-	1	CDSo	42047 -	42352	53.74	42047 -	42352	306
15	-		TSS	42613		2.59			

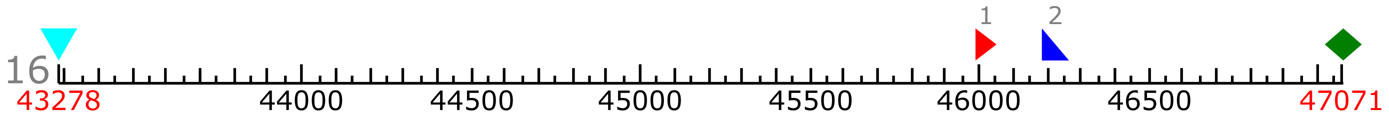

16	+		TSS	43278		0.88			
16	+	1	CDSf	45985 -	46047	9.73	45985 -	46047	63
16	+	2	CDSl	46181 -	46261	0.52	46181 -	46261	81
16	+		PolA	47071		1.88			

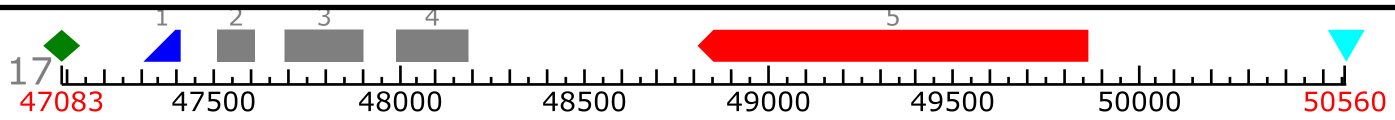

17	-		PolA	47083		1.88			
17	-	1	CDSl	47304 -	47405	2.32	47304 -	47405	102
17	-	2	CDSi	47504 -	47606	12.41	47504 -	47605	102
17	-	3	CDSi	47687 -	47900	27.63	47689 -	47898	210
17	-	4	CDSi	47989 -	48184	22.20	47990 -	48184	195
17	-	5	CDSf	48804 -	49862	167.82	48804 -	49862	1059
17	-		TSS	50560		-1.03			

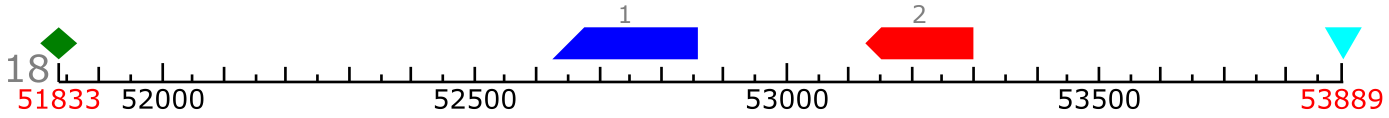

18	-		PolA	51833		1.88			
18	-	1	CDSl	52623 -	52856	13.38	52623 -	52856	234
18	-	2	CDSf	53124 -	53297	8.45	53124 -	53297	174
18	-		TSS	53889		-1.23			

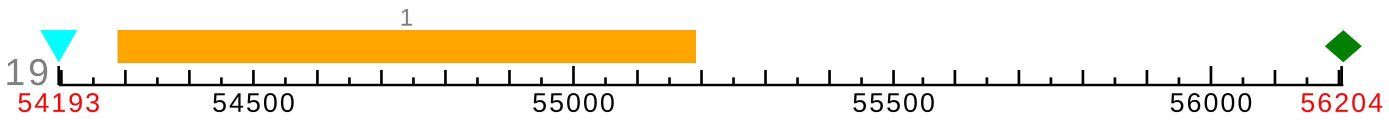

19	+		TSS	54193			1.88				
19	+	1	CDSo	54285	-	55190	22.41	54285	-	55190	906
19	+		PolA	56204			1.88				

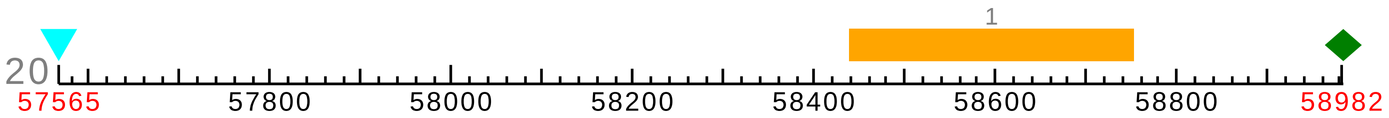

20	+		TSS	57565			3.38				
20	+	1	CDSo	58437	-	58751	16.29	58437	-	58751	315
20	+		PolA	58982			-4.72				

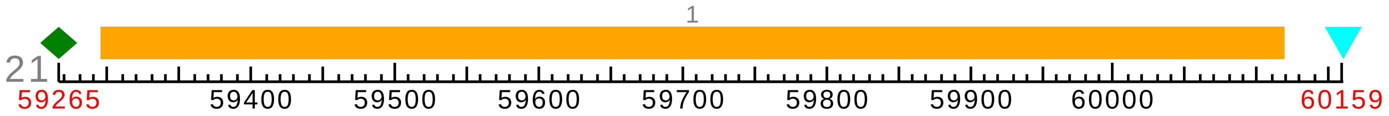

21	-		PolA	59265			1.88				
21	-	1	CDSo	59294	-	60118	189.43	59294	-	60118	825
21	-		TSS	60159			-3.23				

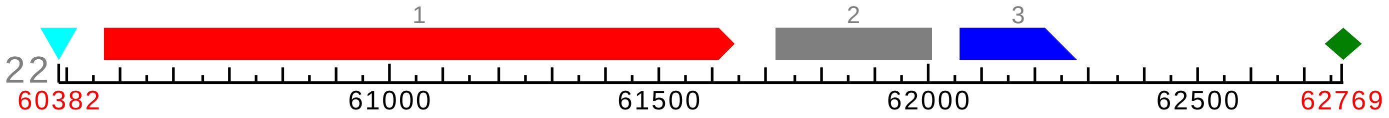

22	+		TSS	60382			-2.13				
22	+	1	CDSf	60466	-	61638	140.70	60466	-	61638	1173
22	+	2	CDSi	61714	-	62004	34.27	61714	-	62004	291
22	+	3	CDSl	62056	-	62274	21.00	62056	-	62274	219
22	+		PolA	62769			-0.32				

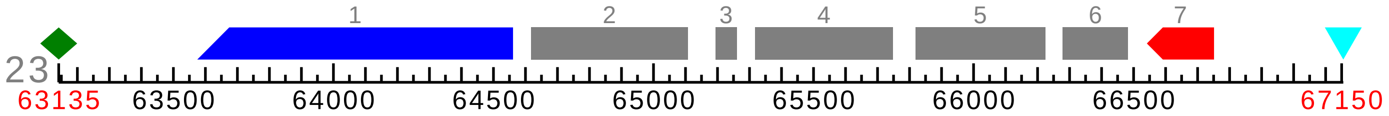

23	-		PolA	63135			1.88				
23	-	1	CDSl	63568	-	64555	121.56	63568	-	64554	987
23	-	2	CDSi	64611	-	65101	51.47	64613	-	65101	489
23	-	3	CDSi	65188	-	65254	6.65	65188	-	65253	66
23	-	4	CDSi	65312	-	65742	41.34	65314	-	65742	429
23	-	5	CDSi	65813	-	66218	39.89	65813	-	66217	405
23	-	6	CDSi	66273	-	66477	15.68	66275	-	66475	201
23	-	7	CDSf	66536	-	66746	25.19	66537	-	66746	210
23	-		TSS	67150			1.17				

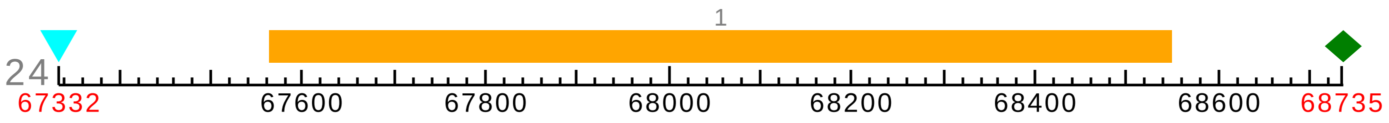

24	+		TSS	67332			-4.82				
24	+	1	CDSo	67562	-	68548	206.09	67562	-	68548	987
24	+		PolA	68735			-5.12				

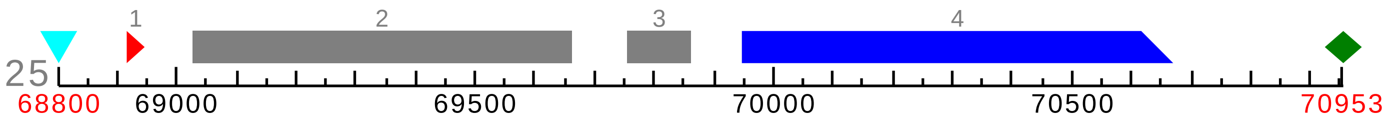

25	+		TSS	68800			-6.83				
25	+	1	CDSf	68914	-	68944	-1.87	68914	-	68943	30
25	+	2	CDSi	69024	-	69660	126.15	69026	-	69658	633
25	+	3	CDSi	69753	-	69860	1.88	69754	-	69858	105
25	+	4	CDSl	69945	-	70668	110.56	69946	-	70668	723
25	+		PolA	70953			-0.32				

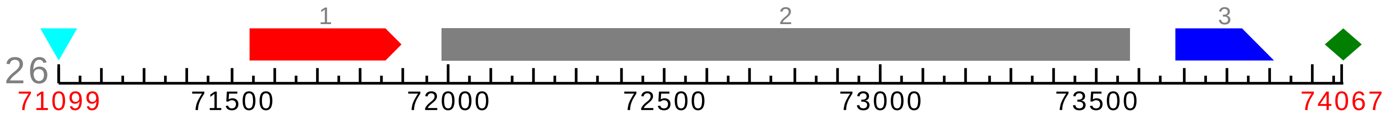

26	+		TSS	71099			2.10				
26	+	1	CDSf	71540	-	71891	39.19	71540	-	71890	351
26	+	2	CDSi	71984	-	73574	159.48	71986	-	73572	1587
26	+	3	CDSl	73679	-	73907	9.65	73680	-	73907	228
26	+		PolA	74067			-2.92				

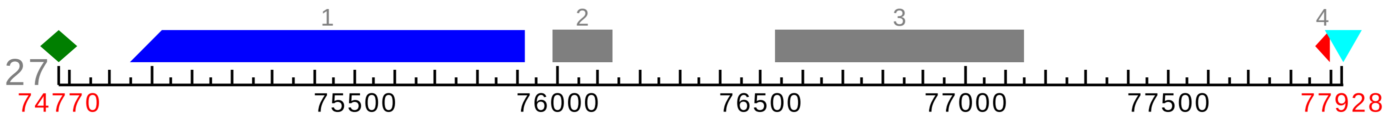

27	-		PolA	74770			1.88				
27	-	1	CDSl	74945	-	75916	149.64	74945	-	75916	972
27	-	2	CDSi	75984	-	76131	12.60	75984	-	76130	147
27	-	3	CDSi	76531	-	77143	114.42	76533	-	77141	609
27	-	4	CDSf	77859	-	77895	1.46	77860	-	77895	36
27	-		TSS	77928			-6.50				

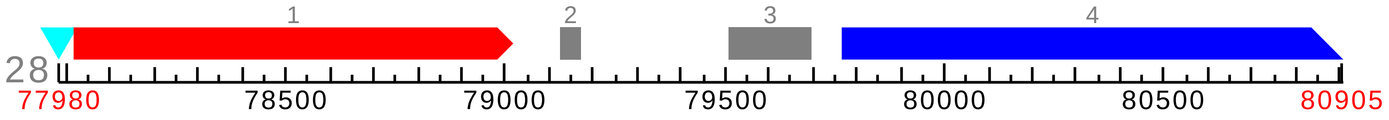

28	+		TSS	77980			-9.42				
28	+	1	CDSf	78014	-	79015	69.70	78014	-	79015	1002
28	+	2	CDSi	79122	-	79169	6.68	79122	-	79169	48
28	+	3	CDSi	79506	-	79694	9.74	79506	-	79694	189
28	+	4	CDSl	79763	-	80905	75.11	79763	-	80905	1143

Predicted protein(s):
>FGENESH:[mRNA] 1 6 exon (s) 1 - 2252 957 bp, chain +
ATGAAGCTCCAAACCCAGTCCTTCGCTCTCAACCTTCTCCCATCTCCGAAATTCTCCAAA
CCCATCGACAAACGCGAATTCATCTCCCAAAAACGAGACCCATCTCGCCCAATCTCCCTC
CGATGCTCCGTCTCTACTGCTTCCTCCACGCCGCTAACGGCGACCAATCACCATCTCTCC
GCTTCGAGCCACAAGCCCTTTCCGGCGGAGGTTTCGAGGAGCATCATGGAGCTTTCCTCA
GTCGGAACTCTCTCCACTTTGACCCACGACGGGTGGCCGCTCGGCGTGGGCGTTCGATTC
GCCGTTGACCAAGACGGCACTCCTGTGCTCTGCCTCAACCGCGCATTGTCTCCTGACAAG
AGGTCTGCTCTTCATGTTCAGTTGGAGCAATGTGGATTGAGAACTCCTCAGTGTACGATA
CAAGGTAGCATTGCTCGACCTGGAGACGACCAAGTTGTAAAGCGTCTTAGTGCTACATGG
AAGAAAAGGTTTGGTGAGCAAGTTGAGGAAGACAGTTTATATGTTGTTGCTGTTGACCGT
GTTCTTCAAATGGAAGACTTCATGGAGGATGGAGTTTGGGTGGCGTCAGTAGATTATAAA
AATGCAAGCCCTGATCCTCTTCGAGGTGTTGCAGAAGACATTGTCAACCAGATTAACGCT
AACAACATGGAAGACATTTTCCGTTTCTGCAACGTATACGTTGATCTGGACTTTGTGGTT
TCAGAGACAAAGATGATATGGATGGATAGGCTTGGATTCGACCTTCGAGTATGGTCTCCA
CGAGGTGTATACGATGTCAGGATTCCTTTTCCAATGGAAGTGACAGATGAAAAAGGAGCC
AAATCATCGTTTAATGGAATGTCACAGCTCGCTTGGGAAGTAGAGAAAAGTTACTGCCCT
GCAGATTTCAACAAGGTAACAGATGTGGCACCGTCTAAAGATTTGCCATCCTTATAA
>FGENESH: 1 6 exon (s) 1 - 2252 318 aa, chain +
MKLQTQSFALNLLPSPKFSKPIDKREFISQKRDPSRPISLRCSVSTASSTPLTATNHHLS
ASSHKPFPAEVSRSIMELSSVGTLSTLTHDGWPLGVGVRFAVDQDGTPVLCLNRALSPDK
RSALHVQLEQCGLRTPQCTIQGSIARPGDDQVVKRLSATWKKRFGEQVEEDSLYVVAVDR
VLQMEDFMEDGVWVASVDYKNASPDPLRGVAEDIVNQINANNMEDIFRFCNVYVDLDFVV
SETKMIWMDRLGFDLRVWSPRGVYDVRIPFPMEVTDEKGAKSSFNGMSQLAWEVEKSYCP
ADFNKVTDVAPSKDLPSL
>FGENESH:[mRNA] 2 2 exon (s) 2806 - 3966 924 bp, chain -
ATGTACATCTGCAAAGAACCTTCTTGCCATATTCCATCACTACAGCATAGTTTCCACAAA
GTGTGTATCGAGCCGATATCAAACCACCCATATCATCCTAAGCACACTCTCCGGCTTATT
TTTACAGATCCAATCCATATAGAGTGTCTAAACATTCCAAGTATCATAAGAATATCACGT
CATGAACACCGTATCTCTTTCAATTCTTCTCTTACTCCCGAAAAATTGATGTGTGGAGTG
TGTCACCGGAAGATAGACACAAATTATGGAAGATATTCTTGCATAAATGGTTGCAAGTAT
GGTGTTCATTCTAAATGCGCAACACGGGAAGATGTATGGGATGGAGAAGAACTTGAAGGA
AAACCAGAAGATCCATATGAAGATATTAGCTCGTTCAATGTGATAAGTGAGGGAATTATA
CAACATTTTAGTCATCAAGATCATCATATGAGACTCGATGAGAAGACCGATAGGGAATAT
GACGATAACGATTATTGTCAAGCATGCACACTTCCTATATGCGATGATATTATTTACAGT
TGCATGCAGTGTGATTTTATCCTCCATCAAGAATGTGCACATCTTCCTCGGAAGAAACAT
CAAGTGACGCATCCTCATTCCCTTAGTCTACGAGTGGATCATCATGGCTGCGCATTGTTC
GAATGTCAAGCTTGTCTTCATTCAAGCAATGGATTTGCGTACTTATGTGAAGAAGAGGAT
TGTGATTATATGTTAGACGTACGTTGTGCTTCTATAGCTGAGCCATTGGATCATCGGTAT
CATCAGCATCCCTTGTTTCTTACATTTGAACCCCGATCTAAACAAATATGTTCGGTTTGC
CATAAATCTCGAGGCCATCGTCTAAATTGTGAAGATGTGGTTTCGTTGTGTGTTTTGGAT
GCGCAACTTTACCCAATAAGTTGA
>FGENESH: 2 2 exon (s) 2806 - 3966 307 aa, chain -
MYICKEPSCHIPSLQHSFHKVCIEPISNHPYHPKHTLRLIFTDPIHIECLNIPSIIRISR
HEHRISFNSSLTPEKLMCGVCHRKIDTNYGRYSCINGCKYGVHSKCATREDVWDGEELEG
KPEDPYEDISSFNVISEGIIQHFSHQDHHMRLDEKTDREYDDNDYCQACTLPICDDIIYS
CMQCDFILHQECAHLPRKKHQVTHPHSLSLRVDHHGCALFECQACLHSSNGFAYLCEEED
CDYMLDVRCASIAEPLDHRYHQHPLFLTFEPRSKQICSVCHKSRGHRLNCEDVVSLCVLD

AQLYPIS
>FGENESH:[mRNA] 3 1 exon (s) 6390 - 7310 921 bp, chain +
ATGACCACCCACTTTACAAAACCAATAAACCTCAGGCAGTTACAAACCTTTACCAACAGA
GTCTTCTACGACGATAGCCTTTATCCAACCGACAACCTAACCAAAAACAGAACTTTCTAT
GAGTATATTCTAGTAGACTCTGGATCAGTAGAAATTACCCACTACCCAGACAGAATAAAC
ACCTCCTACTCAACATGTAAAATCTTGCGAGTCTGCTCTATTCAAGACCTCGGGTTTATC
CACCACCACACTATCAAACCATTCTCAACACCGGGCTACCACATTAAAGGATATTCATAT
GTGGACTATCAAAATGCCTTCTTCAGAACCTTCTACGTCAAATCCTATGACCACTCTTGG
TTTCTCTCCTTCGACCAAAAATGCAACAAAAACATCCCAGGATGGTTCAACGAATGGTGG
TACTGGCTTGGACCATCAGATCAAATTTATCCACCAGAGATCATCAAGACTTCTTTCCCT
TACTACAAGAAACAAGTACCTGACCAACCAGTGGGACCAATTACCAAGATCGCTTTCCAC
ATTGACATGGGAATACCGTGGATCTGTTCGTGGCATTTTAGCCTAGAACTCGCTCTACCT
GACATGCCATACTCCCTAATCAGAGAATACATAATCAAATGGTGGGAAAAATACAATCTC
GACCGTTGCTCCCTAACCAATATCCAAAAATACTTTCAGATATCCAAACAAACCGAAACC
GATATCCAAACCATATCAAAATCCATACCCCTTTTAACTCCAAACCAACTAATGCATCAA
ACCAAACCAAGTCTACCAACAACAACCTCAAAACCAGAATCAAAAAAAGCCCAAACAGAT
AGCCCAACCCATTCATCAACCTCTTCAAACTCCAAAAAATCTTCTAAAAAAGACATCCTC
AAGAAACTTCTCATGAACTAA
>FGENESH: 3 1 exon (s) 6390 - 7310 306 aa, chain +
MTTHFTKPINLRQLQTFTNRVFYDDSLYPTDNLTKNRTFYEYILVDSGSVEITHYPDRIN
TSYSTCKILRVCSIQDLGFIHHHTIKPFSTPGYHIKGYSYVDYQNAFFRTFYVKSYDHSW
FLSFDQKCNKNIPGWFNEWWYWLGPSDQIYPPEIIKTSFPYYKKQVPDQPVGPITKIAFH
IDMGIPWICSWHFSLELALPDMPYSLIREYIIKWWEKYNLDRCSLTNIQKYFQISKQTET
DIQTISKSIPLLTPNQLMHQTKPSLPTTTSKPESKKAQTDSPTHSSTSSNSKKSSKKDIL
KKLLMN
>FGENESH:[mRNA] 4 2 exon (s) 7552 - 8337 123 bp, chain -
ATGGAATGTATGTCATGGTGTCTTGGTGCTGCTGGAGTAGTCTTGGTGGGTTGTGCTGCT
GGAGTTCATGTGCTGCTAGAGTTCTTCACGTGGGTCCTAAGTGTCGGCCATTACGATGCT
TGA
>FGENESH: 4 2 exon (s) 7552 - 8337 40 aa, chain -
MECMSWCLGAAGVVLVGCAAGVHVLLEFFTWVLSVGHYDA
>FGENESH:[mRNA] 5 1 exon (s) 10936 - 11160 225 bp, chain +
ATGTCAGAGAATGAAATGGATGCAAATGCTGACTCTCTGCCTGCAAGATGCATCCTATTC
GTGCAGTTGTTGGAAGATAAAGAAGAGAGTACATCCCTATCCAAGGAACTACTGATATGT
CAAGGCTCCTTGAGCTCCATTTGCACTTGGAGAAGGAAAGAACGAATGGCCGCCACCTTC
CTTCCTTTTTCCATATGGATTCTTGGAAATGTATCATTGTGTTAA
>FGENESH: 5 1 exon (s) 10936 - 11160 74 aa, chain +
MSENEMDANADSLPARCILFVQLLEDKEESTSLSKELLICQGSLSSICTWRRKERMAATF
LPFSIWILGNVSLC
>FGENESH:[mRNA] 6 3 exon (s) 11853 - 13211 687 bp, chain +
ATGGATGGGCGGGAGGTGGCTATCAAAAGGAAAGGAAGTTTTGATAAAATCTATATTAGA
ACTCCGACATATGTTATGTCTACCTTTTACTCCCGCTGGAGATATGTGAGAACCTCGCAA
GTGTCATTGCACAATTCGGTGGAGCTCAAATCCACCAAAGAGAGGGATTCATTGGCTAAA
TGGGAGAAGATGTGTTTACCAAGAGAAGAGGGGATTGGTTTCGTGATGATCCATGAGTTC
AACCTGGCTTTACTAGCCAAACAACTATGGAGACTAGTCCATTCCCGACTCGTTGGTAGC
TCGAAGTTTGGCCATAAGCTCAACTCACCACAGAGACTCCTTCTCGCTGGAATTATACCA
AAATGGCCAATACACGTTAAATCGGGATATTGGGTAGCACGGAACCTAATGAGGAATGAT
GAAGATAAGGAGATCCGGAACCAAGCATCACCAAACCAAGCCTTTGTTTGGAAGTTAGAT

ATGCTGAAAGTGAATGCCAGGCTTGGTTTAATGCCAATGAGAGGATCGGATAACCCACGT
GAGCTGCACAATGAGGAACTCCAAGCCATAAGCTTGGATAATATATGTATGGTGGATGGA
TCATGGACTTCCATGGCACGTTTAGTGGCTGCGGATGGGTCTGGAAGGATAGTCAGGACG
AACACAGCTTATGGAATGCGAACTTAA
>FGENESH: 6 3 exon (s) 11853 - 13211 228 aa, chain +
MDGREVAIKRKGSFDKIYIRTPTYVMSTFYSRWRYVRTSQVSLHNSVELKSTKERDSLAK
WEKMCLPREEGIGFVMIHEFNLALLAKQLWRLVHSRLVGSSKFGHKLNSPQRLLLAGIIP
KWPIHVKSGYWVARNLMRNDEDKEIRNQASPNQAFVWKLDMLKVNARLGLMPMRGSDNPR
ELHNEELQAISLDNICMVDGSWTSMARLVAADGSGRIVRTNTAYGMRT
>FGENESH:[mRNA] 7 9 exon (s) 17393 - 21039 2076 bp, chain -
ATGAAGTGTGAAAACTTGATGAAACAAGGGCAGTCTATCAAGCATGATTTGCATAAACAA
GATGATGTTACAAAGCATGAGTACCAGATTCTGTTGAATGCTTCAGTTGATGCTTCTAGA
TATTTATTGAGGCAAGGGTTACCTTTTCGTGGTCATGATGAGTCCGAACAATCTGCTAAT
AGAGGAAACTTTGTGGAGCTACTGAGATACACTGCAGAACAAAACGAGGTTGTGAGTAAG
GTTGTTTTAGAGAACGCTCCTGGAAATAATCAGATGATATCTCCAAAGATTCAGAAAGAT
ATTATGCATTGTTTTGTAGAAGAACTTGTCAAATCTATTATTGAAGAAATTGACAATGAT
GTCTTTGGTTTATTGGTGGATGAGTCAGCTGATGTTTCTGATAAAGAACAGATGGCGGTA
GTGTTTCGTTATGTTGACAAAAGTGGGATAGTTAAAGAGAGATTCATCAGTATTACACAT
GTGAGTGAAACATCTTCAAAATCTTTGAAATCTGATATTGATTCTTTATTTGCTAAGTAT
GGATTGAGTATCAAAAAGGTGAGAGGACAAGGATATGATAGAGCAAGTAATATGAAAGTA
CATCTTTTGGGACTCACGGACAGCTTGTCTATGGCTCTTCAAAGAAGAGATCAGGATATT
TTGAATGCAATGTCATTGGTGAAATCAACTAAAAGACAACTGCAGAAGTTCAGGGATGAC
GGATGGAGTTCTCTCATGATCAAAGTTTTTTCCTTTTGTGGGAAATATGATATTGAAAAG
GTTAACATGGAAGAAGACTTTGCTGACTCAAGGAACCCAAGGAAAGAAGTAGGAGTAACA
AATCTGCATCACTACAGAGTTAATTGCTTGTATGCTGTTTTGGGTTTGGAAATTCAGAAG
TTTAACAATCGCTTCAATGAGGTAAACACTGAACTGCTCATATGTGCAGCTTCTTTATCT
CCCATTGATTCCTTTGCTGGGTTTGACTACTCAAAAGCGATGAAGTTATCTAAGTTATAC
CCAGACGACTTTAGTTATGCTGAGTGCATATCTCTTGAGCAACAACTAGATATCTACATT
GATAATGTTCGAAGTGATGAAAGGTTTACTCACTTGAAGAATCTTGGAGAACTTGCTCGC
ATGATGGTGGAAACACGGAAACATATGTCACATTCTCTTGTCTATAGGCTTTTGAAGTTG
GTTTTAATTTTACCCGTTGCTACTGCCACTGTTGAAAGATGTTTCTCAGCTGTGAAGTTT
GTGAAGACATATCATCGCAATCGGATTGGAGATCAGTTTCTGAATGATTATTTAGTTTGT
TTTGTCGAGAAAGATCTTCGGACAATCTTCATCGCTGGGCTTCCTGACGATGTTAAAGAG
AGAGAGCTTCTGAATCTCTTGAGATGGTTGCCTGGTTACGAGACTTCTCAGCATCTGGTG
TTTGATGCTGAGTCGAAGTCTGTGATACATACAGAGATGGCCAAGAAGAATCACTTTGTT
AAAAGAGGAATTGTTGGGGATTCAAATGCTTATGATCAAAGTAAGCAGTTACGACTGGTG
GTGGTGACTGCACACATTCTGTTTATAGTCCGCCTCCCTTCCAGTCTCCACCACCACCTC
CAGTTTGGGGTCCACCTCATGGGTGAGGCATTTGACTCGTATATGGCACCCGCACCTCCA
CCATATGACCCCTATGCAGGTTATCACGCTCCCCCTGTACCTGTGCCTCCGCCAACACCT
ATAGCAGCTCCTAGTTCATATGTGCCTGTCCAGAATATTAAGGATAATCCTCCTTGCAAT
ACCTTATTTATTGGTAATCTCGGGGAGAATATTAACGAAGAAGATTTGAGGAGTCTGTTG
AGCTCGCAACCTGGTTTCAAGCAAATGAAGATTCTTAGACAGGAAAGGCATACCGTCTGC
TTTATTGAATTCGAGGATGTGAATAGTGCCACAAACATTCACAATAATTTGCAAGATGCT
GTCATCCCAAGTTCTGGTTCAGTTGGCATGAGGATCCAATATCCTTTAATCACTCACTGT
ACTTGTGTTCTTTTATATTCCAGTATGGCCTGTTAA
>FGENESH: 7 9 exon (s) 17393 - 21039 691 aa, chain -
MKCENLMKQGQSIKHDLHKQDDVTKHEYQILLNASVDASRYLLRQGLPFRGHDESEQSAN
RGNFVELLRYTAEQNEVVSKVVLENAPGNNQMISPKIQKDIMHCFVEELVKSIIEEIDND

VFGLLVDESADVSDKEQMAVVFRYVDKSGIVKERFISITHVSETSSKSLKSDIDSLFAKY
GLSIKKVRGQGYDRASNMKVHLLGLTDSLSMALQRRDQDILNAMSLVKSTKRQLQKFRDD
GWSSLMIKVFSFCGKYDIEKVNMEEDFADSRNPRKEVGVTNLHHYRVNCLYAVLGLEIQK
FNNRFNEVNTELLICAASLSPIDSFAGFDYSKAMKLSKLYPDDFSYAECISLEQQLDIYI
DNVRSDERFTHLKNLGELARMMVETRKHMSHSLVYRLLKLVLILPVATATVERCFSAVKF
VKTYHRNRIGDQFLNDYLVCFVEKDLRTIFIAGLPDDVKERELLNLLRWLPGYETSQHLV
FDAESKSVIHTEMAKKNHFVKRGIVGDSNAYDQSKQLRLVVVTAHILFIVRLPSSLHHHL
QFGVHLMGEAFDSYMAPAPPPYDPYAGYHAPPVPVPPPTPIAAPSSYVPVQNIKDNPPCN
TLFIGNLGENINEEDLRSLLSSQPGFKQMKILRQERHTVCFIEFEDVNSATNIHNNLQDA
VIPSSGSVGMRIQYPLITHCTCVLLYSSMAC
>FGENESH:[mRNA] 8 3 exon (s) 21994 - 24006 1581 bp, chain +
ATGTGGTGTCAATTGAACCCACATTTCGTACATCGATTTGTAATCAAAATAAAGGCTCCA
TCTTCACCTCCAGCTCCATCTTCACCTCCAGCTCCAAGTGATTTAGATGATCTGCCATGG
GATCCTGCTGACCGGAAAAGAATATCAGAATATCCTTTGAATCAAAGAAGTGAAGTAATT
CGCAGATATTTAATTAGAGGTCCTTGTCAACCTCGTGGGCTTATCTTTAAACAAAAAATG
TTTGGAGGCATAATGAGGCGATTTAATCCTGCTTGGTTTGACAAGTATGAGAACTGGTTA
GAGTATAGTGTGAAGAAAGAGGCAGCTTTTTGTCTATGTTGTTATTTGTTCAGAGATAAT
GCTGGAAAGGGTGGACAAAATGATGTATTTACCATTGACGGTTTCTCTAGCTGGAATAAG
TTTCAAAGGCTTAAAGATCACGTGGGAGGTCCTAATAGTTTTCATAACAATGCAGTCATG
AAGTGTGAAAACTTGATGAAACAAGGGCAGTCTATCAAGCATGCTTTGCATAAACAAGAT
GATGTTACAAAGCATGAGTACCGGATTCGGTTGAATGCTTCAGTTGATGCTTCTAGATAT
TTATTGAGGCAAGGGTTACCTTTTTGTGGTCATGATGAGTCCGAACAATCTGCTAATAGA
GGAAACTTTGTGGAGCTACTGAGATACACTGCAGAACAAAACGAGGCTGTGAGTAAGGTT
GTTTTAGAGAACGCTCCTGGAAATAATCAGATGATATCTCCAAAGATTCAGAAAGATATT
ATGCATTGTTTTGTAGAAGAACTTGTCAAATCTATTATTGAAGAAATTGACAATGACGTC
TTTGGTTTATTGGTGGATGAGTCAGCTAATGTTTCTGATAAAGAACAGATGGCGGTGGGA
GCTTCATGTAAAAGGAAAAATATGATACGAGAGACTTACAAGAAAATGATTGAGGAAGGA
ATTAACTGTGGTGAAATGAATACTGGAACAGGATTGAATCAAGAGGTGCTTAAGTATGTT
CAGAATGAAGGCGTTGAAGACTTAAAAAGACGTCAAGCACATGGTCTCATCAGATATTTT
CATAGCCTTGAATATGCATTCTATTTGCAAATGATGGTACATCTTTTGGGACTCATGGAC
AGCTTATTTATGGCTCTGCAAAGAAGAGATCAGGATATTTTGAATGCAATGTCATTGGTG
AAATCAACTAAAAGACAACTGCAGAAGTTCAGGGATGATGGATGGAGTTCTCTCATGATC
AAAGTTTTTTCTTTTTGTGAGAAATATGATACTGAAAAGGTTAACATGGAAGAAGACTTT
GCTGACTCAAGGAACCCAAGGAAAAAAACAGGAGTAACAAATCTGCATCACTACAGAGTT
AATTGCTTGTATGCTGTTTTGGATTTGGAAATTCAGGAGTTTAACGAACGCTTCAATGAG
GTAAACACAGAACTGCTCATATGTGCGGCTTCTTTATCTCCCATTGATTCCTTTGCTGGG
TTTGACCACTCAAAAGCGATGAAGTTATCTAAGTTCTACCCAGACGACTTTAGTTATGCT
GAGTGCATATCTCTTGAGTAA
>FGENESH: 8 3 exon (s) 21994 - 24006 526 aa, chain +
MWCQLNPHFVHRFVIKIKAPSSPPAPSSPPAPSDLDDLPWDPADRKRISEYPLNQRSEVI
RRYLIRGPCQPRGLIFKQKMFGGIMRRFNPAWFDKYENWLEYSVKKEAAFCLCCYLFRDN
AGKGGQNDVFTIDGFSSWNKFQRLKDHVGGPNSFHNNAVMKCENLMKQGQSIKHALHKQD
DVTKHEYRIRLNASVDASRYLLRQGLPFCGHDESEQSANRGNFVELLRYTAEQNEAVSKV
VLENAPGNNQMISPKIQKDIMHCFVEELVKSIIEEIDNDVFGLLVDESANVSDKEQMAVG
ASCKRKNMIRETYKKMIEEGINCGEMNTGTGLNQEVLKYVQNEGVEDLKRRQAHGLIRYF
HSLEYAFYLQMMVHLLGLMDSLFMALQRRDQDILNAMSLVKSTKRQLQKFRDDGWSSLMI
KVFSFCEKYDTEKVNMEEDFADSRNPRKKTGVTNLHHYRVNCLYAVLDLEIQEFNERFNE
VNTELLICAASLSPIDSFAGFDHSKAMKLSKFYPDDFSYAECISLE

>FGENESH:[mRNA] 9 2 exon (s) 26193 - 26664 420 bp, chain +
ATGTCTTGCTCGAGTTCCTGGACCAAGGTTCTCTCGAAGGCGCGCACGTGTGGCATGAGT
AAGAACTCGCCGATCTCACCCAGAAGATTTTCAGCGGATTACCTTCATCGCCGTCACATC
GTTCACCGCAACATCAAACCTTCGAAACTCTTGACTAACTCGGCCAAGAACGTGAAGATC
GCCTATCTTGAACTTAGAACCATCACTTACATGAGCCCGGAGAGGATCAACACCGATCTG
AATCATGGACTTTACAACGGCTACGCTGGAGATATATGGAGTCTTGGTGTTAGCATCTTG
GAGCTCTACTTGAGGAGATTCACTTTCATGTCTCAGCCGCCTGAAGCTCCAGAGAAGGCG
TCTCAGGAGTGTCGTCACTTTATCTCTTGTTGCTTACGAAGTGATCCTCCAAAGAGATAG
>FGENESH: 9 2 exon (s) 26193 - 26664 139 aa, chain +
MSCSSSWTKVLSKARTCGMSKNSPISPRRFSADYLHRRHIVHRNIKPSKLLTNSAKNVKI
AYLELRTITYMSPERINTDLNHGLYNGYAGDIWSLGVSILELYLRRFTFMSQPPEAPEKA
SQECRHFISCCLRSDPPKR
>FGENESH:[mRNA] 10 2 exon (s) 28392 - 28863 420 bp, chain +
ATGTCTTGCTCGAGTTCCTGGACCAAGGTTCTCTCGAAGGCGCGCACGTGTGGCATGAGT
AAGAACTCGCCGATCTCACCCAGAAGATTTTCAGCGGATTACCTTCATCGCCGTCACATC
GTTCACCGCAACATCAAACCTTCGAAACTCTTGACTAACTCGGCCAAGAACGTGAAGATC
GCCTATCTTGAACTTAGAACCATCACTTACATGAGCCCGGAGAGGATCAACACCGATCTG
AATCATGGACTTTACAACGGCTACGCTGGAGATATATGGAGTCTTGGTGTTAGCATCTTG
GAGCTCTACTTGAGGAGATTCACTTTCATGTCTCAGCCGCCTGAAGCTCCAGAGAAGGCG
TCTCAGGAGTGTCGTCACTTTATCTCTTGTTGCTTACGAAGTGATCCTCCAAAGAGATAG
>FGENESH: 10 2 exon (s) 28392 - 28863 139 aa, chain +
MSCSSSWTKVLSKARTCGMSKNSPISPRRFSADYLHRRHIVHRNIKPSKLLTNSAKNVKI
AYLELRTITYMSPERINTDLNHGLYNGYAGDIWSLGVSILELYLRRFTFMSQPPEAPEKA
SQECRHFISCCLRSDPPKR
>FGENESH:[mRNA] 11 4 exon (s) 30674 - 32980 2097 bp, chain +
ATGGCTAATGAAGTGAACGGTGAGGACTTTGATGAAGAAACAGCTGAGAGAGAAGAAAAC
GAGATCGAGCAGGTACTGGAAGAATTCATCGACGAGCCTCCTGTACGCCACGACGAGTGT
CCCTCGAGTGAGGATGAAGATAGTGAGGATGAGCAAGGTGAACCTCGTATGAAGGTGAAG
AAGGTGCTCCCTGTGAAACGCGGTGCTGGAAGTCTTTACAAGGAGCAACGTTTCTTGAAT
GGTGTGGCTTTTAAGGACTCTGTGCTCGATTATGCTCTGAGAACAGGCCGGAATATCAAG
CAGTACAGGTATGATAAGGATAAGATTGGGTTCATATGTGTTGGTGGTGAAGGATGTGAA
TGGAAAGTGTATGCGTCCACTCTTCCTAAGGATAATGTCTGGAAAATTAGAGTCTTCAAA
GAGGAGCATTCATGTATTCCAAATGGTGAATGTGAGATGTTAAAAGTTCCTCAAATTGCA
AGACTTTTTGTTGATAAGATTAGAGAGGAACCGGAGTACTACATGCCCATGAAGATTGAA
GAATTGGTTCATGAAAAATGGGGGTTTACGGTATCTAGGCCTCAATGCCAGGCTGCAAGA
AACAAGGCAGTAAGATGGATTGAGTTCGAGAATGATCATATGTTTGCCCGGCTAAGAGAT
TATGCTGCTGAGTTGCTCGAATCAAACCCGGATTCGACTGTGGAGATTGAGACTGTGACT
AACACGACTGGTGAGAAAGCGAAAGAAGATTTCAACAGGATATTTATTTGCTTTGATAAC
ATCAGAAAGACATGGAAACAGTCATGTAGGCAACTAATAGGAGTCGATGGATGCTTCTTA
AAGCACAAAGTCAAGGGACAGCTTCTTGTTGCATTAGGAAGAGACGCAGATAATGCTATA
TATCCAATAGCATGGGGGGCTGTTCAAGTGGAAAACACAGAAAACTGGTTATGGTTTGTG
AGAAAGATCAAAGAAGCCTTGGGATTGAATCAGGGTCTTGGTTACATACTGGTGTCAGAT
CGTCAAAAGGGATTGATTGCAGCTGTTCAGACTGAGTTACCACAGATTGAACACAGAATG
TGTGTACGCCATATCTATGGTAATCTGAAGAAGAATCATGCTAGTAAAAAGGAGATGAAG
TCATTAATATGGAATTTAGCATGGAGCTACAACGAGGCTGAGTTCCAACAACGTTTAGAT
AGGATCTTCTGCTATGATGCTGATGTGTATGATGATGTTCAGAAGAGTAAGCCTAGGACA
TGGTGTAGAGCCTTCTACAAGCTGGGTAACTACTGTGAAGATGTAGAAAATAATTCTACA
GAGTCTTTCAACAACACAATCTCTAAGGCTAGGGAGTTTGCTTTTGTGCCAATGCTAGAG

GGTATTCGTAGATTGGCAATGGCTCGAATTGCAAAGAGATCAGTCATCGCCAATGCCCAC
AAAGGTAAATGTACACCATATGTTGCAGACTTTCTTAAGGCTGAGCATAAGCAGGCATCT
CTGTGCAAGGTTACTAGAAGTACAAATGGGATGTATGAAGTAAAGTTGAGTTCATGCAAT
TACCGTGTTAGCTTGGAGAGGAGGACTTGCACATGCATGAAGTTCGAAATCTGTGGCATC
CTTTGTGAGCATGCTTATGGAGTGATACTGCATAAGAAGTTGGTCCCTGAAGATTATGTG
TGTGACTGGTTTCACACAGCAACATGGCGAAGAAACTACAGAGATGGTATTGTCCCTGTG
CGAGGTGCTGATTTCTGGCCTAAAACGTCTGCTCCTGATGTCCATATTCCTCCTGAACCG
CCTCAACCTGGTCGAAAGAAGATTACCAAAGCAGATAAGAAAAGGAAGAAAGGAGTCAAT
GAATCACCAACAAAGAAAGCCCCAAAGAACAAGAAACGTACTATGCATTGTGGGATTTGT
GGACAACCGAATCACAACTCAAGGTTTCACACAAAGCAACAGGCTTCTCAAGGTCCTCCT
CGAGTTCTTTCTCAAGTTGCTTCTCCAGCCGCATCTCAGACTGCTTCTCAAGTGGCTTCA
CCTGCTTCTTCAGCTTCTGGTTGCTATGTTATACACGGCTATGAAGACATGTGCTGA
>FGENESH: 11 4 exon (s) 30674 - 32980 698 aa, chain +
MANEVNGEDFDEETAEREENEIEQVLEEFIDEPPVRHDECPSSEDEDSEDEQGEPRMKVK
KVLPVKRGAGSLYKEQRFLNGVAFKDSVLDYALRTGRNIKQYRYDKDKIGFICVGGEGCE
WKVYASTLPKDNVWKIRVFKEEHSCIPNGECEMLKVPQIARLFVDKIREEPEYYMPMKIE
ELVHEKWGFTVSRPQCQAARNKAVRWIEFENDHMFARLRDYAAELLESNPDSTVEIETVT
NTTGEKAKEDFNRIFICFDNIRKTWKQSCRQLIGVDGCFLKHKVKGQLLVALGRDADNAI
YPIAWGAVQVENTENWLWFVRKIKEALGLNQGLGYILVSDRQKGLIAAVQTELPQIEHRM
CVRHIYGNLKKNHASKKEMKSLIWNLAWSYNEAEFQQRLDRIFCYDADVYDDVQKSKPRT
WCRAFYKLGNYCEDVENNSTESFNNTISKAREFAFVPMLEGIRRLAMARIAKRSVIANAH
KGKCTPYVADFLKAEHKQASLCKVTRSTNGMYEVKLSSCNYRVSLERRTCTCMKFEICGI
LCEHAYGVILHKKLVPEDYVCDWFHTATWRRNYRDGIVPVRGADFWPKTSAPDVHIPPEP
PQPGRKKITKADKKRKKGVNESPTKKAPKNKKRTMHCGICGQPNHNSRFHTKQQASQGPP
RVLSQVASPAASQTASQVASPASSASGCYVIHGYEDMC
>FGENESH:[mRNA] 12 2 exon (s) 33345 - 33797 366 bp, chain -
ATGTCGAAATCTGACAACAATCCTTACCGAAGGTATTTCCGTTGTGCTTATGCTGTAAGA
AAGAAGCTTGAGAATGATAATCATGTCTTCAAATGGATTGATGAGGCATTGATAAATGAG
ATTGAAAAACTTGAATTCAAAAACGCAAGACTTGAAGACGAGTTGAAAGAAGTGAGAAGA
AATGAGATATTGTTGGGTGAGAGAGTTCAAATGAAAGTGGAGAAAGAGCTGTTTGATAAG
GTGGATGATAAGGTGGATGAAGTGTTGGCTGAAGCTAAAGCTAGCATGAAGAAAATGATG
CTATCAGCTGTCATTGGATGTGTGGTGTTGGTTGGTATCATGGAGGTTTTTCACAAAGCC
TGGTGA
>FGENESH: 12 2 exon (s) 33345 - 33797 121 aa, chain -
MSKSDNNPYRRYFRCAYAVRKKLENDNHVFKWIDEALINEIEKLEFKNARLEDELKEVRR
NEILLGERVQMKVEKELFDKVDDKVDEVLAEAKASMKKMMLSAVIGCVVLVGIMEVFHKA
W
>FGENESH:[mRNA] 13 8 exon (s) 35637 - 38901 2577 bp, chain +
ATGACGCCTCTGGTAATCGCGCCTCCTCGCTTCTATAAATTCGTACCTCCTCTTCATTAT
CTTCACTTCTTCTGCCGCTACAGCAACATGCGCCCGTGGAAGAGGTTGTCGCGAGAGGAA
AAGGAAAAAGATATTGCGGTGGTTCCGAGTCCCGCTAGGGATGTGACAACCAACGAACCC
CTAGAGGAGTTTGATATGGTTCACCGGGAAGCGCTAAGAGACATGTCCGGTCTCGATCTT
TCGCAGAGGCTTCTCGTTGTTGACGCGGAGAGGGACTTAATAGGAGAAGACGGCGGGGAT
GCGTTGGCTGAGGTGATGAATGATCCGAGGCCACGACGATCCCGAGCAGTGCGTCGCGAA
GCGACTCAGCGTAATCCTGCTCGTCGAACCTGCCGGCGTCTGAGAAGAGAGATATTCTCC
CCAAGGTCCCAACTCGCTATCTCGGGGACGGCGTCTTCGAGAGAGTCGTGGGGAAACGTG
TTGCCGACTAGGTCGACGGTCGAGAGCGTGGAGAAGCTTCTTATAGACTGTGACGCGAAG
GGAGTGACATATCTGATTCCGACCGAGGGACAATGGCCTTGGTCACCTCCGATCAGATAT

CAGTGCGTATACGAGTTTTACTTCCAGAGGGACACAAAGCTTTGGTTCCCGATACCGCGC
CTGATTACGTCCTACGTTTCTCGCCGTGACTTGGCCATTAGTCAGATGGTGAATGGGTCG
TTTCGTTTGGCGGTCGCGCTGTTGGTCATGGCTGCGGAAATTGGCATATCGTTGTGTCCT
CGGGCGTTCGAGGAGCTGACGTCAGTGACTGCACTGGAGTCCGGTCTTTACTCGGTGAAG
ATGAGGCCGAGCTACAACATAATCACTGGTCATCCTGTCAAGACCACTGACTGGCAGCGT
TGGTATTTCTTCGTTAAGTCCGACGAGTACGCCTTTGTCGATCCGCCGGATGATGAGTAC
AGGGTTTTGTGGAATTGGCGTACCGTTAATCATCCATATTTCACTGGAGTTCCGGGTCAA
TTCTTCGACAACGCACGAGCACTGGTTGAGCTTGGGATTTGGAATTGGCCTGATATAAGT
GAGGCTCAGATTCGTAGGTCTCTGCAGCGTATTGATAGAGGTGATTTGGATTCAAAGCTT
CCTTGTCCAGATCGAAAAGGGAAGAAGATATCGGCCTTGTTCACCAAAGCAGAACAACAA
GAGATTACCCGAGCATTGAAGATGAGGGATCTGCCAGACCTTTCTTCGATTATCGAGTTC
AGGCCGGAGGGTCCTTTGCCGGCAAGTGGGCTGATGGCGGTTTTGGACGGGTTTGGATTA
GACGGGACTGTTCCATCTGCCCGCGAGGATCGGCCTGCCGTCTTGCCTGCCTACGTCGCG
GCGCCTGCTCCAGTCGACACTGGGAGGAAGAGGAAGGCGTTCCCTCGGTTACCCCGTCGA
AGAAAAAGAGGAAGAAGAAGACGAAGGTTAATCCGGAGGTCGAGGCGCAAGTCCTCCAAG
AGGTCGATCGTGCGGACGATCGTGCCGAGGACGCATTTGCCTGATGATGACTTGGTTTTC
CGGGCTTCATATCTCGACGCCGTTTGTCAAAGGCAAGCGGCTAATGACTGTATGGACGCC
TTGGTTGTGAAATACGACGCCGAGCTCAAGGCGTCGTTTGTGTGTTTGGGGAAAGCTCAG
GAGGAGGCAGGCCGGGGGACGGAGCGCGTTGGTGTGTTGGAGGCTTCTCTCGGGAAGGCT
GTAGTCAAGAGGGATGAAGCCACTCGTCGTGCCAAGATTTCGTCGATGCATGCCGAGGAT
ATTTTGAGCAAGTTGAAGGCTTCGAGGATCTCGGCCGAGAGACTACGAAAGGAGAGAGAT
CAGCTCGAGCGGGAGAAGGACGAAATTGCTGAGGATCACGCTCGGGAGGCTGCTCGTTTG
AGGGAGTCATGCATTTATGAAGTCACTCGCGACGAGCAAGAGACCACGCCGCCATGGACG
AGGAAATGCCTGCAACTGCTCAAGGGTCAGGGAGAGGCCATCCCGCAAGAGAGGATCGAT
TTCTTCAAGGCCCAAGAAGAGGAGTTGGCTGTTCGGGCTAAGGAGCTTTGTCCCGGAGAG
ATCTCTGAGGCCTTTGTGACAATGTCTCCTCTTGTCCTTGAATCCTCCTTCGTGAACAAG
GAGCTCCTGGCCGCCTTATCGGCTGAGGCCCTTCGTAGCGAGACTCCCGTTGGTGTCGAC
CCGCACGGGTCCAATGTTGGTCTGATAGGCGTTGAAGCCGTCGCCGGTCTTCAGACCTCT
GAGCATGCCCAAGAGGGACGCTCGGGTGGTAGCGTGGGAGCCCCTTCGGTTGGTCTGGGT
AAGGGCGCTTCCGAGGGAAACGGGAAGGACAGTACGGCGGTCATCTCGGATAACATGGAA
GAAGAAGAAGAGATCTCGGGCTTCGATTCCGGCGATGAGAAAGGTCTTCTGGAGAGCGAT
AAGGCCAGACCTATGGGCGAGTACGCGGAGTTGCAGGTTTCTACGACAGGTGCATCTCGC
CCTTCTCCCGGACAAGAAGGAGCTCAGGGAGCCGATCCTGCCGTGCCAAAGGAGTGA
>FGENESH: 13 8 exon (s) 35637 - 38901 858 aa, chain +
MTPLVIAPPRFYKFVPPLHYLHFFCRYSNMRPWKRLSREEKEKDIAVVPSPARDVTTNEP
LEEFDMVHREALRDMSGLDLSQRLLVVDAERDLIGEDGGDALAEVMNDPRPRRSRAVRRE
ATQRNPARRTCRRLRREIFSPRSQLAISGTASSRESWGNVLPTRSTVESVEKLLIDCDAK
GVTYLIPTEGQWPWSPPIRYQCVYEFYFQRDTKLWFPIPRLITSYVSRRDLAISQMVNGS
FRLAVALLVMAAEIGISLCPRAFEELTSVTALESGLYSVKMRPSYNIITGHPVKTTDWQR
WYFFVKSDEYAFVDPPDDEYRVLWNWRTVNHPYFTGVPGQFFDNARALVELGIWNWPDIS
EAQIRRSLQRIDRGDLDSKLPCPDRKGKKISALFTKAEQQEITRALKMRDLPDLSSIIEF
RPEGPLPASGLMAVLDGFGLDGTVPSAREDRPAVLPAYVAAPAPVDTGRKRKAFPRLPRR
RKRGRRRRRLIRRSRRKSSKRSIVRTIVPRTHLPDDDLVFRASYLDAVCQRQAANDCMDA
LVVKYDAELKASFVCLGKAQEEAGRGTERVGVLEASLGKAVVKRDEATRRAKISSMHAED
ILSKLKASRISAERLRKERDQLEREKDEIAEDHAREAARLRESCIYEVTRDEQETTPPWT
RKCLQLLKGQGEAIPQERIDFFKAQEEELAVRAKELCPGEISEAFVTMSPLVLESSFVNK
ELLAALSAEALRSETPVGVDPHGSNVGLIGVEAVAGLQTSEHAQEGRSGGSVGAPSVGLG
KGASEGNGKDSTAVISDNMEEEEEISGFDSGDEKGLLESDKARPMGEYAELQVSTTGASR

PSPGQEGAQGADPAVPKE
>FGENESH:[mRNA] 14 4 exon (s) 39766 - 41639 1644 bp, chain -
ATGTTCACCAAACACGAGGCAACAGCATCTAGCCTTTGGAACCTCAAGCAGTCAAAAGAT
CAAAGCCTCCGAGACTACATGGAACGATTCAAGTCGGTCGTCTCAAGGATTAGCATCCCC
GACCACGTCGCTATCGATACTTTGATGAACACCCTCCTGGTCGAATGTAAATTTCGAGAG
TACTTGTACCGAAGCCCCACCTCGTCACTTCAGGACGCCATCGCTCGGTCACATAACTTC
ATCCGGATGGAGGAGGACACCAAGGCTATCATGGGCAAACTAGGTGTGGCGAAACAGTCG
GCACCCAAAGGCACCGACAATACACGCGTCGAGCCTCGCCAGCATGCCCAGAACGACAAA
AACAATCTCAAGAACGGCCTCCTAGAAACCGACTCTTCGAGCGAACCACCTCGCGAGCCA
AGTACTGCAGTAGTCGCTCAAGTCGACTCAACGACGGGACCGTCTCGAACACCTCCAGAC
CATACCAAGCAATGCAAGTACCATGGAGTCAAAGGTCACGACACGTCTGAGTGCAAAACC
TTGTATGCGCAGTTCCTTTCCGCCCTTGACAGTGGCGAGTTCAAGATCCCTCCGAAACCG
AAAACTGAAAGCAATTGGAGTAGAAACAAGGATAGGAAGAACCAACGGAAACCTCAGGGC
AAACCGCGTTCAACCGATCAGCGACCGAAAGACGTAGAACAGGCTCCCCGACCAACGGAC
AATGACGATACCTCGGCATACGAGGAACAACCTTCGACTCGACAAAGAATCGAACGAAAG
GTTGCCCAGACAATCCACGAATCGCCTAGATCTTCCGACCTCCGATTCGTGATAAATGCC
AACCGAGCGAAGCGAGCATCGACTCATGATCCTCCATTGGAACAACCGAACGGTACGCTC
GTCGTTGATTTGCGAGAACAACTCAATGCCCGAATGGACGACCTGCGCAAGACGCTCGAT
GGCAAAAGGCCACGGATAAACGTAATCATGGGAGGTTCGCCGCCTTGCGGAGATTCGGTT
CGATCTGTCACAGATCACTGTCGACAGGCGATCACGTCACAACGTTGGCCCCAGAAACCA
CCAAGCGATCCCTCGATTTCTTTCTTGCCCGACGATCTACTCGGAGTATGCCTTCCTCAC
AACGATCCCCTTCTCATTGTCCTTGCTGTTGACAAATACGACGTTACGAATGTGCTCGTT
GATACTGGAAGCTCGGTCGACATCATTTTTCGCGAAACTCTCGTGAAAATGGGGATCAAT
CTGAGTGACGTAAAACCGTCTTCCCGAACCCTGACAGGCTTCAATGGATCTTCCGAAGTG
ATCTTAAGAACGATCTGCCTCTCGGTTCGCGCGGAAGGGGTCACACGGATGGTCAAATTT
TCGGTGGTTGGCTCCAAACGCCCCCTACAACGTGATACTCGGAACTCCTTGGCTGCATTT
TATGCGAGCAATCACATCAACGTATCATTAGTGCATCAAATTCCCGGGAATCGACGGCAC
AGTCAAGACGTTGAAAGGAGACCAACAAGCCGCAAGGGATCTTCTAATCATGACCGCAAA
GGTAAATCACTCAGAGGCCCTCGTCAACTCGGTATCGGCGACTCGGACGCTAGCAAAGTC
GTACGAGCAGGCGCCGACCCATGA
>FGENESH: 14 4 exon (s) 39766 - 41639 547 aa, chain -
MFTKHEATASSLWNLKQSKDQSLRDYMERFKSVVSRISIPDHVAIDTLMNTLLVECKFRE
YLYRSPTSSLQDAIARSHNFIRMEEDTKAIMGKLGVAKQSAPKGTDNTRVEPRQHAQNDK
NNLKNGLLETDSSSEPPREPSTAVVAQVDSTTGPSRTPPDHTKQCKYHGVKGHDTSECKT
LYAQFLSALDSGEFKIPPKPKTESNWSRNKDRKNQRKPQGKPRSTDQRPKDVEQAPRPTD
NDDTSAYEEQPSTRQRIERKVAQTIHESPRSSDLRFVINANRAKRASTHDPPLEQPNGTL
VVDLREQLNARMDDLRKTLDGKRPRINVIMGGSPPCGDSVRSVTDHCRQAITSQRWPQKP
PSDPSISFLPDDLLGVCLPHNDPLLIVLAVDKYDVTNVLVDTGSSVDIIFRETLVKMGIN
LSDVKPSSRTLTGFNGSSEVILRTICLSVRAEGVTRMVKFSVVGSKRPLQRDTRNSLAAF
YASNHINVSLVHQIPGNRRHSQDVERRPTSRKGSSNHDRKGKSLRGPRQLGIGDSDASKV
VRAGADP
>FGENESH:[mRNA] 15 1 exon (s) 42047 - 42352 306 bp, chain -
ATGACGACCAGCGATACGAACCCAGAAACTTCGGGCGAGGAGCGAGCGCCTCCACCCGTT
CCACCGCTCTCGTCGGAGTTCATGAGCTCCGTGATGGCACGACTCGCCCACCAAGAAGAA
GTCCAGAAGGCGACAAACGAGCAACTAGCTGCCATCATAGCCTCTCTCAGCGTACCCGCC
GGACAGACGAGCAATCCCCAACCGTTCCGCCGTCACCTCTTCAACACAAACCCTGCAACA
CCAGTGGATGGTCGCGTAGCGAACGAATCCGAGCAAAATGAAGCTCCGTACCCGACGCTT
CCCTAA

>FGENESH: 15 1 exon (s) 42047 - 42352 101 aa, chain -
MTTSDTNPETSGEERAPPPVPPLSSEFMSSVMARLAHQEEVQKATNEQLAAIIASLSVPA
GQTSNPQPFRRHLFNTNPATPVDGRVANESEQNEAPYPTLP
>FGENESH:[mRNA] 16 2 exon (s) 45985 - 46261 144 bp, chain +
ATGGCAGAGGGTCTGGCTTTAAGGGAAGCTTTAGTGAGGTGCAAGGATCAAGGGATAAAG
TCGATTCCAAGATTGAGAAACAGCCATGCGGACCACTTGGCAAAACAATCTTTAGCTTTT
GTATTAGCTGAACCGAGCCATTAG
>FGENESH: 16 2 exon (s) 45985 - 46261 47 aa, chain +
MAEGLALREALVRCKDQGIKSIPRLRNSHADHLAKQSLAFVLAEPSH
>FGENESH:[mRNA] 17 5 exon (s) 47304 - 49862 1674 bp, chain -
ATGGCGTTCCGACAACAAGAAGCACTTTCTCTTACAAAAAAGACAGATCAAGATCCTTCT
CAAGATTTGATTTTCCGGTCCAAACTTCCCGATATCTCTATTCCAAACCACCTTCCTCTC
ACCGACTACATCTTCCAGAAGTTCTCCGGCCACGGAGACGGCGACTCCACCACCACATGT
CTCATAGACGGTGTGACCGGACGTACCTTTACCTACGCCGACGTGCAGATCACTTTACGG
AGGATTGCTGCCGGAATCTACGGGCTGGGTATCCGCCATGGTGACACCGTGATGCTCCTT
CTCCCCAATTCGCCGGAGTTTGCTCTATCTTTCCTCGCCGTGGTTGACCTCGGAGCCGTA
TCGACCTCTGCTAATCCGTTATTTACTCAAACGGAGATCGCAAAACAGGCAAAGGGCTCC
GCCGCGAAGATGATCATCACGAAACCATGTTACGTCCATAAACTAACAAACCTTCAACGC
CTTGGTGTTGTCATCGTTTGTGTAGACGATGGAAATGACGTCGTGTCGTTAGCTGACGGT
TGCGTGAGTTTCACGGAACTTACTCAAGCGGACGAGACAAAGCTGCCTAAACCGGAGATC
TCTCCGGAGGACACGGTGTCGATTCCCTACTCCTCCGGGACCACGGGACTACCAAAGGGA
GTGATGATTACTCATAAAGGGTTAGTTACGAGCGTTGCTCAGAAAGTCGACGGAGAGAAC
CCTAATCTCAATTTCACCGGAGATGACGTCATCATCTGTTTTCTCCCAATGTTTCACACT
TTCACGCACAGCTCGCTGATGCTTTCGGCGATGAGGACCGGTGCGGCGTTCTTAATCTTG
CCGAGGTTCGAGTTGAATCTAGTGATGGAACTGATTCAGAGGTACAAGGTCACCGTAGTT
CCGGTGGTTCCTCCCGTGGTTCTAGCGTTCGTGAAGTCCCCGGAGACGGAGAAGTACGAC
CTGAGCTCCGTGAGGATGATGCTTTCAGGCGCAGCTACGCTCAAGAAGGAGCTTGAAGAC
GCCGTGCGTCTCAAGCTTCCCAATGCCATATTTGGTCAGAGTTATGGAATGACTGAGGCA
GGAACAGTGGCTAACTCATTGGCATTTGCAAAGAACCCGTTTAAAACCAAATCCGGTTCG
TGTGGGACTGTGATTAGAAACGCTGAGATGAAAGTGGTCGACACAATTAGCGGAGTCTCT
TTACCACGCAATAAGCCTGGAGAAATATGCATCAGAGGCGATCAACTCATGACGGGTTAT
TTGAATGACCCGGAAGCTACTGCCCGAACCATAGATAAAGATGGGTGGTTACACACAGGA
GATATTGGGTTTGTGGATGAGGACGATGAGATCTTCATTGTTGATCGGTTGAAGGAACTC
ATCAAATTCAAAGGCTATCAAGTGGCTCCAGCTGAGCTTGAAGCATTGCTTATTTCTCAT
CCTTATATTGAAGATGCTGCTGTTGTAGCAATGACAGATGAAGTAGCTAATGAGGTTCCA
GTAGCGTTTGTGGTCAAATCAGAAGGATATCACATAACCGAAGAAGATGTCAAGAATTTT
GTCAACAAACAGGTGGTTCACTACAAGAGAATCAAGATGGTGTTTTTCGTAAAAGCTATA
CCAAAATCAGCTTCTGGAAAGCTTTTGAGAAAGGTTCTCCGAGCTAAACTGTAA
>FGENESH: 17 5 exon (s) 47304 - 49862 557 aa, chain -
MAFRQQEALSLTKKTDQDPSQDLIFRSKLPDISIPNHLPLTDYIFQKFSGHGDGDSTTTC
LIDGVTGRTFTYADVQITLRRIAAGIYGLGIRHGDTVMLLLPNSPEFALSFLAVVDLGAV
STSANPLFTQTEIAKQAKGSAAKMIITKPCYVHKLTNLQRLGVVIVCVDDGNDVVSLADG
CVSFTELTQADETKLPKPEISPEDTVSIPYSSGTTGLPKGVMITHKGLVTSVAQKVDGEN
PNLNFTGDDVIICFLPMFHTFTHSSLMLSAMRTGAAFLILPRFELNLVMELIQRYKVTVV
PVVPPVVLAFVKSPETEKYDLSSVRMMLSGAATLKKELEDAVRLKLPNAIFGQSYGMTEA
GTVANSLAFAKNPFKTKSGSCGTVIRNAEMKVVDTISGVSLPRNKPGEICIRGDQLMTGY
LNDPEATARTIDKDGWLHTGDIGFVDEDDEIFIVDRLKELIKFKGYQVAPAELEALLISH
PYIEDAAVVAMTDEVANEVPVAFVVKSEGYHITEEDVKNFVNKQVVHYKRIKMVFFVKAI

PKSASGKLLRKVLRAKL

>FGENESH:[mRNA] 18 2 exon (s) 52623 - 53297 408 bp, chain -
ATGTACACAACCTCTGCTTTGCCTATCAGGTATTTGGGATTGCCCCTCACCACAAAAATT
ATGAGTAAGCATGACGATGAACCCTTTCTTATTAAGATCAGAGATCGCTTTCAAAGCTGG
ACAAGTAGATATCTCTCCTTTGCTGGACGCCTCACGCTCATCAATTCGATTATTGAGTTT
CTGCTCAGACAAGAGTCATTTTGGGACGTCAGTGACACAGGATTGGGTTCATGGATATGG
TGGAAGTTACTTAAACTCCGGCCGATTGCTCAACAGTTCATTCGTATGGAGGTTCACAAC
GGCCAAACGGTCAGATTCTGGACAGATCCTTGGCATCCTCTGGGCCGGCTTATAGATATA
ACTGGTGCAGTTGGTACTCAAAAACTTGGTATTCATAGATCAGCCTGA

>FGENESH: 18 2 exon (s) 52623 - 53297 135 aa, chain -
MYTTSALPIRYLGLPLTTKIMSKHDDEPFLIKIRDRFQSWTSRYLSFAGRLTLINSIIEF
LLRQESFWDVSDTGLGSWIWWKLLKLRPIAQQFIRMEVHNGQTVRFWTDPWHPLGRLIDI
TGAVGTQKLGIHRSA

>FGENESH:[mRNA] 19 1 exon (s) 54285 - 55190 906 bp, chain +
ATGCTTGTAAGTTCTTCTCATTCAAAAAGAAATAGACGATCACGTGCATGGGACTACTTT
ACAATAGGAAAAGATGAGAATGGCCAAGAGAGAGCGTATTGTAAGAAGTGTCCCAAAAAC
TATTTGTGGCTTAGAAATAGTGGAACTTCTAACCTGAAACGTCACTCTGAGAAATGCTCA
CTTGGATTAGATGGTGAGAGAAAAGAGAGGAAGTTTGATGAAAAAGTTGCTAGGAAAAAA
TTCAATAGAGTAATTATCCGGCATAACCTTCCTTTCCGTTCAGTTGAATATGAAGAAATT
AGAGATTATCTTAGTTATTTAAACCCAGACTACAAGTGTTATACTAGGAACACTGCTGCA
GCTGATGTGGTTAAAACTTGGGAGAAAGAGAAGCAAAAACTGAAGTCCGAGTTAGAAAAT
ATTCCTAGTAGGATATGTTTAACCTCTGACTGTTGGACTGCTTCAGTCTCAGGAGATGGA
TATATAGCCTTGACTGCACATTATGTAGATGCGAAGTGGGTATTGCATAGCAAAATTTTG
TCATTTTGTAATTTGCTTCCTCCACACACTGGTGAGGTTTTAGCTAGTAAAATTCATGAG
TGCTTGAAGGAGTGGGGAATTGAGAGGAAAGTGTCAACTTTAACATTAGACAATGCTACA
GCTAACGATTGTATGCAAGACATTTTGAAGGATAGGCTGAATTTGGATGATAATCTCTTG
TGTAAAGGAGATTTTTTTCACGTGCGGTGCTGTGCTCACATCTTAAACCTTATAGTACAA
GATGGTCATCTTTTAGTGTCATCAGAAGCATGGCCAAGTCAGTTCTGCAAATGGATAGAA
GTTTTGAGTTCACTAACAAAGTTAGTTTCACAAAATTTCTTGACGCTTGCATATGATTCT
TCCTGA

>FGENESH: 19 1 exon (s) 54285 - 55190 301 aa, chain +
MLVSSSHSKRNRRSRAWDYFTIGKDENGQERAYCKKCPKNYLWLRNSGTSNLKRHSEKCS
LGLDGERKERKFDEKVARKKFNRVIIRHNLPFRSVEYEEIRDYLSYLNPDYKCYTRNTAA
ADVVKTWEKEKQKLKSELENIPSRICLTSDCWTASVSGDGYIALTAHYVDAKWVLHSKIL
SFCNLLPPHTGEVLASKIHECLKEWGIERKVSTLTLDNATANDCMQDILKDRLNLDDNLL
CKGDFFHVRCCAHILNLIVQDGHLLVSSEAWPSQFCKWIEVLSSLTKLVSQNFLTLAYDS
S

>FGENESH:[mRNA] 20 1 exon (s) 58437 - 58751 315 bp, chain +
ATGTGGGAACCAAAATTCGCACTGTCGATGTTAGTTGATTTGGAGGAAAGACAAGTGAAC
CTAGCCTTCCCTGAAGGTCCCGGTTATCTGCTGGGCCACACACGACACAATCAATACGAC
GGAAAGATAAAAAAACGGAATATAAAAATCGTAAAAAGAGATAGAGCAAAAGGTTTTATT
TCCGAATTTGCGTTTAAGCGTGAACAACAGGAAAGAGATCGGCTACAAGAGCTGTCGATG
AGTTCGCTAGTCCAGCCTCCTATATCTAACCTAGTTGAGTCGCAGCTCGTTAGTAAAAAC
GGAAAAAGTGCCTAA

>FGENESH: 20 1 exon (s) 58437 - 58751 104 aa, chain +
MWEPKFALSMLVDLEERQVNLAFPEGPGYLLGHTRHNQYDGKIKKRNIKIVKRDRAKGFI
SEFAFKREQQERDRLQELSMSSLVQPPISNLVESQLVSKNGKSA

>FGENESH:[mRNA] 21 1 exon (s) 59294 - 60118 825 bp, chain -

ATGGAAGCGCCCGGCCTAGCCAGTGCGGGCTCCGGCTTCGCCAGCTGGCCTGGGCGCCGG
CGCCGGCTTCGCCGATGCTGGCTCGGCGCCGGCTTCGCCGATGCTGGCTCGGCGCCGGCT
TCGCCGATGCTGGCTCGGCGCCGGCTTCGCCGATGCTGGCTCGGCGCCGGCTTCGCCGAT
GCTGGCTCGGCGCCGGCTTCGCCGATGCTGGCTCGGCGCCGGCTTCGCCGATGCTGGCTC
GGCGCCGGCTTCGCCGATGCTGGCTCGGCGCCGGCTTCGCCGATGCTGGCTCGGCGCCGG
CTTCGCCGATGCTGGCTCGGCGCCGGCTTCGCCGATGCTGGCTCGGCGCCGGCTTCGCCG
ATGCTGGCTCGGCGCCGGCTTCGCCGATGCTGGCTCGGCGCCGGCTTCGCCGATGCTGGC
TCGGCGCCGGCTTCGCCGATGCTGGCTCGGCGCCGGCTTCGCCGATGCTGGCTCGGCGCC
GGCTTCGCCGATGCTGGCTCGGCGCCGGCTTCGCCGATGCTGGCTCGGCGCCGGCTTCGC
CGATGCTGGCTCGGCGCCGGCTTCGCCGATGCTGGCTCGGCGCCGGCTTCGCCGATGCTG
GCTCGGCGCCGGCTTCGCCGATGCTGGTCCCGGCGCCAGCTCACCCGCCCTGGTCACGGG
CGCCGACGCCAGCTCACCCGCCCTGGTCACGGGCGCCGCGATACGCATATTTACCGACAG
GTGCGACCTTCTCCCGTAAAACCGTCTCGACAACTAGTCCAACTCTATATTTTGTATCTT
TGCTCGAAGACTCACCGTTCAACTCCTCGAAGACATGAATCCTGA
>FGENESH: 21 1 exon (s) 59294 - 60118 274 aa, chain -
MEAPGLASAGSGFASWPGRRRRLRRCWLGAGFADAGSAPASPMLARRRLRRCWLGAGFAD
AGSAPASPMLARRRLRRCWLGAGFADAGSAPASPMLARRRLRRCWLGAGFADAGSAPASP
MLARRRLRRCWLGAGFADAGSAPASPMLARRRLRRCWLGAGFADAGSAPASPMLARRRLR
RCWLGAGFADAGSAPASPMLARRRLRRCWSRRQLTRPGHGRRRQLTRPGHGRRDTHIYRQ
VRPSPVKPSRQLVQLYILYLCSKTHRSTPRRHES
>FGENESH:[mRNA] 22 3 exon (s) 60466 - 62274 1683 bp, chain +
ATGTCTTCAAGATCAAGATCCTCTAAAAGAAATTCTTCTTCTCACTCATCTTCGGGTGAT
TCTCGTGTCGACGAAGTAGTTGCGCCAAAGCACGAGGAAGGAGTTGAGGAAGATACGAGG
GAGGCATACTACAGAGACCTCCGAGGGTGCCGGGCGCGCCATTCTCACCAAGCATGGTCT
CTCCCGAGTACCTCACGATTCTTCGAAACTTTTTACCAAGTTCCGAGTGGTGTTACGCCC
GATTCGACCGCTGGCGAGTGCAAGGAATCCTCCGCAGGGCTTTTTACTTGTTATGAAGCC
TTTCTCGCGTACTGCCGCATGTGGTTCCCGATCCCTGGTGCTATTGTCTGTGCGCTTCGT
CTTTTCGGACTTTTGATCAGCCAGCTCACCGTCCCGTCCTTAGAGAGTTGGCTTGGCGTG
CTAGTCTCAAGTTACGAGTTGGGCATGGACGTTAGTCCTAGCGATTTCGAGGGACTTTGG
TATACTAAGCCAACGTCGATCGAAGGTGCGTATTCCGTGATCCCAAGGAAGAACATGGCC
ATAATTCAGGGGACTACTTCGAACCCCAAATCGTGGTTCGATCGCTTCTTCTTTGTTCGA
ATAGACGGGGAGTCCGTTGAGGAAAGCTGCCTTCACTTATTCCCTCAGGAGTGGAACTTC
GATCGCGGTAACGCGACTAATTCTGCTTTTTGTTTTCGTACTTTGAACGTGCATGAAGAT
AAAACTTTTGTTTATCGTGCAGCGAACAGGACCGTTGCGCACACTCATGCTGATCTTTTC
GTCAAACGAGACCTTCTTCGGGAAAGGCCATTCTTCTGGAGTACCTTTTCCGTTGAGCGG
ATTCGAAGTGCGGTGGAGCTCCATCGATCCCGAGTCACTCTTCAGCCATCCGATGTTCCA
TGCGACGTCGAGCCGATTGCTGTCTTGCCTGTTCGGAGGCGGAGACATAGGTCTAGGAAA
GGCAAAGAGGTCGAATGCGAGATGGTCTCGGGAGATCCTTCGCCGTTAGGAGTAGACCCT
AGCTTTGTTCCGGGGGAAGGGGGTGGAACCAGCGAGTTTCTCCTGCCCAGCAACTTTTTC
GATGGTCTCCCTCAAGCGTTTACTACTGATGAATCACTCGAGGATGACGCGAAGAAGAAA
GTGTTTGCTGAGGGTTCTCGTCTAATCAATGTGGGAATGATGGTGTTCGGAGCGGCGCTC
GATGGAAGCATTCGAGGATCGAGGATCTCTCACTTCAAAGCCGAGGAAGCCGAACGCGAA
CTCTTCCGTTTCCGGAAAGAGGTCAAGGAGCAAAGCCGGAAGCAGGCCAAACTTCATTCT
CGAGCCCTTGTGCGTGCGGAAAGGAGAGGAAGGAGAGCGATTGCTGCTGACATGGCGTGG
AGGGCTGAATTGTTTGCCACCGAGTTCGAGAGCTTGAAGGAAGATCAAGAGTTTGTGGGT
GATTTTCGCGAGTGTCGGGGATCGATCGCTGAGATGGAAGGTCTCATGAGCGAATGTTCT
CACGCCGAATCCTTGGTTCCTCCGATCGAGGGAAGGGTCTGGAAGCTTTGGGACCCTATC
GAGGTTTCGGAGGACACGGTGGAGACCGCAGGTGGAAACACGGAAGGCGTAGATGAAGAA

GTTGACCAGCCTGTGACTTCATTCGGGATCTTTACATCCGAATACCTTGACCTCGATTAC
TGA
>FGENESH: 22 3 exon (s) 60466 - 62274 560 aa, chain +
MSSRSRSSKRNSSSHSSSGDSRVDEVVAPKHEEGVEEDTREAYYRDLRGCRARHSHQAWS
LPSTSRFFETFYQVPSGVTPDSTAGECKESSAGLFTCYEAFLAYCRMWFPIPGAIVCALR
LFGLLISQLTVPSLESWLGVLVSSYELGMDVSPSDFEGLWYTKPTSIEGAYSVIPRKNMA
IIQGTTSNPKSWFDRFFFVRIDGESVEESCLHLFPQEWNFDRGNATNSAFCFRTLNVHED
KTFVYRAANRTVAHTHADLFVKRDLLRERPFFWSTFSVERIRSAVELHRSRVTLQPSDVP
CDVEPIAVLPVRRRRHRSRKGKEVECEMVSGDPSPLGVDPSFVPGEGGGTSEFLLPSNFF
DGLPQAFTTDESLEDDAKKKVFAEGSRLINVGMMVFGAALDGSIRGSRISHFKAEEAERE
LFRFRKEVKEQSRKQAKLHSRALVRAERRGRRAIAADMAWRAELFATEFESLKEDQEFVG
DFRECRGSIAEMEGLMSECSHAESLVPPIEGRVWKLWDPIEVSEDTVETAGGNTEGVDEE
VDQPVTSFGIFTSEYLDLDY
>FGENESH:[mRNA] 23 7 exon (s) 63568 - 66746 2799 bp, chain -
ATGGCGACCGATACAGACAACCCGCAAACGCACGACGGAACTCCTGTCGATGCCAACGCC
ACCAACACTCCAGCTGGAAACGTATCAACGGTCACCGCTGACACCACGATACTGGACCAG
ATGAAAGAAATGTTCGCCTCCGCTCAGAAACAGGTGGACGAACAAGGAAAATGGCGAAGG
CCAAGAGCAAGCCCCGCGAGGGATCACAAGAACAAGACTTCTTCAGGCCAGAGCCCTGAC
GAACCTCTCCAAGCGGGTGTGCAACCGACCACGGAGAATCTTCCACCTCCCGCTGGGGAA
AACGAAGAGAACAACGTCGAGCGAATCAACTTGGACATTAGCGATCGATCCGATCATTCG
GACGATAGCGCTGGCGTTCGTCCTAGAAGGACCGAAGTCAGTCTGCCCGATAGACGGAAG
AACTGGCGGAGGATCAAACTCGGATCCATCGTAATCAGCGCTGACAAGCTCGGAAGAACG
CAGGTAGCAATAGCGAGATCCACGATCTCCGCGAAGTCTCGCAAAACAGGTGGACGAACA
AGGAAAATGGCGAAGGCCAAGAGCAAGCCCCGCGAGGGATCACAAGAGTCCGCAGCGGCG
AAGACTCGATTTGAGACTCGGCAATCGAGCTGCAGCGGGAACAAGACTTCTTCAGGCCAG
AGCCCCGACGAACCTCTCCAGCGGGTGTGCAACCGACCACGGAGAATCTTCCACCTCCGC
TGGGAAAGCGAAGAACAACGTCGGCGAATCAACTTGGACATTAGCGATCGATCCGATCAT
TCGACGATAGCGCTGGCGTTCGTCCTAGAAGGACCGAAGTCAGAAGAACTGGCGGAGGAT
CAAACTCGATCCATCGTAATCAGCGCTGACAAGCTCGGAAGAACGCAGGTAGCAATAGCG
AGATCCACGATCTCCGCGAAGTCTCGCAAAACAGGTGGACGAACAAGGAAAATGGCGAAG
GCCAAGAGCAAGCCCCGCGAGGGATCACAAGAGTCCGCAGCGGCGAAGACTCGATTTGAG
ACTCGGCAATCGAAGCTGCACGCGCAGACAAGACTTCTTCAGGCCAGAGCCCTGACGAAC
CTCTCCAAGCGGGTGTGCAACCGACCACGGAGAATCTTCCACCTCCCGCTGGGGAAAACG
AAGAGAACAACGTCGAGCGAATCAACTTGGACATTAGCGATCGATCCGATCATTCGGACG
ATAGCGCTGACGTTCGTCCTAGAAGGACCCGAAGTCAGTCTGCCCGATAGACGGAAGAAC
TGGCGGAGGATCAAACTCGGATCCATCGTAATCAGCGCTGACAAGCTCGGAAGAACGCAG
GCAACTAGTGCCGCCCCCGAGATCGATAGACTTCTCGAGGAAGTACGAAAAACGCCGTTC
ACCACTCGGATCACAGAAACGCAAATCTCAGACCCTGGAAAGATCAGGATTCCCGTCTAC
GACGGCACCACAGATCCAAAGCCACACTTACAGTCCTTCCAGATCGCGATGGGAAGATGC
AAACTCCCGGAGCGTGAACGAGACGCCAGTCACAGCCTTCTCTTTGTCGAGAATCTCAGG
GGTGGCGCGCTCGAATGGTTCTCTCGGCTCAAAAGAAACTCCATCGGAAGTTTCCGCCAA
CTAGCCTCGGCGTTTCTCAAACAGTATTCCATGTTTATGGATAGAGAGACTTCCGACGTG
GACTTATGGAGTCTGGCTCAAAAGGAAGACGAATCACTTTGCAACTTCATGAGAAGGTTT
AAGCTGGTGATGGCCAGAGTGACGGGTATCAGCGACAAAGTCGCAATCGACGCCCTGAGA
AAAACTCTCTGGATACCCTCCACAAAGCAACGGACTTCATCCTCAAAGAAGAAACCCTCT
CGTAACGACAAGTACGTCCATCACGAAGGGGAAGACGTCCAAGGCGAACATAACTACGCC
ATCAACTCGGAGCAGGGCAAAACCTCAGGAAACACCTGGACCAGGAACCAGTACAAGGAT
AATTCCTACTGCGAGTTCCACGACACCAGAGGTCACCCCACCGCGAACTGCAAGGTTCTC

GGTGCCAGGATTGGCGCAAAACTCCTCGCCGGCGAACTGTCGAAGGTCAAGAGTATAAAA
GACTTGCTCCTGGAGTCCGACCGCCCAAAAACCAACAAAACTGTCTCCGAGAACCACGCG
CCTGAAAGCCAATCGGGTGAGAAGCGCGGAAGGAGACAGGACGACCAAGGGAACAACAGT
AGTCGCCAGAGGGTAAATATGATCATCAGAGGATCGCAGTTCTACCAAGACTCGGTCTCG
TCGATCAAAGCCTACGGGCGAAAAGCCGAGACGAGTTCCAACTGGCTTCCAGATAGCGAT
ATTCCCAACCACGCGATCATTTTCGAGGAACAAGAAACTGTCGGGATCGAGAAACCTCAT
TACGATCCTCTAGTCATCGACCTGGTGATCCAGGATCTCGGAGTTGGCCGCATCCTTGTC
GATACAGGAAGTACGGTCAACGTCATCTTCTGCGACACCCTCTATAGGATGACCAACGCA
CTAGGAGAAGTCATCCCAGAGCCAAAACCTTTGACTTGGTTTTCAGGCGTCACCTCCATG
ACCCTCGGGCGTATCAAGCTCCCGGTCATGGCTAAGGAAGTTACAAAGATCGTCGAGTTA
GCAGTAGTCGATAACCCGGCTATCTACAACGCCATCATGGGAACCCCTTGGATCAACTCC
ATGAAGGCAGTACCGCCCACATATCACCTCGGCATCAAGTTTCGACTCCAACCGAACCGC
AGTAACTGGGAAGTCGAACAGTCAAGACTCGCTACCTAG
>FGENESH: 23 7 exon (s) 63568 - 66746 932 aa, chain -
MATDTDNPQTHDGTPVDANATNTPAGNVSTVTADTTILDQMKEMFASAQKQVDEQGKWRR
PRASPARDHKNKTSSGQSPDEPLQAGVQPTTENLPPPAGENEENNVERINLDISDRSDHS
DDSAGVRPRRTEVSLPDRRKNWRRIKLGSIVISADKLGRTQVAIARSTISAKSRKTGGRT
RKMAKAKSKPREGSQESAAAKTRFETRQSSCSGNKTSSGQSPDEPLQRVCNRPRRIFHLR
WESEEQRRRINLDISDRSDHSTIALAFVLEGPKSEELAEDQTRSIVISADKLGRTQVAIA
RSTISAKSRKTGGRTRKMAKAKSKPREGSQESAAAKTRFETRQSKLHAQTRLLQARALTN
LSKRVCNRPRRIFHLPLGKTKRTTSSESTWTLAIDPIIRTIALTFVLEGPEVSLPDRRKN
WRRIKLGSIVISADKLGRTQATSAAPEIDRLLEEVRKTPFTTRITETQISDPGKIRIPVY
DGTTDPKPHLQSFQIAMGRCKLPERERDASHSLLFVENLRGGALEWFSRLKRNSIGSFRQ
LASAFLKQYSMFMDRETSDVDLWSLAQKEDESLCNFMRRFKLVMARVTGISDKVAIDALR
KTLWIPSTKQRTSSSKKKPSRNDKYVHHEGEDVQGEHNYAINSEQGKTSGNTWTRNQYKD
NSYCEFHDTRGHPTANCKVLGARIGAKLLAGELSKVKSIKDLLLESDRPKTNKTVSENHA
PESQSGEKRGRRQDDQGNNSSRQRVNMIIRGSQFYQDSVSSIKAYGRKAETSSNWLPDSD
IPNHAIIFEEQETVGIEKPHYDPLVIDLVIQDLGVGRILVDTGSTVNVIFCDTLYRMTNA
LGEVIPEPKPLTWFSGVTSMTLGRIKLPVMAKEVTKIVELAVVDNPAIYNAIMGTPWINS
MKAVPPTYHLGIKFRLQPNRSNWEVEQSRLAT
>FGENESH:[mRNA] 24 1 exon (s) 67562 - 68548 987 bp, chain +
ATGAATCGAAATCCATTCGTCTACCAAACTTTGGTGGTCTTCCGAAGCCTTCCCGATTTA
TTTCGACCAGAACGAAATAGGGTTCTCAGGTTTCATGTCTCCGAGGTGTTGAACGGTGAG
TCTTCGAGCAAAGATACAAAATATAGAGTTGGACTAGTTGTCAAGACGGTTTTGCAGGTA
GAGCTGGCGCCTGGCACCCGTGACCAGGACGGGCAAGCTGGAGCCCGGCGCCCGGGGCCA
GCACGGCGAAGCTGGCGCCGGGGCCAGCTCACGGGCGAAGCTGGCGTCCGGCGCTGGGAC
CAGCAGGCGAGCTGGCGCCGGGGCCAGCACGGCGAAGCTGGCGCCGGGGCCAGCACGGCG
AAGCCGGCGCCGGGGCCAGCACGGCGAAGCTGGCGCCAGGACCAGCAGGCGAGCTGGCGC
CGGGGCCAGCACGGAGAGCCGGCGCCCGGCAGCCGGGCCAGCATGGGCGAAGCTGGCGCC
AGGACCAGCAGGCGAGCTGGCGCCGGGGCCAGCACGGCGAAGCTGGCGCCAGGACCAGCA
GGCGAGCTGGCGCCGGGGCCAGCACGGCGAAGCTGGCGCCGGGGCCAGCACGGCGAAGCT
GGCGCCGGGGCCAGCAGGCGAGCTGGCGCCGGGGCCAGCTCACGGGCGAAGCTGGCGTCC
GGCGCTGGGACCAGCAGGCGAGCTGGCGCCGGGGCCAGCACGGCGAAGCTGGCGCCAGGA
CCAGCAGGCGAGCTGGCGCCGGGGCCAGCACGGCGAAGCTGGCGCCGGGGCCAGCACGGC
GAAGCTGGCGCCAGGACCAGCAGGCGAGCTGGCGCCGGGGCCAGCACGGCGAAGCTGGCG
CCAGGACCAGCAGGCGAGCTGGCGCCGGGGCCAGCAGGCGAGCTGGCGCCGGGGCCAGCA
GGCGAGCGGCCCGGCGCCGGGGCCAGCACGGCGAAGCTGGCGCCGGGGCCAGCAGGCGAG
CGGCCCGGCGCCCGGCGCCGAGGCTAG

>FGENESH: 24 1 exon (s) 67562 - 68548 328 aa, chain +
MNRNPFVYQTLVVFRSLPDLFRPERNRVLRFHVSEVLNGESSSKDTKYRVGLVVKTVLQV
ELAPGTRDQDGQAGARRPGPARRSWRRGQLTGEAGVRRWDQQASWRRGQHGEAGAGASTA
KPAPGPARRSWRQDQQASWRRGQHGEPAPGSRASMGEAGARTSRRAGAGASTAKLAPGPA
GELAPGPARRSWRRGQHGEAGAGASRRAGAGASSRAKLASGAGTSRRAGAGASTAKLAPG
PAGELAPGPARRSWRRGQHGEAGARTSRRAGAGASTAKLAPGPAGELAPGPAGELAPGPA
GERPGAGASTAKLAPGPAGERPGARRRG
>FGENESH:[mRNA] 25 4 exon (s) 68914 - 70668 1500 bp, chain +
ATGATCGAAGATGTTGCGGCTGATGTTGGGGGTTGGATCGGAAAAGAACGGCCACGGCCT
AGCCCCGGGCTCCGGCGCAAGCACGCCAGGCCTGGTCCTGGCGCTGGCGCCGGGCCAGCT
CACGGGCGAAGCTGGCGCCGGGCCAGCACGGCGAAGCCGGCGCCGGTGCCTGTGGCCAGC
ATCGGCGAAGCCGGGCGCCGGTGCCTGTGGCCAGCATCGGCGAAGCCGGCGCCGGGCCAG
CTCACGGGCGAAGCTGGCGTCCGGCGCTGGGACCAGCATCGGCGAAGCCGGCGCCGGGCC
AGCACGGCGAAGCCGGCGCCGGTGCCTGTGGCCAGCATCGGCGAAGCCGGGCGCCGGTGC
CTGTGGCCAGCATCGGCGAAGCCGGCGCCGGTGCCTGTGGCCAGCATCGGCGAAGCCGGG
CGCCGGTGCCTGTGGCCAGCATCGGCGAAGCCGGGCGCCGGTGCCTGTGGCCAGCATCGG
CGAAGCCGGCGCCGGGCCAGCACGGCGAAGCCGGGCGCCGGTGCCTGTGGCCAGCATCGG
CGAAGCCGGGCGCCGGTGCCTGTGGCCAGCATCGGCGAAGCCGGGCGCCTCGGCTACCAG
GCTAGGCCGCGGTCGCCCTTCTGGTGTCCGAACTACGATTCGATCATCCCTTCGACGCTT
CGCCGAAAGAAATTCTTTAAGGAAACTTTCAAAGGTAAAACAAACGAGGTTACTGGAAAA
TGGAGTCAAGCTCTAGCAAAAGTGGCCACACTCGCTGGTTACCATTCAAATAACTGGGAT
AATGAGGCAAATATGATCAAGATGTTGCGGCTGATGTTGGGGTCAAAAACGGTTATCTCG
AAAACAACGGCTAAGATCATTTCCTGTCAACGGACTATCCGCAAAAACAATCGACAAAGG
TTGATCGAAAAGAACGGCCACGCTAGCCTCGGCTCCGCGCGCACGCCAGGCCTGGTCCTG
GCTGGCGCCCGGCTTCGCCGTGCTGGTCGAGCGGCGCCTGCATCTATGTCCAAGTCCCTG
GTGCCGGCGCCAGCATCCATGTCCTGGCCCCGGCGCCGGGCCAGCACGGCGAAGCCGGGC
GCCGGTGCCTGTGGCCAGCACGGCGAAGCTGGCGCCCGGCTTCGCCGTGCTGGCCAGGCT
GGCGCCCGGCTTCGCCGTGCTGGTCGAGCGGCGCCTGCATCTATGTCCAAGTCCCTGGTG
CCGGCGCCAGCATCTATGTCTAAGTCCCGAGCGCCAGCGCCAGCATCCATGTCCAAGTCC
CTGGTGCCGGCGCCAGCATCTATGTCCAAGTCCCTGGTGCCGGCGCCAGCATCTATGTCT
AAGTCCCGAGCGCCAGCGCCAGCATCCATGTCCAAGTCCCTGGTGCCGGCGCCAGCATCC
ATGTCCAAGTTCCGGGAGCCGGTGCCAGCATCCATGTCCTGGCCCCGGGCGTCGACGCGT
CCAGCCTGTTCCCAGCCCGTCGGATTTAGATCGCACCTTGTGACTTCCGTGAAGTCGTAG
>FGENESH: 25 4 exon (s) 68914 - 70668 499 aa, chain +
MIEDVAADVGGWIGKERPRPSPGLRRKHARPGPGAGAGPAHGRSWRRASTAKPAPVPVAS
IGEAGRRCLWPASAKPAPGQLTGEAGVRRWDQHRRSRRRASTAKPAPVPVASIGEAGRRC
LWPASAKPAPVPVASIGEAGRRCLWPASAKPGAGACGQHRRSRRRASTAKPGAGACGQHR
RSRAPVPVASIGEAGRLGYQARPRSPFWCPNYDSIIPSTLRRKKFFKETFKGKTNEVTGK
WSQALAKVATLAGYHSNNWDNEANMIKMLRLMLGSKTVISKTTAKIISCQRTIRKNNRQR
LIEKNGHASLGSARTPGLVLAGARLRRAGRAAPASMSKSLVPAPASMSWPRRRASTAKPG
AGACGQHGEAGARLRRAGQAGARLRRAGRAAPASMSKSLVPAPASMSKSRAPAPASMSKS
LVPAPASMSKSLVPAPASMSKSRAPAPASMSKSLVPAPASMSKFREPVPASMSWPRASTR
PACSQPVGFRSHLVTSVKS
>FGENESH:[mRNA] 26 3 exon (s) 71540 - 73907 2172 bp, chain +
ATGTCCACTCCAGTAGCCAGCGATGTTGTTTCCTTCGAGGATCGGGCAACGGCCGACCAG
GCCAAACCCCACAACGATCTCCTTGTAATTAAACTAACGATCCAGGACATCGACGTAGCG
AGAGTGTTAGTCGACACCGGATGCTCGGCCTACATTATCTACAATAGTACCCTCGAGAGA
ATGGAGGTCGACCTGTGCGCCGTCACGGAAGAACCTAGCCCGATATTCGGACTCTCGGGA

GATGCTACTATGACTCTCGGCTCGATCAGCCTCCTGGTTAAAGCTGGGAGCGTCGCCAAA
ATCACGGAATTCTTAGTCGTCAACCGTCCAACATCGTATAACGCGATCATCGGAAATCGC
AGGATGGCGCAAGTATGTTTCGCTGCCGAACTGAAAAGAAAAAACTCGGCAGTCGAAGCC
TCCCAAAGCAAAAAAGTAAAGCCTACTCCCGATGAGAATGCCCCAGAACAAGACTCGGAC
ATCTTCTGGCAATCTCAGAGGATCGAAGCCCTAGACGGGAAGCGCGAACCAACTTGCGAA
CCAGTAATCTCGGTCTGCCTAGACAAATCGTTCCCAGAACGATGCGTCGAGATCGGATCC
AACCTCCGCGAACCAGTAATCTCGGTCTGCCTAGCATTCGCGGTTGTAAAGTCGGCGAGA
AAACTCCGGCCTTATTTTCAGTCGCATACCATTGTAATCCTCACCACCTTCCCCTTGCGA
ACGATTTTGCACAGTCCGAGTCAGTCAGGACAACTCGCAAAATGGGCAATCGAACTAAGT
GAGTACGACGTAGAGTACCGTCCAAGAACCTGCGCAAAATCTCAAGTATTGGCAGACTTC
TTGGTGGAATTGCCCATGGGAGATATGACAAACACGGAACCGAACTTAACTTGGATCCTT
CATGTCGATGGATCATCGTCCAAACAAGGGTCCGGGAACGGAATCCGGCTCACATCTCCG
ACTGGCGAAATCTTGGAACAGTCGTTCCGATTGGATTTCCATGCATCAACCAACGAGGCC
GAGTATGAGGCACACATTGCGGCCTTACGACTATCCCAAGGGCTGAAGATCCGCAACATT
CACGCTTACTGTGACTCTCAGTTAGTCGCAAGTCAGTACAGCGGGGAATATGAAGCAAGG
GACGAAAGAATGGGTGCATATCTTAAACTCATCCAAGATCTCTCCCGAAACTTCGACCAC
TTCGCCCTCACTAGGATTCCTCGCTCGGAAAACACTCAGGCGGATGCCTTGGCCGCACTC
GCGTCAAGTTCGGATCCTGGACTAAGTCGGGTAATCCCCGTCGAGTTCATTGAACATCCG
AGCATCAGACCTCCAGTGATCGCCAATCTTATTCGAGCACAAATTGAAAACGCGGGGGAA
ATAGATGACCCACCGGAAGAAAAAGTGGATCAGTCAGAATACGGCTGCGACAGCCCTTGG
CTGGAACCAATCCGAGCATACATAATCGACGGAACACTTCCCACCGAAAAATGGGCAGCC
CGCAAAATCAAGACCCAGGCCGCACGATACGTAACGGTAGAAGGAGAAATCTACAAATGG
AGATTCTCCTGCCCACTCATGACCTGCGTTGAAGGCGAAAAAGCGAGAAGAGTAATGGAG
GAGGTCCATTCCGGGTCGTGCGGAAACCATTCCGGCGGAAGATCTCTTGCAGTCAAAATA
AAAAGCCACGGTTATTACTGGCCAACTATGATCAAAGACTGCGAGAAGTTCGCCGAAAGT
GCGAAAATGCCAAAGGCACGCGACCGACGATCCATCAGCCCGCTGAAGTCCTCTCGCCCA
TCTCATCTCCTCAGATCCTTTATGCGATGGTCCATGGATATTGTCGGGCCATTCCATAAC
TCGAAACAAAAACGTTTCTCCTGGTCCTCACGGATTTCTTCTCAAAATGGGTGGAGGCAG
ACTCCAACGCAAGTATCAAAGACGCATTCTGCGAGAAGTGGAAGATACGACTTACCAAGT
CGACTCCCAGATATCCGCAATGCAACGCCAGGCAGAAACCATCAACAAGACCGCCCTGAC
GGACTGAAAAGCTTTAGACGCCAAAAGGGCTGGGCGAGGAGCTCGAAGGAGTCCTTTGGT
CGCATCGTACAACCCCGAGATGGGCTACGGGTGAAACCTCATTCGCCCTCGTTTACGGAA
CGGAATGCATGA
>FGENESH: 26 3 exon (s) 71540 - 73907 723 aa, chain +
MSTPVASDVVSFEDRATADQAKPHNDLLVIKLTIQDIDVARVLVDTGCSAYIIYNSTLER
MEVDLCAVTEEPSPIFGLSGDATMTLGSISLLVKAGSVAKITEFLVVNRPTSYNAIIGNR
RMAQVCFAAELKRKNSAVEASQSKKVKPTPDENAPEQDSDIFWQSQRIEALDGKREPTCE
PVISVCLDKSFPERCVEIGSNLREPVISVCLAFAVVKSARKLRPYFQSHTIVILTTFPLR
TILHSPSQSGQLAKWAIELSEYDVEYRPRTCAKSQVLADFLVELPMGDMTNTEPNLTWIL
HVDGSSSKQGSGNGIRLTSPTGEILEQSFRLDFHASTNEAEYEAHIAALRLSQGLKIRNI
HAYCDSQLVASQYSGEYEARDERMGAYLKLIQDLSRNFDHFALTRIPRSENTQADALAAL
ASSSDPGLSRVIPVEFIEHPSIRPPVIANLIRAQIENAGEIDDPPEEKVDQSEYGCDSPW
LEPIRAYIIDGTLPTEKWAARKIKTQAARYVTVEGEIYKWRFSCPLMTCVEGEKARRVME
EVHSGSCGNHSGGRSLAVKIKSHGYYWPTMIKDCEKFAESAKMPKARDRRSISPLKSSRP
SHLLRSFMRWSMDIVGPFHNSKQKRFSWSSRISSQNGWRQTPTQVSKTHSARSGRYDLPS
RLPDIRNATPGRNHQQDRPDGLKSFRRQKGWARSSKESFGRIVQPRDGLRVKPHSPSFTE
RNA
>FGENESH:[mRNA] 27 4 exon (s) 74945 - 77895 1770 bp, chain -

ATGGAGAAAAACCTAGTTAACCCGATCTTCCTTGAAGCTAAGGTGCTACAACTTCACGGA
AGCCACGAGGTGCGATCTAAATCGGACGGGCTGGGAACAGTCTGGACACGTCGGCGCCCG
GGGCCAGGACATGGATGCTGGCGCCGGCGCCCGGGACTTGGACATGGATGCCGGCGCCGG
CGCCGGGACTGGACATGGATGCTGGCGCCGGCGCCGGGACTGGACATGGATGCTGGCGCC
GGCGCCGGACTTGACATGGATGCTGGCGCCGGCGCCGGGACTGGACATAGATGCTGGCGC
CGGCGCCGGACTTGGACATGGATGCCGGCGCCGGCGCCCGGGACTTGGACATGGATGCTG
GCGCCGGCGCCCGGGACTTGGACATGGATGCTGGCGCCGGCGCCCGGGACTTGGACATCG
ATGCTGGCGCCGGCGCCGGACTTGGACATGGATGCTGGCGCCGGCGCCGGACTTGGACAT
GGATGCTGGCGCCGGCGCCGGACTTGGACATGGATGCTGGCGCCGGCGCCGGACTTGGAC
ATGGATGCTGGCGCCGGCGCCGGACTTGGACATGGATGCGGCGCCGGTGCCGGGGCTAGG
CCGTGGCCATTCTTTTCCGATCCAACCTTTGTCGATTATTTTGGCGGAAACGCTGCGAAA
GGCTCGAACCGGGTGAGAGCCGAAGCACCTAGCGTTCCTTCTTCGCCTGTGCCTTCGTCC
CCATCCCCTTGTTCCGAGGTTCCGCCCGATGCTCCCTTGGCCGTGGGAAGGGCAGTCGTT
CCCGAGCAGATTCCCGACAGTCCACGCCTCCGGCTTCGAAGATCGTTTTGGGACTCCCAG
CACCCAGTGCTGCACTGTTGCCTAAGAATTCGTTCGAGGGCGGTTACCGGAGGCGATGAA
GAGGAAGCGCTAAGCGGCGAAGCGAGCCTTCGGGACTGTTGTCGCAACATCGTTCCAAGG
TTTGATTTTGATTTATCCGTAATTATTCTTTTCTTTTCTTTTGCGATGATCCTTACCCTT
TCTTTTTGCGGACAGTTTGTGTCGCTGATTGATGGGATGCTTGGTGACTGCGGATCGGAG
GTTGCGCGTCTGTCAAAGGAGTTAGAGTCGTCGCAGGAAGCGTTGAAACGTACCGAGGCC
GCATTGCAGACCATCGAAAACGCCCATGCTGCCCAGACGTCTCAGCTCGAAGTTCGAATC
GGCGACCTCGAACGCGATCTCGGAAAGATCGCGAGTTCGTTGCTCGAAGTGAAGAAAGAG
AAGAGACACAAATCCTCGGAAATCCGTCGTCTCCAACAAAAGATCCAGGATTACGAAGAG
ATGAGTGCTCGCAAGTTGAAAGAGACCGTCGATCCCCGCGGCGACTTCTTCGCTCGTTTG
ACGAGGATGGCCAGTTTGTTCGAGTCTCTGATAGCTGTCCGTGAGAGGGACTTAGCTTTG
GCGGGAATCGAAGGGAGCTTGAGCGAGCTTCAGCTGCTTAGGGGCAACGAGGCCCTATCT
CTGGATTCCGAGGAAGCCAGGCTGCTGTCTTGCAAGGGAGAGTTGGCGGTTTCCGAAGGG
GACTTTGACTCGATTCTCGCTGGTCTGAAGTCCGAGTGCACCCTTGTACCGGATTCGGAG
GGCTCGGAGGATCAGGTTCAGGCAGCAGAGGATCGTGAGGCTGGCGCGGATGAGGCATGC
AGTGGAGGTGTGACCGAGGGAGTCGATGGCGAGGCTGTTTCCGCTGCGGGTGAGTTTGAG
GATGAAGGCGACGCTCCGAGGAGCATCTAG
>FGENESH: 27 4 exon (s) 74945 - 77895 589 aa, chain -
MEKNLVNPIFLEAKVLQLHGSHEVRSKSDGLGTVWTRRRPGPGHGCWRRRPGLGHGCRRR
RRDWTWMLAPAPGLDMDAGAGAGLDMDAGAGAGTGHRCWRRRRTWTWMPAPAPGTWTWML
APAPGTWTWMLAPAPGTWTSMLAPAPDLDMDAGAGAGLGHGCWRRRRTWTWMLAPAPDLD
MDAGAGAGLGHGCGAGAGARPWPFFSDPTFVDYFGGNAAKGSNRVRAEAPSVPSSPVPSS
PSPCSEVPPDAPLAVGRAVVPEQIPDSPRLRLRRSFWDSQHPVLHCCLRIRSRAVTGGDE
EEALSGEASLRDCCRNIVPRFDFDLSVIILFFSFAMILTLSFCGQFVSLIDGMLGDCGSE
VARLSKELESSQEALKRTEAALQTIENAHAAQTSQLEVRIGDLERDLGKIASSLLEVKKE
KRHKSSEIRRLQQKIQDYEEMSARKLKETVDPRGDFFARLTRMASLFESLIAVRERDLAL
AGIEGSLSELQLLRGNEALSLDSEEARLLSCKGELAVSEGDFDSILAGLKSECTLVPDSE
GSEDQVQAAEDREAGADEACSGGVTEGVDGEAVSAAGEFEDEGDAPRSI
>FGENESH:[mRNA] 28 4 exon (s) 78014 - 80905 2382 bp, chain +
ATGAACAAGATTTCGCGGGTTTTGCGCAGAGATTTGGATGAAGTGAGGATGATAGGGATT
TTGGGGCCGGCTGGGATAGGCAAGACCACCATTGCTAGGTGTCTGTTCAATCAACTATCC
CATACTTTTCAATACAGTGTCTTTATGATGAATGTCAAAGCAATGTATACGCCACCGGTT
TGTTCAGACGACTACAATGTGAAGTTGCATTTGCAGCAAAAGCTTTTGTCTCAACTACTC
AACCAGAAGGAGGATCTCAAGATCTCTCACTTGGGAGTTGCACAAGAAATACTTAAAGAT
AAGAAAGTTCTTGTTGTTCTTGATAACGTTGACCGGTTAGTTCAACTAGAAGCCATGGCC

AAAGAAACTCGGTGGTTTGGTCATGGAAGTCAGATAATCATTACGACGCAAGATCGAAAG
ATCCTGGAGGCACACGAGATCTGTCATATTTACAAGGTTGATTTTCCATCAACTTGTGAA
GCTATTCAGATGTTTTGTATGTATGCTTTTAGTCAAAAGTCCCCTAAAGATGGTTTTGAG
AAGCTTGTATGGGAAGTTACAGGACTCGCCGGTAACCTCCCTTTGGGACTAAAGGTTATG
GGATCTTATTTTCGTGGAATGTCCAAGCCGGAATGGGAAAGTGAACTACCGAAGCTAAGG
ATGTGCCTTGATGGAGAAATTGAGAGCATTTTGATGTTCGGTTATATTGCATTATCTCAT
GAAAATAAAGATTTGTTTCTTCATATAGCCTGCTTTTTCAACTTTGAAAGGATAGAGAAA
GTGGTAGAGCATCTTTCAAATGGATTTTCTGATGTGAGGCAACGGCTTAACGTCTTAGCT
GAGAAGTCTCTCATATCTTTGGAAATTGGATGGGTGAGAATGCATGATCTGCTAGTCCAG
CTGGGTAGAGATATTGTCAGGAAACAGTTCATTGAGCCTGGACAATGCCAGTTTCTGGTC
GATGAAAGAGAGACTTTTGAAGTACTTGCTGATGATGCAGCGGGTAGTAGAAGTGTTATT
GGCATAAAGTTCAGGGGAGACAATATAAATCCGCTTAGCAATCTCAAGTGGGTGAATTTG
TCGTGTTCAGAAAATTTGAAGGATGTTTCTAGTCTCTCAACTGCCACTAGTCTAGAGGAA
TTAGATCTCACTGGATGTTCAAGTTTAGTGGAGCTTCCTTCCTCTATTGGGAATGCCATT
CATCTAAAAAGATTGGATCTCGGTGGATGTTCAAGTTGGTTTGGTGGAGCTCCCTCATGC
ATTGGGAATGCCATAAAAAACCTCGAGGATTTGGATTTTAGTGATTGCTCAAGTTTAGTG
GGAGTCCCTTTCTCTATTGGAAACGCCTCTAATCTCAAGCGTTTGTATTTTATTAGATGC
TCAAGTCTGGTGGAGCTTCCTGCCTCTATTGGAGACCTCCATGAACTAGAATCTTTGATT
TTGAGAGAATGTTCCAAGCTCGAGGTTCTTTCGGTCAACATCAATTTGAAATCTCTCACA
AAACTTGATCTCACTGATTGCTCCTTGATGAAAAGTTTTCCTGAGATCTCGACCAACATC
GAATATATGTATCTCACTGGAACTGCGATTAAACAAATGCCTTCATCAATCAGCTTGTGG
CCTCGTCGTCTAGATGAATTGCATATGTCATACAACGAGAACCTCGAGGAGTTCCCTCAT
GTTCTTGACATCATGACAGATTTGGTCATGAGCAACACAGAAATACAAGAGATTTCTCCT
TGGATCAAGAGAAGCTCTCGTCTTCGTCGACTTGTACTCAACGGATGCAAAGACCTCTTA
TCACTCCCACAGCTTCCAAGTTCATTATCAGAGCTAGACGCAGAAAACTGCGAGTCTCTG
GAGAGACTAGACTGCTCCTTTCTTAATCAAAAGATTGCTCTCAACTTCGCCAACTGCTTC
AAACTAAACAAAGAAGCGAGAGATGTCATCATCAAGACATCGACTAATGAAGTTACGATG
TTACCCGGTAAAGAAATGCCTAACTACTTCAATTATCAAGCTAATGGAGGTTCCCTAGTA
ATGAAGTTGAACGAGAGGCCTTCACGTTCACCAATAATATGGAAGGCTTGCATATTGCTG
GTTAGTAAAGATGAGGTTGAAGCTGGTAAAGGAAAAAGGATGGATGTCGATCATGGGATC
AAACAGAATAGCCTCGATGTCCCGTGCAGTCCATTTTACCACACTCTGTATCACCCTTTA
ACGGAACATCTCTACATCTTTGAATTCGAAGCAGAGGTGACTTCAGACAAACTTTGCTTA
AAGTTTGAGGTCAAGTTACCCAAGTTTTTCTCTGAGTGTGAGGCCGACAGTGAGGAATGG
ATGATAAAAGAAAGTGGAGTGCATTACCTTAATACTAGTTGA
>FGENESH: 28 4 exon (s) 78014 - 80905 793 aa, chain +
MNKISRVLRRDLDEVRMIGILGPAGIGKTTIARCLFNQLSHTFQYSVFMMNVKAMYTPPV
CSDDYNVKLHLQQKLLSQLLNQKEDLKISHLGVAQEILKDKKVLVVLDNVDRLVQLEAMA
KETRWFGHGSQIIITTQDRKILEAHEICHIYKVDFPSTCEAIQMFCMYAFSQKSPKDGFE
KLVWEVTGLAGNLPLGLKVMGSYFRGMSKPEWESELPKLRMCLDGEIESILMFGYIALSH
ENKDLFLHIACFFNFERIEKVVEHLSNGFSDVRQRLNVLAEKSLISLEIGWVRMHDLLVQ
LGRDIVRKQFIEPGQCQFLVDERETFEVLADDAAGSRSVIGIKFRGDNINPLSNLKWVNL
SCSENLKDVSSLSTATSLEELDLTGCSSLVELPSSIGNAIHLKRLDLGGCSSWFGGAPSC
IGNAIKNLEDLDFSDCSSLVGVPFSIGNASNLKRLYFIRCSSLVELPASIGDLHELESLI
LRECSKLEVLSVNINLKSLTKLDLTDCSLMKSFPEISTNIEYMYLTGTAIKQMPSSISLW
PRRLDELHMSYNENLEEFPHVLDIMTDLVMSNTEIQEISPWIKRSSRLRRLVLNGCKDLL
SLPQLPSSLSELDAENCESLERLDCSFLNQKIALNFANCFKLNKEARDVIIKTSTNEVTM
LPGKEMPNYFNYQANGGSLVMKLNERPSRSPIIWKACILLVSKDEVEAGKGKRMDVDHGI
KQNSLDVPCSPFYHTLYHPLTEHLYIFEFEAEVTSDKLCLKFEVKLPKFFSECEADSEEW

MIKESGVHYLNTS
