# Supplementary material for: Genome-Wide Identification Analysis of the 4-Coumarate: Coa Ligase (4CL) Gene Family in Brassica U’s Triangle Species and Its Potential Role in the Accumulation of Flavonoids in Brassica napus L
Source: Plants (Basel). 2025 Feb 26;14(5):714. doi: 10.3390/plants14050714 (PMC11902127; doi:10.3390/plants14050714)
Supplement: Supplementary file 1 [file plants-14-00714-s001.zip › Supplementary Materials/Figure S3/Bca4CL6.pdf]

FGENESH 2.6 Prediction of potential genes in Brassica_rapa genomic DNA
Seq name: ChrC01 44155518 44186776
Length of sequence: 31259
Number of predicted genes 8: in +chain 5, in -chain 3.
Number of predicted exons 28: in +chain 20, in -chain 8.
Positions of predicted genes and exons: Variant 1 from 1, Score:816.525781

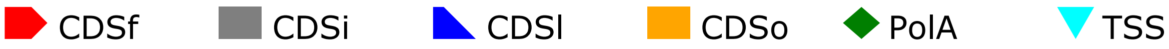

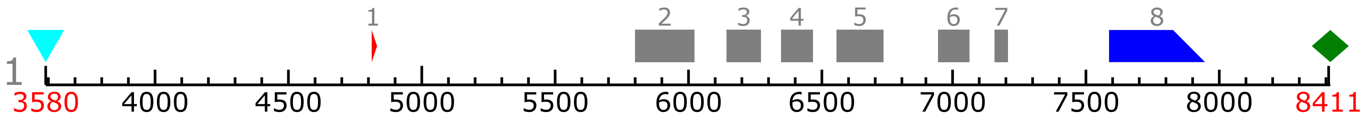

1	+		TSS	3580			-3.84				
1	+	1	CDSf	4806	-	4815	-5.73	4806	-	4814	9
1	+	2	CDSi	5797	-	6019	27.04	5799	-	6017	219
1	+	3	CDSi	6141	-	6270	26.71	6142	-	6270	129
1	+	4	CDSi	6346	-	6465	5.77	6346	-	6465	120
1	+	5	CDSi	6554	-	6730	28.09	6554	-	6730	177
1	+	6	CDSi	6935	-	7053	-0.59	6935	-	7051	117
1	+	7	CDSi	7149	-	7198	-5.42	7150	-	7197	48
1	+	8	CDSl	7579	-	7940	24.27	7581	-	7940	360
1	+		PolA	8411			1.87				

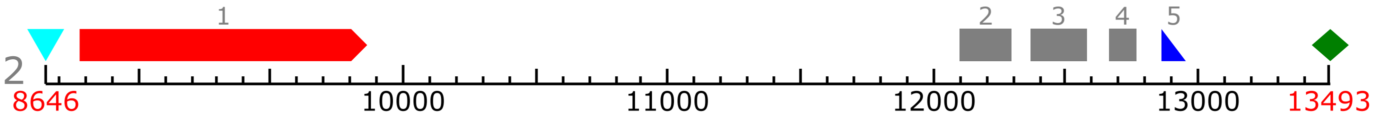

2	+		TSS	8646			-2.54				
2	+	1	CDSf	8774	-	9859	173.08	8774	-	9859	1086
2	+	2	CDSi	12095	-	12290	29.11	12095	-	12289	195
2	+	3	CDSi	12361	-	12574	22.92	12363	-	12572	210
2	+	4	CDSi	12658	-	12760	13.38	12659	-	12760	102
2	+	5	CDSl	12856	-	12948	11.02	12856	-	12948	93
2	+		PolA	13493			1.87				

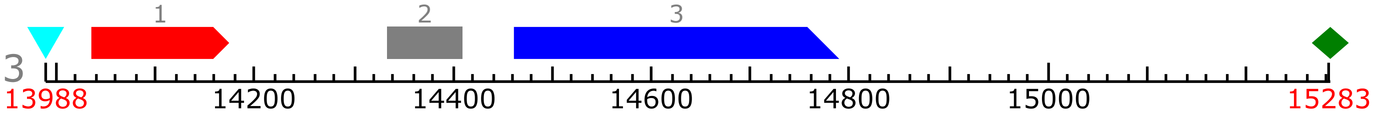

3	+		TSS	13988			-4.34				
3	+	1	CDSf	14034	-	14173	1.27	14034	-	14171	138
3	+	2	CDSi	14332	-	14408	0.08	14333	-	14407	75
3	+	3	CDSl	14460	-	14788	33.69	14462	-	14788	327
3	+		PolA	15283			1.87				

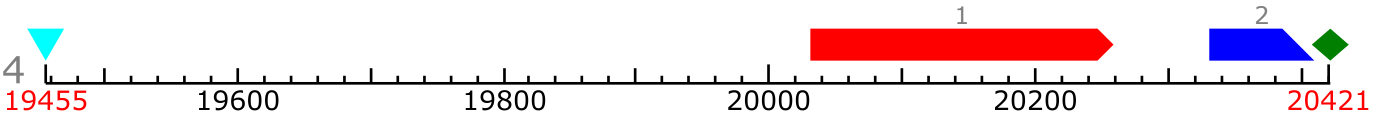

4	+		TSS	19455	-4.94		
4	+	1	CDSf	20030 - 20258	24.12	20030 - 20257	228
4	+	2	CDSl	20330 - 20409	-6.06	20332 - 20409	78
4	+		PolA	20421	-4.93		

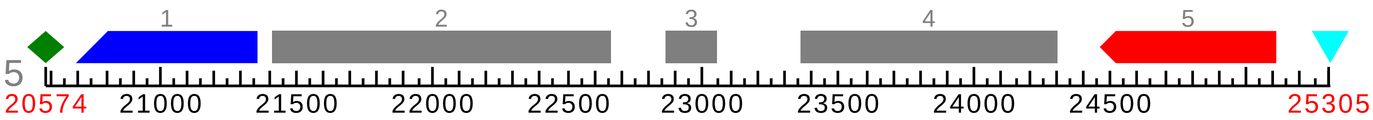

5	-		PolA	20574	-5.33		
5	-	1	CDSl	20685 - 21354	37.91	20685 - 21353	669
5	-	2	CDSi	21408 - 22655	88.92	21410 - 22654	1245
5	-	3	CDSi	22858 - 23046	4.35	22860 - 23045	186
5	-	4	CDSi	23354 - 24300	58.08	23356 - 24300	945
5	-	5	CDSf	24456 - 25106	87.54	24456 - 25106	651
5	-		TSS	25305	-2.94		

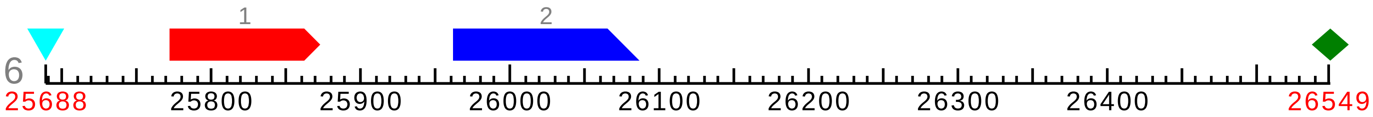

6	+		TSS	25688	-6.14		
6	+	1	CDSf	25771 - 25872	14.34	25771 - 25872	102
6	+	2	CDSl	25961 - 26086	11.77	25961 - 26086	126
6	+		PolA	26549	1.87		

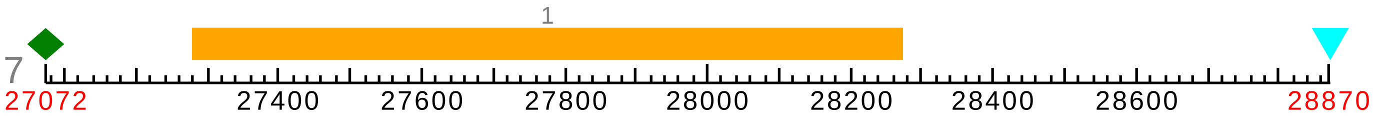

7	-		PolA	27072	1.87		
7	-	1	CDSo	27277 - 28272	170.85	27277 - 28272	996
7	-		TSS	28870	-2.14		

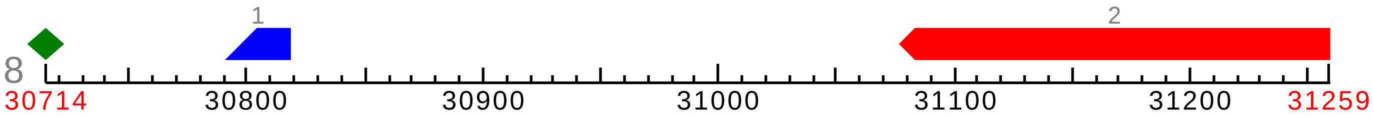

8	-		PolA	30714	1.87		
8	-	1	CDSl	30790 - 30818	0.48	30790 - 30816	27
8	-	2	CDSf	31076 - 31259	29.04	31077 - 31259	183

Predicted protein(s):
>FGENESH:[mRNA] 1 8 exon (s) 4806 - 7940 1191 bp, chain +
ATGGTTAAAGACCCGGCTTCGACAGCGTCGTGGTCGTGGTGGCACGAGGGAGCGATCCTC
GTGATTCCGCCAGTTGGATTCCGCTTAGCCCCACAGCTCTACCATACATCTCCTCTCCAT
TTCCGCTCCTGCTCCTCTCGCTCCCGCTCCTCTCGCTCCCGCTGCTGCACCCGCTCCTGC
TCCTCCGGGTCCTCCGGGAGTGATGAGTGTTGCGGAGTTGGTTCGACAGCCCGGTTCAAC

CGATCCGGGAACAGGATCAGCGCATGGATCAACCGTATGATGTACTCGGCCCTCAACAAG
GGACATCCGACTTTCACTGACTTCCCTACCGACAAGCAGCATATGTGGTTTCGTCAGTTT
GCGCAAGAATTCAACTGGAATTCCGATGATACGCTATTTATCTATCACCACTTCGTCCGT
AAAGTTATGGACAACTATGGGAAGCAAATGCACGAGTGGAAGAAGAAGTGGGAAATCAAC
AAAGTCCCAAAGTCGATGAACAACACGGTCTGGACGGAGTTGTGTGTGCATTGGGATAAG
GAAGAGACGAAAGAAACTTCTTCCACCAACTCCACCAACCGCATGAGCGACCGTAAAGGG
AAGGGCGTCTTCAAGCATAACTTGGGTGCTCAATCTATTGCCACTATGGGGGATCGCATG
TGGTCACCCTGGTCCAAACTCAGGTGCAAGACGAAGTGTCTCAGCTTCAAACCGAGGATG
ACGATTCGACGGCTTAGATCAACTTGTCCCGGTTTCGAATCAACGAAATCGTTGAATCGT
CGTTTCCAAAGAAGAAGTGACGTTTGGTCGGTTTGGGTCGTCCCCCCCGGAAATGAACCC
TCGGAAATTTCCGACGAACATTTCCTCGGAATAAGTAGTCGGAATATACCGAGGGACTCC
TTCCTCAGAATTTTCCGAGGGCTCCGTTCCTCGGAAATTTCCGATGAAAATTCCGAGGAA
CATTTCGTCGGAACTTCCGAGGATTGGACCACCGGAAAGTCCATCGAAATATCCCGAGGG
ACTCATTCCTCGGAATTTTCCGAGGGCTCCGTTCCTCAGAAATTCCCGATGAAAATTCTG
AGGAACATTTCGTTGGAACTTCCGAGGATTGGACCATCGGAAAGTCCATCGAAATATCCC
GAGGAAGTTCTCCCTCGATATATTCCGAGGACCTTTCCGACGAATTGGTGA
>FGENESH: 1 8 exon (s) 4806 - 7940 396 aa, chain +
MVKDPASTASWSWWHEGAILVIPPVGFRLAPQLYHTSPLHFRSCSSRSRSSRSRCCTRSC
SSGSSGSDECCGVGSTARFNRSGNRISAWINRMMYSALNKGHPTFTDFPTDKQHMWFRQF
AQEFNWNSDDTLFIYHHFVRKVMDNYGKQMHEWKKKWEINKVPKSMNNTVWTELCVHWDK
EETKETSSTNSTNRMSDRKGKGVFKHNLGAQSIATMGDRMWSPWSKLRCKTKCLSFKPRM
TIRRLRSTCPGFESTKSLNRRFQRRSDVWSVWVVPPGNEPSEISDEHFLGISSRNIPRDS
FLRIFRGLRSSEISDENSEEHFVGTSEDWTTGKSIEISRGTHSSEFSEGSVPQKFPMKIL
RNISLELPRIGPSESPSKYPEEVLPRYIPRTFPTNW
>FGENESH:[mRNA] 2 5 exon (s) 8774 - 12948 1692 bp, chain +
ATGGTGCTCCAACAACAACAACAAACGCTTTTTGTCACAAAGAAGACAGATCAAGAACTT
CCTCTTAAGACAGATCCAGAGCCTTCTCGTGAATTCATTTTCCGATCCAAACTTCCCGAC
ATCTCCATCCCTAATCATCTTCCTCTCACCGATTACGTCTTCCAGAAGTTCTCCGGCGAC
GGCGACGGACACTCCACAGCCACATGTCTCATCAACGGTGCCACCGGACGTATCTTTACC
TACGGTGATGTACAAATCAGTTTACGGCGTATCGCCGCCGGGATCCATCGGCTAGGGATC
CGCCAACGTGACACCGTCATGCTCCTTCTCCCAAACTCGCCGGAGTTTGCTCTCTCCTTC
CTCGCCGTCGCTTACCTTGGAGCCGTATCCACCACTGCGAATCCGTTCTATACAGAGTCG
GAGATCGAGAAACAGGCGAAAGCCTCAGCCACGAAGATGATCATCACTAAGTCATGTTAC
GTAGATAAACTTACAAACATGAAGAACGACAGCGTTTTGATCGTTTGCGTGGAAGATGAA
AACGACGCAGTTCCAGTAGCTAACGGTTGCGTGAGTTTCAAGGAACTGGCTCAAGCGGAC
GAAACAGAGCTCCCTAAACCGGAGATCTCACCGGAAGACACTGTAGCGATGCCGTACTCC
TCCGGAACCACGGGGCTTCCGAAGGGAGTGATGATCACTCACAAGGGATTAGTTACGAGC
ATCGCTCAGAAAGTAGACGGAGAAAACCCTAACGTCAACTTCACCGGAGATGACGTCATT
CTATGTTTCCTCCCTATGTTTCACATTTACGCGCTCGACGCGTTGATGCTCTCGGCTATG
AGGTCGGGTGCGGCGATCTTGATCGTTCCGAGGTTCGAGTTGAATCTGGTGATGGAGCTT
ATTCAGAGGTATAAGGTCACCGTGGTTCCGGTGGCTCCTCCGGTGGTTCTAGCGTTTGTT
AAGTCTCAGGAGACGGAGAGGTATGACCTGAGCTCAGTGAGGATGATGCTTTCAGGCGCA
GCTACGCTCAAGAAGGAGCTTGAAGACGCCGTGCGTCTCAAGTTTCCCAATGCCATATTT
GGACAGGGTTATGGAATGACTGAGTCAGGAACGGTGGCTAAGTCATTAGCGTTTGCAAAG
AACCCATTCAAGACCAAGTCTGGTGCGTGCGGGACTGTGATCAGAAACGCGGAGATGAAA
GTAGTCGACACCATAACCGGAGTCTCTTTACCACGCAACAAACCTGGCGAAATATGCATC
CGAGGTGATCAACTCATGAAAGGTTATTTGAATGATCCGGAGGCTACCGCAATGACCATA
GACAAAGACGGATGGTTACACACAGGAGATATTGGGTTCGTGGATGATGACGATGAGGTA

TTCATTGTTGATCGGTTAAAGGAACTTATCAAATTCAAAGGCTATCAAGTGGCTCCAGCT
GAGCTTGAAGCATTGCTTATTTCTCATCCTTGTATTGAAGATGCTGCAGTTGTCGCAATG
AAGGATGAAGTAGCTGATGAGGTTCCAGTAGCATTTGTGGTTAGATCAGAAGGGTCTCAG
TTAACCGAAGATGTCGTCAAGAGTTATGTCAACAAACAGGTGGTTCATTACAAGAGAATC
AAGATGGTGTTTTTCATAGAAGCTATACCTAAAGCAGTCTCGAAAGATATTGCGGAAGGA
ACTCCGAGCTAA
>FGENESH: 2 5 exon (s) 8774 - 12948 563 aa, chain +
MVLQQQQQTLFVTKKTDQELPLKTDPEPSREFIFRSKLPDISIPNHLPLTDYVFQKFSGD
GDGHSTATCLINGATGRIFTYGDVQISLRRIAAGIHRLGIRQRDTVMLLLPNSPEFALSF
LAVAYLGAVSTTANPFYTESEIEKQAKASATKMIITKSCYVDKLTNMKNDSVLIVCVEDE
NDAVPVANGCVSFKELAQADETELPKPEISPEDTVAMPYSSGTTGLPKGVMITHKGLVTS
IAQKVDGENPNVNFTGDDVILCFLPMFHIYALDALMLSAMRSGAAILIVPRFELNLVMEL
IQRYKVTVVPVAPPVVLAFVKSQETERYDLSSVRMMLSGAATLKKELEDAVRLKFPNAIF
GQGYGMTESGTVAKSLAFAKNPFKTKSGACGTVIRNAEMKVVDTITGVSLPRNKPGEICI
RGDQLMKGYLNDPEATAMTIDKDGWLHTGDIGFVDDDDEVFIVDRLKELIKFKGYQVAPA
ELEALLISHPCIEDAAVVAMKDEVADEVPVAFVVRSEGSQLTEDVVKSYVNKQVVHYKRI
KMVFFIEAIPKAVSKDIAEGTPS
>FGENESH:[mRNA] 3 3 exon (s) 14034 - 14788 546 bp, chain +
ATGAAGGGAGAGGACGAGGCGGGGCGGCAACATTTGACCAAGATTCGGACCTCCACTCGA
TTTGAGTATAAAAGGATGCTGCAAAGCTGTTGCGCAGACCATCTCTTAGGAGGATCACTC
TGCAAGCAACAAGAGATAAACGTACCCGTCGTAACGCCCGTGGTTCAGATCGGTGTTGAT
CTCTCGGGGCTCACGCGATGGTACGCGATAGTTTCCGGATGTCGAGATCTGGCGCCTCAC
GTTGTCGTCGTGGTTTCCGTAGATCACCCGAGGGCGAAGGGGCGGGAGGTGGGACGGTGG
ATCACACGTAGACCGTTCCTCCGGCGCCGCTTCCGATCGGTTCACGCTCTAGCTCGGATA
AGCTCTTGGCGGCGGAGATGTTGGTGGAGATCGCGGAAGAGGAGGCAGGAGCAGAGGAGG
AAGGAGGGAGGGAGTGGGACAGCGAGAGCCACGTCGCGGTTAGGGAGTGGTAGGCTGAGG
TCAGGACGTTTGTTTCGTCTCATGGCTTCTTCTTCTTCGTGTGATAATCACTCGACAAGG
GTTTGA
>FGENESH: 3 3 exon (s) 14034 - 14788 181 aa, chain +
MKGEDEAGRQHLTKIRTSTRFEYKRMLQSCCADHLLGGSLCKQQEINVPVVTPVVQIGVD
LSGLTRWYAIVSGCRDLAPHVVVVVSVDHPRAKGREVGRWITRRPFLRRRFRSVHALARI
SSWRRRCWWRSRKRRQEQRRKEGGSGTARATSRLGSGRLRSGRLFRLMASSSSCDNHSTR
V
>FGENESH:[mRNA] 4 2 exon (s) 20030 - 20409 309 bp, chain +
ATGACTAAACTGAAAAAGGAAACACGTTTTCAGGGTTATGGAATGACTGAGTCAGGAACG
GTGGCTAAGTCATTAGCGTTTGCAAAGAACCCATTCAAGACCAAGTCTGGTGCGTGCGGG
ACTGTGATCAGAAACGCGGAGATGAAAGTAGTCGACACCATAACCGGAGTCTCTTTACCA
CGCAACAAACCTGGCGAAATATGCATCCGAGGTGATCAACTCATGAAAGGTGATAGAGAT
AATCAGAGTTCTCTCTATAGGATCAAACTACCGTTCTCACAAACAATAGCTTGTAGCTCA
ATCGTATAG
>FGENESH: 4 2 exon (s) 20030 - 20409 102 aa, chain +
MTKLKKETRFQGYGMTESGTVAKSLAFAKNPFKTKSGACGTVIRNAEMKVVDTITGVSLP
RNKPGEICIRGDQLMKGDRDNQSSLYRIKLPFSQTIACSSIV
>FGENESH:[mRNA] 5 5 exon (s) 20685 - 25106 3705 bp, chain -
ATGGCGGAAACAAGATCGAAGGGAGCGGCGCGTGAAGCAGAAGGTTCGACAACAAATCGA
ATCGAAGCGATTGAGAAAGCGTTAGCGTTGCAGAATGAACGAGCATCGATAATGGATGAG
AGGCTGCAAACAATGCTCGAGGCGGTGAACGTGATGACGACGCAGATGCAACGGAACGCA
GCGACAAACGGAGAGATCAATCAGAGAGGCGGTGGTGAAGGTTTTGTTCAAGATCCGAAT

CATCGTCACCATCACAATAACAACTCTGGTATGACGAGAATGGCGAAGATTGATTTCCCA
AGATTTGATGGATCTAAATTGAAGGAGTGGTTGAGCAAAGCGGAAGAATTTTTCGAGATT
GCTAACACCCCTGAGGAGTGTAAAGTAGGAATCGCTTCCATTCACTTTGACGGAGAAGCA
TCTACTTGGCACTTAGCGTTGAAACAAGAAGATGAGAATGCGATGATCTTACGAAGCTGG
AGAGTGTATAAGAATCGAATAAAAGAAAGGTTTGAAGAAGTGTTGGATGATCCCATGGCG
GAGCTTAAAGAACTGAGGGAAACGGATGGAATTGCAGATTACCACAAGCGTTTCGAGCTG
ATTGAGCTCGACCGCCTATCTGAAGAGTACTTGTTGAGTGCGATCTTGCTGCAGAAAGGA
TTGATACCATTGAAGAAGGAAGAATGGGTGAAACCAAAGGAAACTGGAGGAAAGCTACGA
CCTTTTCTTTCACAAGCTGAAATGGCTGACAGAAGAGCAAAAGGTTTATGCTACTATTGT
GATGAGAAGTTTTCGCAAGAACATGCGTTGAAACATAGAAAAACGCAATTATATTCGATG
GATGTTGAGGAAACTACTGAGAGTGAAGAGTGGGAAGAAGAGGAAGCTGGTGAGAGGGAA
GTAGCTCAGATTTCGTTGAATGCAGTAATTGGAAGTACTGACTATACAACGATGAGGGTC
AGAGGTACACAAGGAAAAAAGAATCTGTACATTCTGCTCGACTCAGGATCAACACACAAC
TTCATTGACACAAGGATTGCAGATATTTTGGGGTGTAAGGTTGAACCTGCAGGTCGAAAA
CAAGTTGCAGTAGCTGATGGGAGCAAAATTGGGGTTTGTGGTAAAGTTAGCAACTTGCGT
TGGAACTTCATGAACACTGAGTTTCGAGCAGATTTTATGGTCATTCCATTGGGGTGTCAT
GACATTGTACTAGGCGTTCAGTGGTTATCAACTCTTGGTCCAATTACATGGGACTTTAAG
GAGCTTGAAATGTCTTTCAAATGGCACAACAAGAGGGTGATGTTACACGGGATCAAAGCA
GGATCCGTGAGGGAAGTCAAAGCCAAATGGTTAGAGAGTCGAAAGGAGGAAGAGATGCAG
CTTCACATGATATATGCGTGTGAAGAACCTGAAATGGAGTTATGGAGCATAAAAACAGAA
GCTGAAAACAGAGTCAATACGGAGGGAGAGTATGAGTTACAGAGGATATTGGAGGAGTAT
GGGAAAGTCTTTGAAGAACCTACGCAATTACCTCCATTTCGAGAGCATCACAATCACAAG
ATAGCTCTGATGGAGGGATCAAACCCAGTGAATCAAAGCTTTCAGAACTCACAGTGGGCA
TTACGAGTATTTGGTTATGCCATTCGGTCTCACAAATGCTCCAGCGACCTTTCGGGATTG
ATGAACCATGTGTTCAAAGATTATCTACGAAAGTTTGTTCTCATCTTTTTCGATGACATT
CTCATCTATAGTGCTACGGTGGAGGAGCATTTGAATCATTTCGTAAGAAACTATGTTAAA
GGCTACGGCATCATAGCTAGACCATTGACTGCTTTGACAAAGAAAGATGCTTACTGTTGG
AATGAGGAGGCACAAGAGTCATTCGTTAAATTGAAGAAAGCATTATGTTCTGCACCAGTT
TGGCATTACCACGCTGACTTACCATTTGTGGTTGAAACGGATGCGTGTAAGGAAGGTATT
GGTGCAGTACTTATGCAAGAAGGAAAGCCCCTGGCATACATCAGTAGACACTTAAAGGGC
AAGCAACTAAACCTTTCAATCTATGAAAAGGAGCTTCTGGCGGTTGTGTTTGCAGTGCAG
AAATGGAGGCATTATCTTCTCACTAACCACTTCATTATAAAGGCTGATCAGAGGAGTTTG
AAATATTTACTGGAGCAGAGGTTGAACACTCCGATACAGCAACAGTGGTTACCTAAGCTT
CTTGAGTTTGACTATGAAATTCAATACAAGCAAGGGAAAGATAATGTAGCAGCGGATGCG
TTATCTAGAGTGGAGGGAGCAGAGATTTTACACATGGCAATGTCAGTTCTTGATTGTGAC
CTTCTGACAAAAATACAAGAAGCGTATGCCAGTGATTCTAAAGCTCAAGAGATGATTGAG
AAGCTAAAAAATGAACCATCTACAGTAAAGAAGTACTCGTGGATCAATAATGTTCTGAGA
AGGGGAAGCAAGATAGTGGTCCCAGATGTAGTAGAGATCAAAAATATGATTTTGGAGTGG
CTACATTGTTCAAGCCAAGGAGGTCACTCAGGTCGTGAGGTGACAAGACAGAGAGTGAAA
GGGTTGTTTTACTGGAAGGGATTGATACGTGACATTCAAGCCTATCTCCGTAACTGCAGA
GTGTGTCAACAATGTAAATATGATGGGGCTGCTTCTCCTGGACTGCTACAACCTTTACCG
ATACCTGAAGCTGTGTGGGTGGACGTGTCAATGGATTTCATCGATGGTCTTCCACCATCG
TATGGGAAAACTGTCATCTTTGTGGTAGTAGATCGACTGAGTAAAGCAGCTCACTTTATT
GCATTGGCTCACCCTTATTCAGCAGCGTCGGTTGCTCAGGCATTTTTGGATAACGTCTAC
AAACTCCATGGCTTTCCGAGATCGATAGTCAGTGACAGAGACACAGTTTTCCTTAGTGAC
TTTTGGCAAGAGTTGTTCAAGTTGCAAGGCTGTTCTCTTAACATGTCAACAGCTTACCAT
CCTCAGAGTGATGGACAGACGGAGGTTGTCAATAGCAAATGGTTACCTTTGGCAGAATTT
TGGTACAACACTAACTATCATACAGCAACCCAAGTAACTCCGTATGAGGTTGTGTATGGC

CAAGCCCCACCAGTGCATCTACCTTATCTACCGGGTGAATCAAAAGTTCAGGTGGTAGCA
AAGTGTTTGGAGGATCGGGAAAAGATGTTGTTGTTATTGAAGTTTCATTTGCTACGCGCT
CAGCACCGAATGAAACAACAGGCTGACTTACATAGATCTGAGAGGAGTTTTCAGATAGGG
GATTGGGTCTATGTCAAGCTTCAGCCGTATCGACAACAGACCGTAGTACGGAGATCGAAT
GATAAGATTGTTCCCAAGTACTTTGGTCCTTACTGCATAATTGATAAGATGGGTGAGGTA
GCGTACAAACTTCGGTTTCCAACTGAGACGAGGGTCCATTCTGTTTTCCACGTTTCTCAG
TTGAAAAAAGCTGTGGGTGATGTGTCAACTTCTACGCAGCTACCTTCAATTGTGAATGAT
GACTATGTCAAGAAACCGGAGCTTATTCTGGAGCGTAAAATGGTGAAGCGGCAAGGACGA
GCAGCAACAATGGTGCTTGTACAGTGGAAAGGTCAAAATGCCGATGAAGCGACGTGGGAG
TATCTGTTTGACTTGCAGAAAAAGTTTCCTGATCTGGATCTATGA
>FGENESH: 5 5 exon (s) 20685 - 25106 1234 aa, chain -
MAETRSKGAAREAEGSTTNRIEAIEKALALQNERASIMDERLQTMLEAVNVMTTQMQRNA
ATNGEINQRGGGEGFVQDPNHRHHHNNNSGMTRMAKIDFPRFDGSKLKEWLSKAEEFFEI
ANTPEECKVGIASIHFDGEASTWHLALKQEDENAMILRSWRVYKNRIKERFEEVLDDPMA
ELKELRETDGIADYHKRFELIELDRLSEEYLLSAILLQKGLIPLKKEEWVKPKETGGKLR
PFLSQAEMADRRAKGLCYYCDEKFSQEHALKHRKTQLYSMDVEETTESEEWEEEEAGERE
VAQISLNAVIGSTDYTTMRVRGTQGKKNLYILLDSGSTHNFIDTRIADILGCKVEPAGRK
QVAVADGSKIGVCGKVSNLRWNFMNTEFRADFMVIPLGCHDIVLGVQWLSTLGPITWDFK
ELEMSFKWHNKRVMLHGIKAGSVREVKAKWLESRKEEEMQLHMIYACEEPEMELWSIKTE
AENRVNTEGEYELQRILEEYGKVFEEPTQLPPFREHHNHKIALMEGSNPVNQSFQNSQWA
LRVFGYAIRSHKCSSDLSGLMNHVFKDYLRKFVLIFFDDILIYSATVEEHLNHFVRNYVK
GYGIIARPLTALTKKDAYCWNEEAQESFVKLKKALCSAPVWHYHADLPFVVETDACKEGI
GAVLMQEGKPLAYISRHLKGKQLNLSIYEKELLAVVFAVQKWRHYLLTNHFIIKADQRSL
KYLLEQRLNTPIQQQWLPKLLEFDYEIQYKQGKDNVAADALSRVEGAEILHMAMSVLDCD
LLTKIQEAYASDSKAQEMIEKLKNEPSTVKKYSWINNVLRRGSKIVVPDVVEIKNMILEW
LHCSSQGGHSGREVTRQRVKGLFYWKGLIRDIQAYLRNCRVCQQCKYDGAASPGLLQPLP
IPEAVWVDVSMDFIDGLPPSYGKTVIFVVVDRLSKAAHFIALAHPYSAASVAQAFLDNVY
KLHGFPRSIVSDRDTVFLSDFWQELFKLQGCSLNMSTAYHPQSDGQTEVVNSKWLPLAEF
WYNTNYHTATQVTPYEVVYGQAPPVHLPYLPGESKVQVVAKCLEDREKMLLLLKFHLLRA
QHRMKQQADLHRSERSFQIGDWVYVKLQPYRQQTVVRRSNDKIVPKYFGPYCIIDKMGEV
AYKLRFPTETRVHSVFHVSQLKKAVGDVSTSTQLPSIVNDDYVKKPELILERKMVKRQGR
AATMVLVQWKGQNADEATWEYLFDLQKKFPDLDL
>FGENESH:[mRNA] 6 2 exon (s) 25771 - 26086 228 bp, chain +
ATGAAGGATGAAGTTGCTGATGAGGTTCCAGTAGCATATGTGGTTAGATCAGAAGGGTCT
CAGTTAACCGAAGATGATGTCAAGAGTTATGTCAACAAACAGGTGGTTCATTACAAGAGA
ATCAAGATGGTGTTTTTCATAGAAGCAATTCCGAAAGCAGTGTCCGGAAAGATTCTGAGG
AAGGAACTCCGAGCTAAACTGGAATCTGAGTACCCTAAACAGATTTAA
>FGENESH: 6 2 exon (s) 25771 - 26086 75 aa, chain +
MKDEVADEVPVAYVVRSEGSQLTEDDVKSYVNKQVVHYKRIKMVFFIEAIPKAVSGKILR
KELRAKLESEYPKQI
>FGENESH:[mRNA] 7 1 exon (s) 27277 - 28272 996 bp, chain -
ATGAGACGAAACAAACGTCCTGACCTCAGCTTACCACTCCCTAACCGCAACGTGGCTCTC
GCTGTCCCTCTCCCCCTCCCTCCTCCTCCATCCTCCTCCTCTGCTCTGGCCTCCTCTTCC
GCGATCTCCACCAACATCTCCGCCGCCAAGAGCTTATCCGAGCTAGAGCGCGTGAACCGA
ATCGGGAGCGGCGCCGGAGGAACGGTCTACAAAGTGATCCACCGTCCCACCTCGCGCCCC
TTCGCTCTCAAGGTGATCTACGGTAACCACGACGACAACGTGAGGCGCCAGATCTGCAGA
GAGATCGAGATCCTTCGGAGTGTTGACCACCCCAACGTCGTGAAATGCCACGACATGTTC
GATCACAACGGCGAGGTCCAGGTCTTGCTCGAGTTCATGGACAAAGGGTCCCTCGAAGGA

AGACACGTATCGCGAGAAGACGAGCTCGCTGATCTCACGCGCCAGATTCTCAGCGGCTTG
GCGTATATCCACCGCCGCCACATCGTCCACCGCGACATCAAACCGTCGAATCTTCTCATA
AACTCGGCCAATAACGTCAAGATTGCTGATTTTGGAGTGAGTCGGATCTTGGCGCAGACC
ATGGATCCTTGCAACTCCTCTGTCGGAACCATCGCTTACATGAGTCCCGAGAGGATCAAC
ATCGATCTTAATCACGGTCGTTACGACGGTTACGCGGGGGATATATGGAGTCTTGGTGTT
AGCGTGTTGGAGTTTTACTTGGGGAGGTTCCCTTTCGCTGTGAGTAGACAGGGTGACTGG
GCGAGTTTAATGTGTGCTATATGTATGACTCAGCCGCCGGAAGCTCCCGCGACGGCGTCT
GAGGAGTTTCGTCACTTCATCTCTTGTTGCTTGCAGAGTGATCCTCCTAAGAGATGGTCT
GCGCAGCAGCTTTTGCAGCATCCTTTTATAGTTAAATCAAGTGGTGGTCCGAATCTTCGT
CAAATGTTGCCGCCGCCGCCGCCTCCGGCGTCTTAG
>FGENESH: 7 1 exon (s) 27277 - 28272 331 aa, chain -
MRRNKRPDLSLPLPNRNVALAVPLPLPPPPSSSSALASSSAISTNISAAKSLSELERVNR
IGSGAGGTVYKVIHRPTSRPFALKVIYGNHDDNVRRQICREIEILRSVDHPNVVKCHDMF
DHNGEVQVLLEFMDKGSLEGRHVSREDELADLTRQILSGLAYIHRRHIVHRDIKPSNLLI
NSANNVKIADFGVSRILAQTMDPCNSSVGTIAYMSPERINIDLNHGRYDGYAGDIWSLGV
SVLEFYLGRFPFAVSRQGDWASLMCAICMTQPPEAPATASEEFRHFISCCLQSDPPKRWS
AQQLLQHPFIVKSSGGPNLRQMLPPPPPPAS
>FGENESH:[mRNA] 8 2 exon (s) 30790 - 31259 213 bp, chain -
ATGGCATCGACTGCGGCGGTTCCGTTCTGGAGAGCGGCGGGGATGACGTATATAACGTAC
TCAAACATCTGCGCGAATCTCGTCAGGAACTGTCTGAAAGAACCCTTCAAGGCCGAATCC
ATGAGCCGCGAGAAGGTTCACTTCTCCCTCTCCAAATGGGCCGATGGAAAGCCCCAGAAA
CCAGTTTTGCGCTCAGACGCACCTCAAGTTTGA
>FGENESH: 8 2 exon (s) 30790 - 31259 70 aa, chain -
MASTAAVPFWRAAGMTYITYSNICANLVRNCLKEPFKAESMSREKVHFSLSKWADGKPQK
PVLRSDAPQV
