# Supplementary figures and images for: Genome-Wide Identification Analysis of the 4-Coumarate: Coa Ligase (4CL) Gene Family in Brassica U’s Triangle Species and Its Potential Role in the Accumulation of Flavonoids in Brassica napus L
Source: Plants (Basel). 2025 Feb 26;14(5):714. doi: 10.3390/plants14050714 (PMC11902127; doi:10.3390/plants14050714)

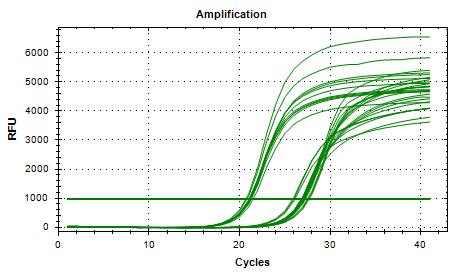

Supplement: Supplementary file 1 [file plants-14-00714-s001.zip › Supplementary Materials/Figure S2/Bna4CL1.8.12/Amplification-GH06.L1188.jpg]

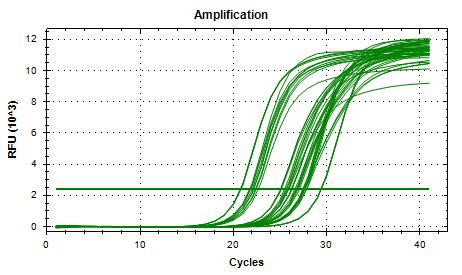

Supplement: Supplementary file 1 [file plants-14-00714-s001.zip › Supplementary Materials/Figure S2/Bna4CL1.8.12/Amplification-ZS11.ZY821.jpg]

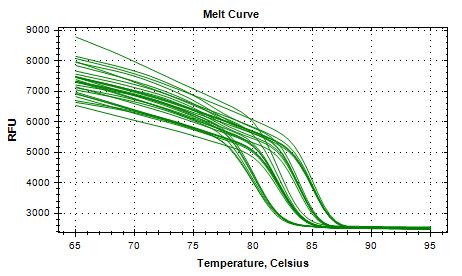

Supplement: Supplementary file 1 [file plants-14-00714-s001.zip › Supplementary Materials/Figure S2/Bna4CL1.8.12/Melt Curve-GH06.L1188.jpg]

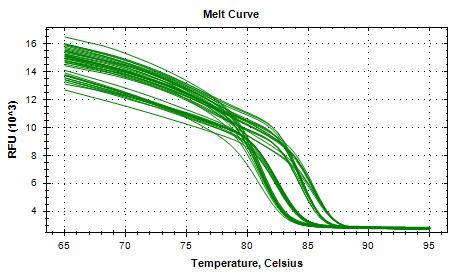

Supplement: Supplementary file 1 [file plants-14-00714-s001.zip › Supplementary Materials/Figure S2/Bna4CL1.8.12/Melt Curve-ZS11.ZY821.jpg]

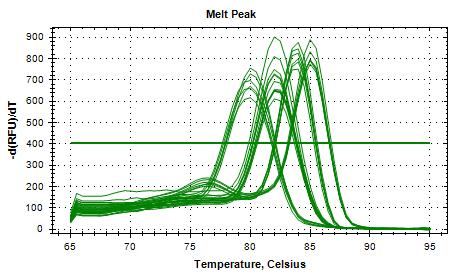

Supplement: Supplementary file 1 [file plants-14-00714-s001.zip › Supplementary Materials/Figure S2/Bna4CL1.8.12/Melt Peak-GH06.L1188.jpg]

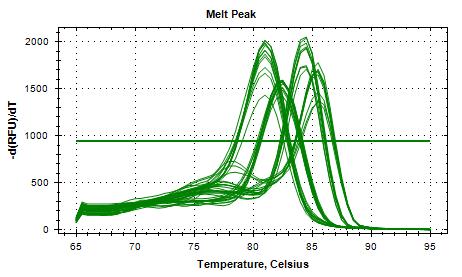

Supplement: Supplementary file 1 [file plants-14-00714-s001.zip › Supplementary Materials/Figure S2/Bna4CL1.8.12/Melt Peak-ZS11.ZY821.jpg]

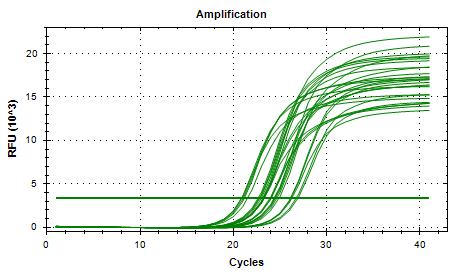

Supplement: Supplementary file 1 [file plants-14-00714-s001.zip › Supplementary Materials/Figure S2/Bna4CL3.4.7.11/Amplification-GH06.jpg]

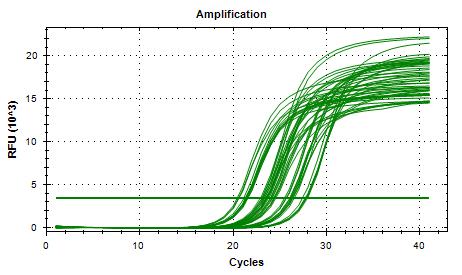

Supplement: Supplementary file 1 [file plants-14-00714-s001.zip › Supplementary Materials/Figure S2/Bna4CL3.4.7.11/Amplification-L1188.ZS11.jpg]

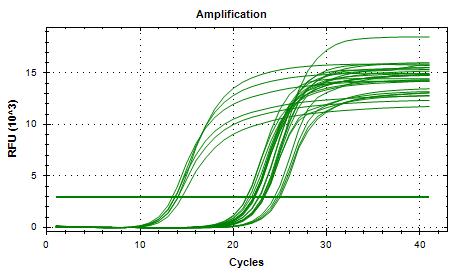

Supplement: Supplementary file 1 [file plants-14-00714-s001.zip › Supplementary Materials/Figure S2/Bna4CL3.4.7.11/Amplification-ZY821.jpg]

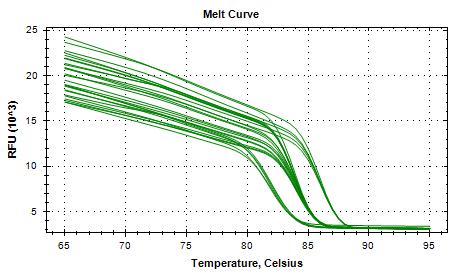

Supplement: Supplementary file 1 [file plants-14-00714-s001.zip › Supplementary Materials/Figure S2/Bna4CL3.4.7.11/Melt Curve-GH06.jpg]

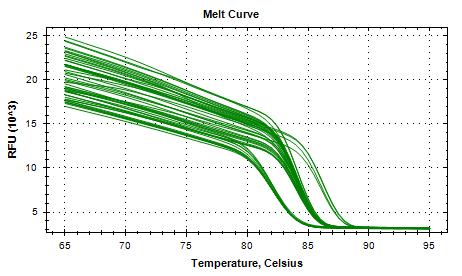

Supplement: Supplementary file 1 [file plants-14-00714-s001.zip › Supplementary Materials/Figure S2/Bna4CL3.4.7.11/Melt Curve-L1188.ZS11.jpg]

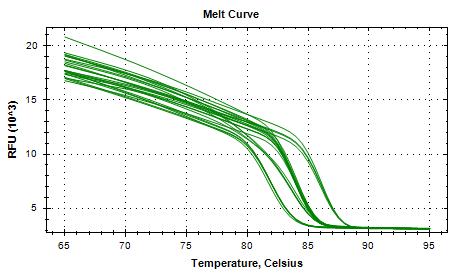

Supplement: Supplementary file 1 [file plants-14-00714-s001.zip › Supplementary Materials/Figure S2/Bna4CL3.4.7.11/Melt Curve-ZY821.jpg]

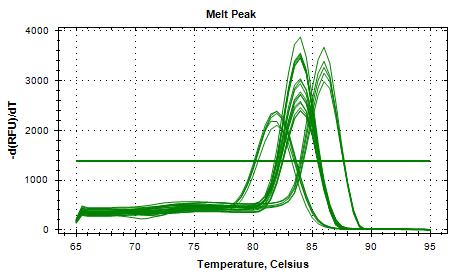

Supplement: Supplementary file 1 [file plants-14-00714-s001.zip › Supplementary Materials/Figure S2/Bna4CL3.4.7.11/Melt Peak-GH06.jpg]

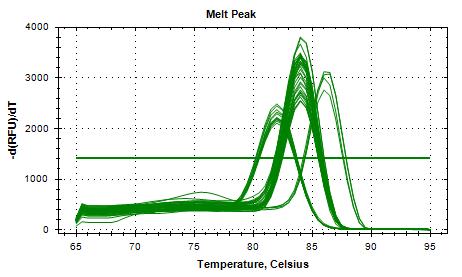

Supplement: Supplementary file 1 [file plants-14-00714-s001.zip › Supplementary Materials/Figure S2/Bna4CL3.4.7.11/Melt Peak-L1188.ZS11.jpg]

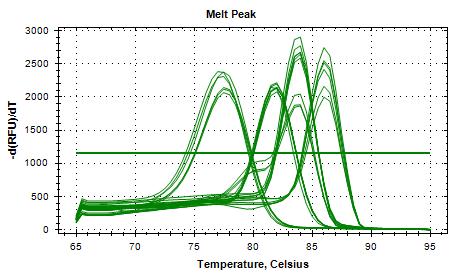

Supplement: Supplementary file 1 [file plants-14-00714-s001.zip › Supplementary Materials/Figure S2/Bna4CL3.4.7.11/Melt Peak-ZY821.jpg]

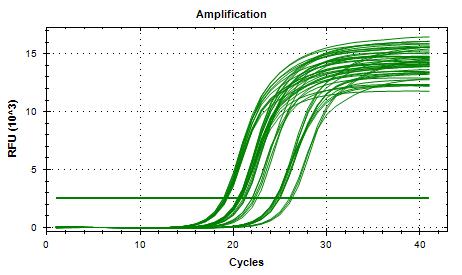

Supplement: Supplementary file 1 [file plants-14-00714-s001.zip › Supplementary Materials/Figure S2/BnaDFR/Amplification-GH06.ZY821.jpg]

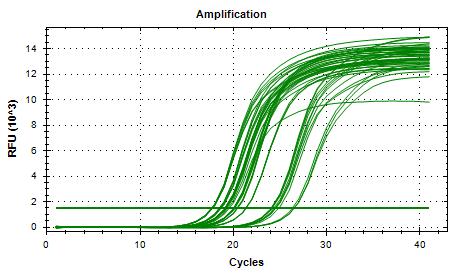

Supplement: Supplementary file 1 [file plants-14-00714-s001.zip › Supplementary Materials/Figure S2/BnaDFR/Amplification-L1188.ZS11.jpg]

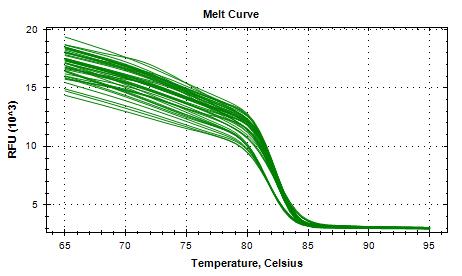

Supplement: Supplementary file 1 [file plants-14-00714-s001.zip › Supplementary Materials/Figure S2/BnaDFR/Melt Curve-GH06 ZY821.jpg]

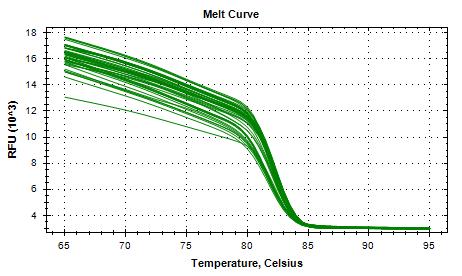

Supplement: Supplementary file 1 [file plants-14-00714-s001.zip › Supplementary Materials/Figure S2/BnaDFR/Melt Curve-L1188.ZS11.jpg]

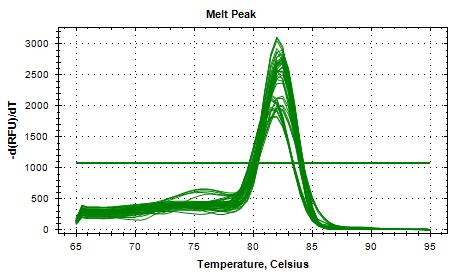

Supplement: Supplementary file 1 [file plants-14-00714-s001.zip › Supplementary Materials/Figure S2/BnaDFR/Melt Peak-GH06.ZY821.jpg]

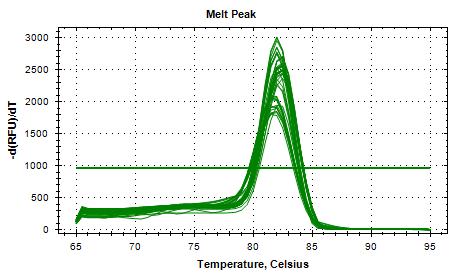

Supplement: Supplementary file 1 [file plants-14-00714-s001.zip › Supplementary Materials/Figure S2/BnaDFR/Melt Peak-L1188.ZS11.jpg]
